# Benzo[*c*][1,2,5]Thiadiazole Linkers Enhance the Effectiveness of Cavity‐Containing Supramolecular Photocatalysts

**DOI:** 10.1002/chem.71443

**Published:** 2026-08-03

**Authors:** Leonardo Amicosante, Dominic Taylor, Cameron L. Campbell, Daniel M. Lambden, Georgina M. Rosair, Filipe Vilela, Scott J. Dalgarno

**Affiliations:** ^1^ Institute of Chemical Sciences, Heriot‐Watt University Riccarton, Edinburgh UK

**Keywords:** benzo[*c*][1,2,5]thiadiazole, calixarenes, photocatalysis, singlet oxygen, supramolecular catalysis

## Abstract

A family of seven novel supramolecular photosensitizers comprising benzo[*c*][1,2,5]thiadiazole (**BTZ**) tethered calix[4]arene (C[4]) scaffolds has been synthesized using cross‐coupling strategies with selectively halogenated C[4]s, affording target compounds in high yield using facile purification. Their respective abilities to produce singlet oxygen has been evaluated through the photo‐oxidation of *α*‐terpinene, and structure–activity relationships established, probing factors such as cavity:photosensitizer ratio, distance of cavity to photosensitizer linker, and steric hindrance around the upper‐rim of the cavity. The presence of two C[4] cavities proximal to the photosensitizing **BTZ** unit provided vastly increased effectiveness in enhancing effective concentration through host:guest interactions, resulting in an eightfold increase in efficiency for the best‐performing member of the compound library benchmarked against a small‐molecule analogue, and reaching reaction completion in 1 h at 2.5 mol% loading. In contrast, systematically increasing the cavity:photosensitizing linker distance led to a reduction in improvement to 1.1‐fold relative to the small molecule analogue, confirming that these factors are pivotal in designing a suitable system based on the guest substrate.

## Graphical abstract

Seven novel supramolecular photosensitizers based on benzo[*c*][1,2,5]thiadiazole (**BTZ**) tethered to calix[4]arene (C[4]) scaffolds are reported with facile, scalable, and selective syntheses. Their abilities to produce singlet oxygen have been tested through the photo‐oxidation of *α*‐terpinene. Probing for different structural and electronic features, outstanding performance was observed for the catalyst with two C[4] cavities proximal to **BTZ**, providing an impressive eightfold enhancement in reactivity compared to its non‐cavity‐containing analogue.

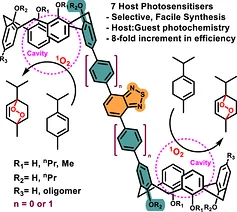

## 1 Introduction

Recent years have witnessed intense focus on the development of organic photosensitizers (PSs) able to produce singlet oxygen (^1^O_2_) [1, 2] for potential application in areas such as water and wastewater treatment [3], selective chemical synthesis [4], and antibacterial and selective cancer treatment through photodynamic therapy to name but a few [5, 6, 7]. It is well‐established that ^1^O_2_ can be produced photochemically from its ground triplet state (^3^O_2_) via intersystem crossing and triplet–triplet annihilation with a photo‐excited organic PS [1, 2], and there is abundant literature concerned with a plethora of organic dyes (such as methylene blue and rose Bengal) and their ability to produce ^1^O_2_ [8, 9]. Among them, synthetic chromophores including BODIPY [10, 11], acridines [12], and benzo[c][1,2,5]thiadiazoles (**BTZ**) [13, 14, 15] have become more popular due to their adaptability, with the latter receiving particular attention for its synthetic versatility and tuneability of optoelectronic properties granted by their electron donor–acceptor (EDA) type structure (Figure 1) [16]. This has ultimately led to widespread reports concerned with their incorporation in photoactive polymers, metal‐organic frameworks (MOFs), covalent organic frameworks (COFs) and 3D‐printed applications [14, 17, 18, 19, 20, 21, 22].

**FIGURE 1:**
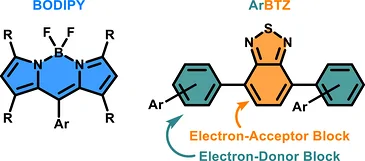
Structures of BODIPY‐ and **BTZ**‐based EDA photosensitizers (PSs), with teal and orange in the latter representing the electron‐donor and electron‐acceptor blocks, respectively.

Despite the abovementioned chromophores having been proven to successfully photosensitize ^1^O_2_ in solution, this process is inherently mass transfer limited by the amount of oxygen that can be transported to the vicinity of the PS itself, and therefore its local concentration [23]. To overcome this limitation, increasing the local effective concentration of substrate and oxygen in the immediate vicinity of the PS is one possible solution, drawing a parallel to increasing efficiency of the reactor or absolute amount of oxygen in solution [23]. This can be achieved by tethering an efficient PS such as **BTZ** onto a macrocyclic scaffold, noting that cavities can be utilized in the formation of host:guest (H:G) complexes. Many literature examples demonstrate the effectiveness of forming interactions between macrocycles and guests in alternative applications [17, 24, 25, 26, 27, 28, 29], and such interactions are deemed key as the host can be designed appropriately to suit a chosen strategy. For example, one can design or select a host with a shallow cavity that will form weak intermolecular interactions with a target guest, for example, through the formation of CH… π interactions, affording a system that has increased substrate occupancy while limiting the potential for cavity blocking through strong binding. Calix[4]arenes (C[4]s) have been utilized widely as supramolecular scaffolds in the literature for various applications due to their synthetic accessibility and ease of functionalization, for example in MOF synthesis and the subsequent use of H:G interactions for CO_2_ separation and catalysis [30]. Ongoing synthetic efforts on the C[4] framework continue to deliver novel hosts with fascinating physical properties, an excellent example being the synthetic transformation of a suitably functionalized C[4] to afford polyaromatic hosts (calix[4]pyrenes) as reported by Dobrovetsky, Vigalok and co‐workers [31].

Recent work in our group reported on the incorporation of the BODIPY PS moiety onto a number of C[4] hosts for photosensitization applications, operating through increased effective concentration between a substrate (α‐terpinene) that would scavenge ^1^O_2_ generated at the C[4] upper‐rim (Figure 2A) [32]. We specifically targeted weak H:G interactions in those BODIPY‐C[4]s to avoid product inhibition, and these photocatalysts were found to deliver up to a 28‐fold enhancement in the rate of consumption of ^1^O_2_ due to the presence of the cavity. Progression in the use of BODIPY‐C[4]s in this area was and remains hindered by the complex synthesis of the PS moiety, characterized by low yields, poor tuneability, and challenging and prolonged purifications, all of which limit overall applicability and potential utility of these materials on a wider scale.

**FIGURE 2:**
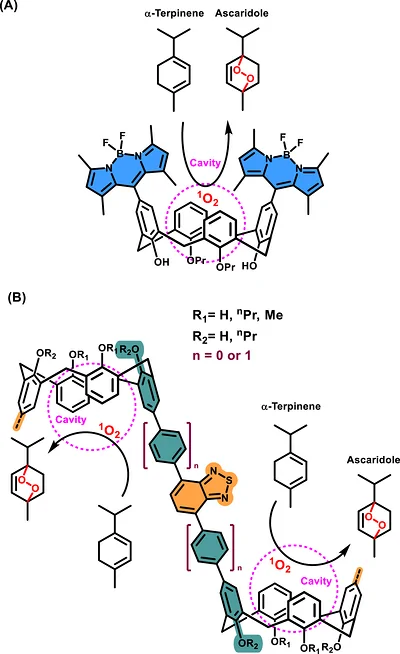
(A) Schematic showing the enhanced conversion of *α*‐terpinene to ascaridole via the use of BODIPY‐appended C[4] photosensitizers (PSs) that operate through enhanced effective concentration between guest substrate and ^1^O_2_ generated near the C[4] upper‐rim. (B) Schematic of new discrete/polymeric **BTZ**‐appended C[4] PSs for photosensitization and exploration of the effects outlined in the introduction, noting lower rim variations (R_1_ = H, ^n^Pr, or Me, and R_2_ = H or ^n^Pr) and distance between constituent cavities (with or without phenyl spacers present). Orange dashed line on upper rim indicates possible anchoring point for other **BTZ** units, that is for linear oligomer formation.

Noting our findings with BODIPY‐C[4]s, the synthetic benefits offered by the general **BTZ** system presented an exciting opportunity to build on the concept of enhanced concentration in PS‐appended C[4]s by (i) incorporating an additional cavity to evaluate cavity:PS ratio influence, (ii) evaluating the effects of C[4] lower‐rim substitution, (iii) forming oligomeric **BTZ‐C[4]** chains, and (iv) exploring the effects of systematically increasing the distance between the cavity and the PS moiety. Herein we report a series of **BTZ**‐appended multi‐C[4] systems (Figure 2B) that probe the effects of these systematic changes through careful synthetic design, binding studies and by evaluating photocatalytic performance using the benchmark ^1^O_2_ scavenger *α*‐terpinene. Our findings provide insight not just into the effects of varying cavity number and positioning, but also the future potential for application of these systems in photoactive materials and reaction‐ware, for example.

## 2 Results and Discussion

### 2.1 Synthesis

As previously reported, the attachment of BODIPY dyes to the upper‐rim of C[4]s accelerated the rate of *α*‐terpinene photo‐oxidation due to the presence of a cavity being accessible to the guest substrate [32]. From the BODIPY‐C[4]s studied, we were able to draw two key observations. First, it was observed that a single upper‐rim BODIPY chromophore was found to be more effective for photo‐oxidation than two, presumably as the second dye moiety hindered guest access to the cavity. Second, blocking the cavity through self‐inclusion of the BODIPY moieties effectively turned off the H:G chemistry/effect as expected. Notably, the conformation and shape of C[4]s is known to be heavily influenced by the lower‐rim substitution pattern [33]. In our previous study we solely investigated C[4]s that featured a distal lower‐rim dipropoxy substitution pattern. C[4]s with this substitution pattern are known to adopt a pseudo‐*C_4v_* “cone” conformation due to two lower‐rim H‐bonding interactions between the hydroxyl groups and the ethereal oxygens, as opposed to the typical “pinched‐cone” conformation (with *C_2v_* symmetry) adopted upon full lower‐rim alkylation [33]. Therefore, to further our understanding of this phenomenon, we sought to investigate the impact of various structural features on this cavity‐driven acceleration effect. These design features included (i) the ratio of cavity to PS, (ii) the shape of the cavity (which can be tailored via controlled C[4] lower‐rim substitution, for example, from cone to pinched cone via controlled lower‐rim alkylation) [33], and (iii) the distance between the PS and the cavity/cavities. **BTZ**‐based EDA systems presented themselves as ideal candidates for exploring these areas due to their synthetic versatility, multiple anchoring points for donor blocks (which permit advantageous coupling to two cavities), and smaller size which could present an improvement to the detrimental self‐blockage of the cavity found with BODIPY chromophores.

To synthesize **BTZ** analogues of the aforementioned BODIPY‐C[4] PSs [32], the first structure targeted involved the introduction of two **BTZ** cores at the upper‐rim of a cavity‐containing C[4] scaffold (**2B1C,** Scheme 1), noting that the presence of two lower‐rim OH groups would lead to a more accessible cavity for potential guest occupation. The synthesis of **2B1C** necessarily involved the attachment of an non‐symmetric **BTZ** to the C[4] scaffold, in this case **Br‐BTZ‐Ph** (Scheme 1, i), and successive attachment was achieved via a convenient one‐pot, two‐step strategy (Scheme 1, ii) involving Miyaura borylation of **Br‐BTZ‐Ph** using bis(pinacolato)diboron followed by Suzuki–Miyaura cross‐coupling with 5,17‐dibromo‐26,28‐dipropyloxycalix[4]arene.

**SCHEME 1:**
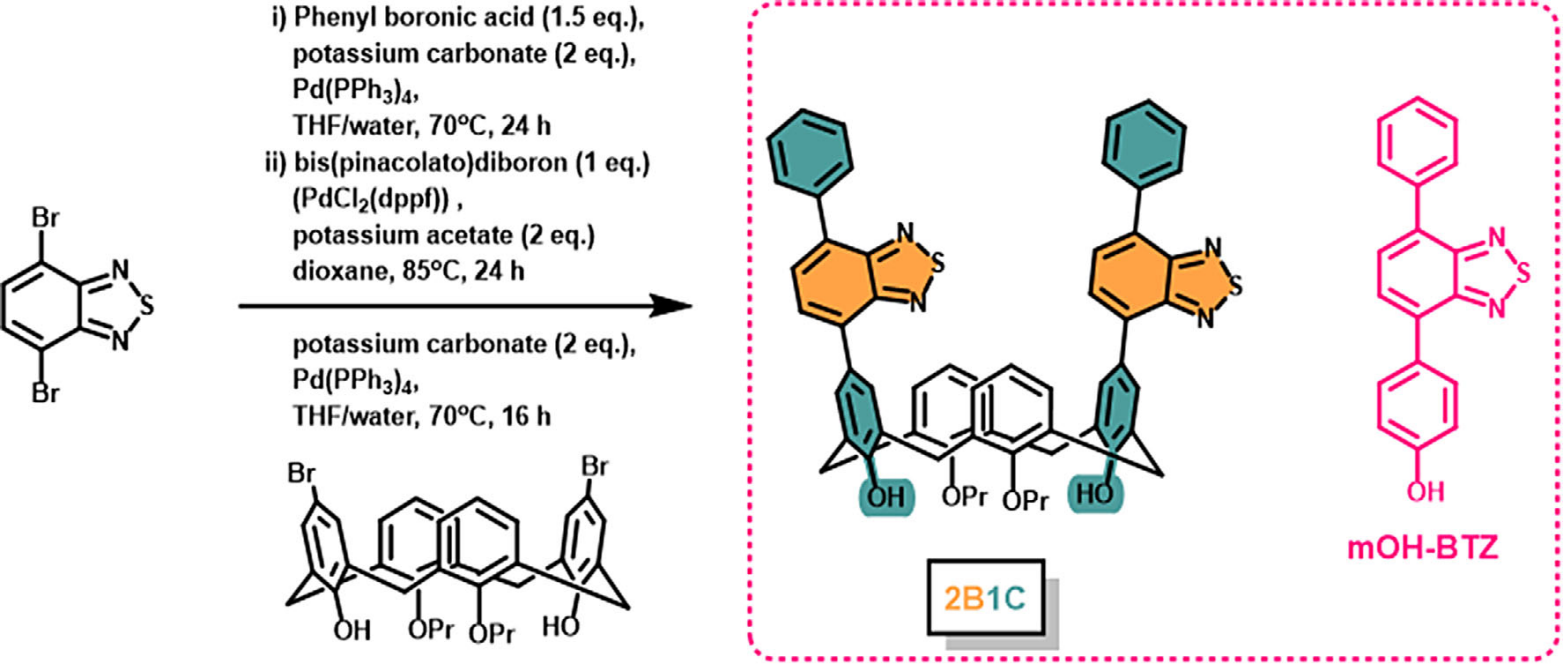
Synthetic procedure and structure of **2B1C**, which is structurally and electronically related to non‐symmetric **mOH‐BTZ**. Teal and orange segments in **2B1C** represent electron‐donor and electron‐acceptor blocks, respectively.

While the final step in the synthesis of **2B1C** proceeded smoothly, isolation of the non‐symmetric **BTZ** starting material was more challenging due to the multistep nature of the synthetic route employed (see Supporting Information). Thankfully all other compounds reported in this contribution involved the facile tethering of two or more C[4]s in a symmetric manner, thus allowing us to exploit a symmetric 4,7‐disubstituted **BTZ** for analogous coupling chemistry. All other compounds described below therefore involve two or more C[4] scaffolds coupled through upper‐rim positions, introducing an additional cavity located near each **BTZ** moiety as shown in Figure 3. The syntheses of the majority of the **BTZ‐C[4]s** below were achieved via general Suzuki–Miyaura cross coupling [34], for example **1B2C** (Figure 3) that was prepared by reaction of mono‐brominated dipropoxycalix[4]arene with the commercially available 2,1,3‐benzothiadiazole‐4,7‐bis(boronic acid pinacol ester) (**BTZ‐BPin**, see Supporting Information). Inspection of Scheme 1 and Figure 3 shows the structural relationship between **2B1C** and **1B2C**, important features of which are: (i) **BTZ** coupling at the C[4] upper‐rims, and (ii) retention of the lower‐rim dipropoxy functionality to promote solubility while preserving the cavity for potential guest occupation.

**FIGURE 3:**
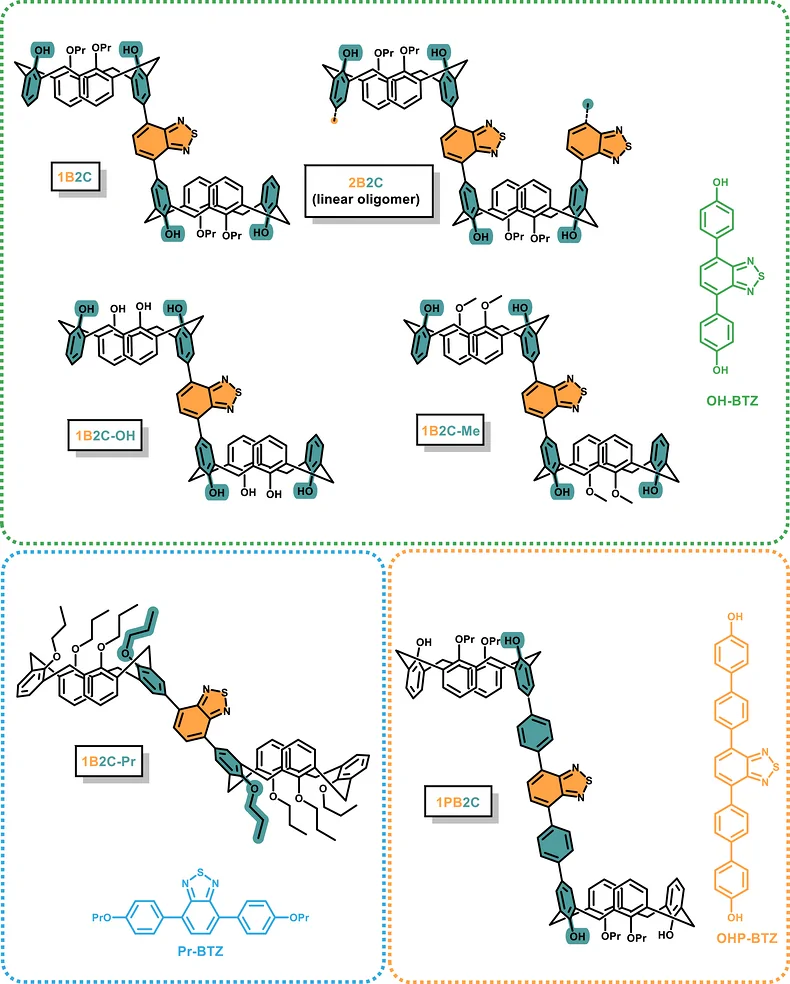
Structures of **BTZ‐C[4]** photosensitizer library members that are accessed via a symmetric 4,7‐disubstituted **BTZ** moiety. All structurally and electronically related to molecular **OH‐BTZ** are grouped in the green dotted perimeter. Structure of **1B2C‐Pr** (drawn to emphasize the pinched‐cone conformation adopted by the tetrapropoxy‐C[4] moieties) is structurally and electronically related to molecular **Pr‐BTZ**, in dotted blue perimeter. **1PB2C** (showing the insertion of phenyl spacers at the upper‐rim of each C[4] moiety) and the structurally and electronically related **OHP‐BTZ** are represented in dotted orange perimeter. For all BTZ C[4] structures, the teal and orange segments represent electron‐donor and electron‐acceptor blocks, respectively.

Performing the same reaction with the C[4] scaffold employed in the synthesis of **2B1C** afforded a linear oligomer, **2B2C**, due to the symmetric **BTZ‐BPin** linker being combined with 5,17‐dibromo‐26,28‐dipropoxycalix[4]arene (Figure 3). Since each reagent presented two coupling sites, this led to the formation of a short‐chained linear oligomer, where the repeating unit comprises one C[4] and one **BTZ**, corroborated by broad ^1^H NMR signals and gel‐permeation chromatography (GPC, see Supporting Information). To produce **1B2C** (Figure 3), instead, a mono‐brominated di‐propoxycalix[4]arene was to be synthesized using the existing literature route reported by Arduini et al. (45% reported yield) [35]. This, however, was found to lead to a tedious isolation and low yields, due to the unselective bromination and the challenging separation combined with the poor solubility of the crude mixture. To overcome this bottleneck, a synthetic strategy was devised to protect one of the two hydroxyl sites of the lower rim, as the hydroxyl groups activate the upper‐rim positions toward bromination (Scheme 2) [35]. The first step involved the mono‐benzoylation of the lower rim of 25,27‐dipropoxycalix[4]arene using 1 eq. of nBuLi at −78°C to selectively deprotonate one hydroxyl group prior to the addition of 1 eq. of benzoyl chloride. Subsequent bromination was achieved using NBS (1.1 eq) in the presence of catalytic amounts of azobisisobutyronitrile (AIBN) [35]. Lastly, the deprotection step was adapted from the procedure reported by Gutsche et al. [36] and involved an overnight reflux of a THF solution of the starting material with ethanol and a 70% NaOH aqueous solution (Scheme 2).

**SCHEME 2:**
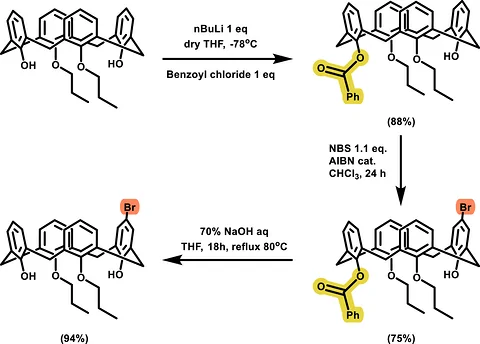
New synthetic route to 5‐bromo‐26,28‐dipropoxycalix[4]arene.

Overall, the total yield for the synthetic route employed was calculated to be 62% which is substantially higher than any direct mono‐bromination route previously attempted by the authors’ research group and the route reported by Arduini et al. (45%) [35]. Moreover, it is important to note that, since all the abovementioned synthetic procedures emerged as being highly selective, the purification required for each step was reduced to simple recrystallization or even merely washing in counter solvent such as methanol, leading to a more seamless process and an attractive alternative for scale up if desired. Once 5‐bromo‐26,28‐dipropoxycalix[4]arene was obtained, Suzuki–Miyaura cross‐coupling to afford **1B2C** was facile, utilizing **BTZ‐BPin** (1 eq.) and 5‐bromo‐26,28‐dipropoxycalix[4]arene (2.05 eq.).

As mentioned above, the shape of the cavity is influenced by H‐bonding interactions at the C[4] lower‐rim [33]. As such, analogues of **1B2C** featuring four hydroxyl groups (**1B2C‐OH**), two hydroxyl and two methoxy (**1B2C‐Me**), and four propoxy groups (**1B2C‐Pr**) were prepared using the same approach as for **1B2C** from their corresponding mono‐halogenated C[4], thus allowing us to explore the effects of different lower‐rim substitutions on activity (vide infra). Compound **1B2C‐OH** (Figure 3) was synthesized following a similar strategy as for **1B2C**, by protecting three lower‐rim sites to direct selective monobromination. This was achieved following the literature procedure from Lee et al. [37], which involved stirring the C[4] starting material in pyridine in an ice bath prior to the addition of benzoyl chloride (83%, Scheme S2). Following protection, the bromination step was followed as reported by Vézina et al. [38], which afforded selective and full conversion for the desired product (92%–95%). Following this, the same deprotection procedure was utilized to obtain the desired 5‐bromocalix[4]arene‐25,26,27,28‐tetrol, which was utilized for the final Suzuki–Miyaura cross‐coupling reaction with **BTZ‐BPin**. Conversely, **1B2C‐Me** (Figure 3), was derived from 5‐iodo‐26,28‐dimethoxycalix[4]arene, which was directly synthesized using literature procedures from Hennrich et al. [39] Similarly, **1B2C‐Pr** (Figure 3) was derived from the direct mono‐bromination leading to 5‐bromo‐25,26,27,28‐tetra(n‐propoxy)calix[4]arene, following the literature procedures from de la Vega et al. and previous work from the authors’ group [40].

Finally, to investigate the effect of increasing the distance between the C[4] cavity and the **BTZ** PS, while keeping the dual cavity system in cone conformation, an analogue of **1B2C** incorporating an additional phenyl spacer between the **BTZ** and C[4] moieties was synthesized (**1PB2C**, Figure 3). This was achieved by Miyaura borylation of 5‐bromo‐26,28‐dipropoxycalix[4]arene with 1.5 eq. of bis(pinacolato)diboron (B2Pin2), potassium acetate and Pd(dppf)Cl_2_, followed by a Suzuki–Miyaura cross‐coupling with 4,7‐bis(4‐bromophenyl)benzo[c][1,2,5]thiadiazole (**Br‐PhBTZ**).

It should be noted that the photoactive **BTZ** group varies slightly between each of the target C[4] PSs discussed above. As such, direct comparison between library members is not possible as there could be optoelectronic or photophysical explanations for differences in performance. To account for this for each C[4] PS, the small molecule analogues shown in Scheme 1 and Figure 3 (**OH‐BTZ**, **mOH‐BTZ**, **Pr‐BTZ**, **OHP‐BTZ**) were prepared as they comprise the same combination of electron donor groups without the C[4] cavity present. It is therefore the relative performance between each molecular/supramolecular PS combination that we discuss herein.

### 2.2 Photophysical Properties

Among photophysical properties of interest for any PS, in this study the focus was directed on the investigation of UV‐visible absorption (*λ*_abs_) and emission maxima (*λ*_em_), Stokes’ shifts, and absolute photoluminescence quantum yield (PLQY). The *λ*_abs_ of the **BTZ‐C[4]** PS library lay in a range between 384 and 432 nm (see Table 1 and Supporting Information for normalized spectra). Overall, a bathochromic shift of 10–28 nm is observed for most members of the library with respect to their corresponding molecular **BTZ** counterparts (e.g., **OH‐BTZ** *λ*_abs_ = 404 nm) [34], with **1B2C** and **2B2C** showing the greatest bathochromic shift, and **1B2C‐Me** the smallest. However, it is noticeable that **1B2C‐OH** instead presented a hypsochromic shift in absorption of 20 nm. This same trend is followed for *λ*_em_ as overall the **BTZ‐C[4]** members show similar Stokes’ shift compared to their molecular counterparts. Hence, *λ*_em_ ranges between 503 and 560 nm (Table 1 and Supporting Information for normalized spectra).

**TABLE 1:** Summary of photophysical and optoelectronic properties of the **BTZ**‐appended C[4] library reported here. All measurements were taken in CHCl_3_.

BTZ PS	*λ*_abs_ (nm)	*λ*_em_ (nm)	Stokes' shift (nm)	PLQY (%)^a^	*K*_sv_
**1B2C**	432	554	122	69.5	6.797 × 10^4^
**2B2C**	431	554	123	43.6	6.789 × 10^4^
**1PB2C**	415	538	123	70.4	1.438 × 10^4^
**1B2C‐Pr**	416	534	118	90.1	6.165 × 10^4^
**1B2C‐Me**	414	533	119	30.5	6.181 × 10^4^
**1B2C‐OH**	384	503	119	54.0	6.259 × 10^4^
**2B1C**	413	534	121	91.5	5.190 × 10^4^

^a^ Absolute PLQY.

This variation could be connected to the change in electron‐donating ability of the donor blocks, as reported by Taylor et al. [16, 34], which in each case is partially or fully lying on the C[4]. More specifically, this might be associated with how the electron donor part of the C[4] that is conjugated to the **BTZ** unit interacts with the rest of the C[4] it belongs to. However, as conjugation to the rest of the C[4] is broken due to the presence of the methylene bridges which are left untouched in the series of compounds, this effect could be mainly linked to the difference in the lower rim H‐bonding between different PSs, for example, the number of hydroxyl groups present. An empirical trend can be drawn from the lower‐rim differences between these PSs, starting from two hydroxyl and two propoxyl groups (e.g., **1B2C**, **2B2C**) showing a larger bathochromic shift of 28 nm; two hydroxyl and two methoxy groups (**1B2C‐Me**) showing a smaller bathochromic shift of 10 nm; and finally four hydroxyl groups (**1B2C‐OH**) showing a hypsochromic shift of 20 nm, due to maximum lower‐rim H‐bonding and therefore less electronic donation from the *para*‐OH of the electron‐donor block into the **BTZ** electron‐acceptor block core. This can be corroborated by the other members of the library, such as **2B1C** and **1PB2C**, which have similar bathochromic shift to **1B2C** as they also comprise two hydroxyl and two propoxyl groups on the lower rim. The absolute PLQY was also measured following the conventions and considerations from Jones et al. [41] The results of the measurements showed an overall wide range, from the lowest **1B2C‐Me** with 30.5% to the highest being **1B2C‐Pr** and **2B1C**, with 90.1% and 91.5%, respectively. This is in line with previously reported **BTZ**‐based molecules and, overall, there is an observable decrease in PLQY upon the formation of **BTZ‐C[4]**, for example, **OH‐BTZ** presents literature value of 89.1%, while its **BTZ‐C[4]** equivalents **1B2C**, **2B2C**, **1B2C‐Me**, and **1B2C‐OH** span between 69.5% and 30.5% (Table 1) [34].

### 2.3 Binding Studies via Stern–Volmer Fluorescence Quenching

The most convincing quantitative indication of affinity and weak/labile binding between the host PSs and the *α*‐terpinene guest is the Stern–Volmer constant (*K*_sv_). Recent literature studies on neutral guest sensing using C[4] derivatives as hosts demonstrated the use of *K*_sv_ as a quantitative measurement of the affinity between the substrate and the C[4] itself [42, 43, 44]. *K*_sv_ can be obtained by monitoring the emission intensity change of the host PSs in a gradually increasing presence of quencher (*α*‐terpinene) as depicted by a Stern–Volmer graph, that can be drawn by plotting *I*_0_/*I* (where *I*_0_ is the initial intensity and *I* is the intensity of each quenched solution) versus the concentration of the quencher ([*α*‐terpinene]/M) on the abscissa (Section 7 of the Supporting Information). The gradient of the linear plot of the resulting points is defined as *K*_sv_ and magnitude varies greatly depending on the affinity between host and guest. The value of *K*_SV_ can range from 10^2^ for very weak binding to 10^6^ for stronger binding [42, 43, 44].

The library of **BTZ‐C[4]** PSs reported here shows H:G binding properties in the same order of magnitude compared to the BODIPY‐C[4], which was also in the order of ×10^4^ (Table 1). The calculated *K*_sv_ for the library members in this study varied from 6.80 × 10^4^ for **1B2C** to a minimum of 1.44 × 10^4^ for **1PB2C**. This still represents a binding of modest strength compared to literature sensors; however, it is slightly higher than those found for BODIPY‐C[4] for the cases of **1B2C**, **1B2C‐Me**, **1B2C‐OH**, **1B2C‐Pr**, and **2B2C**. This variation can be attributed to the effect of the dual cavity tethered to each **BTZ**, thus increasing the ability of each host PS to enhance effective concentration with the substrate. Compound **2B1C** presents one host cavity and two **BTZ** photoactive sites, and the *K*_sv_ is found to be second lowest, at 5.19 × 10^4^ (Table 1). The third lowest *K*_sv_, although not by much, was found to be **1B2C‐Pr**, and like the previous one, this can be attributed to a structural feature, namely the two C[4] moieties being in pinched‐cone rather than cone conformations. For comparison, analogous plots were drawn for control for **BTZ** PSs (section 7 of the Supporting Information), which showed a significantly lower *K*_sv_ within the range of 1.77–3.00 × 10^3^, which is attributed to the total lack of a cavity for substrate occupation. Furthermore, a final control test consisted of the measurement of *K*_sv_ for **1PB2C**. In fact, with its cavities lying farther away from the central **BTZ** core, it was possible to observe a low *K*_sv_ of 1.44 × 10^4^ (Table 1), resulting in the lowest binding of the library, with over three times smaller *K*_sv_ compared to **1B2C** and only one order of magnitude greater than the literature non‐C[4] **BTZs**. This corroborates the validity of the measured *K*_sv_ values and underlines the importance of a cavity (or two) vicinal to the photoactive species, which otherwise serves less purpose for photochemical affinity.

These *K*_sv_ measurements can serve as further evidence for the enhancement of effective concentration of substrate at the cavity site, adding onto the previous proof of this effect [32]. The previous report underlined that the increased concentration arises as a consequence of labile temporary binding of the substrate within the cavity of the C[4] host, which increases the likelihood that substrate guest is close to the photocatalytic site during any photoexcitation event. It follows that any reactive oxygen species (such as ^1^O_2_) produced by **BTZ** are far more likely to react with the substrate before eventual relaxation back to their unreactive ground state, since they are inherently proximal to the host cavity [16, 32]. This was further validated by the insights provided by Michaelis–Menten analysis and the comparison of non‐cavity **BTZ** PSs which present severely lower *K*_sv_ (1.77–3.00 × 10^3^) and reactivity (vide infra).

### 2.4 X‐Ray Crystallographic Structures

Single crystals of **1B2C**, **2B1C**, and **1B2C‐Pr** were successfully grown via slow evaporation from chloroform/methanol and subsequently analyzed as outlined in the Supporting Information. Inspection of the crystal structures corroborates the inferences outlined previously regarding (i) cavity conformation and shape and (ii) cavity access hindrance. First, it confirms (as expected) that cavity conformation and shape is controlled by H‐bonding interactions where these are present, aligning observations from the solid state with those in solution.

Single crystals of **1B2C‐Pr** were found to be of two different morphologies, and analysis showed these to be where the C[4] phenyl rings tethered to the **BTZ** PS linker are either splayed or pinched within the overall pinched cone conformation. Fluctuation between these two extremes is well understood in solution, but observation of both arrangements was unexpected. The diffraction data for the extended/splayed version shown in Figure 4 were of good quality and allowed for full structural analysis. In contrast, the data for the compact/pinched version were found to be badly twinned and it was not possible to arrive at an acceptable disorder model, therefore only a partial structure is included in the Supporting Information along with unit cell parameters (Figure S16). As can be seen in Figures 4 and S16, the C[4] cavities are closed off in both solid‐state arrangements, and in the latter the molecule is particularly crowded around the **BTZ** PS linker. Fluctuation of the aryl groups is well‐documented for tetra‐alkylated C[4]s, and measuring the distance between the upper rim carbon pairs shows these to be in line with those in literature [45]. Both structures of **1B2C‐Pr** are markedly different to those of **1B2C** and **2B1C** as the latter possess the key cavity‐stabilizing lower‐rim hydroxyl groups.

**FIGURE 4:**
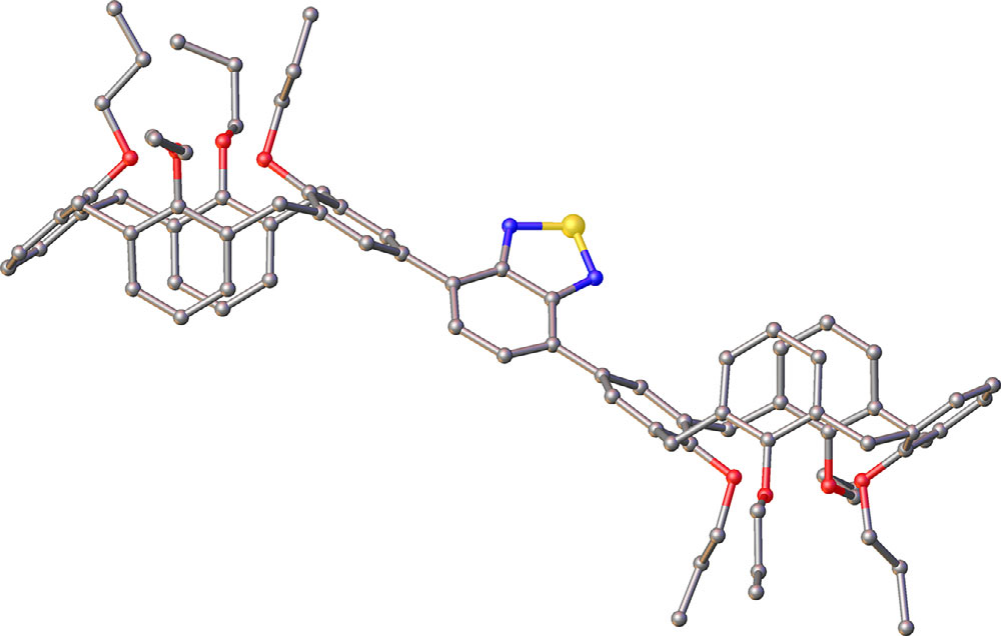
Single crystal x‐ray structure of **1B2C‐Pr** in ball‐and‐stick representation showing the **BTZ** PS linker tethered to aryl rings that are in the splayed positions within their respective C[4] pinched cone conformers. Color code: C—gray, N—blue, O—red, S—yellow. H atoms omitted for clarity.

The single crystal x‐ray structures of **2B1C** and **1B2C** are shown in Figure 5A and Figure 5B, respectively. Inspection of both structures confirms that the constituent C[4]s are all in the cone conformation, stabilized by lower‐rim H‐bonding interactions shown as dashed red lines. As anticipated (based on observations for BODIPY analogues), the structure of **2B1C** (Figure 5A) displays greater crowding around the C[4] cavity due to the presence of the second **BTZ** PS moiety at the distal upper‐rim position, in this case with one **BTZ** pointed toward the cavity interior. The positions of the **BTZ** cores are expected to fluctuate in solution and therefore vary between open and closed positions, however, this evidently represents a greater hinderance to cavity access on average compared to the structure of **1B2C** where only one **BTZ** is present per C[4] unit (Figure 5B). In fact, for **1B2C** (and other dual‐cavity analogues), self‐inclusion of the **BTZ** unit for one cavity renders the other unhindered, and therefore the **BTZ** orientation can be considered to be inherently less impactful on these species compared to **2B1C**.

**FIGURE 5:**
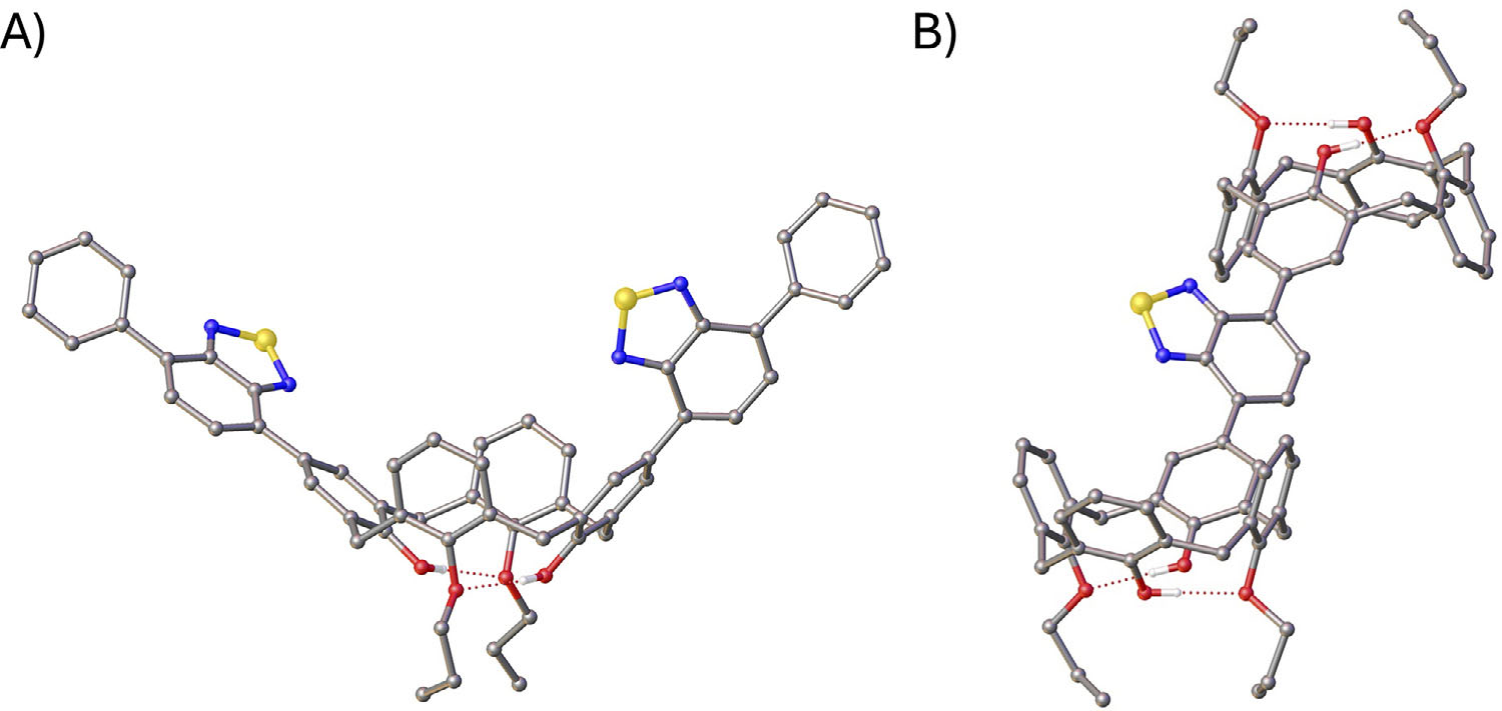
Single‐crystal x‐ray structures of **2B1C** (A) and **1B2C** (B) in ball‐and‐stick representation showing the **BTZ** PS linkers tethered to aryl rings on cavity‐containing C[4]s in the cone conformation. Color code: C—gray, N—blue, O—red, S—yellow. H atoms except those involved in stabilizing H‐bonding interactions (shown as dashed red lines) at the lower‐rim are omitted for clarity.

These structural observations corroborate the findings for the binding studies outlined above. In fact, excellent correlation can be drawn from the affinity of cavity to quencher (*K*_sv_) and the cavity features observed in the crystal structures that vary from the optimal **1B2C** geometry. Thus, **1B2C** presents the highest *K*_sv_, and this can be linked to the features observed in its crystal structure: that is, possessing two cavities, both in cone conformation (unlike **1B2C‐Pr**) and with no hindrance to cavity accessibility (unlike **2B1C**). Furthermore, these findings visually underline the effect of lower‐rim modification on the presence/accessibility of the catalytic cavities. Compounds such as **2B1C** and **1B2C** are stabilized in both solution and the solid state by lower‐rim hydrogen bonding interactions between the hydroxyl groups and ethereal oxygen atoms, and inspection of Figure 5 clearly shows the optimal cone conformation (which will display limited fluctuation in solution at room temperature due to said interactions). Conversely, greater fluctuation is expected for tetra‐*n*‐propoxy C[4] derivatives such as **1B2C‐Pr** due to the complete lack of lower‐rim hydrogen bonding interactions. In this case the molecule is unable to maintain a stable cavity structure and is expected to readily fluctuate between the two extremes of the pinched‐cone conformation, both of which have been observed in the crystal structures obtained in this study (Figures 4, S17, and S18) [33].

### 2.5 Photosensitization Testing

All cavity‐containing and control **BTZ** PSs were tested for their ability to produce ^1^O_2_ through the oxidation of *α*‐terpinene to ascaridole (Scheme 3, with general procedure covered in section 8 of the Supporting Information) [10, 32, 46, 47].

**SCHEME 3:**
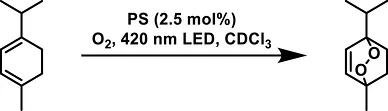
Conversion of *α*‐terpinene into ascaridole via ^1^O_2_ using a PS.

Each PS was employed in a 2.5 mol% loading with respect to the starting material *α*‐terpinene (27.2 mg, 0.2 mmol) and the solvent (3 mL) used in each test is reported in Table S2 (see Supporting Information). Control tests were performed with literature **BTZ** PSs to quantify the conversion for comparison with **BTZ‐C[4]s**. Compounds **OH‐BTZ**, **mOH‐BTZ**, **OHP‐BTZ,** and **Pr‐BTZ** yielded 12%, 16%, 15%, and 24% conversion, respectively (Table S2, entries 1–4). Further control tests were then performed to verify the reaction progress in the absence of PS (Table S2, entry 5, no conversion), and with benchmark PS **1B2C** without oxygen (Table S2, entry 6, trace product), or light (Table S2, entry 7, trace amounts). Additional investigation of different solvents from the standard CDCl_3_ was carried out in acetone, acetonitrile, chloroform, and tetrahydrofuran (83%–94%, Table S2, entries 8–11), with little variation on the outcome observed, especially from non‐deuterated chloroform. Therefore, further tests were carried out using deuterated chloroform to facilitate NMR sampling for the conversion analysis. The first test was carried out with **1B2C** in CDCl_3_ at 2.5 mol% for 1 h, sampling at 10 min intervals (Figure 6). The reaction was found to proceed linearly with time, with ∼18% of the starting material being converted every 10 min until completion. Repeating this in triplicate (Table S2, entries 12–14) afforded 93%–99% conversions with a standard deviation of 2.6% and error of 2.7%, respectively. Since the reaction was observed to follow zero‐order kinetics at high concentrations of *α*‐terpinene, further kinetic investigation was undertaken by performing a series of test runs with **1B2C** at varying concentrations of substrate (H:G ratio 0.5, 1, 2, 5, 10; see Supporting Information, Figures S20 and S21). The conversions (thus concentrations of ascaridole over the first 5 min, *V*_0_) were plotted against the concentration of starting material [*α*‐terpinene] in a Lineweaver–Burk plot to obtain the Michaelis–Menten constant (*K*_m_) for this system [48, 49, 50], thus gaining insight into the affinity of guests to **BTZ‐C[4]** hosts. Literature values for enzyme kinetics show a wide range of *K*_m_ values, between 1 mM for weaker affinity and >10^−6^ mM for strongly binding enzymes [48, 49, 50], and the obtained *K*_m_ for the herein presented system using **1B2C** was 6.21 × 10^−4^ mM. This indicates overall good affinity in the H:G complex, and a consequentially low threshold [*α*‐terpinene] for half‐saturation kinetics (and thus to achieve half‐maximum velocity, *V*_max_), which is in line with previous results highlighted above [48, 49, 50].

**FIGURE 6:**
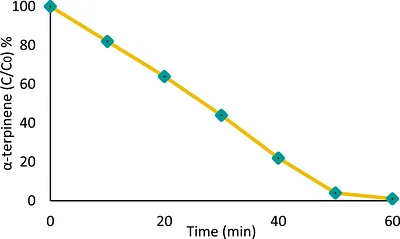
*α*‐terpinene consumption with **1B2C** (2.5 mol%) over 1 h.

The other members of the library were tested using analogous conditions (entries 15–20, Table S2). Notably, all PSs related to **OH‐BTZ** control (Figure 3) showed similar performance, producing above 90% ascaridole in 1 h with eightfold enhancement (Table 2). Other **BTZ‐C[4]** PSs also saw a sharp increase in productivity from both **2B1C** (which produced 33% ascaridole, 2.1‐fold compared to **mOH‐BTZ**) and **1B2C‐Pr** (1.8‐fold enhancement from **Pr‐BTZ**, Table 2). Lastly, **1PB2C** saw the smallest increment in production of ascaridole, with 17% in 1 h compared to its molecular **BTZ** PS equivalent **OHP‐BTZ** (15%, 1.1‐fold, Table 2).

**TABLE 2:** Summary of results of photosensitization tests for ascaridole production for all PSs under optimal conditions: α‐terpinene (27.2 mg, 0.2 mmol), **BTZ** PS (2.5 mol%), 1 h, CDCl_3_ (3 mL), 12 W 410–420 nm LED block.

PS	Conversion to ascaridole (%)^a^	Cavity conformation	Cavity sites to PS ratio	Relative PS structure	Conversion increment from non‐cavity PS
OH‐BTZ	12	—	—	OH‐BTZ	—
**1B2C**	**98**	Cone	2:1	OH‐BTZ	**8.2**
**2B2C** ^b^	**90** ^b^	Cone	1:1^b^	OH‐BTZ	**7.5**
**1B2C‐Me**	**94**	Cone	2:1	OH‐BTZ	**7.8**
**1B2C‐OH**	**92**	Cone	2:1	OH‐BTZ	**7.7**
mOH‐BTZ	16	—	—	mOH‐BTZ	—
**2B1C** ^b^	**33** ^b^	Cone	1:2^b^	mOH‐BTZ	**2.1**
Pr‐BTZ	24	—	—	Pr‐BTZ	—
**1B2C‐Pr**	**44**	Pinched cone	2:1	Pr‐BTZ	**1.8**
OHP‐BTZ	15	—	—	OHP‐BTZ	—
**1PB2C**	**17**	Cone (Ph spacer)	2:1	OHP‐BTZ	**1.1**

^a^ Conversion measured via ^1^H NMR by integration of handle peaks (Please see Section S8).

^b^ Loading halved to 1.25 mol% due to the presence of two **BTZ** catalytic active sites per unit.

The performances of each member of the PS library can therefore be grouped by the corresponding molecular photocatalyst structure and by the conformation and/or accessibility of the cavities adjacent to them. It emerges that all the most efficient photocatalysts belong to the same category (**1B2C**, **2B2C**, **1B2C‐Me**, and **1B2C‐OH**), that being **OH‐BTZ** photocatalyst structure (the least effective without C[4]), and with two cavities in cone conformation (which has pseudo‐C_4v_ symmetry). The second‐best category in ascaridole production increment order is observed for **2B1C**, consisting of a single cone‐shaped C[4] unit equipped with two **BTZ** units. While the 2.1‐fold increment is notable, it is clear (emerging from the *K*_sv_ data) that its cavity remains less accessible to guest substrate, possibly due to self‐inclusion of the PS toward the cavity and the overcrowding of its singular C[4] cavity. A close match in production increment of 1.8‐fold is reported for **1B2C‐Pr**, which may be related to the fluctuating pinched‐cone conformation in solution which also likely renders the cavity less accessible to a potential *α*‐terpinene guest. Lastly, **1PB2C** resulted in the least advantageous C[4]‐**BTZ** structure (despite possessing optimal cone conformations), as its minimal increment in conversion can be attributed to the added phenyl spacer that positions the two cavities farther away from the reactive **BTZ** core. This is corroborated by the abovementioned *K*_sv_ of 1.44 × 10^4^, a significant drop compared to the other library members. On the other hand, the PS in the first group (**1B2C**, **2B2C**, **1B2C‐Me**, and **1B2C‐OH**) performed this reaction with outstanding results, making them viable for scale up thanks to their facile synthesis, and potential utility in the aforementioned applications for ^1^O_2_. Further testing was carried with **1B2C** in the presence of a ^1^O_2_ scavenger 1,4‐diazabicyclo[2.2.2]octane (Table S2, entry 21, DABCO, 2 eq.), resulting in no significant production of ascaridole and further confirming the role the PS plays in this reaction.

Considering these results, a comparison can be drawn between the previously reported BODIPY‐C[4] species and the present **BTZ‐C[4]** ones, considering the differences in reaction conditions and molecular photocatalyst efficiency. The two main differences in the reaction conditions were the loading of PS (which varied in the previous study from 4.8% to 10% for BODIPY‐C[4]s versus the 2.5% of the **BTZ‐C[4]** PSs) and the saturation of oxygen in solution prior to the testing. Both these practices favor the results of the former PS library. Therefore, a test with **1B2C** was carried in such conditions (Table S2, entry 22, O_2_ bubbled through solvent prior to SM addition, 5 mol% loading of PS) which yielded full conversion in just 30 min, vastly outperforming all previously studied BODIPY‐C[4] PSs.

Finally, recycling and recoverability of the **BTZ‐C[4]s** was also assessed on **1B2C** (Table S2, entry 23). This was tested by recovering the PS from a previous run, which could be easily achieved by removing solvent under vacuum and washing the residue with methanol (in which **1B2C** is insoluble). After recovery, the recycling run obtained 88% conversion in 1 h, effectively showing minimal loss in productivity. These results also provide insights into photostability, since the spent PS was obtained from a reaction vial used 5 months prior to the recycling, noting that it was kept under ambient solar irradiation for that entire period. Therefore, the photostability in both solution and solid state was considered robust for **1B2C**, and its recovery was demonstrated to be particularly facile due to the insolubility of the PS in methanol. Thus, recovery and recycling of **1B2C** were deemed not only viable, but more beneficial than other literature homogeneous organic PSs, which usually lack in recycling capabilities as a consequence of both poor photostability and challenging recovery (usually requiring chromatographic separation) [16].

## 3 Conclusions

A library of seven novel PSs based on **BTZ** tethered to C[4] units was developed, building substantially on principles established recently for BODIPY‐C[4] species [32]. The synthesis of symmetric **BTZ** species resulted in good yields and required more straightforward purification compared to their non‐symmetric counterparts, while the optoelectronic properties remained similar to the analogue **BTZ**s without a cavity, with mild bathochromic shifts in absorption and emission, and lower PLQY. Testing of these PS was performed and measured with a literature benchmark of the photo‐oxidation of *α*‐terpinene into ascaridole (Scheme 3) [10, 32, 46, 47]. The results produced were enlightening on the contribution of structural modification onto reactivity toward ascaridole. It was observed that **BTZ‐C[4]** members which presented two cavities vicinal to the active **BTZ** acceptor block and in the cone conformation were the best performing PSs, with over eightfold enhancement in product conversion compared to their non‐cavity containing literature analogues, and reaching completion in only 1 h at 2.5 mol% loading in batch. Distancing the influential cavities from the reactive **BTZ** core by insertion of phenyl spacers led to significant reduction in efficiency, providing valuable insight into the behavior of these supramolecular photocatalysts. Work continues with a view to further exploring the balance between the positioning of cavities relative to PS linkers, improving photo‐oxidation performance through enhanced concentration effects, demonstrating selectivity through system design, and incorporating these systems into materials for downstream application. Results of these studies will be reported in due course.

## Conflicts of Interest

The authors declare no conflicts of interest.

## Supporting information



The supporting information document contains all synthetic procedures, characterization of new molecules, UV‐Visible data, GPC data, single crystal x‐ray diffraction details and data, Stern‐Volmer analysis of the supramolecular and molecular PSs, and complete outline of the photosensitization experiments. The authors have cited additional references within the Supporting Information [51, 52, 53, 54, 55, 56, 57].
**Supporting File 1**: chem71443‐sup‐0001‐SuppMat.docx.

## Data Availability

Supporting information supporting this study are available in the supplementary material of this article. Deposition numbers CCDC 2555361 (for 2B1C), 2555362 (for 1B2C) and 2555363 (for 1B2C‐Pr), contain the supplementary crystallographic data for this paper. These data are provided free of charge by the joint Cambridge Crystallographic Data Centre and Fachinformationszentrum Karlsruhe Access Structures service.

## References

[1] I. Pibiri , S. Buscemi , A. Palumbo Piccionello , and A. Pace , “Photochemically Produced Singlet Oxygen: Applications and Perspectives,” ChemPhotoChem 2 (2018): 535–547, 10.1002/cptc.201800076.

[2] R. Schmidt , “Photosensitized Generation of Singlet Oxygen,” Photochemistry and Photobiology 82 (2006): 1161, 10.1562/2006-03-03-IR-833. 16683906

[3] C. G. Thomson , A. L. Lee , and F. Vilela , “Heterogeneous Photocatalysis in Flow Chemical Reactors,” Beilstein Journal of Organic Chemistry 16 (2020): 1495–1549, 10.3762/bjoc.16.125. PMC7323633 32647551

[4] A. Zhakeyev , J. Tobin , H. Wang , F. Vilela , and J. Xuan , “Additive Manufacturing of Photoactive Polymers for Visible Light Harvesting,” Energy Procedia 158 (2019): 5608–5614, 10.1016/j.egypro.2019.01.579.

[5] A. Kamkaew , S. H. Lim , H. B. Lee , L. V. Kiew , L. Y. Chung , and K. Burgess , “BODIPY Dyes in Photodynamic Therapy,” Chemical Society Reviews 42 (2013): 77–88, 10.1039/C2CS35216H. PMC3514588 23014776

[6] Q. Wang , B. Xia , J. Xu , et al., “Biocompatible Small Organic Molecule Phototheranostics for NIR‐II Fluorescence/Photoacoustic Imaging and Simultaneous Photodynamic/Photothermal Combination Therapy,” Materials Chemistry Frontiers 3 (2019): 650–655, 10.1039/C9QM00036D.

[7] B. C. Ma , S. Ghasimi , K. Landfester , and K. A. I. Zhang , “Enhanced Visible Light Promoted Antibacterial Efficiency of Conjugated Microporous Polymer Nanoparticles via Molecular Doping,” Journal of Materials Chemistry B 4 (2016): 5112–5118, 10.1039/C6TB00943C. 32263508

[8] S. P. Pitre , C. D. McTiernan , H. Ismaili , and J. C. Scaiano , “Metal‐Free Photocatalytic Radical Trifluoromethylation Utilizing Methylene Blue and Visible Light Irradiation,” ACS Catalysis 4 (2014): 2530–2535, 10.1021/cs5005823.

[9] M. Singsardar , A. Dey , R. Sarkar , and A. Hajra , “Visible‐Light‐Induced Organophotoredox‐Catalyzed Phosphonylation of 2 H ‐Indazoles With Diphenylphosphine Oxide,” Journal of Organic Chemistry 83 (2018): 12694–12701, 10.1021/acs.joc.8b02019. 30246531

[10] C. G. Thomson , C. M. S. Jones , G. Rosair , et al., “Continuous‐Flow Synthesis and Application of Polymer‐Supported BODIPY Photosensitisers for the Generation of Singlet Oxygen; Process Optimised by in‐line NMR Spectroscopy,” Journal of Flow Chemistry 10 (2020): 327–345, 10.1007/s41981-019-00067-4.

[11] E. Bassan , A. Gualandi , P. G. Cozzi , and P. Ceroni , “Design of BODIPY Dyes as Triplet Photosensitizers: Electronic Properties Tailored for Solar Energy Conversion, Photoredox Catalysis and Photodynamic Therapy,” Chemical Science 12 (2021): 6607–6628, 10.1039/D1SC00732G. PMC8132938 34040736

[12] S. Noimark , E. Salvadori , R. Gómez‐Bombarelli , A. J. MacRobert , I. P. Parkin , and C. W. M. Kay , “Comparative Study of Singlet Oxygen Production by Photosensitizer Dyes Encapsulated in Silicone: Towards Rational Design of Anti‐Microbial Surfaces,” Physical Chemistry Chemical Physics 18 (2016): 28101–28109. 10.1039/c6cp02529c 27722271

[13] J. M. Tobin , T. J. D. McCabe , A. W. Prentice , et al., “Polymer‐Supported Photosensitizers for Oxidative Organic Transformations in Flow and Under Visible Light Irradiation,” ACS Catalysis 7 (2017): 4602–4612, 10.1021/acscatal.7b00888.

[14] A. Zhakeyev , M. C. Jones , C. G. Thomson , et al., “Additive Manufacturing of Intricate and Inherently Photocatalytic Flow Reactor Components,” Additive Manufacturing 38 (2021): 101828.

[15] H. Urakami , K. Zhang , and F. Vilela , “Modification of Conjugated Microporous Poly‐benzothiadiazole for Photosensitized Singlet Oxygen Generation in Water,” Chemical Communications 49 (2013): 2353, 10.1039/c3cc38956a. 23407715

[16] D. Taylor , L. Amicosante , S. J. Dalgarno , and F. Vilela , “Shining a Light on benzo[c][1,2,5]thiadiazole Photocatalysts,” Dyes and Pigments 244 (2026): 113100, 10.1016/j.dyepig.2025.113100.

[17] T. C. Lovell , Z. R. Garrison , and R. Jasti , “Synthesis, Characterization, and Computational Investigation of Bright Orange‐Emitting Benzothiadiazole [10]Cycloparaphenylene,” Angewandte Chemie International Edition 59 (2020): 14363–14367, 10.1002/anie.202006350. 32515041

[18] D. Taylor , S. J. Dalgarno , Z. Xu , and F. Vilela , “Conjugated Porous Polymers: Incredibly Versatile Materials With Far‐Reaching Applications,” Chemical Society Reviews 49 (2020): 3981–4042, 10.1039/C9CS00315K. 32400783

[19] W. Q. Zhang , Q. Y. Li , J. Y. Cheng , et al., “Ratiometric Luminescent Detection of Organic Amines due to the Induced Lactam–Lactim Tautomerization of Organic Linker in a Metal–Organic Framework,” ACS Applied Materials & Interfaces 9 (2017): 31352–31356, 10.1021/acsami.7b11125. 28853545

[20] S. Bhattacharyya , S. R. Ali , M. Venkateswarulu , et al., “Self‐Assembled Pd_12_ Coordination Cage as Photoregulated Oxidase‐Like Nanozyme,” Journal of the American Chemical Society 142 (2020): 18981–18989, 10.1021/jacs.0c09567. 33104330

[21] S. Li , L. Li , Y. Li , et al., “Fully Conjugated Donor–Acceptor Covalent Organic Frameworks for Photocatalytic Oxidative Amine Coupling and Thioamide Cyclization,” ACS Catalysis 10 (2020): 8717–8726, 10.1021/acscatal.0c01242.

[22] X. Li , S. Yang , F. Zhang , L. Zheng , and X. Lang , “Facile Synthesis of 2D Covalent Organic Frameworks for Cooperative Photocatalysis With TEMPO: The Selective Aerobic Oxidation of Benzyl Amines,” Applied Catalysis B: Environmental 303 (2022): 120846, 10.1016/j.apcatb.2021.120846.

[23] L. Buglioni , F. Raymenants , A. Slattery , S. D. A. Zondag , and T. Noël , “Technological Innovations in Photochemistry for Organic Synthesis: Flow Chemistry, High‐Throughput Experimentation, Scale‐up, and Photoelectrochemistry,” Chemical Reviews 122 (2022): 2752–2906, 10.1021/acs.chemrev.1c00332. PMC8796205 34375082

[24] L. J. Riwar , N. Trapp , K. Root , R. Zenobi , and F. Diederich , “Supramolecular Capsules: Strong Versus Weak Chalcogen Bonding,” Angewandte Chemie International Edition 57 (2018): 17259–17264, 10.1002/anie.201812095. 30480857

[25] S. Li , K. Liu , X.‐C. Feng , et al., “Synthesis and Macrocyclization‐Induced Emission Enhancement of Benzothiadiazole‐Based Macrocycle,” Nature Communications 13 (2022): 2850, 10.1038/s41467-022-30121-0. PMC9126912 35606365

[26] Z. L. Qiu , C. Tang , X. R. Wang , et al., “Tetra‐Benzothiadiazole‐Based [12]Cycloparaphenylene With Bright Emission and Its Supramolecular Assembly,” Angewandte Chemie International Edition 59 (2020): 20868–20872, 10.1002/anie.202008505. 32749018

[27] S. Ito , K. Ono , K. Johmoto , H. Uekusa , and N. Iwasawa , “Switching of the Solid‐State Guest Selectivity: Solvent‐Dependent Selective Guest Inclusion in a Crystalline Macrocyclic Boronic Ester,” Chemical Science 7 (2016): 5765–5769, 10.1039/C5SC04766H. PMC6054024 30090290

[28] T. Uchikura , K. Ono , K. Takahashi , and N. Iwasawa , “Utilization of Donor–Acceptor Interactions for the Catalytic Acceleration of Nucleophilic Additions to Aromatic Carbonyl Compounds,” Angewandte Chemie International Edition 57 (2018): 2130–2133, 10.1002/anie.201711726. 29314440

[29] C. W. Chu and M. Horie , “Synthesis and Characterization of Cyclic Conjugated Architectures Composed of Thiophene and Benzothiadiazole Units,” Asian Journal of Organic Chemistry 2 (2013): 838–842, 10.1002/ajoc.201300158.

[30] S. S. Navid , R. Hosseinzadeh , R. Oestreich , S. Abdpour , T. H. Y. Beglau , and C. Janiak , “Calix[4]arene@MIL‐101 as Host@MOF for Cage‐in‐Cage Pore Space Partitioning for Enhanced CO_2_ Separation and Catalysis,” Journal of Materials Chemistry A 13 (2025): 3894–3902, 10.1039/D4TA07357F.

[31] M. Farber , V. Rawat , Y. Diskin‐posner , R. Dobrovetsky , and A. Vigalok , “Polyaromatic Calixarene Hosts: Calix[4]pyrenes,” Organic Letters 26 (2024): 5731–5735, 10.1021/acs.orglett.4c01850. PMC11249777 38935556

[32] M. U. Main , D. Taylor , L. Amicosante , et al., “Host Cavities Enhance the Photocatalytic Conversion of α ‐Terpinene to Ascaridole Under Visible‐Light Irradiation,” Chemistry—A European Journal 31 (2025): e02113, 10.1002/chem.202502113. PMC12498068 40891547

[33] J. Scheerder , R. H. Vreekamp , J. F. J. Engbersen , W. Verboom , J. P. M. Van Duynhoven , and D. N. Reinhoudt , “The Pinched Cone Conformation of Calix[4]Arenes: Noncovalent Rigidification of the Calix[4]Arene Skeleton,” Journal of Organic Chemistry 61 (1996): 3476–3481, 10.1021/jo9600262.

[34] D. Taylor , T. Malcomson , A. Zhakeyev , et al., “4,7‐Diarylbenzo[ c ][1,2,5]Thiadiazoles as Fluorophores and Visible Light Organophotocatalysts,” Organic Chemistry Frontiers 9 (2022): 5473–5484, 10.1039/D2QO01316A.

[35] A. Arduini , E. Brindani , G. Giorgi , A. Pochini , and A. Secchi , “Recognition of Guests Bearing Donor and Acceptor Hydrogen Bonding Groups by Heteroditopic Calix [ 4 ] Arene Receptors,” Tetrahedron 59 (2003): 7587–7594, 10.1016/S0040-4020(03)01126-8.

[36] C. D. Gutsche and L.‐G. Lin , “Calixarenes 12,” Tetrahedron 42 (1986): 1633–1640, 10.1016/S0040-4020(01)87580-3.

[37] M. Lee , K. Yang , C. Tsoo , C. Shu , and L. Lin , “Calix[4]quinone. Part 1: Synthesis of 5‐hydroxycalix[4]arene by calix[4]quinone Monoketal Route,” Tetrahedron 57 (2001): 8095–8099, 10.1016/S0040-4020(01)00787-6.

[38] M. Ve , J. Gagnon , K. Villeneuve , M. Drouin , and P. D. Harvey , “( η 5 ‐Pentamethylcyclopentadienyl ) Rhodium Complexes of Upper‐Rim Monophosphinated Calix [4] Arene,” Organometallics 20 (2001): 273–281.

[39] G. Hennrich , M. T. Murillo , P. Prados , et al., “Alkynyl Expanded Donor–Acceptor Calixarenes: Geometry and Second‐Order Nonlinear Optical Properties,” Chemistry—A European Journal 13 (2007): 7753–7761, 10.1002/chem.200700615. 17610220

[40] F. de la Vega and Y. Sasson , “Selective Para‐Bromination of Toluene Catalysed by Na‐Y Zeolite in the Presence of an Epoxide,” Journal of the Chemical Society, Chemical Communications 25 (1989): 653–654.

[41] C. M. S. Jones , A. Gakamsky , and J. Marques‐Hueso , “The Upconversion Quantum Yield (UCQY): A Review to Standardize the Measurement Methodology, Improve Comparability, and Define Efficiency Standards,” Science and Technology of Advanced Materials 22 (2021): 810–848, 10.1080/14686996.2021.1967698. PMC8725918 34992499

[42] R. Kumar , A. Sharma , H. Singh , et al., “Revisiting Fluorescent Calixarenes: From Molecular Sensors to Smart Materials,” Chemical Reviews 119 (2019): 9657–9721, 10.1021/acs.chemrev.8b00605. 31306015

[43] S. K. Dinda , M. Althaf Hussain , A. Upadhyay , and C. P. Rao , “Supramolecular Sensing of 2,4,6‐Trinitrophenol by a Tetrapyrenyl Conjugate of Calix[4]Arene: Applicability in Solution, in Solid State, and on the Strips of Cellulose and Silica Gel and the Image Processing by a Cellular Phone,” ACS Omega 4 (2019): 17060–17071, 10.1021/acsomega.9b02855. PMC6796239 31646253

[44] K. Boonkitpatarakul , Y. Yodta , N. Niamnont , and M. Sukwattanasinitt , “Fluorescent Phenylethynylene Calix[4]Arenes for Sensing TNT in Aqueous Media and Vapor Phase,” RSC Advances 5 (2015): 33306–33311, 10.1039/C5RA02758F.

[45] F. B. Ildikó Mohammed‐Ziegler , “Optical Spectroscopy and Theoretical Studies in Calixarene Chemistry,” Journal of Inclusion Phenomena and Macrocyclic Chemistry 58 (2007): 19–42, 10.1007/s10847-006-9132-z.

[46] D. S. Lee , Z. Amara , C. A. Clark , et al., “Continuous Photo‐Oxidation in a Vortex Reactor: Efficient Operations Using Air Drawn From the Laboratory,” Organic Process Research & Development 21 (2017): 1042–1050, 10.1021/acs.oprd.7b00153. PMC5526652 28781513

[47] K. Behm , E. Fazekas , M. J. Paterson , F. Vilela , and R. D. McIntosh , “Discrete Ti−O−Ti Complexes: Visible‐Light‐Activated, Homogeneous Alternative to TiO_2_ Photosensitisers,” Chemistry—A European Journal 26 (2020): 9486–9494, 10.1002/chem.202001678. PMC7496837 32428304

[48] P. K. Robinson , “Enzymes: Principles and Biotechnological Applications,” Essays in Biochemistry 59 (2015): 1–41, 10.1042/bse0590001. PMC4692135 26504249

[49] A. Cornish‐Bowden , Fundamentals of Enzyme Kinetics (Wiley, 2012).

[50] Symbolism and Terminology in Enzyme Kinetics Recommendations 1981, European Journal of Biochemistry 128 (1982): 281–291, 10.1111/j.1432-1033.1982.tb06963.x. 7151780

[51] C. D. Gutsche and P. F. Pagoria , “Calixarenes. 16. Functionalized Calixarenes: The Direct Substitution Route,” Journal of Organic Chemistry 50 (1985): 5795–5802, 10.1021/jo00350a071.

[52] M. A. Tairov , M. O. Vysotsky , O. I. Kalchenko , V. V. Pirozhenko , and V. I. Kalchenko , “Symmetrical and Inherently Chiral Water‐Soluble Calix [4] Arenes Bearing Dihydroxyphosphoryl Groups,” Journal of the Chemical Society, Perkin Transactions 1 31 (2002): 1405–1411.

[53] E. H. G. da Cruz , P. H. P. R. Carvalho , J. R. Corrêa , et al., “Design, Synthesis and Application of Fluorescent 2,1,3‐benzothiadiazole‐triazole‐Linked Biologically Active Lapachone Derivatives,” New Journal of Chemistry 38 (2014): 2569.

[54] J. P. Heiskanen , P. Vivo , N. M. Saari , et al., “Synthesis of Benzothiadiazole Derivatives by Applying C–C Cross‐Couplings,” Journal of Organic Chemistry 81 (2016): 1535–1546, 10.1021/acs.joc.5b02689. 26771655

[55] X. Zhang , H. Gorohmaru , M. Kadowaki , et al., “Benzo‐2,1,3‐thiadiazole‐Based, Highly Dichroic Fluorescent Dyes for Fluorescent Host–Guest Liquid Crystal Displays,” Journal of Materials Chemistry (2004): 1901–1904, 10.1039/B402645D.

[56] J. Shen , R. Steinbach , J. M. Tobin , et al., “Photoactive and Metal‐Free Polyamide‐Based Polymers for Water and Wastewater Treatment Under Visible Light Irradiation,” Applied Catalysis B: Environmental 193 (2016): 226–233, 10.1016/j.apcatb.2016.04.015.

[57] P. H. Jun Zhang , A. Konsmo , A. Sandberg , et al., “Phenolic Bis‐styrylbenzo[c]‐1,2,5‐thiadiazoles as Probes for Fluorescence Microscopy Mapping of Aβ Plaque Heterogeneity,” Journal of Medicinal Chemistry 62 (2019): 2038–2048. 10.1021/acs.jmedchem.8b01681 30707834

