# Supplementary material for: Benzo[c][1,2,5]Thiadiazole Linkers Enhance the Effectiveness of Cavity‐Containing Supramolecular Photocatalysts
Source: Chemistry. 2026 Aug 3;32(41):e71443. doi: 10.1002/chem.71443 (PMC13729942; doi:10.1002/chem.71443)
Supplement: Supplementary file 1 [file CHEM-32-e71443-s001.docx]

Benzo[*c*][1,2,5]thiadiazole Linkers Enhance the Effectiveness of Cavity-Containing Supramolecular Photocatalysts

**Authors:** Leonardo Amicosante,^a^ Dominic Taylor,^a,b^ Cameron Campbell,^a^ Daniel M. Lambden,^a^ Georgina M. Rosair,^a^ Filipe Vilela,*^a^ Scott J. Dalgarno.*^a^

*Corresponding authors

^a^School of Engineering and Physical Sciences, Heriot-Watt University, Edinburgh, UK

E-mail: s.j.dalgarno@hw.ac.uk, f.vilela@hw.ac.uk

^b^Current address: EaStCHEM School of Chemistry, University of Edinburgh, David Brewster Road, Edinburgh, UK

All commercially available compounds were used as received and purchased from Sigma-Aldrich, Fluorochem, Apollo Scientific or Fischer Scientific. Where not stated otherwise, the reactions were performed at room temperature under ambient conditions.

NMR spectra of synthetic products were recorded using a Bruker AVIII 400 MHz and a Bruker AVIII 300 MHz spectrometers, using the residual solvent peak as an internal reference. All the NMR experiments were conducted in CDCl_3_ at 298 K and were referenced to the residual solvent signal.

UV-Visible absorption spectra for the synthesised photosensitisers were obtained using a Perkin-Elmer Lambda 35 spectrometer in chloroform solution in quartz cuvettes with a path length of 1 cm. Emission spectra in the visible region were recorded using a Perkin Elmer LS 55 fluorescence spectrometer in chloroform solution using quartz cuvettes with a path length of 1 cm.

The photoluminescence quantum yields (PLQY) were measured following the conventions and considerations from Jones *et al*.^1^ A FLS920 spectrofluorometer (Edinburgh Instruments Ltd) equipped with an extended red-sensitive photon multiplier detector (Hamamatsu, R2658P) and an integrating sphere with a 102 mm inner diameter (Yobin Yvon) were used for all PLQY measurements. For the excitation, a 450 W Xenon lamp (Edinburgh Instruments Ltd, Xe2) was employed. The samples consisted of diluted dyes in CHCl_3_ at 20 µM. The sample was contained in a square quartz cuvette with 10 mm light path and placed in the centre of the integrating sphere. The reference sample, or blank, was pure CHCl_3_. The excitation wavelengths were set to the peak absorption of each sample with a bandwidth set to 10 nm. The excitation and emission regions were typically measured with a 1 nm step size. The associated measurement error is 0.2%.

All HRMS (ESI-MS) spectra of the synthesised photocatalysts were measured using time-of flight mass spectrometry using a Waters Acquity UPLC-Xevo G2 QTof with the electrospray ionisation source in positive mode.

All IR spectra were recorded on solid powder/crystals using a NicoletTMiSTM5 FTIR spectrometer.

Gel Permeation Chromatography (GPC) analysis was performed on a Shimadzu High Performance Liquid Chromatograph equipped with 7.5 mm internal diameter Agilent GPC Column. The detector used was a Shimadzu SPD-20A UV-Vis detector set to 254 nm and a Shimadzu RID-20A. HPLC grade tetrahydrofuran (THF, 99.8%, Acros Organics) was utilized as the eluent with flow rate of 1 mL/min, with oven temperature of 30 ^o^C. The system was calibrated against 10 polystyrene standards in the range of 162–364,000 g mol⁻¹. Injection volume was 20 µL.

X-Ray crystallographic data was collected using a D8 Venture diffractometer mounted with Cu source. The temperature of collection was 100(2) K. All crystals were grown using slow evaporation (from toluene or chloroform) or vapour diffusion (chloroform/ methanol). Anisotropic displacement parameters (ADPs) of atoms are displayed at a 50% probability level

1. Experimental procedures:

Calix[4]arene was produced according to literature procedure from Gutsche *et al.*^2^

Scheme S1. synthetic path towards 5-Bromo-26,28-dipropoxy-calix[4]arene

**25,27-Dipropoxycalix[4]arene**

The reaction was carried following the literature procedure reported in Main *et al.*^3^

**25,27-Dipropoxy-26-benzoyloxycalix[4]arene**

25,27-Dipropoxycalix[4]arene (1.50 g, 2.95 mmol) was added to a 100 mL round bottom flask and dissolved in dry THF (50 mL). The solution was then cooled to -78^o^C and n-BuLi (1.3 mL, 1.1 eq, 2.5 M in hexane) was added dropwise. The reaction was allowed to stir for 30 min at -78 ^o^C and then benzoyl chloride (0.38 mL, 1.1 eq) was added dropwise. After warming up to room temperature the reaction was then stirred for addition 90 mins, methanol was added to quench unreacted benzoyl chloride. The solvent was then removed under reduced pressure and the colourless residue was taken up in DCM and washed with 1M HCl (100 mL), deionised water (3x 100 mL), brine (2 x 100 mL) then dried over MgSO_4_ and solvent removed under vacuum. The crude product was purified by recrystallisation from DCM/MeOH to give a white crystalline solid (1.58 g, 88%).	**^1^H NMR** (400 MHz, CDCl_3_) δ_H_ 8.84 (d, J=7.52 Hz, 4H), 8.18 (t, J = 23.81 Hz, 2H), 7.81 (d, J = 8.25 Hz, 4H), 7.65 (s, 2H), 7.60 (m, 2H), 7.53 (m, 4H), 7.27 (m, 7H), 7.04 (m, 6H), 6.73 (d, 4H), 6.65 (m, 6H), 6.52 (t, J=7.36 Hz, 4H), 4.28 (d, J=13.01, 4H), 4.14 (d, J=13.01, 4H), 3.44 (m, 8H), 3.29 (d, J=12.22, 8H), 1.17 (m, 8H), 0.60 (t, J=7.44, 12H).**^13^C NMR** (100.6 MHz, CDCl_3_) δ_C_ 167.42 (C), 153.44 (C),153.26 (C),146.9 (C),135.76 (C), 133.18 (CH), 132.98 (C),132.78 (C),132.58 (CH), 129.64 (C), 128.72 (CH), 128.61 (CH), 128.54 (CH), 128.34 (CH), 128.24 (C), 127.94 (CH), 126.03 (CH), 123.94 (CH), 118.70 (CH), 78.48 (CH_2_), 31.10 (CH_2_), 30.45 (CH_2_), 22.53 (CH_2_), 10.56 (CH_3_). **HRMS** (ESI) m/z: [M + K]+ Calcd for C₄₁H₄₀O₅ 651.25073; Found 651.25052.

Literature procedure adapted from Tairov *et al.*^4^

**5-Bromo-26,28-dipropoxy-27-benzoyloxycalix[4]arene**

25,27-Dipropoxy-26-benzoyloxycalix[4]arene (306 mg, 0.5 mmol) was dissolved in CHCl_3_ (3 mL). N-bromosuccinimide (148 mg, 0.83 mmol) and Azobisisobutyronitrile (AIBN) (2 mg) were added, and reaction stirred at room temperature for 24 hours. The reaction mixture was then washed with 10% sodium thiosulphate solution (100 mL), and twice with deionised water (2 x 100 mL). The organic phase was dried over MgSO_4_, and solvent removed under vacuum to yield a yellow solid (295 mg, 75%). **^1^H NMR** (400 MHz, CDCl_3_) δ_H_ 8.81 (d, J=7.52 Hz, 4H), 7.61 (m, 2H), 7.54 (t, J = 6.73 Hz, 4H), 7.37 (s, 2H), 7.27 (m, 4H), 7.15 (s, 4H), 7.05 (m, 2H), 6.75 (m, 4H), 6.63 (m, 4H), 6.55 (t, J=7.36 Hz, 4H), 4.24 (d, J=13.01, 4H), 4.11 (d, J=13.01, 4H), 3.42 (m, 8H), 3.26 (dd, J=12.54, 26.05, 8H), 1.16 (m, 8H), 0.60 (t, J=7.44, 12H).**^13^C NMR** (100.6 MHz, CDCl_3_) δ_C_ 167.35 (C), 153.25 (C), 152.69 (C), 146.85 (C), 135.69 (C), 133.27 (CH), 133.15 (C), 132.47 (CH), 131.91 (C), 130.91 (CH), 130.23 (C), 129.64 (C), 128.72 (CH), 128.70 (CH), 128.56 (CH), 127.96 (CH), 126.11 (CH), 124.08 (CH), 110.33 (C), 78.56 (CH_2_), 31.09 (CH_2_), 30.38 (CH_2_), 22.53 (CH_2_), 10.55 (CH_3_).

Literature procedure adapted from Arduini *et al.*^5^

**5-Bromo-26,28-dipropoxy-calix[4]arene**

5-Bromo-26,28-dipropoxy-27-benzoyloxycalix[4]arene (897 mg, 1.1 mmol) was dissolved in ethanol (13 mL) and THF (38 mL). NaOH (4.4 g) was dissolved in deionised water (25 mL) and the two solutions combined and subsequently heated to reflux overnight, changing colour to dark purple upon heating. The solvents were removed under reduced pressure and the resulting purple solid was then washed with 1M HCl solution (2x 100 mL), and once with brine (1x 100 mL) and deionised water (1x 100 mL). The organic phase was dried over MgSO_4_, and solvent removed under vacuum. The residue was then washed with methanol (100 mL) to yield a pale orange powder (558 mg, 96 %). **^1^H NMR** (400 MHz, CDCl_3_) δ_H_ 8.43 (s, 1H), 8.23 (s, 1H), 7.17 (s, 2H), 7.06 (d, J = 7.48 Hz, 2H), 6.93 (m, 4H), 6.77 (t, J = 7.60 Hz, 2H), 6.65 (t, J = 7.50 Hz, 1H), 4.29 (dd, J = 12.81 Hz, 10.69 Hz, 4H), 3.97 (dt, J = 7.96 Hz, 6.34 Hz, 4H), 3.35 (dd, J = 24.07 Hz, 12.97 Hz, 4H), 2.07 (sextet, J = 7.37 Hz, 4H), 1.31 (t, J = 7.44 Hz, 6H), **^13^C NMR** (100.6 MHz, CDCl_3_) δ_C_ 153.4 (C), 152.8 (C), 152.0 (C), 133.7 (C), 132.8 (C), 130.8 (CH), 130.3 (C), 129.4 (CH), 129.0 (CH), 128.6 (CH), 128.1 (C), 125.5 (CH), 119.2 (CH), 110.5 (C), 78.5 (CH_2_), 31.5 (CH_2_), 31.3 (CH_2_), 23.6 (CH_2_), 11.0 (CH_3_).

Literature procedure adapted from Gutsche *et al.*^6^

**5,17-Dibromo-26,28-dipropyloxycalix[4]arene**

25,27-Dipropoxycalix[4]arene (3.19 g, 6.28 mmol) was dissolved in dry DCM (140 mL) then a solution of bromine (1.1 mL, 3.43 g, 21.5 mmol) in DCM (40 mL) was added dropwise over a period of 30 minutes. During this time a white precipitate formed, which was filtered off and washed with cold methanol to yield the product as a white powder (3.7495 g, 80%). The filtrate was then washed with a 10% sodium thiosulfate solution to remove bromine before disposal (added slowly). **^1^H NMR** (CDCl_3_, 300 MHz) δ_H_ 8.36 (s, 2 H), 7.17 (s, 4H), 6.94 (d, J = 7.5 Hz, 4H), 6.80 (t, J = 7.6 Hz, 2H), 4.28 (d, J = 13.0 Hz, 4H), 3.95 (t, J = 6.2 Hz, 4H), 3.33 (d, J = 13.1 Hz, 4H), 2.06 (m, 4H), 1.29 (t, J = 7.7 Hz, 6H). **IR** 𝜈̅ (cm^-1^) 3120 (m, O-H str.), 2958 (m, C-H str.). A ^13^C NMR spectra of suitable quality could not be obtained due to the low solubility in common solvents.

**5-(4,4,5,5-tetramethyl-1,3,2-dioxaborolan-2-yl)-26,28-dipropoxy-calix[4]arene**

5-Bromo-26,28-dipropoxy-calix[4]arene (1.17 g, 2 mmol), Bis(pinacolato)diboron (0.765 g, 3 mmol), potassium acetate (400 g, 4 mmol), and Pd(dppf)Cl_2_ (5 mg) were charged in a 2- neck round bottomed flask, air was replaced with nitrogen gas, and dry dioxane (100 mL) was added. The Miyaura borylation reaction was carried at 95 ^o^C overnight under a stream of nitrogen. The crude was then dissolved in dichloromethane and washed twice with brine (100 mL) and twice with water (100 mL). The solution was dried over MgSO_4_ and the solvent removed under reduced pressure. The resulting brown crude was then washed with methanol to remove excess bis(pinacolato)diboron and the resulting powder filtered and purified via flash column chromatography (dichloromethane 100%), yielding an off-white solid (0.211 g, 16.5%). **^1^H NMR** (400 MHz, CDCl_3_) δ_H_ 8.62 (s, 1 H), 8.25 (s, 1 H), 7.54 (s, 2H), 7.05 (d, J=7.56 Hz, 2H), 6.99 (m, 2H), 6.91 (m, 2H), 6.74 (t, J=7.94 Hz, 2H), 6.63 (t, J=7.94 Hz, 1H), 4.32 (d, J=13.17 Hz, 2H), 4.29 (d, J=13.17 Hz, 2H), 3.97 (t, J=6.20 Hz, 4H), 3.45 (d, J=12.91 Hz, 2H), 3.38 (d, J=12.91 Hz, 2H), 2.05 (m, 4H), 1.31 (t, J=7.49 Hz, 6H). **^13^C NMR** (100.6 MHz, CDCl_3_) δ_C_ 156.7 (C), 153.3 (C), 151.8 (C), 135.5 (CH), 133.4 (C), 133.3 (C), 129.12 (CH), 128.9 (CH), 128.3 (CH), 128.2 (C), 127.6 (C), 125.3 (CH), 118.9 (CH), 83.3 (C), 78.3 (CH_2_), 31.4 (CH_2_), 31.2 (CH_2_), 24.9 (CH_3_), 23.5 (CH_2_), 10.9 (CH_3_). **HRMS** (ESI) m/z: [M + K]+ Calcd for C_40_H_47_BO_6_ 634.34657; Found 634.34778.

Scheme S2. synthetic path towards 5-Bromo-calix[4]arene-25,26,27,28-tetrol

**25,26,27-Tribenzoyloxycalix[4]arene**

Calix[4]arene (4.24 g, 10 mmol) was dissolved in pyridine (50 mL) and cooled in an ice bath. Benzoyl chloride (10 mL, 86 mmol) was added and the reaction mixture stirred for 3 hours. The reaction mixture was then slowly poured over water (300 mL), crashing out a white solid. The product was then filtered and recrystallised from a 2-layer system of methanol/chloroform (50 mL/ 10 mL) overnight, to yield off white crystals. (6.065 g, 83%) **^1^H NMR** (CDCl_3_, 300 MHz):  δ_H_ 8.12 (d, J = 7.35 Hz, 4H), 7.75 (t, J = 7.44 Hz, 2H), 7.59 (t, J = 7.45 Hz, 1H), 7.55 (t, J = 7.45 Hz, 4H), 7.30−7.20 (m, 4H), 7.08 (d, J = 7.45Hz, 2H), 7.03 (dd, J = 6.67, 2.66, 2H), 6.92 (d, J = 7.50 Hz, 2H), 6.79 (t, J = 7.36 Hz, 1H), 6.76 (t, J = 7.44 Hz, 1H), 6.65−6.55 (m, 4H), 5.49 (s, 1H, OH), 3.95 (d, J = 14.34 Hz, 2H), 3.85 (d, J = 15.55 Hz, 2H), 3.75 (d, J = 15.37 Hz, 2H), 3.53 (d, J = 14.23 Hz, 2H).

Literature procedure adapted from Lee *et al.*^7^

Full characterisation in literature procedures from Vézina *et al.*^8^

**5-Bromo-26,27,28-tribenzoyloxycalix[4]arene**

25,26,27-Tribenzoyloxycalix[4]arene (4.41 g, 6 mmol) was charged in a 2 neck-round bottomed flask connected to a condenser and a nitrogen line and dissolved in chloroform (200 mL). Bromine (0.31 mL, 6 mmol) was injected into the second arm, which was then connected to a trap containing 10% sodium thiosulfate solution. The reaction was carried for 30 minutes at room temperature and a positive nitrogen pressure, inducing any excess bromine fume into the trap. Following that, the reaction was quenched by addition of 10% sodium thiosulfate solution (2x 100 mL) and washed further with deionised water (2x 100 mL). the organic layer was dried over MgSO_4_ and the solvent removed under reduced pressure to yield a yellow solid. (4.70 g, 95%). **^1^H NMR** (CDCl_3_, 300 MHz):  δ_H_ 8.05 (d, J = 7.30 Hz, 4H), 7.77 (t, J = 7.43 Hz, 2H), 7.60−7.55 (2t, 5H), 7.35−7.32 (m, 4H), 7.10 (s, 2H), 7.07 (dd, J = 6.56 and 2.74 Hz, 2H), 6.87 (d, J = 7.50 Hz, 2H), 6.75−6.60 (3t + dd, 5H), 5.27 (s, 1H), 3.85−3.65 (3d, 6 H), 3.53 (d, J = 14.48 Hz, 2H).

Literature procedure adapted from Lee *et al.*^7^

Full characterisation in literature procedures from Vézina *et al.*^8^

**5-Bromo-calix[4]arene-25,26,27,28-tetrol**

5-Bromo-26,27,28-tribenzoyloxycalix[4]arene (3.26 g, 4 mmol) was dissolved in ethanol (50 mL) and THF (150 mL). NaOH (12 g) was dissolved in deionised water (100 mL) and the two solutions combined and subsequently heated to reflux overnight, changing colour to dark purple upon heating. The solvents were removed under reduced pressure, and the resulting purple solid was then washed with 1M HCl solution (2x 100 mL), and once each with brine (100 mL) and deionised water (100 mL). The organic phase was dried over MgSO_4_, and solvent removed under reduced pressure. The residue was then washed with methanol (100 mL) to yield a pale beige powder. (1.85 g; 92 %). **^1^H NMR** (CDCl_3_, 300 MHz):  δ_H_ 10.13 (s, 4H), 7.15 (s, 2H), 7.10−7.03 (m, 6H), 6.79−6.73 (m, 3H), 4.25 (br s, 4H), 3.50 (br s, 4H).

Literature procedure adapted from Gutsche *et al.*^6^

Full characterisation in literature procedures from Vézina *et al.*^8^

**5-Formyl-25,27-dipropoxycalix[4]arene**

25,27-Dipropoxycalix[4]arene (1.000 g, 1.97 mmol) was dissolved in dry dichloromethane (45 mL) under a N_2_ atmosphere. Dichloromethyl methyl ether (0.407 g, 3.59 mmol) was added and the resultant mixture stirred for 15 minutes at room temperature. After this time, TiCl_4_ (1.47 g, 7.75 mmol) was added and the stirring continued for another 45 minutes before dichloromethane (5 mL) and water (30 mL) were added sequentially. The organic phase was collected and washed with sat. NaHCO_3_ (2 x 30 mL). The resultant organic phase was dried over Na_2_SO_4_ and rotary evaporated to dryness producing a yellow solid. The product was then purified by flash column chromatography (acetone : dichloromethane (2.5 : 97.5)) yielding an off-white powder (0.474 g, 45 %). **^1^H NMR** (300 MHz, CDCl_3_) δ_H_ 9.79 (s, 1H), 9.31 (s, 1H), 8.26 (s, 1H), 7.63 (s, 2H), 7.06 (d, J = 7.47 Hz, 2H), 6.95 (m, 4H), 6.77 (t, J = 7.55 Hz, 2H), 6.65 (t, J = 7.47 Hz, 1H), 4.31 (dd, J = 13.03, 2.79 Hz, 4H), 4.00 (m, 4H), 3.44 (dd, J = 27.39, 13.07 Hz, 4H), 2.08 (m, J = 6.95 Hz, 4H), 1.33 (t, J = 7.38 Hz, 6H).

Full characterisation in literature procedures from Main *et al.*^3^

**5-Iodo-26,28-dimethoxycalix[4]arene**

25,27-Dimethoxycalix[4]arene (904 mg, 2 mmol), benzyltriammonium dichloroiodate (1.392 g, 4 mmol), and CaCO_3_ (1 g, 10 mmol) were mixed in a round bottomed flask and dissolved in DCM (50 mL) and methanol (30 mL). The reaction mixture was stirred and carried at room temperature overnight. Following that, the reaction crude was washed with brine (100 mL) and with deionised water (2 x 100 mL). The organic layer was then dried over MgSO_4_ and the solvent removed under reduced pressure. The resulting crude product was purified by column chromatography (DCM/Hexane 1:4) to yield an off-white powder. (647 mg, 56%). **^1^H NMR** (300 MHz, CDCl_3_) δ_H_ 7.81 (s, 1H), 7.59 (s, 1H), 7.30 (s, 2H), 7.01 (d, J=7.43 Hz, 2H), 6.80 (m, 4H), 6.65 (m, 3H), 4.19 (dd, 12.94, 20.44 Hz, 4H), 3.90 (s, 6H), 3.30 (dd, 12.94, 28.61 Hz, 4H).

Full characterisation in literature procedures from Hennrich *et al.*^9^

**5-Bromo-25,26,27,28-tetra(*n*-propyloxy)calix[4]arene**

Calix[4]arene (5.00 g, 8.44 mmol) and N-bromosuccinimide (1.607 g, 9.03 mmol) in 150 mL of 2-butanone was stirred for 1 day at room temperature under N_2_. The mixture was diluted with water (100 mL) and extracted with CHCl_3_ (50 mL) before being washed with saturated Na_2_SO_3_ and water (3 x 100 mL). After the extract was dried over anhydrous Na_2_SO_4_, the solvent was removed under reduced pressure. The crude product was triturated with MeOH to give a beige solid (4.71 g, 83 %).**^1^H NMR** (400 MHz, CDCl_3_) δ_H_ 6.85 (m, 11H), 4.49 (m, 4H), 3.95 (m, 8H), 3.21 (m, 4H), 1.99 (m, 8H), 1.10 (m, 12H).

Full characterisation in literature procedures from de la Vega *et al*.^10a^ and Fong, A. (2019). “The Synthesis and Coordination Chemistry of Methylene Bridge-Substituted Calix[4]arenes” Angela Fong. PhD. Heriot-Watt University.^10b^

**Bromination of Benzo[c][1,2,5]thiadiazole (Br_2_BTZ)**

Benzo[*c*][1,2,5]thiadiazole (10 g, 73.6 mmol, 1 equiv.) and *N*-bromosuccinimide (NBS) (26.34 g, 147.2 mmol, 2 equiv.) were put into a two-necked round bottomed flask. Concentrated sulfuric acid (100 mL) was added and the reaction mixture heated to 60 ^o^C for 5 hours. An orange liquid and a yellow solid were formed. The solution was then cooled down to room temperature and poured into an ice water mixture. A white powder precipitated that was filtered by gravity, washed with copious amounts of deionised water, and dried under vacuum to yield white powder. The product was then recrystallised from chloroform (16.215 g, 75% yield).**^1^H NMR** data (CDCl_3_, 300 MHz, 25.0 ^o^C) δH 7.73 (s, 2H).

Full characterisation in literature procedures from Heiskanen *et al* and de la Cruz *et al.*^11,12^

**General Suzuki-Miyaura cross coupling procedure (4,7-diarylBTZ)**

All 4,7-diarylBTZ were synthesised *via* Suzuki-Miyaura cross-coupling by adapating a literature procedure by Taylor *et al.* unless specified otherwise.^13^ Where specified, the starting materials used for the Suzuki-Miyaura cross-coupling were instead chosen to be 2,1,3-Benzothiadiazole-4,7-bis(boronic acid pinacol ester) (**BTZ-Bpin**) (1 eq.) and the relative brominated calix[4]arene (2.0 eq.).

4,7-Dibromobenzothiadiazole (**Br_2_BTZ**) (1 equiv.), aryl-boronic acid/boronic ester (2.5 equiv.), and K_2_CO_3_ (2 equiv.) were mixed in an oven-dry two-necked round bottomed flask and repeatedly evacuated and back-filled with N_2_ gas twice under Schlenk line. Pd(PPh_3_)_4_ (5 mol%) was then added, and the flask sealed and cycled another three times. THF (40 mL) and water (5 mL) were degassed for 10 minutes and then added to the flask. The resulting mixture was heated with an aluminium heating block up to 70 ^o^C under N_2_ for 2 days. Hence, it was cooled down to room temperature and washed three times with water (3 x 20 mL) and DCM (3 x 20 mL), and organic layer collected and dried over MgSO_4_. DCM was then removed by vacuum and product was recrystallised from hot methylated spirits.

**4,4'-(Benzo[c][1,2,5]thiadiazole-4,7-diyl)diphenol (OH-BTZ)**

Synthesis of the compound was achieved following the literature procedure from Taylor *et al.*^13^

**4,7-bis(4-propoxyphenyl)benzo[c][1,2,5]thiadiazole (Pr-BTZ)**

Synthesis was achieved using the general procedure A for Suzuki-Miyaura coupling using 4-propoxyphenyl boronic acid (450 mg, 2.5 mmol), **Br_2_BTZ** (294 mg, 1.0 mmol), potassium carbonate (276 mg, 2.0 mmol) and Pd(PPh_3_)_4_ (5 mol%). The crude product was recrystallised from hot methylated spirits to yield bright yellow crystals (374 mg, 92%). **^1^H NMR** (CDCl_3_, 300 MHz) δ_H_ 7.85 (d, J=9.31 Hz, 4H), 7.65 (s, 2H), 7.00 (d, J=9.31 Hz, 4H), 3.95 (t, J=6.71 Hz, 4H), 1.79 (q, J=7.38 Hz, 4H), 1.00 (t, J=7.43 HZ, 6H). **UV-Vis** (CHCl_3_) λ_max_ (nm) 411.

Full characterisation in literature procedure from Zhang *et al.*^14^

**4-Bromo-7-phenylbenzo[c][1,2,5]thiadiazole (Br-BTZ-Ph)**

**Br_2_BTZ** (5.88 g, 20.0 mmol), phenyl boronic acid (3.66 g, 30.0 mmol, 1.5 equivalents), potassium carbonate (5.53 g, 2.0 equivalents) and Pd(PPh_3_)_4_ (5 mol%) were added to a dry two-neck round bottom flask. The flask was then evacuated and backfilled with nitrogen three times. Degassed THF (40 mL) and degassed deionised water (5 mL) were added and then the reaction heated to 70 ^o^C overnight. The mixture was allowed to cool to room temperature, poured onto deionised water and extracted with DCM (3 x 25 mL). The combined organic phases were washed with deionised water (50 mL), dried over MgSO_4_ and the solvent removed under reduced pressure. The crude product was then subjected to silica gel column chromatography using DCM: petroleum ether 1:40 (v:v) as the eluent system, yielding the product as a yellow powder (1.050 g, 36%). **^1^H NMR** (CDCl_3_, 300 MHz) δ_H_ 7.94 (d, J = 7.6 Hz, 1 *H*), 7.89 (m, 2 *H*), 7.58 (d, J = 7.6 Hz, 1 *H*), 7.54 (m, 2 *H*), 7.47 (m, 2 *H*). **^13^C NMR** (CDCl_3_, 75.5 MHz) δ_C_ 153.9 (C), 153.2 (C), 136.6 (C), 134.1 (C), 132.3 (CH), 129.2 (CH), 128.7 (CH), 128.3 (CH), 113.1 (C). **IR** 𝜈̅ (cm-1) 3020 (w, C-H str.). **UV-Vis** (CHCl_3_) λ_max_ (nm) 370.

**4-(7-Phenylbenzo[*c*][1,2,5]thiadiazol-4-yl)phenol (mOH-BTZ)**

4-Boronophenol (166 mg, 1.2 mmol), **Br-BTZ-Ph** (291 mg, 1.0 mmol), potassium carbonate (128 mg, 1.0 mmol) and Pd(PPh_3_)_4_ (5 mol%) were added to a two-neck round bottom flask. The flask was then evacuated and backfilled with nitrogen three times. Degassed THF (40 mL) and degassed deionised water (5 mL) were added and the reaction heated to 70 ^o^C for 16 hours. Following this time the mixture was poured onto water and extracted with DCM (3 x 25 mL). The combined organic phases were washed with deionised water (50 mL), dried over MgSO_4_ and the solvent removed under reduced pressure. The crude product was then recrystallised from hot methylated spirits to yield a yellow powder (150 mg, 50%). **^1^H NMR** (CDCl_3_, 300 MHz) δ_H_ 7.96 (m, 2H), 7.89 (m, 2H), 7.77 (d, J = 7.3 Hz, 1H), 7.73 (d, J = 7.3 Hz, 1H), 7.55 (m, 2H), 7.45 (m, 1H), 7.01 (m, 2H), 5.22 (bs, 1H). **^13^C NMR** (CDCl_3_, 75.5 MHz) δ_C_ 156.0 (C), 154.2 (C), 154.2 (C), 137.5 (C), 133.0 (C), 132.8 (C), 130.7 (CH), 130.1 (C), 129.2 (CH), 128.6 (CH), 128.3 (CH), 128.3 (CH), 127.4 (CH), 115.6 (CH). **UV-Vis** (CHCl_3_) λ_max_ (nm) 393. **IR** 𝜈̅ (cm-1) 3250 (w, O-H str.), 3020 (w. C-H str.).

**4,7-bis(4-bromophenyl)benzo[c][1,2,5]thiadiazole (Br-PhBTZ)**

Synthesis of the compound was achieved following the literature procedure from Taylor *et al.*^13^

**4′,4‴-(Benzo[*c*][1,2,5]thiadiazole-4,7-diyl)bis(([1″,1‴-biphenyl]-4-ol)) (OHP-BTZ)**

Synthesis of the compound was achieved following the literature procedure from Zhang et al.^15^

**2B1C**

**Br-BTZ-Ph** (291 mg, 1.0 mmol), bis(pinacolato)diboron (254 mg, 1.0 mmol), [1,1′ bis(diphenylphosphino)ferrocene]dichloropalladium(II) (PdCl_2_(dppf)) (73 mg, 0.1 mmol) and potassium acetate (196 mg, 2.0 mmol) were added to a dry two-neck round bottom flask, which was evacuated and backfilled with nitrogen three times. Dry and degassed 1,4-dioxane (15 mL) was added and the resulting solution heated at 85 ^o^C for 24 h under nitrogen. Following this a degassed suspension of 5,17-Dibromo-26,28-dipropyloxycalix[4]arene (200 mg, 0.3 mmol) in THF (40 mL) was added along with potassium carbonate (249 mg, 1.8 mmol) dissolved in degassed deionised water (5 mL). Pd(PPh_3_)_4_ (5 mol%) was added and the resulting mixture was then heated to 70 ^o^C for a further 16 hours. Following this time, the mixture was cooled to room temperature and poured onto deionised water (50 mL). The product was then extracted with DCM (3 x 50 mL), washed with deionised water (2 x 25 mL) and then dried over MgSO_4_. The solvent was removed under reduced pressure and the crude product purified *via* silica-gel column chromatography using chloroform:hexane 4:1 (v:v) as the eluent to yield a bright yellow powder (98 mg, 36%). **^1^H NMR** (CDCl_3_, 300 MHz) δ_H_ 8.69 (s, 2H), 7.95 (m, 4H), 7.75 (d, J = 7.1 Hz, 2H), 7.74 (s, 4H), 7.70 (d, J = 7.1 Hz, 2H), 7.54 (m, 4H), 7.44 (m, 2H), 7.09 (d, J = 7.5 Hz, 4H), 6.84 (t, J = 7.5 Hz, 2H), 4.45 (d, J = 13.2 Hz, 4H), 4.07 (t, J = 6.0 Hz, 4H), 3.56 (d, J = 13.2 Hz, 4H), 2.15 (m, 4H), 1.38 (t, J = 7.5 Hz, 6H). **^13^C NMR** (CDCl_3_, 75.5 MHz) δ_C_ 154.3 (C), 154.2 (C), 154.1 (C), 152.0 (C), 137.7 (C), 133.7 (C), 133.5 (C), 132.1 (C), 129.5 (CH), 129.2 (CH), 128.6 (CH), 128.3 (CH), 128.3 (C), 128.2 (C), 128.1 (CH), 126.9 (CH), 125.5 (CH), 78.5 (CH_2_), 31.7 (CH_2_), 23.6 (CH_2_), 11.0 (CH_3_). **UV-Vis** (CHCl_3_) λ_max_ (nm) 413. **HRMS** (ESI) m/z: [M]+ Calcd for C₅₈H₄₈N₄O₄S₂ 928.31115; Found 928.31064.

**1B2C**

Synthetic procedure followed as above, *via* Suzuki-Miyaura cross coupling using 2,1,3-Benzothiadiazole-4,7-bis(boronic acid pinacol ester) (**BTZ-BPin**) (194 mg, 0.5 mmol), 5-Bromo-26,28-dipropoxy-calix[4]arene (602 mg, 1.025 mmol) and K_2_CO_3_ (138 mg, 1.0 mmol). The product was purified by wash with hot methanol resulting in a bright ochre powder (484 mg, 84%). **^1^H NMR** (CDCl_3_, 400 MHz) δ_H_ 8.60 (s, 2H), 8.35 (s, 2H), 7.70 (s, 4H), 7.65 (s, 2H), 7.06 (m, 8H), 6.95 (m, 4H), 6.79 (t, J=9.17 Hz, 4H), 6.64 (t, J=8.26 Hz, 2H), 4.41 (d, J=12.96 Hz, 4H), 4.36 (d, J=12.96 Hz, 4H), 4.01 (t, J=6.82 Hz, 8H), 3.74 (t, J=6.75, 8H), 3.54 (d, J=12.96 Hz, 4H), 3.41 (d, J=12.96 Hz, 4H), 2.10 (m, 8H), 1.85 (m, 8H), 1.35 (t, J=7.25 Hz, 12H). **^13^C NMR** {1H} (CDCl_3_, 75.5 MHz) δ_C_ 154.3 (C), 153.9 (C), 153.3 (C), 152.0 (C), 129.4 (CH), 129.0 (CH), 128.4 (CH), 128.3 (C), 128.2 (C), 127.1 (CH), 125.4 (CH), 119.0 (CH), 78.4 (CH_2_), 68.02 (CH_2_), 31.7 (CH_2_), 31.4 (CH_2_), 30.39 (CH), 25.6 (CH_2_), 24.9 (CH), 23.5 (CH), 10.9 (CH). **UV-Vis** (CHCl_3_) λ_max_ (nm) 432. **HRMS** (ESI) m/z: [M]+ Calcd for C₇₄H₇₂N₂O₈S, 1148.50039; Found 1148.50014.

**2B2C**

Synthetic procedure followed as above, *via* Suzuki-Miyaura cross coupling using 2,1,3-Benzothiadiazole-4,7-bis(boronic acid pinacol ester) (**BTZ-BPin**) (194 mg, 0.5 mmol), 5,17-Dibromo-26,28-dipropyloxycalix[4]arene (333 mg, 0.5 mmol) and K_2_CO_3_ (138 mg, 1.0 mmol). The product was purified by wash with hot methylated spirits resulting in a bright ochre powder (563 mg, 86%). **^1^H NMR** (CDCl_3_, 400 MHz) δ_H_ 8.70 (m, 2H), 7.69 (m, 6H), 7.07 (m, 4H), 6.82 (m, 2H), 4.46 (br d, J=13.14 Hz, 4H), 4.05 (m, 4H), 3.55 (br. D, J=13.14 Hz, 4H), 2.17 (m, 4 H), 1.37 (m, 6H). A ^13^C NMR spectra of suitable quality could not be obtained due to the low solubility of **2B2C** in common solvents. **UV-Vis** (CHCl_3_) λ_max_ (nm) 431.

**1B2C-OH**

Synthetic procedure followed as above, *via* Suzuki-Miyaura cross coupling using **BTZ-BPin** (116 mg, 0.3 mmol), 5-Bromo-calix[4]arene-25,26,27,28-tetrol (302 mg, 0.6 mmol) and K_2_CO_3_ (83 mg, 0.6 mmol). The product was purified by wash with hot methanol resulting in a bright yellow powder (178 mg, 60%). **^1^H NMR** (CDCl_3_, 300 MHz) δ_H_ 10.14 (br. s, 4H), 7.16 (s, 2H), 7.08 (m, 6H), 6.77 (t, J=7.83 Hz, 3H), 4.23 (br. s, 4H), 3.53(br. s, 4H). **^13^C NMR** {1H} (CDCl_3_, 75.5 MHz) δ_C_ 147.7 (C), 147.6 (C), 147.0 (C), 131.1 (CH), 131.0 (CH), 130.5 (CH), 130.4 (CH), 129.3 (C), 129.2 (C), 128.3 (CH), 128.2 (CH), 128.0 (CH), 127.9 (CH), 127.6 (CH), 127.4 (C), 127.3 (C), 127.0 (C), 126.5 (C), 126.4 (C), 124.5 (CH), 121.5 (CH), 121.4 (CH), 121.3 (CH), 112.8 (C), 66.9 (CH_2_), 33.2 (C), 30.6 (CH_2_), 30.5 (CH_2_), 30.4 (CH_2_), 29.3 (CH). **UV-Vis** (CHCl_3_) λ_max_ (nm) 384. **HRMS** (ESI) m/z: [M + H]+ Calcd for C₆₂H₄₈N₂O₈S 981.32041; Found 981.32112.

**1B2C-Me**

Synthetic procedure followed as above, *via* Suzuki-Miyaura cross coupling using **BTZ-BPin** (82 mg, 0.212 mmol), 5-Iodo-26,28-dimethoxycalix[4]arene (311 mg, 0.53 mmol) and K_2_CO_3_ (60 mg, 0.42 mmol). The product was purified by recrystallisation from DCM/Methanol, and further washed with hot ethyl acetate, resulting in an ochre solid (156 mg, 71%).

**^1^H NMR** (CDCl_3_, 400 MHz) δ_H_ 8.10 (d, J=12.21 Hz, 2H), 7.95 (m, 2H), 7.74 (m, 8H), 7.555 (m, 2H), 7.09 (d, J=7.58 Hz, 4H), 7.00 (d, J=7.58 Hz, 4H), 6.93 (d, J=6.82 Hz, 4H), 6.77 (t, J=6.82 Hz, 4H), 6.68 (t, J=6.82 Hz, 2H), 4.40 (d, J=14.78Hz, 4H), 4.42 (d, J=14.78Hz, 4H), 4.03 (s, 12H), 3.58 (d, J=13.65 Hz, 4H), 3.45 (d, J=13.65 Hz, 4H). **^13^C NMR** {1H} (CDCl_3_, 75.5 MHz) δ_C_ 153.1 (C), 152.8 (C), 152.6 (C), 152.2 (C), 152.0 (C), 136.6 (C), 132.4 (C), 132.1 (C), 131.8 (C), 131.7 (C), 131.2 (C), 128.6 (C), 128.4 (CH), 128.3 (CH), 128.2 (CH), 127.6 (CH), 127.5 (CH), 127.3 (CH), 127.2 (CH), 127.1 (CH), 126.1 (CH), 126.0 (CH), 124.4 (CH), 118.2 (CH), 62.7 (CH_3_), 30.3 (CH_2_), 30.2 (CH_2_). **UV-Vis** (CHCl_3_) λ_max_ (nm) 414. **HRMS** (ESI) m/z: [M + K]+ Calcd for C₆₆H₅₆N₂O₈S 1075.33890; Found 1075.33830.

**1B2C-Pr**

Synthetic procedure followed as above, *via* Suzuki-Miyaura cross coupling using **BTZ-BPin** (194 mg, 0.5 mmol), 5-Bromo-25,26,27,28-tetra(n-propyloxy)calix[4]arene (738 mg, 1.1 mmol) and K_2_CO_3_ (280 mg, 1 mmol). The product was purified by wash with hot methylated spirits, followed by column chromatography in ethyl acetate/hexane (0.5: 99.5 v/v) resulting in a bright yellow powder (501 mg, 76%). **^1^H NMR** (CDCl_3_, 400 MHz) δ_H_ 7.28 (s, 2H), 7.18 (s, 4H), 6.74 (m 4H), 6.62 (m, 12H), 6.35 (t, J=7.51 Hz, 2H), 4.56 (d, J=12.42 Hz, 4H), 4.50 (d, J=12.42 Hz, 4H), 3.90 (m, 16 H), 3.31 (d, J=12.42 Hz, 4H), 3.18 (d, J=12.42 Hz, 4H), 1.97 (m, 16H), 1.01 (m, 24H). **^13^C NMR** {1H} (CDCl_3_, 75.5 MHz) δ_C_ 156.7 (C), 135.3 (C), 135.1 (C), 129.0 (CH), 128.3 (CH), 128.2 (CH), 128.0 (CH), 127.4 (CH), 122.0 (CH), 121.8 (CH), 31.2 (C), 23.2 (CH_2_), 10.4 (CH_2_), 10.3 (CH_3_). **UV-Vis** (CHCl_3_) λ_max_ (nm) 416. **HRMS** (ESI) m/z: [M + K]+ Calcd for C₈₆H₉₆N₂O₈S 1355.65190; Found 1355.65181.

**1PB2C**

Synthetic procedure followed as above, *via* Suzuki-Miyaura cross coupling using **Br-PhBTZ** (74 mg, 0.165 mmol), 5-(4,4,5,5-tetramethyl-1,3,2-dioxaborolan-2-yl)-26,28-dipropoxy-calix[4]arene (211 mg, 0.33 mmol) and K_2_CO_3_ (46 mg, 0.33 mmol). The product was purified by recrystallisation in hot dichloromethane/ methanol, resulting in a bright yellow powder (167 mg, 78%). **^1^H NMR** (CDCl_3_, 400 MHz) δ_H_ 8.57 (s, 2H), 8.38 (s, 2H), 8.04 (d, J=10.40 Hz, 4H), 7.85 (s, 2H), 7.73 (d, J=10.40 Hz, 4H), 7.39 (s, 4H), 7.07 (d, J=7.52 Hz, 4H), 7.01 (m, 8H), 6.80 (t, J=7.19 Hz, 4H), 6.65 (t, J=7.52 Hz, 2H), 4.42 (d, J= 12.81 Hz, 2H), 4.36 (d, J= 12.81 Hz, 2H), 4.02 (t, J=5.82 Hz, 6H), 3.51 (d, J=13.18 Hz, 4H), 3.42 (d, J=13.18 Hz, 4H), 2.12 (m, 8H), 1.35 (t, J=7.38 Hz, 12 H). **^13^C NMR** {1H} (CDCl_3_, 75.5 MHz) δ_C_ 141.5 (C), 135.3 (C), 133.6 (C), 133.3 (C), 132.9 (C), 131.6 (C), 129.4 (CH), 129.1 (CH), 129.0 (CH), 128.6 (C), 128.5 (CH), 128.1 (C), 127.8 (CH), 127.3 (CH), 126.9 (CH), 125.4 (CH), 119.0 (CH), 78.4 (CH_2_), 31.7 (CH_2_), 31.5 (CH_2_), 23.5 (CH_2_), 10.9 (CH_3_). **UV-Vis** (CHCl_3_) λ_max_ (nm) 415. **HRMS** (ESI) m/z: [M]+ Calcd for C_86_H_80_N_2_O_8_S 1300.56354; Found 1300.56121.

2. UV-Visible Absorption and Emission spectra

Absorbance (A.U.)

| Wavelength (nm) | 1B2C | 2B2C | 1PB2C | 1B2C-Pr | 1B2C-Me | 1B2C-OH | 2B1C |
| --- | --- | --- | --- | --- | --- | --- | --- |
| 300 | 2.18947 | 2.35462 | 2.57689 | 1.82942 | 1.9412 | 2.576 | 1.5598 |
| 300.5 | 2.16513 | 2.32269 | 2.54324 | 1.79574 | 1.91614 | 2.456 | 1.53308 |
| 301 | 2.15066 | 2.2883 | 2.64467 | 1.75818 | 1.87602 | 2.35789 | 1.50636 |
| 301.5 | 2.10987 | 2.27228 | 2.61183 | 1.71699 | 1.86133 | 2.27621 | 1.48251 |
| 302 | 2.09737 | 2.25018 | 2.57607 | 1.69251 | 1.83398 | 2.20653 | 1.45865 |
| 302.5 | 2.06118 | 2.23333 | 2.73179 | 1.66126 | 1.82265 | 2.14547 | 1.43925 |
| 303 | 2.03421 | 2.21602 | 2.5673 | 1.63363 | 1.79108 | 2.11558 | 1.41985 |
| 303.5 | 2.02566 | 2.17836 | 2.5447 | 1.61086 | 1.77988 | 2.08316 | 1.40363 |
| 304 | 1.99803 | 2.16561 | 2.5907 | 1.58444 | 1.76349 | 2.07242 | 1.3874 |
| 304.5 | 1.97434 | 2.14 | 2.58306 | 1.56118 | 1.74651 | 2.05958 | 1.37087 |
| 305 | 1.95 | 2.10947 | 2.59802 | 1.54689 | 1.72325 | 2.08632 | 1.35433 |
| 305.5 | 1.92763 | 2.0876 | 2.58518 | 1.52702 | 1.7088 | 2.10653 | 1.33651 |
| 306 | 1.89868 | 2.07298 | 2.70172 | 1.49794 | 1.67711 | 2.11832 | 1.3187 |
| 306.5 | 1.88026 | 2.03041 | 2.57152 | 1.47686 | 1.66506 | 2.15305 | 1.29739 |
| 307 | 1.84342 | 2.01263 | 2.5894 | 1.44391 | 1.64277 | 2.17326 | 1.27608 |
| 307.5 | 1.81579 | 1.98012 | 2.50406 | 1.41386 | 1.61747 | 2.18063 | 1.25445 |
| 308 | 1.78684 | 1.93965 | 2.53885 | 1.378 | 1.59614 | 2.19663 | 1.23282 |
| 308.5 | 1.75987 | 1.91813 | 2.49155 | 1.34844 | 1.57108 | 2.19684 | 1.21183 |
| 309 | 1.70526 | 1.89474 | 2.4171 | 1.32518 | 1.54795 | 2.18821 | 1.19084 |
| 309.5 | 1.68618 | 1.8614 | 2.42572 | 1.29343 | 1.52036 | 2.18042 | 1.17048 |
| 310 | 1.63816 | 1.82702 | 2.41564 | 1.26363 | 1.48759 | 2.16274 | 1.15013 |
| 310.5 | 1.60263 | 1.79485 | 2.41044 | 1.24206 | 1.47072 | 2.15495 | 1.12818 |
| 311 | 1.56316 | 1.75591 | 2.34672 | 1.21517 | 1.43747 | 2.13705 | 1.10623 |
| 311.5 | 1.54211 | 1.71532 | 2.35387 | 1.18221 | 1.41663 | 2.14232 | 1.08174 |
| 312 | 1.49079 | 1.67263 | 2.35842 | 1.14975 | 1.37723 | 2.14653 | 1.05725 |
| 312.5 | 1.42368 | 1.62749 | 2.28056 | 1.12043 | 1.34494 | 2.15874 | 1.03085 |
| 313 | 1.37434 | 1.58632 | 2.29665 | 1.08311 | 1.30506 | 2.17263 | 1.00445 |
| 313.5 | 1.30592 | 1.54573 | 2.24707 | 1.03707 | 1.26795 | 2.172 | 0.980598 |
| 314 | 1.26974 | 1.50433 | 2.26593 | 1.00242 | 1.23867 | 2.17937 | 0.956743 |
| 314.5 | 1.225 | 1.48187 | 2.19555 | 0.966804 | 1.2141 | 2.16758 | 0.94243 |
| 315 | 1.17829 | 1.4462 | 2.16222 | 0.928762 | 1.19145 | 2.15116 | 0.928117 |
| 315.5 | 1.12632 | 1.43006 | 2.15475 | 0.904773 | 1.17747 | 2.12463 | 0.925573 |
| 316 | 1.10526 | 1.41556 | 2.15231 | 0.881512 | 1.17217 | 2.10568 | 0.923028 |
| 316.5 | 1.08355 | 1.40468 | 2.04616 | 0.874 | 1.17133 | 2.076 | 0.929071 |
| 317 | 1.07632 | 1.38784 | 2.0842 | 0.869397 | 1.17964 | 2.05453 | 0.935115 |
| 317.5 | 1.05395 | 1.37708 | 2.0117 | 0.866247 | 1.18205 | 2.05242 | 0.941794 |
| 318 | 1.04605 | 1.35439 | 2.02373 | 0.869397 | 1.18578 | 2.05116 | 0.948473 |
| 318.5 | 1.0375 | 1.32316 | 2.00634 | 0.8803 | 1.18072 | 2.07495 | 0.945929 |
| 319 | 1.02368 | 1.2993 | 1.96668 | 0.878362 | 1.17265 | 2.10316 | 0.943384 |
| 319.5 | 0.992105 | 1.24901 | 1.95806 | 0.869154 | 1.1541 | 2.14716 | 0.92271 |
| 320 | 0.948026 | 1.19895 | 1.93352 | 0.855827 | 1.12807 | 2.15768 | 0.902036 |
| 320.5 | 0.926316 | 1.14094 | 1.89711 | 0.834262 | 1.0912 | 2.17221 | 0.864186 |
| 321 | 0.869737 | 1.08386 | 1.85566 | 0.800824 | 1.04976 | 2.156 | 0.826336 |
| 321.5 | 0.827632 | 1.02573 | 1.78544 | 0.763509 | 0.998193 | 2.124 | 0.776399 |
| 322 | 0.769737 | 0.954854 | 1.74252 | 0.719166 | 0.938072 | 2.05516 | 0.726463 |
| 322.5 | 0.690132 | 0.877193 | 1.6814 | 0.66489 | 0.872651 | 1.94484 | 0.672392 |
| 323 | 0.642105 | 0.808655 | 1.61704 | 0.605525 | 0.809759 | 1.82926 | 0.618321 |
| 323.5 | 0.578289 | 0.741988 | 1.59818 | 0.552217 | 0.748554 | 1.69411 | 0.566476 |
| 324 | 0.507237 | 0.678713 | 1.50374 | 0.491156 | 0.68494 | 1.55347 | 0.514631 |
| 324.5 | 0.45 | 0.621988 | 1.47968 | 0.444875 | 0.631084 | 1.43537 | 0.467557 |
| 325 | 0.400658 | 0.569357 | 1.43596 | 0.39811 | 0.580964 | 1.31789 | 0.420483 |
| 325.5 | 0.353947 | 0.519649 | 1.3672 | 0.355222 | 0.530482 | 1.20632 | 0.381679 |
| 326 | 0.301316 | 0.475322 | 1.29551 | 0.319118 | 0.489277 | 1.10968 | 0.342875 |
| 326.5 | 0.268421 | 0.437193 | 1.27048 | 0.286407 | 0.450241 | 1.02653 | 0.312659 |
| 327 | 0.228289 | 0.394386 | 1.19343 | 0.249818 | 0.411446 | 0.949053 | 0.282443 |
| 327.5 | 0.193421 | 0.363977 | 1.15897 | 0.21953 | 0.38253 | 0.885263 | 0.257316 |
| 328 | 0.166447 | 0.330292 | 1.1302 | 0.19433 | 0.352169 | 0.825895 | 0.232188 |
| 328.5 | 0.132895 | 0.306433 | 1.07835 | 0.175188 | 0.326386 | 0.781895 | 0.21374 |
| 329 | 0.118421 | 0.28152 | 1.04194 | 0.158226 | 0.303976 | 0.744 | 0.195293 |
| 329.5 | 0.1 | 0.262339 | 0.986671 | 0.139811 | 0.289518 | 0.709263 | 0.180662 |
| 330 | 0.0710526 | 0.242339 | 0.931567 | 0.123334 | 0.271084 | 0.685263 | 0.166031 |
| 330.5 | 0.0546053 | 0.225497 | 0.905072 | 0.110007 | 0.25506 | 0.662105 | 0.156807 |
| 331 | 0.0348684 | 0.211111 | 0.880202 | 0.100073 | 0.244578 | 0.645263 | 0.147583 |
| 331.5 | 0.0263158 | 0.195673 | 0.819571 | 0.088442 | 0.234337 | 0.627368 | 0.139313 |
| 332 | 0.00592105 | 0.189123 | 0.792588 | 0.0833535 | 0.22506 | 0.617895 | 0.131043 |
| 332.5 | 0.00657895 | 0.180468 | 0.75764 | 0.0785074 | 0.217952 | 0.602316 | 0.126908 |
| 333 | -0.00592105 | 0.173801 | 0.735533 | 0.0736613 | 0.21241 | 0.597263 | 0.122774 |
| 333.5 | -0.00723684 | 0.163977 | 0.690995 | 0.0656651 | 0.208072 | 0.592842 | 0.119911 |
| 334 | -0.0118421 | 0.159415 | 0.65751 | 0.0659074 | 0.201807 | 0.590105 | 0.117048 |
| 334.5 | -0.0125 | 0.155789 | 0.629551 | 0.0637267 | 0.199277 | 0.583789 | 0.114186 |
| 335 | -0.0197368 | 0.150175 | 0.601918 | 0.057669 | 0.196627 | 0.581895 | 0.111323 |
| 335.5 | -0.0342105 | 0.145029 | 0.577048 | 0.0569421 | 0.195904 | 0.572632 | 0.109733 |
| 336 | -0.0296053 | 0.141988 | 0.540475 | 0.0487037 | 0.19241 | 0.573895 | 0.108142 |
| 336.5 | -0.0348684 | 0.141287 | 0.539824 | 0.0525806 | 0.192048 | 0.576421 | 0.108779 |
| 337 | -0.0348684 | 0.139181 | 0.497399 | 0.0523383 | 0.191687 | 0.576842 | 0.109415 |
| 337.5 | -0.0217105 | 0.137778 | 0.463914 | 0.0554882 | 0.193253 | 0.577263 | 0.110051 |
| 338 | -0.0348684 | 0.13614 | 0.460176 | 0.0528229 | 0.191928 | 0.579368 | 0.110687 |
| 338.5 | -0.0296053 | 0.135205 | 0.449935 | 0.051369 | 0.193253 | 0.580632 | 0.110687 |
| 339 | -0.0355263 | 0.132749 | 0.418563 | 0.0496729 | 0.19253 | 0.578316 | 0.110687 |
| 339.5 | -0.0519737 | 0.133918 | 0.404259 | 0.0506421 | 0.19241 | 0.579158 | 0.112277 |
| 340 | -0.0440789 | 0.134152 | 0.390117 | 0.0501575 | 0.195542 | 0.582316 | 0.113868 |
| 340.5 | -0.0407895 | 0.13345 | 0.381664 | 0.0525806 | 0.196988 | 0.587158 | 0.117048 |
| 341 | -0.0368421 | 0.135088 | 0.350455 | 0.0533075 | 0.197711 | 0.588421 | 0.120229 |
| 341.5 | -0.0460526 | 0.136023 | 0.337451 | 0.0557306 | 0.199398 | 0.593053 | 0.121501 |
| 342 | -0.0368421 | 0.135322 | 0.335663 | 0.0564575 | 0.200964 | 0.598105 | 0.122774 |
| 342.5 | -0.0348684 | 0.137193 | 0.32526 | 0.0634844 | 0.204096 | 0.601895 | 0.125954 |
| 343 | -0.0381579 | 0.136959 | 0.310631 | 0.0583959 | 0.205301 | 0.604 | 0.129135 |
| 343.5 | -0.0421053 | 0.137661 | 0.314207 | 0.0579113 | 0.206988 | 0.605263 | 0.131361 |
| 344 | -0.0381579 | 0.139883 | 0.313069 | 0.0620305 | 0.209639 | 0.606737 | 0.133588 |
| 344.5 | -0.0407895 | 0.139415 | 0.300878 | 0.0634844 | 0.213012 | 0.612 | 0.13645 |
| 345 | -0.0348684 | 0.140351 | 0.289662 | 0.0654228 | 0.214458 | 0.617474 | 0.139313 |
| 345.5 | -0.0381579 | 0.142807 | 0.294538 | 0.067119 | 0.217229 | 0.623789 | 0.142494 |
| 346 | -0.0342105 | 0.143977 | 0.270969 | 0.0719651 | 0.220602 | 0.626316 | 0.145674 |
| 346.5 | -0.0388158 | 0.144678 | 0.26723 | 0.0709959 | 0.223012 | 0.626737 | 0.149173 |
| 347 | -0.0407895 | 0.147719 | 0.263979 | 0.072692 | 0.226988 | 0.632211 | 0.152672 |
| 347.5 | -0.0394737 | 0.148538 | 0.273082 | 0.0739036 | 0.230482 | 0.640421 | 0.156807 |
| 348 | -0.0322368 | 0.15076 | 0.256339 | 0.0794766 | 0.234578 | 0.645895 | 0.160941 |
| 348.5 | -0.0361842 | 0.154503 | 0.253739 | 0.0855343 | 0.23747 | 0.652421 | 0.165076 |
| 349 | -0.0217105 | 0.156725 | 0.254876 | 0.0874727 | 0.241084 | 0.658947 | 0.169211 |
| 349.5 | -0.0263158 | 0.15848 | 0.266092 | 0.0894112 | 0.244217 | 0.66 | 0.173028 |
| 350 | -0.0309211 | 0.161871 | 0.260891 | 0.0918343 | 0.248434 | 0.665895 | 0.176845 |
| 350.5 | -0.025 | 0.163275 | 0.246261 | 0.0942573 | 0.252892 | 0.674526 | 0.181298 |
| 351 | -0.0223684 | 0.166316 | 0.251463 | 0.0981342 | 0.256145 | 0.676842 | 0.185751 |
| 351.5 | -0.0243421 | 0.167135 | 0.261866 | 0.103707 | 0.260361 | 0.685684 | 0.190204 |
| 352 | -0.0184211 | 0.171345 | 0.265442 | 0.105888 | 0.265783 | 0.691158 | 0.194656 |
| 352.5 | -0.0164474 | 0.174971 | 0.280559 | 0.109523 | 0.271566 | 0.700842 | 0.199427 |
| 353 | -0.0118421 | 0.177193 | 0.273895 | 0.114126 | 0.274458 | 0.706316 | 0.204198 |
| 353.5 | -0.00723684 | 0.18 | 0.289662 | 0.118246 | 0.28 | 0.710526 | 0.209288 |
| 354 | -0.00526316 | 0.184678 | 0.274707 | 0.121638 | 0.286265 | 0.716632 | 0.214377 |
| 354.5 | -0.00657895 | 0.188421 | 0.273407 | 0.128423 | 0.290602 | 0.723579 | 0.219466 |
| 355 | 0.000657895 | 0.190643 | 0.284298 | 0.13448 | 0.294458 | 0.730947 | 0.224555 |
| 355.5 | 0.00197368 | 0.194152 | 0.273407 | 0.138115 | 0.300964 | 0.740421 | 0.23028 |
| 356 | 0.00592105 | 0.197076 | 0.283322 | 0.140296 | 0.305663 | 0.746316 | 0.236005 |
| 356.5 | 0.00263158 | 0.199532 | 0.288849 | 0.145869 | 0.310241 | 0.753263 | 0.242048 |
| 357 | 0.00723684 | 0.204094 | 0.29909 | 0.148776 | 0.313976 | 0.757895 | 0.248092 |
| 357.5 | 0.00657895 | 0.20807 | 0.289824 | 0.158469 | 0.321446 | 0.763579 | 0.254453 |
| 358 | 0.0131579 | 0.21193 | 0.302666 | 0.163315 | 0.326145 | 0.771158 | 0.260814 |
| 358.5 | 0.0125 | 0.214854 | 0.311118 | 0.166707 | 0.33241 | 0.778526 | 0.265903 |
| 359 | 0.0125 | 0.219649 | 0.312419 | 0.17228 | 0.33759 | 0.787158 | 0.270992 |
| 359.5 | 0.0184211 | 0.223392 | 0.313557 | 0.179792 | 0.343855 | 0.790947 | 0.277354 |
| 360 | 0.0210526 | 0.227251 | 0.320059 | 0.185849 | 0.348434 | 0.796421 | 0.283715 |
| 360.5 | 0.0309211 | 0.231462 | 0.327536 | 0.192149 | 0.35506 | 0.806737 | 0.289758 |
| 361 | 0.0322368 | 0.237427 | 0.327048 | 0.196268 | 0.36012 | 0.816632 | 0.295802 |
| 361.5 | 0.0315789 | 0.24 | 0.338589 | 0.20063 | 0.36747 | 0.822526 | 0.303753 |
| 362 | 0.0355263 | 0.244912 | 0.353381 | 0.21008 | 0.37241 | 0.823368 | 0.311705 |
| 362.5 | 0.0388158 | 0.248187 | 0.35907 | 0.21323 | 0.376988 | 0.833895 | 0.318702 |
| 363 | 0.0414474 | 0.253567 | 0.356632 | 0.221953 | 0.385542 | 0.841474 | 0.3257 |
| 363.5 | 0.0440789 | 0.258246 | 0.366222 | 0.228011 | 0.392169 | 0.843579 | 0.332697 |
| 364 | 0.0539474 | 0.263626 | 0.364922 | 0.233826 | 0.398795 | 0.853474 | 0.339695 |
| 364.5 | 0.0651316 | 0.26807 | 0.367035 | 0.240853 | 0.405783 | 0.860842 | 0.346692 |
| 365 | 0.0651316 | 0.274269 | 0.378576 | 0.250545 | 0.411928 | 0.868421 | 0.35369 |
| 365.5 | 0.0763158 | 0.279649 | 0.384265 | 0.255634 | 0.419157 | 0.873895 | 0.361641 |
| 366 | 0.0736842 | 0.283743 | 0.390767 | 0.265326 | 0.425301 | 0.880421 | 0.369593 |
| 366.5 | 0.0782895 | 0.290292 | 0.390442 | 0.271141 | 0.433133 | 0.885895 | 0.378181 |
| 367 | 0.0855263 | 0.295556 | 0.4171 | 0.278168 | 0.441687 | 0.894737 | 0.386768 |
| 367.5 | 0.0921053 | 0.300234 | 0.416125 | 0.287376 | 0.447349 | 0.900632 | 0.394402 |
| 368 | 0.0940789 | 0.305848 | 0.417588 | 0.293676 | 0.455301 | 0.907789 | 0.402036 |
| 368.5 | 0.0947368 | 0.312982 | 0.428316 | 0.303126 | 0.463012 | 0.914105 | 0.410623 |
| 369 | 0.108553 | 0.31731 | 0.443921 | 0.31088 | 0.469759 | 0.917263 | 0.419211 |
| 369.5 | 0.115132 | 0.322573 | 0.445384 | 0.316937 | 0.47747 | 0.920632 | 0.426527 |
| 370 | 0.114474 | 0.329006 | 0.439532 | 0.324449 | 0.484337 | 0.926105 | 0.433842 |
| 370.5 | 0.128289 | 0.334503 | 0.45855 | 0.333414 | 0.493373 | 0.931368 | 0.443702 |
| 371 | 0.126316 | 0.338012 | 0.48212 | 0.342379 | 0.500602 | 0.938105 | 0.453562 |
| 371.5 | 0.136184 | 0.345965 | 0.482282 | 0.351587 | 0.508193 | 0.942737 | 0.461832 |
| 372 | 0.140132 | 0.352515 | 0.483583 | 0.361522 | 0.515301 | 0.946947 | 0.470102 |
| 372.5 | 0.149342 | 0.359415 | 0.486834 | 0.368791 | 0.523735 | 0.952842 | 0.478053 |
| 373 | 0.160526 | 0.364444 | 0.492848 | 0.378725 | 0.530602 | 0.957684 | 0.486005 |
| 373.5 | 0.165132 | 0.371228 | 0.501788 | 0.385268 | 0.54012 | 0.960632 | 0.496183 |
| 374 | 0.163158 | 0.375673 | 0.518693 | 0.396656 | 0.547952 | 0.961474 | 0.506361 |
| 374.5 | 0.173684 | 0.381637 | 0.509428 | 0.402714 | 0.554337 | 0.966105 | 0.515585 |
| 375 | 0.177632 | 0.388187 | 0.52552 | 0.412164 | 0.562048 | 0.969263 | 0.524809 |
| 375.5 | 0.190132 | 0.39462 | 0.530722 | 0.420887 | 0.570241 | 0.973263 | 0.531807 |
| 376 | 0.201316 | 0.40152 | 0.535598 | 0.428398 | 0.579036 | 0.977684 | 0.538804 |
| 376.5 | 0.206579 | 0.408421 | 0.550878 | 0.440271 | 0.586988 | 0.981053 | 0.548028 |
| 377 | 0.211184 | 0.415439 | 0.563719 | 0.449721 | 0.594699 | 0.986316 | 0.557252 |
| 377.5 | 0.226316 | 0.421053 | 0.572659 | 0.459898 | 0.604699 | 0.986947 | 0.566794 |
| 378 | 0.228947 | 0.428187 | 0.572334 | 0.472014 | 0.612048 | 0.988842 | 0.576336 |
| 378.5 | 0.238158 | 0.434854 | 0.584363 | 0.48001 | 0.619036 | 0.990526 | 0.584924 |
| 379 | 0.253289 | 0.443041 | 0.585013 | 0.490429 | 0.625904 | 0.991158 | 0.593511 |
| 379.5 | 0.253947 | 0.450292 | 0.595579 | 0.49891 | 0.63506 | 0.993263 | 0.604008 |
| 380 | 0.261842 | 0.456491 | 0.609558 | 0.50836 | 0.643133 | 0.993263 | 0.614504 |
| 380.5 | 0.271711 | 0.464678 | 0.619311 | 0.518536 | 0.651325 | 0.996 | 0.623728 |
| 381 | 0.281579 | 0.470058 | 0.63329 | 0.528713 | 0.656988 | 0.997684 | 0.632952 |
| 381.5 | 0.2875 | 0.47848 | 0.637191 | 0.537194 | 0.666265 | 0.996211 | 0.642494 |
| 382 | 0.300658 | 0.485731 | 0.638654 | 0.54834 | 0.673012 | 0.998947 | 0.652036 |
| 382.5 | 0.309868 | 0.493333 | 0.64223 | 0.56094 | 0.683373 | 1 | 0.662532 |
| 383 | 0.319079 | 0.502105 | 0.657022 | 0.568694 | 0.690964 | 0.999789 | 0.673028 |
| 383.5 | 0.330921 | 0.508187 | 0.666612 | 0.577175 | 0.696867 | 0.999368 | 0.683524 |
| 384 | 0.332895 | 0.515205 | 0.677178 | 0.588563 | 0.705783 | 0.996 | 0.69402 |
| 384.5 | 0.348684 | 0.523743 | 0.686443 | 0.597286 | 0.714337 | 0.995789 | 0.701972 |
| 385 | 0.356579 | 0.529591 | 0.692783 | 0.607705 | 0.720482 | 0.994316 | 0.709924 |
| 385.5 | 0.363158 | 0.538129 | 0.707087 | 0.616913 | 0.729518 | 0.992421 | 0.712468 |
| 386 | 0.378947 | 0.545497 | 0.70855 | 0.625394 | 0.735783 | 0.990526 | 0.715013 |
| 386.5 | 0.383553 | 0.554503 | 0.72383 | 0.634844 | 0.744819 | 0.988 | 0.723919 |
| 387 | 0.395395 | 0.560585 | 0.730169 | 0.646959 | 0.751807 | 0.983789 | 0.732824 |
| 387.5 | 0.403289 | 0.567018 | 0.73342 | 0.653744 | 0.760723 | 0.982526 | 0.741412 |
| 388 | 0.409211 | 0.575673 | 0.747724 | 0.663436 | 0.766506 | 0.978947 | 0.75 |
| 388.5 | 0.415789 | 0.58269 | 0.748375 | 0.670463 | 0.772892 | 0.975789 | 0.75827 |
| 389 | 0.427632 | 0.589474 | 0.754389 | 0.680155 | 0.77988 | 0.970316 | 0.766539 |
| 389.5 | 0.434211 | 0.598713 | 0.766092 | 0.689363 | 0.788072 | 0.965053 | 0.775127 |
| 390 | 0.45 | 0.606784 | 0.775845 | 0.698813 | 0.797711 | 0.962526 | 0.783715 |
| 390.5 | 0.460526 | 0.614386 | 0.794538 | 0.710443 | 0.803976 | 0.956632 | 0.791349 |
| 391 | 0.471053 | 0.62269 | 0.791938 | 0.718197 | 0.811084 | 0.953895 | 0.798982 |
| 391.5 | 0.475658 | 0.631345 | 0.803966 | 0.73007 | 0.818072 | 0.949474 | 0.807252 |
| 392 | 0.488158 | 0.640117 | 0.80673 | 0.740005 | 0.826988 | 0.943579 | 0.815522 |
| 392.5 | 0.498026 | 0.646784 | 0.810631 | 0.749939 | 0.83241 | 0.938737 | 0.823473 |
| 393 | 0.503947 | 0.655673 | 0.82526 | 0.756966 | 0.839759 | 0.933053 | 0.831425 |
| 393.5 | 0.5125 | 0.662105 | 0.831437 | 0.766659 | 0.847349 | 0.927158 | 0.839377 |
| 394 | 0.523026 | 0.669708 | 0.845904 | 0.774655 | 0.851566 | 0.921684 | 0.847328 |
| 394.5 | 0.534868 | 0.677895 | 0.848505 | 0.782893 | 0.858554 | 0.915158 | 0.854962 |
| 395 | 0.541447 | 0.687485 | 0.858095 | 0.790162 | 0.864699 | 0.906316 | 0.862595 |
| 395.5 | 0.559211 | 0.694737 | 0.857607 | 0.799855 | 0.871807 | 0.904421 | 0.869275 |
| 396 | 0.569737 | 0.703743 | 0.863622 | 0.807366 | 0.878916 | 0.896211 | 0.875954 |
| 396.5 | 0.577632 | 0.711813 | 0.874512 | 0.816331 | 0.883133 | 0.890737 | 0.88327 |
| 397 | 0.588816 | 0.719883 | 0.893043 | 0.826024 | 0.892048 | 0.883368 | 0.890585 |
| 397.5 | 0.594079 | 0.730526 | 0.887841 | 0.834504 | 0.896988 | 0.874105 | 0.896628 |
| 398 | 0.6125 | 0.73731 | 0.909948 | 0.841531 | 0.902892 | 0.866526 | 0.902672 |
| 398.5 | 0.617105 | 0.744912 | 0.915637 | 0.849043 | 0.908072 | 0.858737 | 0.909033 |
| 399 | 0.623684 | 0.752281 | 0.914337 | 0.857281 | 0.914699 | 0.852 | 0.915394 |
| 399.5 | 0.633553 | 0.759883 | 0.919213 | 0.864551 | 0.919277 | 0.844421 | 0.921438 |

**Figure S1.** Normalised absorption spectra of photosensitiser BTZ-C[4] library

Emission Intensity (a.u.)

| Wavelength (nm) | 1B2C | 2B2C | 1PB2C | 1B2C-Pr | 1B2C-Me | 1B2C-OH | 2B1C |
| --- | --- | --- | --- | --- | --- | --- | --- |
| 450 | 0.0224482 | 0.00766619 | 0.0250576 | 0.00602312 | 0.10376 | 0.185775 | -0.004 |
| 450.5 | 0.0221797 | 0.00799777 | 0.0257394 | 0.00629652 | 0.10866 | 0.194233 | -0.004 |
| 451 | 0.0220234 | 0.00832536 | 0.0263353 | 0.00671311 | 0.113116 | 0.202754 | -0.004 |
| 451.5 | 0.0221114 | 0.00859615 | 0.0269168 | 0.00697844 | 0.117568 | 0.212852 | -0.003 |
| 452 | 0.0221207 | 0.0088612 | 0.0275672 | 0.00723292 | 0.122454 | 0.222551 | -0.003 |
| 452.5 | 0.0219235 | 0.00902101 | 0.028309 | 0.00739806 | 0.126528 | 0.230891 | -0.002 |
| 453 | 0.0220739 | 0.00937685 | 0.0295525 | 0.00775373 | 0.130555 | 0.240651 | -0.002 |
| 453.5 | 0.0220092 | 0.00961911 | 0.0307149 | 0.00829477 | 0.134193 | 0.250211 | -0.002 |
| 454 | 0.0219617 | 0.00982971 | 0.031539 | 0.00859078 | 0.138208 | 0.259808 | -0.001 |
| 454.5 | 0.0221146 | 0.00995958 | 0.0320155 | 0.00898843 | 0.141369 | 0.268928 | -0.001 |
| 455 | 0.0222437 | 0.0101557 | 0.0327228 | 0.00942484 | 0.144323 | 0.278009 | 0 |
| 455.5 | 0.0220547 | 0.0104858 | 0.0334522 | 0.00990491 | 0.147565 | 0.288193 | 0 |
| 456 | 0.0218389 | 0.0110412 | 0.0343504 | 0.010597 | 0.151206 | 0.298603 | 0 |
| 456.5 | 0.0216605 | 0.0114913 | 0.0351317 | 0.0111662 | 0.155137 | 0.308455 | 0 |
| 457 | 0.0214463 | 0.0118098 | 0.0361781 | 0.0117915 | 0.158997 | 0.318278 | 0.001 |
| 457.5 | 0.0213241 | 0.0121859 | 0.0370992 | 0.0122844 | 0.162972 | 0.327746 | 0.001 |
| 458 | 0.021418 | 0.0128288 | 0.0379181 | 0.012993 | 0.166524 | 0.335958 | 0.002 |
| 458.5 | 0.0216939 | 0.0133147 | 0.0388507 | 0.0138392 | 0.170205 | 0.343298 | 0.003 |
| 459 | 0.0220982 | 0.0138811 | 0.0396695 | 0.0147018 | 0.173088 | 0.350877 | 0.003 |
| 459.5 | 0.0226478 | 0.0145184 | 0.0408949 | 0.0155022 | 0.176508 | 0.35935 | 0.004 |
| 460 | 0.023111 | 0.0148914 | 0.0414764 | 0.0161094 | 0.179201 | 0.368537 | 0.005 |
| 460.5 | 0.023605 | 0.0154584 | 0.0425554 | 0.0169103 | 0.182966 | 0.377736 | 0.006 |
| 461 | 0.0239257 | 0.0162945 | 0.0436379 | 0.0178925 | 0.18661 | 0.388097 | 0.007 |
| 461.5 | 0.0239267 | 0.0172217 | 0.0447303 | 0.0192058 | 0.190007 | 0.39719 | 0.008 |
| 462 | 0.0243729 | 0.0179595 | 0.0458381 | 0.020384 | 0.192719 | 0.407077 | 0.009 |
| 462.5 | 0.0247936 | 0.0186356 | 0.0466721 | 0.021523 | 0.196471 | 0.41771 | 0.01 |
| 463 | 0.0249537 | 0.0195681 | 0.0477149 | 0.022707 | 0.199105 | 0.42706 | 0.011 |
| 463.5 | 0.0253603 | 0.0204278 | 0.0489744 | 0.0241323 | 0.203003 | 0.437753 | 0.013 |
| 464 | 0.0259512 | 0.0213219 | 0.0506657 | 0.0255214 | 0.206302 | 0.447036 | 0.014 |
| 464.5 | 0.0265285 | 0.0220736 | 0.0522078 | 0.0272016 | 0.209107 | 0.456306 | 0.016 |
| 465 | 0.0270275 | 0.0229849 | 0.0537855 | 0.0286764 | 0.212259 | 0.466945 | 0.017 |
| 465.5 | 0.0275882 | 0.0239235 | 0.0554359 | 0.0305385 | 0.214849 | 0.478195 | 0.019 |
| 466 | 0.028098 | 0.0248689 | 0.0569426 | 0.0319309 | 0.217621 | 0.487797 | 0.02 |
| 466.5 | 0.0289417 | 0.0260368 | 0.0585937 | 0.0336913 | 0.220649 | 0.497623 | 0.022 |
| 467 | 0.0294953 | 0.0269292 | 0.0604658 | 0.0357835 | 0.224144 | 0.506179 | 0.024 |
| 467.5 | 0.0299265 | 0.0277901 | 0.0618708 | 0.0381284 | 0.226699 | 0.515643 | 0.026 |
| 468 | 0.0306569 | 0.0286758 | 0.0638081 | 0.0406239 | 0.229163 | 0.526275 | 0.028 |
| 468.5 | 0.031239 | 0.0297058 | 0.0652535 | 0.0431258 | 0.231766 | 0.535972 | 0.03 |
| 469 | 0.0319178 | 0.0308469 | 0.0669636 | 0.0454672 | 0.235268 | 0.546289 | 0.032 |
| 469.5 | 0.0326527 | 0.0318984 | 0.0688046 | 0.0483948 | 0.238778 | 0.555719 | 0.034 |
| 470 | 0.0332584 | 0.0330205 | 0.0710041 | 0.0510153 | 0.242661 | 0.56645 | 0.037 |
| 470.5 | 0.0339724 | 0.0340153 | 0.0730197 | 0.0533358 | 0.245789 | 0.577197 | 0.039 |
| 471 | 0.0348355 | 0.0353629 | 0.0750011 | 0.0560391 | 0.250213 | 0.586896 | 0.042 |
| 471.5 | 0.035581 | 0.0364417 | 0.0771238 | 0.0589137 | 0.254143 | 0.597124 | 0.044 |
| 472 | 0.0366252 | 0.0378337 | 0.0791543 | 0.0617567 | 0.257742 | 0.609079 | 0.047 |
| 472.5 | 0.0373886 | 0.0392315 | 0.0811073 | 0.0648609 | 0.262171 | 0.619692 | 0.05 |
| 473 | 0.0385104 | 0.0407985 | 0.0837624 | 0.0679332 | 0.265572 | 0.630865 | 0.053 |
| 473.5 | 0.039679 | 0.0422426 | 0.086541 | 0.0713066 | 0.269823 | 0.642676 | 0.057 |
| 474 | 0.0407903 | 0.0437912 | 0.0891934 | 0.0751368 | 0.273774 | 0.65555 | 0.06 |
| 474.5 | 0.0421059 | 0.0453338 | 0.0916343 | 0.0789662 | 0.278352 | 0.666277 | 0.063 |
| 475 | 0.0430039 | 0.0465803 | 0.0939272 | 0.0828316 | 0.282106 | 0.677039 | 0.067 |
| 475.5 | 0.0442035 | 0.0480173 | 0.0970026 | 0.0871814 | 0.286738 | 0.686618 | 0.071 |
| 476 | 0.0454687 | 0.0495644 | 0.100266 | 0.0915928 | 0.291732 | 0.696673 | 0.075 |
| 476.5 | 0.0468532 | 0.0510064 | 0.103222 | 0.0959113 | 0.296864 | 0.707859 | 0.079 |
| 477 | 0.0485505 | 0.0522528 | 0.106619 | 0.100038 | 0.300995 | 0.717617 | 0.083 |
| 477.5 | 0.0503813 | 0.0538709 | 0.110192 | 0.104493 | 0.30649 | 0.726099 | 0.087 |
| 478 | 0.0521599 | 0.0558706 | 0.113871 | 0.109371 | 0.311145 | 0.735598 | 0.092 |
| 478.5 | 0.0540581 | 0.0579089 | 0.117474 | 0.114543 | 0.317186 | 0.746636 | 0.097 |
| 479 | 0.0561039 | 0.059985 | 0.12115 | 0.119812 | 0.322053 | 0.75714 | 0.102 |
| 479.5 | 0.0578194 | 0.0619907 | 0.124669 | 0.124848 | 0.326974 | 0.767764 | 0.107 |
| 480 | 0.0594524 | 0.0641285 | 0.128328 | 0.130317 | 0.331542 | 0.778475 | 0.112 |
| 480.5 | 0.0614543 | 0.0659683 | 0.132228 | 0.135678 | 0.336271 | 0.788089 | 0.117 |
| 481 | 0.0635714 | 0.0680994 | 0.136022 | 0.141431 | 0.341699 | 0.796781 | 0.123 |
| 481.5 | 0.0655573 | 0.0704135 | 0.140086 | 0.148086 | 0.347172 | 0.805717 | 0.129 |
| 482 | 0.0673138 | 0.0724867 | 0.144348 | 0.154409 | 0.35225 | 0.813576 | 0.135 |
| 482.5 | 0.0694633 | 0.0749292 | 0.148619 | 0.160147 | 0.357626 | 0.822672 | 0.141 |
| 483 | 0.0717673 | 0.0780804 | 0.153493 | 0.165915 | 0.363128 | 0.830842 | 0.147 |
| 483.5 | 0.0739163 | 0.0810342 | 0.157877 | 0.172173 | 0.369844 | 0.838284 | 0.154 |
| 484 | 0.0760755 | 0.0833745 | 0.162895 | 0.178287 | 0.376404 | 0.846092 | 0.161 |
| 484.5 | 0.078354 | 0.0858367 | 0.167463 | 0.184961 | 0.381876 | 0.85483 | 0.167 |
| 485 | 0.080792 | 0.0883 | 0.172769 | 0.191515 | 0.388527 | 0.86536 | 0.174 |
| 485.5 | 0.0832935 | 0.0914984 | 0.178182 | 0.198498 | 0.395408 | 0.873843 | 0.181 |
| 486 | 0.0857994 | 0.0950988 | 0.183686 | 0.206095 | 0.402829 | 0.880795 | 0.188 |
| 486.5 | 0.0885352 | 0.0981783 | 0.189337 | 0.213576 | 0.408284 | 0.886348 | 0.196 |
| 487 | 0.0917487 | 0.101563 | 0.195044 | 0.221053 | 0.414451 | 0.892584 | 0.203 |
| 487.5 | 0.0946855 | 0.104612 | 0.20095 | 0.229899 | 0.41963 | 0.899154 | 0.211 |
| 488 | 0.0979592 | 0.107552 | 0.207554 | 0.238273 | 0.425864 | 0.906193 | 0.219 |
| 488.5 | 0.1012 | 0.110965 | 0.213366 | 0.246175 | 0.432722 | 0.910886 | 0.227 |
| 489 | 0.1047 | 0.11463 | 0.219112 | 0.254383 | 0.440113 | 0.915129 | 0.235 |
| 489.5 | 0.108611 | 0.117844 | 0.22552 | 0.262267 | 0.447801 | 0.920006 | 0.244 |
| 490 | 0.11229 | 0.121198 | 0.233279 | 0.270567 | 0.456943 | 0.926523 | 0.252 |
| 490.5 | 0.116464 | 0.125687 | 0.240399 | 0.279486 | 0.465582 | 0.931709 | 0.261 |
| 491 | 0.120438 | 0.12979 | 0.246638 | 0.288121 | 0.473663 | 0.937016 | 0.27 |
| 491.5 | 0.124201 | 0.133831 | 0.253774 | 0.296874 | 0.481036 | 0.941047 | 0.279 |
| 492 | 0.128449 | 0.137777 | 0.261157 | 0.30696 | 0.488263 | 0.942725 | 0.288 |
| 492.5 | 0.13231 | 0.142213 | 0.268902 | 0.316753 | 0.496759 | 0.94733 | 0.297 |
| 493 | 0.136449 | 0.146793 | 0.276461 | 0.32689 | 0.504775 | 0.951962 | 0.307 |
| 493.5 | 0.141132 | 0.151058 | 0.284553 | 0.336614 | 0.512279 | 0.956408 | 0.316 |
| 494 | 0.145505 | 0.155485 | 0.292809 | 0.346652 | 0.519293 | 0.958555 | 0.326 |
| 494.5 | 0.150393 | 0.161034 | 0.301471 | 0.356783 | 0.52646 | 0.961827 | 0.336 |
| 495 | 0.155371 | 0.165972 | 0.309752 | 0.366557 | 0.533824 | 0.965928 | 0.346 |
| 495.5 | 0.159816 | 0.171093 | 0.318179 | 0.375506 | 0.542139 | 0.968868 | 0.356 |
| 496 | 0.165036 | 0.176029 | 0.326681 | 0.385202 | 0.549815 | 0.969901 | 0.366 |
| 496.5 | 0.170209 | 0.181054 | 0.33568 | 0.395407 | 0.557837 | 0.972737 | 0.377 |
| 497 | 0.176137 | 0.186344 | 0.344807 | 0.405135 | 0.565273 | 0.976192 | 0.387 |
| 497.5 | 0.181955 | 0.192072 | 0.353709 | 0.414904 | 0.573446 | 0.981213 | 0.398 |
| 498 | 0.187391 | 0.197281 | 0.362385 | 0.424781 | 0.582004 | 0.984548 | 0.409 |
| 498.5 | 0.193473 | 0.202428 | 0.371435 | 0.435909 | 0.590523 | 0.987585 | 0.42 |
| 499 | 0.199683 | 0.208227 | 0.381276 | 0.446951 | 0.59929 | 0.990465 | 0.432 |
| 499.5 | 0.205957 | 0.215528 | 0.390351 | 0.457609 | 0.607844 | 0.993723 | 0.443 |
| 500 | 0.212897 | 0.222088 | 0.400137 | 0.467903 | 0.616838 | 0.993956 | 0.454 |
| 500.5 | 0.219264 | 0.227909 | 0.410059 | 0.478524 | 0.624595 | 0.993076 | 0.466 |
| 501 | 0.226455 | 0.234189 | 0.42058 | 0.489468 | 0.632214 | 0.992931 | 0.478 |
| 501.5 | 0.23338 | 0.240301 | 0.431336 | 0.499237 | 0.639613 | 0.994464 | 0.489 |
| 502 | 0.23978 | 0.246374 | 0.441449 | 0.509881 | 0.647281 | 0.997631 | 0.501 |
| 502.5 | 0.246684 | 0.253004 | 0.451627 | 0.520652 | 0.654606 | 0.999828 | 0.513 |
| 503 | 0.254432 | 0.260487 | 0.461556 | 0.530354 | 0.663697 | 1 | 0.524 |
| 503.5 | 0.262404 | 0.26759 | 0.471414 | 0.540207 | 0.670345 | 0.999012 | 0.536 |
| 504 | 0.269494 | 0.273822 | 0.482005 | 0.551529 | 0.678679 | 0.999154 | 0.548 |
| 504.5 | 0.27667 | 0.280556 | 0.493409 | 0.562325 | 0.686582 | 0.999401 | 0.56 |
| 505 | 0.284145 | 0.288062 | 0.503974 | 0.572957 | 0.693132 | 0.997666 | 0.572 |
| 505.5 | 0.29131 | 0.295851 | 0.514055 | 0.583834 | 0.698806 | 0.99459 | 0.583 |
| 506 | 0.298961 | 0.303383 | 0.524967 | 0.59415 | 0.705412 | 0.992337 | 0.595 |
| 506.5 | 0.306864 | 0.311519 | 0.537421 | 0.604828 | 0.714327 | 0.989638 | 0.607 |
| 507 | 0.315223 | 0.319318 | 0.547739 | 0.616019 | 0.722123 | 0.98738 | 0.618 |
| 507.5 | 0.324002 | 0.327497 | 0.558539 | 0.626397 | 0.731191 | 0.987123 | 0.63 |
| 508 | 0.332376 | 0.335654 | 0.568889 | 0.636697 | 0.737106 | 0.985454 | 0.642 |
| 508.5 | 0.341041 | 0.343416 | 0.578672 | 0.646627 | 0.743105 | 0.983525 | 0.653 |
| 509 | 0.349601 | 0.351614 | 0.588704 | 0.656441 | 0.751403 | 0.980751 | 0.665 |
| 509.5 | 0.358912 | 0.360248 | 0.599293 | 0.66629 | 0.758381 | 0.978199 | 0.677 |
| 510 | 0.367404 | 0.368025 | 0.610053 | 0.676252 | 0.767792 | 0.975226 | 0.688 |
| 510.5 | 0.375726 | 0.376383 | 0.621432 | 0.687015 | 0.777001 | 0.970394 | 0.699 |
| 511 | 0.385423 | 0.383921 | 0.632813 | 0.698198 | 0.78734 | 0.964897 | 0.711 |
| 511.5 | 0.394353 | 0.392931 | 0.643185 | 0.709296 | 0.796105 | 0.960864 | 0.722 |
| 512 | 0.40399 | 0.401464 | 0.653956 | 0.719357 | 0.804604 | 0.957367 | 0.733 |
| 512.5 | 0.41323 | 0.410259 | 0.664243 | 0.72911 | 0.813322 | 0.952597 | 0.744 |
| 513 | 0.423091 | 0.419744 | 0.674508 | 0.739234 | 0.821909 | 0.946175 | 0.755 |
| 513.5 | 0.433788 | 0.428994 | 0.685689 | 0.749211 | 0.829931 | 0.939971 | 0.766 |
| 514 | 0.444016 | 0.437969 | 0.696084 | 0.759111 | 0.837951 | 0.935659 | 0.776 |
| 514.5 | 0.453636 | 0.446469 | 0.707048 | 0.767483 | 0.84454 | 0.930361 | 0.787 |
| 515 | 0.463823 | 0.454691 | 0.717949 | 0.776898 | 0.851965 | 0.925905 | 0.797 |
| 515.5 | 0.474419 | 0.463764 | 0.728888 | 0.787128 | 0.859126 | 0.920803 | 0.808 |
| 516 | 0.48537 | 0.47485 | 0.739047 | 0.796572 | 0.864823 | 0.9161 | 0.818 |
| 516.5 | 0.496238 | 0.485178 | 0.74879 | 0.805472 | 0.87143 | 0.91116 | 0.828 |
| 517 | 0.507653 | 0.495659 | 0.75875 | 0.815185 | 0.879033 | 0.903709 | 0.838 |
| 517.5 | 0.517787 | 0.507397 | 0.769373 | 0.8251 | 0.887166 | 0.897449 | 0.847 |
| 518 | 0.529723 | 0.518475 | 0.780231 | 0.834437 | 0.895518 | 0.891585 | 0.857 |
| 518.5 | 0.540775 | 0.528768 | 0.790369 | 0.844148 | 0.901338 | 0.885305 | 0.866 |
| 519 | 0.55175 | 0.540274 | 0.80004 | 0.853777 | 0.91035 | 0.880699 | 0.875 |
| 519.5 | 0.563112 | 0.551791 | 0.80889 | 0.862105 | 0.917248 | 0.877002 | 0.884 |
| 520 | 0.574604 | 0.562626 | 0.81807 | 0.870668 | 0.923413 | 0.872203 | 0.892 |
| 520.5 | 0.585263 | 0.574209 | 0.827464 | 0.879216 | 0.930709 | 0.866808 | 0.9 |
| 521 | 0.597476 | 0.584166 | 0.835847 | 0.886556 | 0.935893 | 0.861853 | 0.908 |
| 521.5 | 0.608304 | 0.594664 | 0.845349 | 0.893951 | 0.942126 | 0.856632 | 0.915 |
| 522 | 0.61935 | 0.605239 | 0.854827 | 0.902138 | 0.947819 | 0.851195 | 0.922 |
| 522.5 | 0.631248 | 0.615553 | 0.863749 | 0.908781 | 0.952924 | 0.847261 | 0.929 |
| 523 | 0.641766 | 0.626098 | 0.871628 | 0.914963 | 0.956634 | 0.840308 | 0.935 |
| 523.5 | 0.652626 | 0.637011 | 0.880671 | 0.920544 | 0.962493 | 0.833301 | 0.941 |
| 524 | 0.663496 | 0.649248 | 0.889886 | 0.927588 | 0.967292 | 0.82804 | 0.947 |
| 524.5 | 0.674179 | 0.660882 | 0.898507 | 0.93545 | 0.972199 | 0.821221 | 0.952 |
| 525 | 0.684628 | 0.671784 | 0.906031 | 0.942351 | 0.977156 | 0.815061 | 0.957 |
| 525.5 | 0.696074 | 0.682963 | 0.912323 | 0.948008 | 0.980858 | 0.807272 | 0.962 |
| 526 | 0.708159 | 0.693964 | 0.919849 | 0.953119 | 0.984647 | 0.800171 | 0.966 |
| 526.5 | 0.719326 | 0.706383 | 0.926247 | 0.958322 | 0.984971 | 0.794033 | 0.971 |
| 527 | 0.730778 | 0.71702 | 0.932945 | 0.962974 | 0.987735 | 0.788317 | 0.975 |
| 527.5 | 0.74177 | 0.728033 | 0.938967 | 0.966569 | 0.989511 | 0.78165 | 0.978 |
| 528 | 0.752752 | 0.737555 | 0.945308 | 0.972242 | 0.992158 | 0.775953 | 0.981 |
| 528.5 | 0.762887 | 0.747375 | 0.951633 | 0.977696 | 0.994731 | 0.770573 | 0.984 |
| 529 | 0.772316 | 0.757336 | 0.956234 | 0.982742 | 0.995531 | 0.764496 | 0.987 |
| 529.5 | 0.78221 | 0.767271 | 0.961584 | 0.984981 | 0.997115 | 0.756034 | 0.99 |
| 530 | 0.792341 | 0.7777 | 0.966365 | 0.988228 | 0.997043 | 0.748805 | 0.992 |
| 530.5 | 0.802274 | 0.786818 | 0.971492 | 0.991442 | 0.996818 | 0.740262 | 0.993 |
| 531 | 0.811482 | 0.796804 | 0.975751 | 0.993095 | 0.997348 | 0.733247 | 0.995 |
| 531.5 | 0.820452 | 0.806372 | 0.97999 | 0.99513 | 0.998283 | 0.726247 | 0.996 |
| 532 | 0.828603 | 0.815109 | 0.982861 | 0.996874 | 0.999344 | 0.719893 | 0.997 |
| 532.5 | 0.837105 | 0.823295 | 0.986452 | 0.998488 | 1 | 0.713108 | 0.998 |
| 533 | 0.846579 | 0.831091 | 0.988903 | 1 | 0.999304 | 0.704447 | 0.999 |
| 533.5 | 0.853315 | 0.839611 | 0.991268 | 0.999527 | 0.998867 | 0.696316 | 0.999 |
| 534 | 0.860085 | 0.847893 | 0.993089 | 0.998817 | 1 | 0.68808 | 0.999 |
| 534.5 | 0.867937 | 0.85541 | 0.995281 | 0.99764 | 0.998877 | 0.680739 | 1 |
| 535 | 0.875464 | 0.862741 | 0.997477 | 0.997353 | 0.996967 | 0.671731 | 0.999 |
| 535.5 | 0.882691 | 0.87134 | 0.997224 | 0.997358 | 0.993885 | 0.663818 | 0.999 |
| 536 | 0.891275 | 0.87895 | 0.998689 | 0.996348 | 0.990886 | 0.656474 | 0.998 |
| 536.5 | 0.898459 | 0.887012 | 1 | 0.995638 | 0.989641 | 0.649093 | 0.997 |
| 537 | 0.905153 | 0.894459 | 0.999036 | 0.994721 | 0.987178 | 0.640649 | 0.996 |
| 537.5 | 0.911102 | 0.901105 | 0.997884 | 0.99258 | 0.984257 | 0.63203 | 0.994 |
| 538 | 0.916134 | 0.909176 | 0.996748 | 0.990459 | 0.98273 | 0.622839 | 0.992 |
| 538.5 | 0.922845 | 0.915666 | 0.996722 | 0.987976 | 0.981088 | 0.615247 | 0.991 |
| 539 | 0.929185 | 0.921516 | 0.995413 | 0.986958 | 0.978628 | 0.608705 | 0.988 |
| 539.5 | 0.93402 | 0.925624 | 0.99375 | 0.983945 | 0.975453 | 0.599853 | 0.986 |
| 540 | 0.939744 | 0.93006 | 0.99201 | 0.979079 | 0.971172 | 0.59291 | 0.983 |
| 540.5 | 0.945443 | 0.936112 | 0.990796 | 0.974517 | 0.969109 | 0.586173 | 0.98 |
| 541 | 0.950137 | 0.940434 | 0.989584 | 0.969984 | 0.964953 | 0.578022 | 0.977 |
| 541.5 | 0.953846 | 0.945379 | 0.985849 | 0.966199 | 0.96059 | 0.570504 | 0.974 |
| 542 | 0.958092 | 0.950035 | 0.983653 | 0.961051 | 0.957264 | 0.563062 | 0.97 |
| 542.5 | 0.961972 | 0.955358 | 0.981448 | 0.956809 | 0.952686 | 0.555025 | 0.966 |
| 543 | 0.965238 | 0.959588 | 0.979419 | 0.952027 | 0.949535 | 0.545978 | 0.962 |
| 543.5 | 0.968178 | 0.964605 | 0.976837 | 0.94608 | 0.944533 | 0.5368 | 0.958 |
| 544 | 0.970921 | 0.968973 | 0.974504 | 0.940257 | 0.939061 | 0.528613 | 0.954 |
| 544.5 | 0.974282 | 0.974034 | 0.972091 | 0.935108 | 0.934868 | 0.521589 | 0.95 |
| 545 | 0.976863 | 0.978016 | 0.967972 | 0.930426 | 0.92955 | 0.514989 | 0.945 |
| 545.5 | 0.98007 | 0.981717 | 0.963319 | 0.926534 | 0.927342 | 0.508155 | 0.941 |
| 546 | 0.981752 | 0.98503 | 0.958394 | 0.921586 | 0.924435 | 0.501265 | 0.936 |
| 546.5 | 0.98294 | 0.988018 | 0.953352 | 0.915888 | 0.919044 | 0.493817 | 0.932 |
| 547 | 0.984585 | 0.989693 | 0.94834 | 0.910903 | 0.914869 | 0.487487 | 0.927 |
| 547.5 | 0.986516 | 0.991964 | 0.944629 | 0.905345 | 0.908814 | 0.479452 | 0.922 |
| 548 | 0.987892 | 0.99478 | 0.93957 | 0.899305 | 0.902824 | 0.473314 | 0.918 |
| 548.5 | 0.989792 | 0.996447 | 0.935918 | 0.891435 | 0.897861 | 0.468128 | 0.913 |
| 549 | 0.992222 | 0.99681 | 0.930495 | 0.886346 | 0.89143 | 0.461716 | 0.908 |
| 549.5 | 0.994545 | 0.99757 | 0.926691 | 0.880577 | 0.883917 | 0.454419 | 0.902 |

**Figure S2.** Normalised fluorescence spectra of photosensitiser BTZ-C[4] library

3. [NMR Spectra](#_Toc205299235)

^1^H NMR spectrum of **C-Pr2_Bz**

^13^C NMR {1H} spectrum of **C-Pr2_Bz**

^1^H NMR spectrum of **Br-C-Pr2_Bz**

^13^C NMR {1H} spectrum of **Br-C-Pr2_Bz**

^1^H NMR spectrum of **Br-C-Pr2**

^1^H NMR spectrum of **BPin-C-Pr2**

**^13^C NMR {1H} spectrum of BPin-C-Pr2**

^1^H NMR spectrum of **2B1C**

^13^C NMR {1H} spectrum of **2B1C**

^1^H NMR spectrum of **1B2C**

^13^C NMR {1H} spectrum of **1B2C**

^1^H NMR spectrum of **2B2C**

^1^H NMR spectrum of **1B2C-OH**

^13^C NMR {1H} spectrum of **1B2C-OH**

^1^H NMR spectrum of **1B2C-Me**

^13^C NMR {1H} spectrum of **1B2C-Me**

^1^H NMR spectrum of **1B2C-Pr**

^13^C NMR {1H} spectrum of **1B2C-Pr**

^1^H NMR spectrum of **1PB2C**

^13^C NMR {1H} spectrum of **1PB2C**

4. Binding studies and Stern-Volmer plots

Emission spectra in the visible region were recorded using a Perkin Elmer LS 55 fluorescence spectrometer in chloroform solution using quartz cuvettes with a path length of 1 cm. This instrument presents high sensitivity and a low intensity maximum limit. Therefore, the concentrations used in Figures S1-S9 of the host photoactive species differ as each PS required high dilution. Then, stepwise addition of 2 incremental equivalents of guest *α-*terpinene was performed using *ad hoc* stock solutions (1 x10^-6^ mM), to maintain stoichiometric ratios exact while minimising volume change in the cuvette (which was still accounted for in the total volume of solution).

Emission intensity/ /counts

| wavelength/ nm | 0 eq. | 2 eq. | 4 eq. | 6 eq. | 8 eq. | 10 eq. |
| --- | --- | --- | --- | --- | --- | --- |
| 450 | 0.806 | 0.256 | 1.704 | 0.720999 | 17.377 | 0.696999 |
| 450.5 | 0.896451 | 0.320322 | 1.71381 | 0.750483 | 16.3054 | 0.731935 |
| 451 | 1.05819 | 0.420193 | 1.66635 | 0.770903 | 15.2041 | 0.73587 |
| 451.5 | 1.11319 | 0.484354 | 1.59481 | 0.800967 | 14.1323 | 0.655354 |
| 452 | 1.14765 | 0.498129 | 1.54761 | 0.840129 | 13.0211 | 0.610387 |
| 452.5 | 1.11094 | 0.526967 | 1.50035 | 0.869999 | 11.9346 | 0.596387 |
| 453 | 1.08952 | 0.596193 | 1.46274 | 0.995967 | 10.9074 | 0.626612 |
| 453.5 | 1.17013 | 0.780741 | 1.42981 | 1.04981 | 9.85019 | 0.63058 |
| 454 | 1.34826 | 0.89058 | 1.49339 | 1.0459 | 8.74893 | 0.558967 |
| 454.5 | 1.31671 | 0.980322 | 1.45577 | 1.08552 | 7.79126 | 0.540709 |
| 455 | 1.49377 | 1.03929 | 1.45165 | 1.10587 | 6.77416 | 0.549258 |
| 455.5 | 1.56426 | 1.08316 | 1.43793 | 1.11193 | 5.71255 | 0.62858 |
| 456 | 1.74139 | 1.0309 | 1.44871 | 1.13219 | 4.75506 | 0.721258 |
| 456.5 | 1.93932 | 0.956612 | 1.421 | 1.20977 | 3.75284 | 0.738516 |
| 457 | 2.06635 | 1.00555 | 1.38384 | 1.25403 | 2.64661 | 0.693612 |
| 457.5 | 2.4099 | 1.09013 | 1.36074 | 1.29845 | 1.53555 | 0.697677 |
| 458 | 2.66606 | 1.17758 | 1.36377 | 1.35626 | 0.999774 | 0.751225 |
| 458.5 | 2.66661 | 1.27252 | 1.45026 | 1.41297 | 1.03432 | 0.817774 |
| 459 | 2.79922 | 1.34829 | 1.58471 | 1.34603 | 1.00952 | 0.808806 |
| 459.5 | 3.02355 | 1.42413 | 1.671 | 1.38864 | 0.98987 | 0.844129 |
| 460 | 3.13913 | 1.48945 | 1.71919 | 1.41213 | 0.984967 | 0.844064 |
| 460.5 | 3.28642 | 1.50961 | 1.78139 | 1.43603 | 0.980129 | 0.857483 |
| 461 | 3.3579 | 1.53477 | 1.88713 | 1.4649 | 1.03016 | 0.937483 |
| 461.5 | 3.55503 | 1.65026 | 1.93506 | 1.51268 | 1.00555 | 1.06274 |
| 462 | 3.53868 | 1.69965 | 1.97348 | 1.54642 | 1.10926 | 1.13823 |
| 462.5 | 3.59958 | 1.67971 | 2.00706 | 1.51281 | 1.29161 | 1.165 |
| 463 | 3.77171 | 1.79058 | 2.14171 | 1.47403 | 1.30645 | 1.21406 |
| 463.5 | 3.82226 | 1.95126 | 2.15597 | 1.57939 | 1.33132 | 1.28068 |
| 464 | 3.88781 | 2.12735 | 2.24703 | 1.69374 | 1.53442 | 1.3609 |
| 464.5 | 3.99881 | 2.22787 | 2.37726 | 1.70803 | 1.648 | 1.49035 |
| 465 | 4.0941 | 2.44894 | 2.38687 | 1.77452 | 1.7179 | 1.58358 |
| 465.5 | 4.36719 | 2.72481 | 2.50726 | 1.82684 | 1.77258 | 1.60513 |
| 466 | 4.61 | 2.87532 | 2.54635 | 1.89381 | 1.84794 | 1.64971 |
| 466.5 | 4.82777 | 2.95506 | 2.66742 | 1.94681 | 1.93713 | 1.77439 |
| 467 | 5.0791 | 3.15639 | 2.865 | 2.02822 | 2.08577 | 1.85919 |
| 467.5 | 5.23239 | 3.36268 | 2.98087 | 2.15297 | 2.22561 | 1.94384 |
| 468 | 5.39568 | 3.58894 | 3.12032 | 2.28665 | 2.30523 | 2.09106 |
| 468.5 | 5.55865 | 3.80974 | 3.44806 | 2.34855 | 2.32042 | 2.211 |
| 469 | 5.72077 | 4.02597 | 3.76219 | 2.33884 | 2.38513 | 2.36258 |
| 469.5 | 6.11471 | 4.23129 | 3.82906 | 2.53848 | 2.41506 | 2.4999 |
| 470 | 6.46455 | 4.17048 | 4.04448 | 2.61442 | 2.49477 | 2.63197 |
| 470.5 | 6.77906 | 4.21094 | 4.19871 | 2.7861 | 2.66403 | 2.67206 |
| 471 | 7.03871 | 4.42203 | 4.47784 | 2.9491 | 2.709 | 2.68532 |
| 471.5 | 7.28381 | 4.71406 | 4.71681 | 3.32126 | 2.74464 | 2.67642 |
| 472 | 7.42574 | 5.14722 | 4.95087 | 3.46781 | 2.86413 | 2.75748 |
| 472.5 | 7.68245 | 5.50074 | 5.17087 | 3.62619 | 3.15716 | 2.96168 |
| 473 | 7.76913 | 5.91452 | 5.39771 | 3.74561 | 3.39661 | 3.10813 |
| 473.5 | 7.94239 | 6.15626 | 5.57993 | 3.89861 | 3.49565 | 3.31135 |
| 474 | 8.28926 | 6.39381 | 5.82477 | 4.21223 | 3.61403 | 3.50745 |
| 474.5 | 8.61042 | 6.7749 | 6.08316 | 4.63584 | 3.81219 | 3.62323 |
| 475 | 8.91316 | 7.19739 | 6.33258 | 5.0049 | 4.03119 | 3.82758 |
| 475.5 | 9.26555 | 7.68535 | 6.6499 | 5.22887 | 4.35697 | 3.97084 |
| 476 | 9.81829 | 8.102 | 7.024 | 5.46335 | 4.73068 | 4.18213 |
| 476.5 | 10.292 | 8.50893 | 7.20861 | 5.61942 | 4.81961 | 4.50677 |
| 477 | 10.6368 | 9.04545 | 7.5779 | 5.93252 | 4.94345 | 4.8149 |
| 477.5 | 11.3129 | 9.59784 | 8.05429 | 6.26939 | 5.29119 | 5.19242 |
| 478 | 11.8421 | 10.0098 | 8.48255 | 6.65413 | 5.42232 | 5.53519 |
| 478.5 | 12.3844 | 10.3735 | 8.8991 | 7.16152 | 5.845 | 5.75803 |
| 479 | 13.0995 | 10.7631 | 9.37371 | 7.63371 | 6.14258 | 6.10868 |
| 479.5 | 13.8872 | 11.1833 | 9.92368 | 7.86594 | 6.3309 | 6.61506 |
| 480 | 14.4903 | 11.8778 | 10.2042 | 8.32519 | 6.53813 | 7.07406 |
| 480.5 | 15.0847 | 12.2892 | 10.6569 | 8.98835 | 6.98219 | 7.40742 |
| 481 | 15.7227 | 13.0028 | 11.1713 | 9.58319 | 7.41684 | 7.76977 |
| 481.5 | 16.3916 | 13.6338 | 11.6979 | 10.027 | 7.80555 | 8.23287 |
| 482 | 17.1228 | 14.2263 | 12.406 | 10.6752 | 8.16371 | 8.66535 |
| 482.5 | 17.9454 | 14.7296 | 12.7975 | 11.1883 | 8.56593 | 9.05045 |
| 483 | 18.8218 | 15.4087 | 13.312 | 11.8345 | 8.99761 | 9.49061 |
| 483.5 | 19.6727 | 15.966 | 13.8527 | 12.5444 | 9.46255 | 9.98745 |
| 484 | 20.3277 | 16.4561 | 14.3499 | 13.4467 | 9.9191 | 10.4417 |
| 484.5 | 21.3151 | 17.0526 | 14.9136 | 14.3076 | 10.416 | 10.9475 |
| 485 | 21.9582 | 17.9604 | 15.7099 | 15.0215 | 10.9519 | 11.5011 |
| 485.5 | 22.5612 | 19.0189 | 16.1824 | 15.8567 | 11.5192 | 11.9912 |
| 486 | 23.3033 | 20.1623 | 16.801 | 16.8337 | 12.0509 | 12.63 |
| 486.5 | 24.2653 | 21.0742 | 17.4851 | 17.6071 | 12.9135 | 13.261 |
| 487 | 25.2443 | 21.8019 | 18.2073 | 18.0381 | 13.7508 | 13.9347 |
| 487.5 | 26.3066 | 22.5017 | 18.9642 | 18.877 | 14.534 | 14.6109 |
| 488 | 27.1013 | 23.1712 | 19.8026 | 19.8172 | 15.1378 | 15.2371 |
| 488.5 | 28.1399 | 23.8136 | 20.5021 | 20.8395 | 15.6527 | 15.8836 |
| 489 | 29.3626 | 24.7373 | 21.4187 | 21.8851 | 16.5214 | 16.5913 |
| 489.5 | 30.6507 | 25.6307 | 22.3777 | 22.7138 | 17.6166 | 17.3666 |
| 490 | 31.9984 | 26.4867 | 23.4086 | 23.5193 | 18.7465 | 18.5095 |
| 490.5 | 33.0234 | 27.4295 | 24.4911 | 24.3661 | 19.8652 | 19.1047 |
| 491 | 34.1081 | 28.3443 | 25.542 | 25.3622 | 20.6107 | 20.0972 |
| 491.5 | 35.216 | 29.3102 | 26.5071 | 26.4727 | 21.3726 | 20.9849 |
| 492 | 36.3375 | 30.3368 | 27.6622 | 27.874 | 22.228 | 21.7581 |
| 492.5 | 37.4395 | 31.2914 | 28.8451 | 28.8797 | 23.5356 | 22.8038 |
| 493 | 38.7226 | 32.0987 | 30.0808 | 29.5741 | 24.4959 | 23.9385 |
| 493.5 | 40.1374 | 33.1797 | 31.2291 | 30.5261 | 25.527 | 25.1819 |
| 494 | 41.6053 | 34.4998 | 32.1257 | 31.6391 | 26.5554 | 26.6217 |
| 494.5 | 42.9185 | 35.9487 | 33.305 | 32.6955 | 27.7717 | 27.6119 |
| 495 | 44.4465 | 37.083 | 34.4601 | 33.952 | 28.6999 | 28.8637 |
| 495.5 | 46.1036 | 38.11 | 35.838 | 35.3485 | 29.712 | 29.9664 |
| 496 | 47.8571 | 39.4845 | 37.2939 | 36.2453 | 30.7016 | 31.2257 |
| 496.5 | 49.061 | 40.6168 | 38.5172 | 37.3515 | 32.0073 | 32.6312 |
| 497 | 50.5364 | 41.8085 | 39.8855 | 38.9023 | 33.2877 | 34.114 |
| 497.5 | 52.1349 | 43.4714 | 41.6285 | 40.4813 | 34.9321 | 35.5271 |
| 498 | 53.6031 | 44.9827 | 43.4382 | 42.132 | 36.4384 | 37.1446 |
| 498.5 | 55.2662 | 46.3828 | 45.242 | 43.8222 | 37.9129 | 38.7128 |
| 499 | 57.6415 | 48.1697 | 47.3546 | 45.3558 | 39.6376 | 40.1132 |
| 499.5 | 60.1603 | 49.9967 | 49.0604 | 46.8383 | 41.2812 | 41.4344 |
| 500 | 62.347 | 51.2967 | 50.558 | 48.4053 | 42.9659 | 42.8731 |
| 500.5 | 64.4171 | 52.8761 | 52.0107 | 49.9112 | 44.5674 | 44.1794 |
| 501 | 66.8876 | 54.4397 | 53.8945 | 51.4968 | 46.2024 | 46.0622 |
| 501.5 | 69.0511 | 56.318 | 55.5919 | 53.0158 | 48.0393 | 48.1751 |
| 502 | 71.2506 | 58.0156 | 57.5243 | 54.6305 | 49.5165 | 50.0599 |
| 502.5 | 73.2648 | 59.8344 | 59.7263 | 56.4654 | 51.0803 | 51.7776 |
| 503 | 75.3595 | 61.9964 | 61.6304 | 58.3303 | 52.59 | 53.4825 |
| 503.5 | 77.7902 | 63.915 | 63.5354 | 60.1586 | 54.313 | 55.4532 |
| 504 | 80.0328 | 66.273 | 65.6686 | 61.9784 | 56.1532 | 57.6229 |
| 504.5 | 81.8597 | 68.1326 | 67.7979 | 63.7189 | 57.9447 | 59.5562 |
| 505 | 83.9596 | 70.385 | 70.0781 | 65.6983 | 59.4455 | 61.1012 |
| 505.5 | 86.3561 | 72.5455 | 72.1254 | 67.7397 | 61.2458 | 62.7507 |
| 506 | 88.5027 | 74.7469 | 74.2131 | 70.044 | 63.526 | 65.323 |
| 506.5 | 91.1372 | 77.4063 | 76.731 | 72.3488 | 65.2963 | 67.4236 |
| 507 | 94.1685 | 80.3827 | 79.0676 | 74.6995 | 67.0522 | 69.3103 |
| 507.5 | 97.1749 | 83.3406 | 81.651 | 77.0628 | 69.4775 | 71.7112 |
| 508 | 100.284 | 85.5479 | 84.2236 | 79.5963 | 71.3995 | 74.0062 |
| 508.5 | 102.905 | 88.22 | 86.5957 | 82.6525 | 73.5857 | 76.2494 |
| 509 | 105.719 | 91.0788 | 89.1481 | 85.3247 | 76.0785 | 78.2146 |
| 509.5 | 108.288 | 93.7369 | 91.6403 | 87.9467 | 78.7583 | 80.3443 |
| 510 | 111.735 | 96.3138 | 94.2148 | 90.7316 | 80.6143 | 83.0199 |
| 510.5 | 114.425 | 99.5915 | 97.2982 | 93.5894 | 83.1845 | 85.2156 |
| 511 | 116.9 | 103.07 | 100.595 | 96.0311 | 85.871 | 87.6993 |
| 511.5 | 119.347 | 106.024 | 103.753 | 98.8487 | 88.7363 | 90.1052 |
| 512 | 123.256 | 109.275 | 106.926 | 101.293 | 91.2285 | 92.4506 |
| 512.5 | 127.371 | 112.658 | 110.005 | 104.107 | 93.6212 | 94.8816 |
| 513 | 131.636 | 115.701 | 112.271 | 106.485 | 95.9356 | 97.6585 |
| 513.5 | 135.287 | 118.768 | 115.558 | 108.942 | 98.4939 | 100.28 |
| 514 | 139.264 | 121.875 | 118.91 | 111.361 | 101.264 | 102.817 |
| 514.5 | 142.701 | 124.671 | 121.796 | 114.28 | 104.051 | 105.555 |
| 515 | 145.8 | 127.787 | 125.039 | 117.929 | 107.211 | 108.541 |
| 515.5 | 149.865 | 131.57 | 128.721 | 121.136 | 110.041 | 111.412 |
| 516 | 153.507 | 134.471 | 132.211 | 124.641 | 113.07 | 114.425 |
| 516.5 | 157.185 | 138.806 | 135.64 | 127.621 | 116.44 | 117.802 |
| 517 | 160.692 | 142.258 | 139.533 | 130.688 | 119.435 | 120.634 |
| 517.5 | 164.633 | 145.995 | 143.032 | 134.061 | 122.09 | 123.963 |
| 518 | 168.531 | 149.892 | 146.336 | 137.581 | 125.091 | 127.346 |
| 518.5 | 172.272 | 153.479 | 150.265 | 140.954 | 128.337 | 130.822 |
| 519 | 175.974 | 157.561 | 153.933 | 144.427 | 131.347 | 133.615 |
| 519.5 | 179.931 | 160.959 | 157.628 | 147.909 | 134.82 | 136.581 |
| 520 | 184.224 | 165.378 | 161.583 | 151.577 | 137.974 | 140.006 |
| 520.5 | 188.68 | 169.53 | 165.611 | 155.241 | 141.474 | 143.252 |
| 521 | 193.055 | 173.531 | 170.158 | 158.675 | 144.639 | 146.281 |
| 521.5 | 197.502 | 176.875 | 173.942 | 161.597 | 147.805 | 148.861 |
| 522 | 201.176 | 180.529 | 177.147 | 165.205 | 151.72 | 152.095 |
| 522.5 | 204.557 | 183.648 | 180.576 | 169.068 | 155.301 | 155.625 |
| 523 | 208.879 | 187.135 | 184.112 | 172.993 | 158.617 | 158.401 |
| 523.5 | 213.2 | 191.173 | 187.7 | 176.243 | 162.04 | 161.632 |
| 524 | 217.823 | 194.878 | 191.387 | 179.925 | 165.492 | 165.198 |
| 524.5 | 221.713 | 198.786 | 195.295 | 183.616 | 168.99 | 168.596 |
| 525 | 226.05 | 202.361 | 199.868 | 187.056 | 171.853 | 172.044 |
| 525.5 | 230.091 | 206.362 | 203.768 | 190.688 | 175.502 | 175.233 |
| 526 | 233.873 | 209.734 | 207.648 | 194.746 | 178.536 | 178.401 |
| 526.5 | 238.556 | 212.93 | 211.009 | 198.386 | 181.424 | 181.788 |
| 527 | 242.88 | 216.655 | 214.277 | 201.297 | 184.033 | 184.968 |
| 527.5 | 246.467 | 220.392 | 218.347 | 204.575 | 186.665 | 187.737 |
| 528 | 249.873 | 223.783 | 222.027 | 207.722 | 189.387 | 190.04 |
| 528.5 | 252.939 | 227.477 | 226.685 | 211.124 | 192.657 | 192.962 |
| 529 | 256.491 | 231.694 | 230.697 | 214.745 | 195.974 | 195.877 |
| 529.5 | 259.898 | 234.879 | 234.401 | 218.282 | 198.612 | 199.099 |
| 530 | 263.457 | 238.439 | 237.563 | 221.367 | 200.671 | 201.865 |
| 530.5 | 267.646 | 242.713 | 240.534 | 224.103 | 203.438 | 204.847 |
| 531 | 270.331 | 246.407 | 243.582 | 227.243 | 206.111 | 207.861 |
| 531.5 | 274.479 | 250.113 | 246.799 | 229.922 | 208.384 | 210.83 |
| 532 | 278.1 | 252.25 | 249.933 | 233.211 | 211.138 | 213.07 |
| 532.5 | 281.696 | 255.553 | 252.964 | 236.256 | 214.278 | 215.37 |
| 533 | 284.837 | 259.007 | 256.092 | 238.489 | 217.024 | 216.69 |
| 533.5 | 289.269 | 261.904 | 258.935 | 240.806 | 219.537 | 218.318 |
| 534 | 292.417 | 265.169 | 261.95 | 243.052 | 222 | 219.884 |
| 534.5 | 295.475 | 268.304 | 265.085 | 245.228 | 224.151 | 222.244 |
| 535 | 298.769 | 271.387 | 267.922 | 247.655 | 225.923 | 224.47 |
| 535.5 | 302.678 | 273.92 | 270.262 | 250.36 | 227.873 | 226.891 |
| 536 | 305.287 | 276.001 | 272.445 | 252.824 | 229.836 | 229.408 |
| 536.5 | 307.105 | 278.77 | 273.671 | 254.71 | 232.53 | 232.002 |
| 537 | 309.335 | 281.621 | 275.827 | 256.881 | 234.495 | 234.503 |
| 537.5 | 312.717 | 284.3 | 278.573 | 258.719 | 236.166 | 236.355 |
| 538 | 315.328 | 287.021 | 280.978 | 260.522 | 238.11 | 238.101 |
| 538.5 | 317.489 | 289.597 | 282.65 | 261.785 | 239.844 | 240.215 |
| 539 | 320.488 | 291.304 | 285.158 | 263.56 | 241.494 | 241.866 |
| 539.5 | 323.134 | 293.367 | 287.079 | 264.964 | 243.341 | 243.3 |
| 540 | 326.139 | 295.096 | 289.373 | 267.123 | 244.273 | 245.056 |
| 540.5 | 327.997 | 297.664 | 291.531 | 268.566 | 245.997 | 246.13 |
| 541 | 328.851 | 299.867 | 292.833 | 270.767 | 248.159 | 247.234 |
| 541.5 | 331.331 | 302.322 | 293.816 | 271.533 | 250.2 | 249.216 |
| 542 | 333.511 | 303.958 | 294.687 | 272.525 | 252.065 | 250.696 |
| 542.5 | 336.038 | 305.691 | 295.674 | 274.427 | 254.385 | 252.263 |
| 543 | 337.754 | 307.701 | 296.188 | 276.495 | 256.439 | 254.007 |
| 543.5 | 339.77 | 309.383 | 297.674 | 277.937 | 257.972 | 255.703 |
| 544 | 341.534 | 310.124 | 298.428 | 279.757 | 258.732 | 256.201 |
| 544.5 | 342.688 | 310.536 | 299.313 | 281.005 | 259.61 | 257.247 |
| 545 | 344.037 | 311.66 | 300.025 | 282.897 | 260.751 | 257.758 |
| 545.5 | 344.967 | 313.112 | 300.788 | 284.678 | 262.111 | 259.367 |
| 546 | 345.614 | 313.573 | 302.009 | 285.546 | 263.023 | 261.054 |
| 546.5 | 346.727 | 315.267 | 303.143 | 286.535 | 264.293 | 262.533 |
| 547 | 347.43 | 316.127 | 304.526 | 287.218 | 265.822 | 263.523 |
| 547.5 | 348.635 | 317.415 | 304.478 | 288.556 | 265.91 | 264.325 |
| 548 | 349.978 | 317.83 | 304.598 | 289.496 | 266.608 | 264.827 |
| 548.5 | 350.815 | 318.139 | 305.066 | 291.489 | 267.327 | 266.252 |
| 549 | 350.776 | 318.769 | 305.755 | 292.878 | 268.607 | 267.561 |
| 549.5 | 351.439 | 318.533 | 305.849 | 294.064 | 269.551 | 268.612 |

| [a-terp]/ M | I0/I |
| --- | --- |
| 0 | 1 |
| 1e-06 | 1.10867 |
| 2e-06 | 1.15304 |
| 3e-06 | 1.19092 |
| 4e-06 | 1.29808 |
| 5e-06 | 1.3117 |

**Figure S3.** Quenching curves (top) and Stern Volmer plots (bottom) of **1B2C** versus α-terpinene in CHCl_3_ (0, 2, 4, 6, 8, 10 eq.).

Emission intensity/ /counts

| wavelength/ nm | 0 eq. | 2 eq. | 4 eq. | 6 eq. | 8 eq. | 10 eq. |
| --- | --- | --- | --- | --- | --- | --- |
| 450 | 3.412 | -0.382 | 0 | -0.769 | -1.132 | 26.64 |
| 450.5 | 3.2139 | -0.180548 | 0.091677 | -0.626032 | -1.01197 | 24.6359 |
| 451 | 3.07281 | -0.048838 | 0.051451 | -0.479935 | -0.873612 | 22.9017 |
| 451.5 | 2.87468 | 0.012838 | 0.081677 | -0.325935 | -0.604612 | 21.1726 |
| 452 | 2.66361 | 0.085774 | 0.172419 | -0.149645 | -0.459032 | 19.4124 |
| 452.5 | 2.55413 | 0.28058 | 0.228129 | 0.041709 | -0.248354 | 17.7559 |
| 453 | 2.334 | 0.405096 | 0.238258 | 0.214161 | -0.099387 | 16.2431 |
| 453.5 | 2.1359 | 0.485129 | 0.354903 | 0.390161 | 0.093064 | 14.5916 |
| 454 | 2.03906 | 0.694548 | 0.365161 | 0.503322 | 0.347645 | 12.9145 |
| 454.5 | 1.90684 | 0.977258 | 0.461193 | 0.646612 | 0.587516 | 11.3623 |
| 455 | 1.78784 | 1.19061 | 0.557354 | 0.800806 | 0.631806 | 9.60248 |
| 455.5 | 1.69097 | 1.32626 | 0.542193 | 0.827903 | 0.585387 | 7.87348 |
| 456 | 1.56358 | 1.47287 | 0.552258 | 0.877516 | 0.763612 | 6.11368 |
| 456.5 | 1.50203 | 1.60842 | 0.663516 | 1.02406 | 1.01161 | 4.37432 |
| 457 | 1.45397 | 1.76632 | 0.866387 | 1.25152 | 1.24803 | 2.68645 |
| 457.5 | 1.36603 | 1.91322 | 0.906967 | 1.50984 | 1.46661 | 1.02458 |
| 458 | 1.278 | 2.0669 | 0.99829 | 1.781 | 1.66922 | 0.165225 |
| 458.5 | 1.28684 | 2.13687 | 1.0489 | 1.76235 | 1.72426 | 0.160064 |
| 459 | 1.318 | 2.19181 | 1.0999 | 1.79945 | 1.98839 | 0.139451 |
| 459.5 | 1.32748 | 2.26932 | 1.15052 | 1.79571 | 2.16881 | 0.149741 |
| 460 | 1.45545 | 2.41374 | 1.24165 | 1.96726 | 2.25997 | 0.201193 |
| 460.5 | 1.55719 | 2.50242 | 1.4491 | 2.27348 | 2.55394 | 0.252838 |
| 461 | 1.61919 | 2.70184 | 1.49913 | 2.43403 | 2.75261 | 0.304258 |
| 461.5 | 1.70239 | 2.83164 | 1.63987 | 2.50103 | 2.7969 | 0.335129 |
| 462 | 1.94413 | 3.02016 | 1.75684 | 2.62077 | 2.81223 | 0.304161 |
| 462.5 | 2.17371 | 3.13026 | 1.92497 | 2.7991 | 2.88245 | 0.330451 |
| 463 | 2.35455 | 3.15987 | 2.04616 | 3.07781 | 2.93764 | 0.248967 |
| 463.5 | 2.56126 | 3.19274 | 2.14764 | 3.24281 | 3.00745 | 0.228322 |
| 464 | 2.80823 | 3.36552 | 2.25484 | 3.27274 | 3.14542 | 0.543 |
| 464.5 | 3.09358 | 3.58384 | 2.38665 | 3.46258 | 3.30874 | 0.808677 |
| 465 | 3.44064 | 3.83177 | 2.523 | 3.78948 | 3.40352 | 0.849967 |
| 465.5 | 3.66026 | 3.96139 | 2.56297 | 3.9931 | 3.52068 | 1.09268 |
| 466 | 3.82764 | 4.11303 | 2.48164 | 4.11652 | 3.6659 | 1.34177 |
| 466.5 | 4.11413 | 4.33768 | 2.53722 | 4.19771 | 3.79668 | 1.46094 |
| 467 | 4.31726 | 4.52335 | 2.79497 | 4.2799 | 3.89771 | 1.56403 |
| 467.5 | 4.51922 | 4.77084 | 2.96765 | 4.57545 | 4.126 | 1.77074 |
| 468 | 4.68152 | 4.97345 | 3.13003 | 4.72039 | 4.36626 | 1.94013 |
| 468.5 | 4.9629 | 5.2181 | 3.31735 | 4.90029 | 4.49081 | 1.97013 |
| 469 | 5.20564 | 5.56187 | 3.47452 | 4.98813 | 4.66571 | 2.05755 |
| 469.5 | 5.38168 | 5.66971 | 3.69781 | 5.0881 | 4.80652 | 2.21232 |
| 470 | 5.64994 | 5.87939 | 3.713 | 5.14722 | 4.99571 | 2.1281 |
| 470.5 | 5.93665 | 6.23822 | 3.87497 | 5.52042 | 5.44142 | 2.31842 |
| 471 | 6.21745 | 6.52213 | 4.30542 | 5.92687 | 5.91932 | 2.41668 |
| 471.5 | 6.50726 | 6.94635 | 4.49345 | 6.44384 | 6.40274 | 2.42161 |
| 472 | 6.86997 | 7.27416 | 4.8389 | 6.83219 | 6.85903 | 2.55993 |
| 472.5 | 7.08584 | 7.78874 | 4.89787 | 7.06858 | 7.27797 | 2.74565 |
| 473 | 7.32484 | 8.22642 | 5.26181 | 7.38813 | 7.52332 | 2.94174 |
| 473.5 | 7.74674 | 8.49916 | 5.80684 | 7.88748 | 7.71019 | 3.08703 |
| 474 | 8.26245 | 9.06922 | 6.15577 | 8.45016 | 8.12029 | 3.11706 |
| 474.5 | 8.75316 | 9.45468 | 6.68006 | 8.93223 | 8.43761 | 2.9619 |
| 475 | 9.29906 | 9.93539 | 6.83645 | 9.29771 | 8.63435 | 3.20423 |
| 475.5 | 9.68768 | 10.3369 | 7.16474 | 9.64632 | 9.13555 | 3.39045 |
| 476 | 10.2122 | 11.0187 | 7.34729 | 10.072 | 9.70022 | 3.59394 |
| 476.5 | 10.7157 | 11.6544 | 7.71516 | 10.5984 | 10.0871 | 3.78471 |
| 477 | 11.2251 | 12.1908 | 8.01484 | 11.1842 | 10.565 | 4.01161 |
| 477.5 | 11.7806 | 12.8592 | 8.54068 | 11.7001 | 11.0325 | 4.27494 |
| 478 | 12.477 | 13.4041 | 8.94358 | 12.3353 | 11.4847 | 4.74571 |
| 478.5 | 13.2283 | 13.9841 | 9.67903 | 12.6568 | 12.2614 | 5.15535 |
| 479 | 13.8924 | 14.6092 | 10.4036 | 13.2364 | 12.946 | 5.57903 |
| 479.5 | 14.5821 | 15.3428 | 10.7622 | 14.101 | 13.4919 | 5.70281 |
| 480 | 15.3696 | 16.1708 | 11.3021 | 14.7829 | 14.0124 | 5.82019 |
| 480.5 | 16.0726 | 16.8737 | 11.7574 | 15.328 | 14.7325 | 6.40561 |
| 481 | 16.9612 | 17.7082 | 12.4953 | 16.1095 | 15.5367 | 7.01919 |
| 481.5 | 17.9633 | 18.6909 | 13.2757 | 16.8981 | 16.4558 | 7.60235 |
| 482 | 18.9152 | 19.6153 | 14.402 | 17.7013 | 17.2112 | 8.50203 |
| 482.5 | 19.8988 | 20.5379 | 15.0601 | 18.6657 | 18.1189 | 9.15452 |
| 483 | 20.8849 | 21.4753 | 15.9328 | 19.4053 | 19.0839 | 9.77142 |
| 483.5 | 21.7653 | 22.3528 | 16.5668 | 20.1518 | 20.065 | 10.5408 |
| 484 | 22.9391 | 23.329 | 17.706 | 20.7832 | 20.8225 | 11.1463 |
| 484.5 | 24.1013 | 24.6018 | 19.1254 | 21.892 | 21.5548 | 11.6102 |
| 485 | 25.5314 | 25.8787 | 20.1565 | 23.3379 | 22.7512 | 12.2238 |
| 485.5 | 26.7131 | 27.0975 | 21.2792 | 24.5742 | 23.878 | 13.015 |
| 486 | 28.0571 | 28.3955 | 22.6809 | 25.5582 | 24.8573 | 13.828 |
| 486.5 | 29.6622 | 29.6312 | 23.2488 | 26.2646 | 25.7929 | 14.8453 |
| 487 | 31.0912 | 30.9256 | 24.4576 | 27.0005 | 26.5804 | 15.675 |
| 487.5 | 32.5983 | 32.2016 | 25.6921 | 28.0985 | 27.6721 | 16.5316 |
| 488 | 34.2058 | 33.5679 | 27.2087 | 29.8235 | 28.9524 | 17.2251 |
| 488.5 | 35.8177 | 35.0232 | 28.6867 | 31.3685 | 30.2896 | 18.6034 |
| 489 | 37.3697 | 36.6117 | 29.7761 | 32.3843 | 31.7505 | 19.8323 |
| 489.5 | 38.958 | 38.0472 | 30.8827 | 33.7743 | 32.9718 | 21.1088 |
| 490 | 40.5182 | 39.9088 | 31.744 | 35.2671 | 34.5539 | 22.6502 |
| 490.5 | 41.8355 | 41.644 | 33.4222 | 36.9668 | 36.1301 | 23.8764 |
| 491 | 43.8451 | 43.5037 | 35.5447 | 38.9659 | 37.4855 | 25.5153 |
| 491.5 | 45.8227 | 45.3755 | 37.3017 | 40.3979 | 39.0212 | 26.9656 |
| 492 | 47.8883 | 47.2198 | 39.0092 | 42.1309 | 40.5605 | 28.6731 |
| 492.5 | 49.8642 | 49.1565 | 40.6247 | 43.4013 | 42.1545 | 29.8221 |
| 493 | 51.8475 | 51.0132 | 42.2925 | 45.085 | 44.1895 | 31.5595 |
| 493.5 | 53.9808 | 53.2029 | 44.4089 | 46.8593 | 45.9635 | 33.3963 |
| 494 | 56.6232 | 55.1138 | 46.1228 | 49.0693 | 47.2431 | 35.118 |
| 494.5 | 59.1201 | 57.5222 | 48.2068 | 50.8 | 48.8958 | 36.7203 |
| 495 | 61.4388 | 59.6253 | 50.2365 | 52.7141 | 50.9241 | 38.413 |
| 495.5 | 64.1099 | 61.5671 | 51.8708 | 54.7704 | 53.0869 | 40.5412 |
| 496 | 66.8287 | 63.8826 | 54.5677 | 56.6754 | 55.3081 | 42.8514 |
| 496.5 | 69.5932 | 66.6751 | 57.3038 | 59.3606 | 57.5545 | 44.8785 |
| 497 | 72.2213 | 69.8187 | 60.0577 | 62.2472 | 60.3106 | 46.4816 |
| 497.5 | 75.0854 | 72.9526 | 62.6381 | 64.6201 | 62.5046 | 48.3476 |
| 498 | 78.1977 | 75.9842 | 65.7452 | 66.9232 | 64.5776 | 50.2936 |
| 498.5 | 81.5184 | 79.0955 | 68.6004 | 69.3758 | 66.78 | 52.3851 |
| 499 | 85.2596 | 82.3212 | 71.4226 | 72.618 | 69.2615 | 54.565 |
| 499.5 | 88.7015 | 85.5558 | 74.3697 | 75.4621 | 72.2624 | 56.9135 |
| 500 | 92.0308 | 88.4396 | 77.0669 | 78.213 | 75.206 | 59.4341 |
| 500.5 | 95.3024 | 91.4001 | 80.2003 | 80.9785 | 77.8916 | 61.6675 |
| 501 | 99.5054 | 94.552 | 83.5066 | 83.9776 | 80.4273 | 64.5117 |
| 501.5 | 103.199 | 97.7408 | 86.204 | 87.5159 | 83.3863 | 66.8462 |
| 502 | 107.066 | 101.639 | 90.0093 | 91.545 | 87.2043 | 69.6167 |
| 502.5 | 110.985 | 105.284 | 93.8831 | 95.3517 | 90.3714 | 73.2185 |
| 503 | 114.854 | 109.112 | 98.3879 | 99.1598 | 93.0549 | 76.6212 |
| 503.5 | 119.308 | 113.213 | 102.257 | 102.048 | 96.375 | 79.5224 |
| 504 | 123.46 | 117.385 | 106.059 | 104.677 | 100.557 | 82.4596 |
| 504.5 | 128.107 | 121.575 | 109.847 | 108.286 | 104.524 | 85.6512 |
| 505 | 132.838 | 125.938 | 113.594 | 111.69 | 108.044 | 88.1861 |
| 505.5 | 137.448 | 130.317 | 117.434 | 115.446 | 111.347 | 91.0049 |
| 506 | 142.271 | 134.967 | 121.551 | 119.743 | 115.604 | 94.0516 |
| 506.5 | 147.013 | 139.83 | 125.202 | 123.537 | 119.668 | 97.1741 |
| 507 | 152.286 | 144.309 | 129.783 | 128.164 | 122.983 | 100.924 |
| 507.5 | 157.979 | 149.129 | 134.429 | 132.783 | 127.453 | 105.053 |
| 508 | 163.295 | 153.72 | 140.017 | 137.7 | 131.726 | 109.038 |
| 508.5 | 168.777 | 158.478 | 145.987 | 143.009 | 135.177 | 113.368 |
| 509 | 174.099 | 163.35 | 151.187 | 147.64 | 139.774 | 116.927 |
| 509.5 | 178.981 | 168.94 | 156.239 | 152.676 | 144.887 | 121.489 |
| 510 | 184.338 | 174.725 | 161.422 | 158.4 | 149.674 | 126.507 |
| 510.5 | 190.619 | 180.678 | 167.15 | 163.619 | 153.689 | 131.064 |
| 511 | 196.743 | 187.175 | 172.769 | 169.719 | 158.121 | 135.05 |
| 511.5 | 202.835 | 193.144 | 178.244 | 175.833 | 163.342 | 138.678 |
| 512 | 209.303 | 197.995 | 183.187 | 181.199 | 168.145 | 142.627 |
| 512.5 | 215.767 | 202.614 | 188.286 | 185.669 | 173.381 | 147.697 |
| 513 | 221.98 | 208.368 | 194.001 | 191.763 | 178.746 | 152.183 |
| 513.5 | 228.063 | 214.531 | 200.033 | 197.948 | 183.395 | 157.543 |
| 514 | 234.917 | 220.93 | 206.307 | 203.691 | 188.496 | 163.119 |
| 514.5 | 241.156 | 226.636 | 212.607 | 208.872 | 193.38 | 167.658 |
| 515 | 247.767 | 232.732 | 217.856 | 215.396 | 199.174 | 173.125 |
| 515.5 | 254.527 | 239.237 | 223.585 | 221.221 | 204.596 | 179.052 |
| 516 | 261.809 | 245.872 | 229.577 | 227.991 | 209.665 | 184.905 |
| 516.5 | 268.899 | 252.104 | 236.617 | 233.759 | 215.279 | 190.484 |
| 517 | 276.869 | 258.797 | 243.993 | 238.997 | 221.186 | 196.488 |
| 517.5 | 284.015 | 265.388 | 251.225 | 244.698 | 226.651 | 202.262 |
| 518 | 291.034 | 271.943 | 258.28 | 250.677 | 232.211 | 207.453 |
| 518.5 | 298.131 | 279.205 | 264.727 | 256.779 | 238.322 | 212.971 |
| 519 | 307.162 | 286.042 | 270.438 | 262.939 | 244.738 | 219.046 |
| 519.5 | 316.479 | 292.612 | 276.676 | 270.532 | 251.167 | 224.174 |
| 520 | 324.908 | 299.965 | 283.904 | 277.818 | 256.313 | 230.073 |
| 520.5 | 332.075 | 306.951 | 291.975 | 284.952 | 262.061 | 236.043 |
| 521 | 340.366 | 313.806 | 300.337 | 292.202 | 268.17 | 243.079 |
| 521.5 | 349.091 | 320.184 | 307.671 | 298.689 | 274.511 | 249.423 |
| 522 | 356.51 | 327.398 | 315.142 | 306.027 | 281.111 | 256.015 |
| 522.5 | 363.054 | 335.184 | 323.309 | 313.294 | 288.253 | 261.362 |
| 523 | 370.325 | 342.317 | 330.569 | 320.082 | 294.246 | 267.195 |
| 523.5 | 378.664 | 349.649 | 337.011 | 325.541 | 300.331 | 273.684 |
| 524 | 386.992 | 358.057 | 343.983 | 330.512 | 307.315 | 279.626 |
| 524.5 | 395.342 | 366.676 | 350.921 | 337.214 | 313.335 | 286.23 |
| 525 | 404.662 | 374.087 | 357.65 | 343.818 | 319.299 | 291.779 |
| 525.5 | 413.343 | 380.973 | 364.836 | 349.943 | 325.437 | 297.108 |
| 526 | 421.125 | 388.354 | 371.468 | 355.77 | 331.819 | 303.673 |
| 526.5 | 428.606 | 395.272 | 378.438 | 362.226 | 338.211 | 311.018 |
| 527 | 435.581 | 402.673 | 384.237 | 369.585 | 344.676 | 317.326 |
| 527.5 | 443.032 | 409.96 | 392.164 | 376.212 | 351.476 | 323.566 |
| 528 | 451.263 | 417.832 | 399.879 | 383.505 | 356.615 | 330.044 |
| 528.5 | 457.6 | 424.708 | 406.151 | 389.892 | 361.892 | 336.857 |
| 529 | 464.173 | 431.589 | 412.002 | 396.358 | 368.748 | 341.705 |
| 529.5 | 471.365 | 438.204 | 419.156 | 403.07 | 374.917 | 347.144 |
| 530 | 479.343 | 445.296 | 426.081 | 409.609 | 380.84 | 353.832 |
| 530.5 | 487.007 | 452.274 | 434.318 | 415.931 | 385.73 | 359.24 |
| 531 | 494.539 | 458.776 | 439.863 | 422.1 | 390.901 | 365.892 |
| 531.5 | 500.347 | 465.854 | 445.225 | 428.011 | 397.516 | 372.444 |
| 532 | 507.053 | 473.043 | 450.265 | 433.792 | 403.352 | 377.651 |
| 532.5 | 513.495 | 479.961 | 455.997 | 441.561 | 408.291 | 382.697 |
| 533 | 520.184 | 485.836 | 460.858 | 448.377 | 411.986 | 386.269 |
| 533.5 | 527.582 | 492.286 | 465.909 | 456.062 | 417.347 | 389.713 |
| 534 | 534.063 | 498.214 | 471.721 | 462.996 | 424.187 | 394.218 |
| 534.5 | 539.258 | 503.12 | 477.725 | 468.793 | 429.754 | 399.565 |
| 535 | 544.615 | 509.083 | 484.039 | 473.247 | 433.61 | 404.543 |
| 535.5 | 549.73 | 514.623 | 489.137 | 477.508 | 438.018 | 408.354 |
| 536 | 555.601 | 520.603 | 494.119 | 481.848 | 442.441 | 413.761 |
| 536.5 | 561.394 | 526.209 | 498.092 | 485.642 | 446.793 | 416.774 |
| 537 | 565.658 | 532.157 | 502.659 | 490.186 | 449.291 | 421.265 |
| 537.5 | 569.442 | 536.18 | 506.716 | 493.518 | 453.009 | 425.729 |
| 538 | 573.7 | 539.998 | 510.227 | 497.133 | 456.984 | 430.002 |
| 538.5 | 579.81 | 544.276 | 512.866 | 501.029 | 461.407 | 432.492 |
| 539 | 584.908 | 548.949 | 517.892 | 505.204 | 465.566 | 437.007 |
| 539.5 | 588.444 | 552.065 | 521.686 | 510.121 | 468.398 | 440.924 |
| 540 | 592.646 | 554.61 | 525.662 | 513.76 | 471.083 | 443.804 |
| 540.5 | 595.87 | 559.112 | 531.063 | 517.735 | 474.127 | 447.841 |
| 541 | 599.548 | 563.045 | 533.087 | 521.101 | 476.744 | 451.856 |
| 541.5 | 603.59 | 566.439 | 535.791 | 524.46 | 480.278 | 453.72 |
| 542 | 607.956 | 569.114 | 537.612 | 526.793 | 482.219 | 455.935 |
| 542.5 | 611.877 | 571.666 | 540.163 | 528.028 | 483.353 | 457.933 |
| 543 | 615.107 | 575.595 | 542.137 | 529.887 | 485.356 | 460.168 |
| 543.5 | 617.496 | 578.247 | 544.442 | 532.325 | 488.049 | 462.541 |
| 544 | 622.284 | 581.107 | 548.198 | 535.416 | 490.525 | 464.25 |
| 544.5 | 625.936 | 582.443 | 551.781 | 538.298 | 491.81 | 466.682 |
| 545 | 628.5 | 584.431 | 553.778 | 540.881 | 494.126 | 468.101 |
| 545.5 | 630.057 | 586.769 | 556.273 | 542.197 | 496.479 | 468.58 |
| 546 | 632.573 | 589.105 | 556.966 | 543.878 | 498.492 | 469.953 |
| 546.5 | 635.149 | 591.536 | 560.042 | 546.132 | 498.992 | 469.581 |
| 547 | 638.448 | 593.426 | 561.781 | 547.382 | 498.505 | 469.748 |
| 547.5 | 640.734 | 594.912 | 565.542 | 549.356 | 499.867 | 470.851 |
| 548 | 642.893 | 595.719 | 568.43 | 549.968 | 501.65 | 472.842 |
| 548.5 | 644.994 | 597.126 | 571.1 | 550.144 | 504.036 | 476.013 |
| 549 | 646.629 | 598.768 | 572.079 | 549.947 | 506.172 | 478.297 |
| 549.5 | 648.57 | 600.192 | 572.761 | 550.117 | 505.814 | 479.603 |

| [a-terp]/ M | I0/I |
| --- | --- |
| 0 | 1 |
| 1e-06 | 1.08357 |
| 2e-06 | 1.13495 |
| 3e-06 | 1.16883 |
| 4e-06 | 1.28852 |
| 5e-06 | 1.344 |

**Figure S4.** Quenching curves (top) and Stern Volmer plots (bottom) of **2B2C** versus α-terpinene in CHCl_3_ (0, 2, 4, 6, 8, 10 eq.).

Emission intensity/ A.U.

| wavelenth/ nm | 0 eq. | 2 eq. | 4 eq. | 6 eq. | 8 eq. | 10 eq. |
| --- | --- | --- | --- | --- | --- | --- |
| 450 | 1.282 | 3.44256 | -2.21218 | -0.501899 | -4.78 | -1.86707 |
| 450.5 | 1.58277 | 3.73549 | -1.86355 | -0.159404 | -3.69682 | -1.26593 |
| 451 | 2.00126 | 4.02633 | -1.3994 | 0.222779 | -2.90923 | -0.531134 |
| 451.5 | 2.2699 | 4.27832 | -0.96189 | 0.529315 | -2.19066 | -0.00271313 |
| 452 | 2.55442 | 4.51469 | -0.545505 | 1.04756 | -1.40936 | 0.537179 |
| 452.5 | 2.74 | 4.69183 | -0.0787926 | 1.38595 | -0.491631 | 0.99646 |
| 453 | 2.92855 | 4.99612 | 0.420485 | 1.90028 | 0.304532 | 1.72638 |
| 453.5 | 3.19371 | 5.31641 | 0.705959 | 2.37982 | 1.10828 | 2.35275 |
| 454 | 3.37455 | 5.86071 | 0.951898 | 2.76395 | 2.1102 | 2.82119 |
| 454.5 | 3.57542 | 6.10631 | 1.23463 | 3.18545 | 2.85431 | 3.182 |
| 455 | 3.81916 | 6.48994 | 1.54449 | 3.78073 | 3.59225 | 3.58158 |
| 455.5 | 4.30297 | 6.70893 | 2.01932 | 4.1796 | 4.37373 | 3.87599 |
| 456 | 4.51565 | 6.97032 | 2.48221 | 4.58969 | 5.12268 | 4.36516 |
| 456.5 | 4.81339 | 7.35244 | 2.88746 | 4.80131 | 5.78389 | 4.6897 |
| 457 | 5.27055 | 7.69133 | 3.21836 | 5.16817 | 6.48945 | 5.24594 |
| 457.5 | 5.71768 | 8.12279 | 3.74965 | 5.55044 | 7.22779 | 5.86891 |
| 458 | 6.02835 | 8.45915 | 3.99583 | 5.95076 | 7.67683 | 6.20305 |
| 458.5 | 6.15693 | 8.71246 | 3.92783 | 6.26532 | 7.94717 | 6.40915 |
| 459 | 6.24532 | 8.84911 | 4.0952 | 6.41285 | 8.32778 | 6.65265 |
| 459.5 | 6.24522 | 9.13572 | 4.31168 | 6.76536 | 8.48447 | 6.84992 |
| 460 | 6.53003 | 9.59152 | 4.63845 | 6.89797 | 8.6967 | 7.00671 |
| 460.5 | 6.73916 | 9.96001 | 4.98162 | 6.99881 | 8.93502 | 7.28342 |
| 461 | 6.79322 | 10.1889 | 5.02464 | 7.05392 | 9.211 | 7.5701 |
| 461.5 | 6.96935 | 10.5722 | 5.19595 | 7.37403 | 9.39418 | 7.74573 |
| 462 | 7.35271 | 10.986 | 5.28841 | 7.65219 | 9.45607 | 8.13072 |
| 462.5 | 7.78306 | 11.462 | 5.66149 | 8.06438 | 9.83894 | 8.3225 |
| 463 | 8.16752 | 12.0326 | 5.69671 | 8.21722 | 10.3902 | 8.48135 |
| 463.5 | 8.67303 | 12.3206 | 5.78895 | 8.48576 | 10.4525 | 8.7906 |
| 464 | 8.77258 | 12.8452 | 5.82401 | 8.60088 | 10.65 | 9.03879 |
| 464.5 | 8.84413 | 13.0339 | 5.93426 | 8.75465 | 10.6914 | 9.30638 |
| 465 | 8.99448 | 13.1419 | 6.32607 | 8.84565 | 10.8812 | 9.44488 |
| 465.5 | 9.38297 | 13.3328 | 6.65046 | 9.12221 | 10.9711 | 9.7623 |
| 466 | 9.49074 | 13.694 | 6.90506 | 9.41635 | 11.0578 | 10.1612 |
| 466.5 | 9.63506 | 14.0726 | 6.87438 | 9.57927 | 11.4904 | 10.4813 |
| 467 | 9.93597 | 14.5756 | 7.10231 | 10.0023 | 11.759 | 10.6893 |
| 467.5 | 10.2825 | 14.8422 | 7.42975 | 10.3635 | 12.1634 | 11.094 |
| 468 | 10.6307 | 15.1076 | 7.54703 | 10.674 | 12.4629 | 11.4717 |
| 468.5 | 11.0224 | 15.3288 | 7.60436 | 10.7821 | 12.7999 | 11.5877 |
| 469 | 11.502 | 15.5033 | 7.87281 | 10.8477 | 13.1328 | 11.6512 |
| 469.5 | 11.8153 | 15.7735 | 8.28198 | 11.0736 | 13.3013 | 11.8831 |
| 470 | 11.991 | 16.1377 | 8.44077 | 11.3866 | 13.6926 | 12.5359 |
| 470.5 | 12.368 | 16.4848 | 8.71943 | 11.5727 | 14.1078 | 12.8258 |
| 471 | 12.4911 | 17.0869 | 8.94659 | 11.8698 | 14.5356 | 13.2582 |
| 471.5 | 12.855 | 17.5376 | 9.36556 | 11.9579 | 14.8582 | 13.5807 |
| 472 | 13.1727 | 18.0881 | 9.7842 | 12.2026 | 15.1256 | 13.9244 |
| 472.5 | 13.5137 | 18.5377 | 10.2143 | 12.4801 | 15.3753 | 14.2211 |
| 473 | 13.8747 | 18.5227 | 10.5382 | 12.8219 | 15.8426 | 14.5039 |
| 473.5 | 14.2241 | 18.8477 | 10.9535 | 13.0434 | 16.0854 | 14.7751 |
| 474 | 14.9159 | 19.3064 | 11.4316 | 13.2803 | 16.4137 | 15.1565 |
| 474.5 | 15.5484 | 19.7953 | 12.0934 | 13.6623 | 16.3159 | 15.356 |
| 475 | 16.2987 | 20.1128 | 12.5643 | 14.0808 | 16.601 | 15.8275 |
| 475.5 | 17.0617 | 20.2168 | 12.7621 | 14.6748 | 17.2449 | 16.3034 |
| 476 | 17.7628 | 20.7496 | 12.9349 | 15.0728 | 17.6034 | 16.8333 |
| 476.5 | 18.2756 | 21.1768 | 13.4247 | 15.4841 | 17.9979 | 17.156 |
| 477 | 18.7518 | 21.4038 | 13.6262 | 15.8103 | 18.4511 | 17.5815 |
| 477.5 | 19.0416 | 21.5924 | 13.9655 | 16.2363 | 18.8695 | 17.9369 |
| 478 | 19.4607 | 21.899 | 14.2671 | 16.5992 | 19.093 | 18.4416 |
| 478.5 | 19.944 | 22.2091 | 14.9518 | 17.1892 | 19.3312 | 19.0819 |
| 479 | 20.6657 | 22.6964 | 15.4385 | 17.6104 | 19.7183 | 19.3488 |
| 479.5 | 21.2817 | 23.0552 | 15.8474 | 18.0694 | 20.164 | 19.7625 |
| 480 | 22.0766 | 23.749 | 16.4404 | 18.6712 | 20.9676 | 20.1233 |
| 480.5 | 23.044 | 24.3985 | 16.8468 | 19.477 | 21.4661 | 20.3692 |
| 481 | 23.6825 | 25.2239 | 17.0458 | 20.2127 | 21.9818 | 20.6708 |
| 481.5 | 24.4176 | 25.715 | 17.3789 | 20.6088 | 22.3807 | 21.1944 |
| 482 | 25.1491 | 26.4095 | 17.7847 | 20.9899 | 22.7808 | 21.502 |
| 482.5 | 25.861 | 26.9005 | 18.5213 | 21.4967 | 23.2067 | 21.9193 |
| 483 | 26.5921 | 27.5281 | 19.192 | 22.0801 | 23.6689 | 22.4919 |
| 483.5 | 27.2135 | 28.4921 | 19.9093 | 22.4206 | 24.0262 | 23.3352 |
| 484 | 27.7476 | 29.4973 | 20.678 | 22.8387 | 24.4169 | 24.1194 |
| 484.5 | 28.3875 | 30.315 | 21.2354 | 23.3491 | 24.9597 | 24.6027 |
| 485 | 29.5798 | 31.0673 | 21.7088 | 23.8127 | 25.4876 | 25.0604 |
| 485.5 | 30.5228 | 31.8587 | 22.412 | 24.2919 | 25.7111 | 25.6174 |
| 486 | 31.3853 | 32.5228 | 23.5608 | 25.0083 | 26.3351 | 26.2749 |
| 486.5 | 32.395 | 33.1661 | 24.5103 | 25.8044 | 26.8338 | 26.6854 |
| 487 | 33.0131 | 33.9457 | 24.9514 | 26.6143 | 27.2879 | 27.2325 |
| 487.5 | 33.779 | 34.651 | 25.1929 | 27.2956 | 28.0972 | 28.1654 |
| 488 | 34.9698 | 35.4474 | 25.7628 | 28.0128 | 29.0694 | 28.8327 |
| 488.5 | 35.9168 | 36.7338 | 26.4797 | 28.6651 | 29.7509 | 29.6637 |
| 489 | 36.8823 | 37.7881 | 27.2072 | 29.2459 | 30.8809 | 30.4122 |
| 489.5 | 37.3788 | 38.8501 | 27.8911 | 29.8529 | 31.7928 | 31.2141 |
| 490 | 37.9345 | 40.166 | 28.3534 | 30.7921 | 32.8689 | 32.2667 |
| 490.5 | 38.8653 | 41.2557 | 29.4441 | 31.4451 | 33.9239 | 32.7849 |
| 491 | 39.8192 | 42.1484 | 30.3104 | 32.1957 | 34.4681 | 33.658 |
| 491.5 | 40.6011 | 42.8796 | 31.0125 | 33.282 | 35.3935 | 34.3051 |
| 492 | 41.5385 | 43.7878 | 32.0132 | 33.9798 | 36.0356 | 35.2095 |
| 492.5 | 42.54 | 44.9104 | 32.9848 | 34.8821 | 37.0525 | 35.655 |
| 493 | 43.2596 | 45.8205 | 33.9104 | 35.7788 | 37.9296 | 36.3257 |
| 493.5 | 44.0784 | 46.4695 | 34.9979 | 36.5787 | 38.7168 | 36.8587 |
| 494 | 45.4678 | 47.5538 | 35.739 | 37.1623 | 39.7181 | 37.3268 |
| 494.5 | 46.2281 | 48.7432 | 36.5685 | 37.8924 | 40.58 | 38.3723 |
| 495 | 47.3869 | 49.8654 | 37.6432 | 38.798 | 41.3508 | 39.2382 |
| 495.5 | 48.7149 | 50.494 | 38.8725 | 39.6704 | 42.0638 | 40.2495 |
| 496 | 49.6007 | 51.751 | 39.8749 | 40.4509 | 43.1569 | 41.2683 |
| 496.5 | 50.977 | 52.6969 | 41.3055 | 41.4164 | 44.413 | 42.0982 |
| 497 | 51.9955 | 53.9193 | 42.2153 | 42.398 | 45.3264 | 42.9167 |
| 497.5 | 52.9649 | 55.0538 | 43.5144 | 43.4937 | 46.2898 | 43.7679 |
| 498 | 54.1968 | 56.4532 | 44.8539 | 44.3815 | 47.2615 | 44.6611 |
| 498.5 | 55.3498 | 58.0863 | 45.837 | 45.182 | 48.0866 | 45.4798 |
| 499 | 56.4724 | 58.9548 | 46.6333 | 46.6133 | 49.2511 | 45.6209 |
| 499.5 | 57.8426 | 59.5584 | 48.0416 | 48.1302 | 50.644 | 46.5573 |
| 500 | 58.7735 | 60.6183 | 49.0032 | 49.6826 | 51.302 | 48.0435 |
| 500.5 | 59.6595 | 61.6211 | 50.2646 | 50.9978 | 52.17 | 49.3084 |
| 501 | 61.1204 | 62.3951 | 51.6497 | 52.1257 | 53.3819 | 50.1846 |
| 501.5 | 62.3307 | 63.1384 | 52.7677 | 53.017 | 54.5342 | 51.1656 |
| 502 | 63.2268 | 63.8997 | 53.7292 | 53.6711 | 55.4848 | 52.6495 |
| 502.5 | 64.3669 | 64.8056 | 54.6051 | 54.6739 | 56.464 | 53.7483 |
| 503 | 65.6648 | 65.6198 | 55.745 | 55.9654 | 57.419 | 54.4104 |
| 503.5 | 66.5432 | 66.6621 | 57.1336 | 57.012 | 58.366 | 55.555 |
| 504 | 67.7054 | 67.3431 | 58.0978 | 58.1637 | 59.2248 | 56.2369 |
| 504.5 | 68.6316 | 68.4957 | 58.9628 | 59.7244 | 60.2007 | 57.2917 |
| 505 | 69.8049 | 69.0983 | 60.0479 | 61.1913 | 60.7755 | 58.08 |
| 505.5 | 71.6994 | 69.7221 | 61.3281 | 62.3544 | 61.5403 | 58.5138 |
| 506 | 73.6375 | 70.5464 | 62.22 | 63.5223 | 62.2705 | 59.729 |
| 506.5 | 74.9238 | 71.8524 | 63.4687 | 64.4635 | 63.2384 | 60.826 |
| 507 | 76.5915 | 72.946 | 64.4474 | 65.4039 | 63.8649 | 61.9501 |
| 507.5 | 77.8517 | 74.3716 | 65.3578 | 66.7995 | 64.8228 | 62.9863 |
| 508 | 79.1523 | 75.3863 | 66.7551 | 67.6328 | 65.3672 | 64.1398 |
| 508.5 | 80.7719 | 76.5617 | 67.9349 | 68.5964 | 66.1459 | 65.0446 |
| 509 | 82.1366 | 78.0186 | 68.7161 | 69.6629 | 67.1342 | 66.5558 |
| 509.5 | 83.1171 | 78.8409 | 69.7315 | 70.975 | 68.5306 | 67.2699 |
| 510 | 84.6481 | 80.4211 | 70.9821 | 72.308 | 69.4213 | 68.2246 |
| 510.5 | 85.7493 | 81.8084 | 72.197 | 73.4946 | 70.3462 | 68.8782 |
| 511 | 86.4763 | 83.5629 | 73.1725 | 74.534 | 71.2535 | 69.9338 |
| 511.5 | 87.1601 | 84.4245 | 74.514 | 75.1369 | 72.2363 | 71.0227 |
| 512 | 88.2996 | 85.6198 | 75.6197 | 75.7686 | 72.8486 | 72.2155 |
| 512.5 | 89.6863 | 86.7587 | 77.1656 | 76.7526 | 73.8134 | 73.0883 |
| 513 | 91.2628 | 87.638 | 78.2839 | 78.1337 | 74.9239 | 73.7482 |
| 513.5 | 92.5809 | 88.571 | 79.0684 | 79.4818 | 76.0892 | 74.6867 |
| 514 | 93.5024 | 89.1973 | 80.398 | 80.6306 | 77.1581 | 75.0846 |
| 514.5 | 94.8487 | 89.7781 | 81.5589 | 80.7504 | 77.9563 | 76.1912 |
| 515 | 95.5913 | 90.5538 | 82.2845 | 80.9919 | 78.3106 | 76.5122 |
| 515.5 | 96.8675 | 91.4192 | 83.5968 | 81.5809 | 79.1332 | 76.8869 |
| 516 | 97.6273 | 92.6357 | 84.5786 | 82.3914 | 80.1152 | 77.2761 |
| 516.5 | 98.5745 | 93.864 | 84.9179 | 83.1786 | 81.5451 | 77.7674 |
| 517 | 99.9786 | 95.1194 | 85.054 | 84.3743 | 82.3643 | 78.7746 |
| 517.5 | 101.207 | 96.2095 | 85.9089 | 85.6875 | 83.4664 | 79.4051 |
| 518 | 102.431 | 96.8436 | 87.3247 | 86.8516 | 84.5277 | 80.3667 |
| 518.5 | 104.375 | 98.184 | 88.7638 | 87.4239 | 85.6595 | 81.7828 |
| 519 | 105.803 | 99.5305 | 89.5394 | 88.3968 | 86.1353 | 82.7531 |
| 519.5 | 107.463 | 100.787 | 90.4047 | 88.8802 | 86.6289 | 83.3622 |
| 520 | 109.48 | 101.631 | 91.4034 | 89.0982 | 87.3615 | 83.6357 |
| 520.5 | 111.25 | 103.311 | 92.6054 | 89.6875 | 88.5693 | 84.3306 |
| 521 | 111.766 | 104.496 | 93.4887 | 90.396 | 89.7405 | 85.1074 |
| 521.5 | 111.882 | 105.187 | 94.2552 | 91.0061 | 90.6639 | 85.6708 |
| 522 | 112.604 | 106.22 | 94.4823 | 91.9161 | 91.3612 | 85.8726 |
| 522.5 | 113.397 | 106.803 | 95.3398 | 92.6248 | 92.1301 | 86.2083 |
| 523 | 114.31 | 107.284 | 96.0424 | 93.6167 | 92.8575 | 86.3266 |
| 523.5 | 115.861 | 107.836 | 96.4686 | 94.4333 | 93.8347 | 87.0361 |
| 524 | 116.324 | 108.332 | 97.35 | 94.8794 | 94.7778 | 87.9162 |
| 524.5 | 116.591 | 109.008 | 97.6736 | 95.8173 | 95.3984 | 88.1038 |
| 525 | 117.374 | 110.037 | 98.4237 | 96.7286 | 95.4143 | 88.7632 |
| 525.5 | 117.628 | 110.399 | 99.3451 | 96.7805 | 96.4984 | 88.8972 |
| 526 | 118.58 | 110.747 | 99.6881 | 97.1747 | 96.9006 | 90.0934 |
| 526.5 | 119.616 | 110.964 | 99.9353 | 97.7601 | 97.5262 | 90.3352 |
| 527 | 120.621 | 111.442 | 99.7902 | 98.9606 | 97.9223 | 90.6676 |
| 527.5 | 121.145 | 111.432 | 99.3914 | 99.9083 | 98.8349 | 91.1336 |
| 528 | 121.985 | 112.002 | 99.5124 | 101.055 | 99.5307 | 91.3514 |
| 528.5 | 122.384 | 112.589 | 99.4208 | 101.306 | 100.016 | 91.8658 |
| 529 | 122.535 | 112.77 | 99.9246 | 101.702 | 100.589 | 91.9658 |
| 529.5 | 123.165 | 113.397 | 100.34 | 102.302 | 100.847 | 92.8002 |
| 530 | 123.42 | 114.502 | 100.979 | 103.057 | 101.684 | 93.2309 |
| 530.5 | 124.108 | 115.325 | 101.404 | 103.567 | 102.407 | 93.3042 |
| 531 | 124.55 | 116.006 | 101.855 | 104.184 | 103.191 | 93.3614 |
| 531.5 | 125.077 | 116.235 | 102.206 | 104.727 | 103.457 | 94.0927 |
| 532 | 125.254 | 116.471 | 103.353 | 104.956 | 103.35 | 94.7718 |
| 532.5 | 125.273 | 116.736 | 104.063 | 104.952 | 103.089 | 94.5021 |
| 533 | 125.592 | 116.952 | 103.797 | 105.024 | 102.831 | 94.6006 |
| 533.5 | 126.207 | 117.863 | 103.745 | 105.038 | 102.854 | 94.2306 |
| 534 | 126.023 | 117.782 | 103.975 | 105.733 | 102.339 | 93.5157 |
| 534.5 | 125.474 | 116.968 | 104.247 | 105.929 | 102.247 | 93.0955 |
| 535 | 124.585 | 116.528 | 104.712 | 106.355 | 102.585 | 93.3899 |
| 535.5 | 123.786 | 116.367 | 104.948 | 107.079 | 102.286 | 93.8361 |
| 536 | 123.235 | 115.729 | 104.875 | 107.3 | 101.781 | 93.4131 |
| 536.5 | 123.72 | 115.525 | 104.852 | 107.052 | 101.162 | 93.4628 |
| 537 | 123.674 | 116.043 | 104.623 | 107.069 | 100.767 | 93.4163 |
| 537.5 | 123.273 | 115.922 | 105.146 | 106.852 | 100.791 | 93.4543 |
| 538 | 122.929 | 115.99 | 105.066 | 106.713 | 100.819 | 93.1614 |
| 538.5 | 122.85 | 115.866 | 104.989 | 106.475 | 100.793 | 93.2484 |
| 539 | 121.954 | 116.033 | 105.251 | 106.729 | 100.369 | 92.9856 |
| 539.5 | 121.688 | 115.857 | 104.674 | 106.485 | 99.9455 | 92.4082 |
| 540 | 122.113 | 115.107 | 104.898 | 106.245 | 99.3237 | 91.8856 |
| 540.5 | 121.432 | 114.38 | 104.809 | 105.881 | 98.9118 | 91.4656 |
| 541 | 121.714 | 113.323 | 104.597 | 105.983 | 98.2197 | 91.511 |
| 541.5 | 121.306 | 112.719 | 104.362 | 105.957 | 98.0083 | 90.8803 |
| 542 | 120.727 | 112.556 | 104.262 | 105.389 | 97.8254 | 90.5117 |
| 542.5 | 120.319 | 112.327 | 104.595 | 104.93 | 97.1964 | 89.8514 |
| 543 | 119.58 | 111.811 | 105 | 104.253 | 96.3829 | 89.175 |
| 543.5 | 118.898 | 110.934 | 104.781 | 103.387 | 95.9359 | 88.6286 |
| 544 | 118.227 | 110.171 | 104.839 | 103.139 | 95.8553 | 88.5041 |
| 544.5 | 117.949 | 109.469 | 104.402 | 102.433 | 95.5674 | 88.2683 |
| 545 | 117.707 | 108.309 | 103.496 | 101.519 | 95.3005 | 87.7007 |
| 545.5 | 117.617 | 107.3 | 102.684 | 101.014 | 94.1823 | 87.2987 |
| 546 | 116.946 | 106.664 | 102.324 | 100.507 | 93.3743 | 87.4538 |
| 546.5 | 116.556 | 105.605 | 101.791 | 99.8641 | 92.6114 | 87.1535 |
| 547 | 115.946 | 104.688 | 101.326 | 98.7601 | 92.0835 | 86.4157 |
| 547.5 | 115.129 | 103.928 | 100.442 | 98.2628 | 91.7016 | 85.5108 |
| 548 | 114.319 | 103.019 | 99.5428 | 97.5272 | 91.2707 | 85.0261 |
| 548.5 | 113.727 | 103.025 | 99.382 | 97.353 | 90.7505 | 84.4772 |
| 549 | 112.836 | 102.188 | 99.4148 | 97.1515 | 90.107 | 84.1279 |
| 549.5 | 111.574 | 101.509 | 98.2859 | 96.2216 | 90.0069 | 83.2849 |

| [a-terp]/ M | I0/I |
| --- | --- |
| 0 | 1 |
| 1e-06 | 1.06086 |
| 2e-06 | 1.16411 |
| 3e-06 | 1.14936 |
| 4e-06 | 1.22944 |
| 5e-06 | 1.32897 |

**Figure S5.** Quenching curves (top) and Stern Volmer plots (bottom) of **1B2C-Me** versus α-terpinene in CHCl_3_ (0, 2, 4, 6, 8, 10 eq.).

Emission intensity/ A.U.

| wavelenth/ nm | 0 eq. | 2 eq. | 4 eq. | 6 eq. | 8 eq. | 10 eq. |
| --- | --- | --- | --- | --- | --- | --- |
| 430 | 1.539 | 1.405 | 3.282 | 5.562 | 0.040999 | 5.238 |
| 430.5 | 2.96377 | 2.89803 | 4.78913 | 7.0121 | 0.269483 | 6.29358 |
| 431 | 4.70435 | 4.59032 | 6.30087 | 8.20381 | 0.476161 | 7.45048 |
| 431.5 | 6.47913 | 6.32052 | 7.67293 | 9.57597 | 0.824806 | 8.47252 |
| 432 | 7.91042 | 8.14706 | 8.65906 | 10.6894 | 0.18758 | 9.3879 |
| 432.5 | 9.74855 | 9.71719 | 10.2833 | 12.0538 | 0.16787 | 10.1513 |
| 433 | 11.9163 | 11.5019 | 11.6705 | 13.2875 | 0.433548 | 11.1386 |
| 433.5 | 14.0014 | 13.6664 | 13.1935 | 14.6833 | 0.655903 | 12.3555 |
| 434 | 16.1747 | 15.4829 | 14.374 | 15.8316 | 0.574774 | 13.1992 |
| 434.5 | 18.4204 | 17.1615 | 15.3472 | 17.3425 | 0.59087 | 13.9386 |
| 435 | 20.4901 | 18.961 | 16.4401 | 18.5614 | 0.795838 | 15.2894 |
| 435.5 | 22.2579 | 20.6199 | 17.8833 | 19.7402 | 2.15981 | 16.6463 |
| 436 | 24.0391 | 22.5676 | 19.6742 | 21.237 | 3.42284 | 17.8086 |
| 436.5 | 25.9064 | 24.4498 | 21.267 | 22.7765 | 4.94026 | 18.7563 |
| 437 | 28.0189 | 26.4429 | 22.3396 | 24.0304 | 6.03165 | 19.7967 |
| 437.5 | 29.7903 | 28.2784 | 23.8032 | 25.2578 | 7.49868 | 21.0173 |
| 438 | 31.2046 | 29.7288 | 25.1131 | 26.0959 | 8.82097 | 22.609 |
| 438.5 | 32.492 | 31.2454 | 26.2606 | 27.0528 | 9.75145 | 23.8433 |
| 439 | 34.0896 | 32.6674 | 27.6977 | 28.1847 | 11.4458 | 25.0465 |
| 439.5 | 35.3475 | 33.8794 | 28.9184 | 29.4877 | 12.7831 | 26.3116 |
| 440 | 36.9694 | 35.2482 | 29.5926 | 30.7034 | 13.6334 | 27.2769 |
| 440.5 | 39.1625 | 36.4604 | 31.0121 | 32.0845 | 14.7233 | 28.6525 |
| 441 | 40.7876 | 37.8054 | 32.3533 | 33.666 | 16.2411 | 29.9283 |
| 441.5 | 42.3283 | 39.3977 | 34.0837 | 34.7976 | 17.806 | 31.3594 |
| 442 | 44.1747 | 41.0539 | 36.0673 | 36.2902 | 19.2427 | 32.1098 |
| 442.5 | 46.3783 | 42.2976 | 37.7537 | 37.8518 | 20.216 | 33.3382 |
| 443 | 48.4251 | 44.0937 | 39.0499 | 39.6491 | 21.2696 | 34.7824 |
| 443.5 | 50.2064 | 45.8385 | 40.4155 | 40.9932 | 23.1614 | 36.0088 |
| 444 | 52.2068 | 47.2555 | 42.21 | 42.4777 | 24.7401 | 37.4251 |
| 444.5 | 53.9537 | 48.6481 | 43.7337 | 43.9846 | 26.716 | 39.2378 |
| 445 | 55.806 | 50.6375 | 45.1853 | 45.7822 | 29.1684 | 40.6687 |
| 445.5 | 57.7265 | 52.2403 | 47.6151 | 47.657 | 30.9897 | 42.0612 |
| 446 | 59.6086 | 54.3603 | 49.0208 | 49.6663 | 32.8131 | 43.5158 |
| 446.5 | 62.0253 | 55.87 | 51.3031 | 51.6793 | 34.8551 | 44.7081 |
| 447 | 64.3514 | 58.1492 | 53.4648 | 53.4227 | 36.4241 | 46.4015 |
| 447.5 | 66.7749 | 60.7169 | 55.3952 | 56.0861 | 38.0496 | 48.1604 |
| 448 | 69.0498 | 63.2806 | 57.0456 | 58.6203 | 40.3293 | 49.879 |
| 448.5 | 71.4567 | 65.7665 | 59.1825 | 60.5485 | 42.0045 | 51.6053 |
| 449 | 73.6667 | 68.0112 | 61.4246 | 62.5333 | 43.7765 | 53.4147 |
| 449.5 | 75.7008 | 70.4272 | 63.6683 | 64.6011 | 45.8851 | 55.2336 |
| 450 | 78.0847 | 72.4376 | 65.8378 | 66.6384 | 48.4093 | 57.5448 |
| 450.5 | 80.9394 | 74.8001 | 67.9773 | 69.2376 | 50.6355 | 59.6369 |
| 451 | 83.2451 | 77.2546 | 70.3324 | 71.7262 | 53.1926 | 60.9708 |
| 451.5 | 85.4838 | 79.5716 | 72.865 | 73.7286 | 55.5689 | 62.463 |
| 452 | 88.973 | 82.321 | 75.0838 | 75.4147 | 57.6408 | 65.0002 |
| 452.5 | 91.914 | 84.7931 | 77.3066 | 77.6146 | 59.6863 | 67.1171 |
| 453 | 94.9038 | 87.2 | 79.4272 | 80.3436 | 62.2168 | 69.2556 |
| 453.5 | 97.8879 | 89.6921 | 81.6841 | 82.9787 | 64.4604 | 71.4525 |
| 454 | 101.113 | 91.8603 | 84.1879 | 85.1682 | 67.2229 | 73.8798 |
| 454.5 | 103.623 | 94.5621 | 86.6382 | 87.5565 | 69.3601 | 76.2779 |
| 455 | 106.491 | 97.498 | 89.0139 | 90.599 | 71.7881 | 78.4049 |
| 455.5 | 109.645 | 100.161 | 92.1788 | 92.8219 | 73.7504 | 81.0332 |
| 456 | 112.102 | 103.274 | 94.7984 | 94.9282 | 75.6108 | 83.4081 |
| 456.5 | 115.273 | 105.676 | 97.5401 | 97.0377 | 78.2472 | 85.3254 |
| 457 | 118.5 | 108.577 | 100.35 | 100.055 | 80.4766 | 88.2745 |
| 457.5 | 121.258 | 111.147 | 102.948 | 103.108 | 83.1265 | 91.8918 |
| 458 | 123.898 | 114.589 | 105.534 | 105.767 | 85.6041 | 94.3415 |
| 458.5 | 126.548 | 117.25 | 107.917 | 107.827 | 88.6235 | 96.349 |
| 459 | 129.189 | 120.18 | 110.559 | 110.682 | 91.0745 | 98.4269 |
| 459.5 | 132.156 | 123.447 | 113.759 | 114.451 | 93.8479 | 100.696 |
| 460 | 134.887 | 126.532 | 116.676 | 117.884 | 95.7111 | 102.77 |
| 460.5 | 137.628 | 128.925 | 118.937 | 121.137 | 97.365 | 105.683 |
| 461 | 140.929 | 131.899 | 120.876 | 123.645 | 100.526 | 108.258 |
| 461.5 | 143.668 | 135.209 | 123.581 | 126.518 | 103.419 | 110.718 |
| 462 | 146.758 | 138.731 | 126.584 | 129.39 | 105.504 | 113.418 |
| 462.5 | 150.358 | 141.836 | 129.379 | 131.668 | 107.949 | 115.891 |
| 463 | 154.311 | 144.413 | 132.151 | 133.578 | 111.254 | 118.972 |
| 463.5 | 157.702 | 146.529 | 135.079 | 135.695 | 114.201 | 121.801 |
| 464 | 161.217 | 149.023 | 138.378 | 138.924 | 117.362 | 125.065 |
| 464.5 | 165.093 | 152.133 | 140.84 | 141.813 | 120.811 | 128.208 |
| 465 | 168.838 | 154.753 | 144.89 | 144.29 | 123.878 | 131.355 |
| 465.5 | 172.62 | 157.198 | 148.799 | 147.226 | 126.658 | 133.404 |
| 466 | 175.464 | 159.929 | 152.07 | 149.633 | 129.2 | 135.584 |
| 466.5 | 179.859 | 163.474 | 155.617 | 152.45 | 131.853 | 138.152 |
| 467 | 183.923 | 166.611 | 158.012 | 156.388 | 134.704 | 140.719 |
| 467.5 | 186.704 | 169.07 | 160.883 | 159.619 | 137.685 | 142.809 |
| 468 | 190.292 | 172.195 | 165.156 | 162.512 | 140.603 | 145.376 |
| 468.5 | 194.357 | 174.605 | 169.624 | 166.146 | 143.264 | 148.236 |
| 469 | 198.16 | 177.691 | 172.91 | 170.193 | 146.396 | 150.13 |
| 469.5 | 199.984 | 181.173 | 176.186 | 174.085 | 148.932 | 151.719 |
| 470 | 203.291 | 184.467 | 179.302 | 177.153 | 151.563 | 154.459 |
| 470.5 | 207.43 | 187.47 | 182.611 | 179.446 | 154.443 | 157.752 |
| 471 | 211.181 | 190.39 | 185.172 | 183.126 | 157.708 | 160.541 |
| 471.5 | 214.365 | 192.633 | 188.241 | 186.513 | 161.054 | 162.506 |
| 472 | 217.121 | 195.462 | 191.512 | 189.107 | 163.525 | 164.394 |
| 472.5 | 219.743 | 199.015 | 194.224 | 191.547 | 166.387 | 166.282 |
| 473 | 223.218 | 202.065 | 197.326 | 194.277 | 169.701 | 167.718 |
| 473.5 | 227.402 | 205.151 | 199.778 | 197.059 | 173.756 | 169.585 |
| 474 | 230.812 | 208.684 | 202.38 | 200.221 | 176.909 | 171.779 |
| 474.5 | 234.835 | 212.226 | 205.628 | 202.818 | 179.762 | 174.394 |
| 475 | 237.508 | 214.784 | 207.864 | 204.328 | 182.604 | 176.358 |
| 475.5 | 241.497 | 218.569 | 210.567 | 206.337 | 185.454 | 178.995 |
| 476 | 245.171 | 223.269 | 214.186 | 208.917 | 188.46 | 181.278 |
| 476.5 | 248.893 | 226.396 | 217.5 | 211.512 | 189.924 | 184.273 |
| 477 | 253.721 | 229.576 | 220.653 | 214.655 | 193.021 | 187.436 |
| 477.5 | 257.11 | 232.004 | 222.911 | 217.23 | 196.417 | 190.929 |
| 478 | 259.894 | 234.788 | 225.12 | 220.218 | 199.001 | 193.687 |
| 478.5 | 262.671 | 237.497 | 227.796 | 223.081 | 202.342 | 194.712 |
| 479 | 265.812 | 240.958 | 230.614 | 226.262 | 204.911 | 197.188 |
| 479.5 | 269.073 | 243.498 | 232.181 | 229.327 | 208.388 | 198.202 |
| 480 | 271.634 | 246.592 | 234.724 | 232.27 | 210.922 | 199.418 |
| 480.5 | 274.655 | 250.153 | 235.654 | 235.285 | 213.28 | 201.318 |
| 481 | 277.177 | 254.21 | 237.113 | 237.132 | 215.491 | 203.63 |
| 481.5 | 279.872 | 258.147 | 239.793 | 239.57 | 217.694 | 206.565 |
| 482 | 281.87 | 260.038 | 241.217 | 242.092 | 220.064 | 209.632 |
| 482.5 | 284.505 | 263.194 | 243.821 | 243.869 | 222.686 | 212.778 |
| 483 | 287.449 | 266.887 | 246.355 | 246.372 | 224.764 | 215.75 |
| 483.5 | 289.949 | 268.92 | 248.864 | 249.59 | 227.952 | 217.377 |
| 484 | 292.336 | 272.714 | 250.935 | 250.926 | 230.853 | 219.123 |
| 484.5 | 294.897 | 275.329 | 253.188 | 252.437 | 233.685 | 221.606 |
| 485 | 298.478 | 277.125 | 254.713 | 252.62 | 236.03 | 224.282 |
| 485.5 | 301.775 | 277.922 | 256.528 | 253.801 | 238.899 | 226.291 |
| 486 | 303.83 | 279.871 | 258.724 | 256.365 | 241.372 | 227.778 |
| 486.5 | 306.303 | 282.027 | 262.08 | 257.844 | 243.35 | 229.428 |
| 487 | 309.021 | 284.339 | 264.216 | 260.918 | 245.716 | 231.561 |
| 487.5 | 312.369 | 287.3 | 266.59 | 263.706 | 247.571 | 233.708 |
| 488 | 315.054 | 288.358 | 268.24 | 264.846 | 250.108 | 234.661 |
| 488.5 | 316.124 | 290.626 | 269.726 | 265.433 | 251.835 | 236.662 |
| 489 | 317.659 | 292.299 | 270.73 | 266.871 | 253.328 | 238.346 |
| 489.5 | 318.871 | 293.576 | 272.953 | 268.163 | 255.095 | 239.891 |
| 490 | 320.394 | 295.338 | 274.13 | 269.921 | 256.528 | 241.108 |
| 490.5 | 322.364 | 297.257 | 276.117 | 272.585 | 258.064 | 242.991 |
| 491 | 323.637 | 297.009 | 277.073 | 274.348 | 260.124 | 244.269 |
| 491.5 | 326.186 | 297.212 | 278.71 | 276.124 | 261.254 | 246.097 |
| 492 | 326.955 | 298.259 | 280.17 | 278.204 | 263.317 | 247.888 |
| 492.5 | 327.228 | 298.454 | 281.086 | 278.689 | 264.257 | 248.732 |
| 493 | 328.455 | 298.877 | 281.702 | 279.76 | 264.682 | 248.759 |
| 493.5 | 329.931 | 299.432 | 283.053 | 280.844 | 266.731 | 249.698 |
| 494 | 330.246 | 300.415 | 284.158 | 281.814 | 266.826 | 251.241 |
| 494.5 | 331.458 | 302.155 | 283.974 | 282.3 | 267.585 | 251.491 |
| 495 | 331.322 | 304.647 | 285.293 | 282.075 | 267.232 | 252.653 |
| 495.5 | 331.455 | 305.532 | 287.553 | 282.107 | 266.945 | 253.609 |
| 496 | 331.89 | 306.554 | 288.771 | 282.493 | 267.85 | 253.69 |
| 496.5 | 332.455 | 306.673 | 290.242 | 283.101 | 269.036 | 254.492 |
| 497 | 333.778 | 307.053 | 290.868 | 283.872 | 270.276 | 254.25 |
| 497.5 | 334.04 | 308.937 | 291.76 | 284.592 | 270.669 | 253.323 |
| 498 | 333.722 | 309.752 | 292.32 | 285.218 | 269.872 | 252.9 |
| 498.5 | 333.307 | 309.202 | 292.853 | 285.819 | 270.078 | 251.859 |
| 499 | 332.931 | 309.959 | 292.36 | 285.29 | 270.14 | 252.285 |
| 499.5 | 333.288 | 309.708 | 291.598 | 286.56 | 269.527 | 251.9 |
| 500 | 333.415 | 309.653 | 291.755 | 286.242 | 268.686 | 251.506 |
| 500.5 | 333.68 | 310.017 | 292.809 | 287.899 | 268.422 | 251.289 |
| 501 | 333.351 | 311.033 | 293.52 | 287.82 | 267.994 | 250.67 |
| 501.5 | 331.889 | 311.487 | 293.225 | 286.427 | 267.626 | 250.011 |
| 502 | 330.063 | 312.015 | 291.592 | 285.88 | 268.021 | 249.634 |
| 502.5 | 329.474 | 312.095 | 290.835 | 284.64 | 267.515 | 249.9 |
| 503 | 327.942 | 310.21 | 290.079 | 283.679 | 267.346 | 249.589 |
| 503.5 | 327.595 | 309.762 | 290.068 | 283.239 | 266.023 | 250.477 |
| 504 | 328.02 | 309.368 | 289.651 | 283.408 | 265.024 | 249.984 |
| 504.5 | 327.492 | 308.824 | 290.743 | 283.116 | 264.347 | 250.452 |
| 505 | 327.193 | 308.079 | 291.104 | 283.605 | 263.432 | 250.338 |
| 505.5 | 326.744 | 306.858 | 290.677 | 283.23 | 262.511 | 249.889 |
| 506 | 326.824 | 306.416 | 290.383 | 281.589 | 260.983 | 248.984 |
| 506.5 | 326.357 | 306.485 | 290.269 | 280.931 | 259.963 | 248.33 |
| 507 | 325.185 | 306.301 | 289.512 | 279.491 | 259.463 | 246.581 |
| 507.5 | 324.939 | 305.947 | 288.916 | 278.681 | 257.853 | 245.073 |
| 508 | 325.103 | 306.374 | 289.076 | 278.173 | 256.835 | 243.88 |
| 508.5 | 325.341 | 307.391 | 289.365 | 277.083 | 256.577 | 242.546 |
| 509 | 324.162 | 307.074 | 288.872 | 276.242 | 255.054 | 241.391 |
| 509.5 | 322.572 | 306.126 | 288.842 | 275.068 | 253.798 | 240.47 |
| 510 | 321.018 | 304.655 | 289.018 | 274.443 | 251.593 | 239.868 |
| 510.5 | 320.276 | 302.826 | 288.595 | 274.397 | 250.312 | 239.583 |
| 511 | 320.23 | 301.25 | 286.005 | 273.826 | 249.782 | 238.508 |
| 511.5 | 318.371 | 299.282 | 285.148 | 272.641 | 248.625 | 238.619 |
| 512 | 317.057 | 298.479 | 283.929 | 272.799 | 247.17 | 237.607 |
| 512.5 | 314.773 | 296.512 | 282.161 | 271.735 | 245.658 | 236.676 |
| 513 | 313.56 | 295.148 | 280.342 | 269.936 | 245.074 | 236.221 |
| 513.5 | 312.782 | 292.859 | 278.482 | 268.426 | 244.985 | 234.975 |
| 514 | 311.495 | 291.248 | 276.59 | 265.819 | 243.873 | 233.17 |
| 514.5 | 310.312 | 289.342 | 274.914 | 264.583 | 241.608 | 231.976 |
| 515 | 308.218 | 288.129 | 273.183 | 262.851 | 239.974 | 231.314 |
| 515.5 | 306.653 | 286.918 | 272.149 | 260.832 | 238.947 | 229.726 |
| 516 | 303.938 | 285.104 | 270.675 | 258.505 | 237.665 | 228.351 |
| 516.5 | 302.405 | 284.143 | 268.781 | 258.21 | 235.99 | 227.385 |
| 517 | 302.199 | 282.842 | 266.818 | 257.59 | 235.12 | 226.174 |
| 517.5 | 301.064 | 281.662 | 265.3 | 256.659 | 233.221 | 224.899 |
| 518 | 299.154 | 280.315 | 263.855 | 254.941 | 230.859 | 223.186 |
| 518.5 | 297.059 | 279.611 | 261.805 | 253.401 | 228.565 | 221.924 |
| 519 | 295.141 | 278.283 | 260.189 | 253.112 | 227.239 | 220.236 |
| 519.5 | 293.454 | 275.631 | 258.355 | 251.829 | 225.739 | 219.499 |
| 520 | 290.411 | 273.667 | 256.418 | 250.098 | 223.677 | 217.711 |
| 520.5 | 288.79 | 272.065 | 253.798 | 248.233 | 221.601 | 216.962 |
| 521 | 286.406 | 270.275 | 251.875 | 246.539 | 220.187 | 216.33 |
| 521.5 | 283.855 | 268.001 | 249.402 | 245.615 | 218.606 | 214.709 |
| 522 | 280.833 | 266.099 | 247.634 | 244.091 | 216.893 | 213.052 |
| 522.5 | 278.226 | 263.595 | 245.229 | 242.826 | 214.395 | 212.199 |
| 523 | 275.65 | 261.531 | 242.976 | 240.72 | 212.854 | 210.793 |
| 523.5 | 272.278 | 258.774 | 241.085 | 237.897 | 210.929 | 209.525 |
| 524 | 268.323 | 256.087 | 238.432 | 236.006 | 208.08 | 208.704 |
| 524.5 | 265.758 | 253.726 | 235.496 | 234.7 | 205.857 | 207.577 |
| 525 | 264.727 | 251.266 | 232.097 | 233.263 | 204.311 | 206.423 |
| 525.5 | 263.407 | 248.986 | 229.97 | 230.57 | 203.044 | 204.263 |
| 526 | 261.386 | 246.328 | 227.119 | 227.074 | 201.192 | 202.552 |
| 526.5 | 257.522 | 243.739 | 224.783 | 224.291 | 199.557 | 201.121 |
| 527 | 256.244 | 242.752 | 222.612 | 222.681 | 197.183 | 198.323 |
| 527.5 | 254.129 | 240.351 | 219.856 | 219.984 | 195.54 | 195.69 |
| 528 | 251.746 | 238.151 | 217.79 | 217.802 | 192.637 | 193.518 |
| 528.5 | 249.17 | 234.53 | 216.038 | 215.914 | 189.543 | 191.766 |
| 529 | 246.053 | 232.546 | 214.056 | 213.914 | 187.448 | 189.904 |
| 529.5 | 243.728 | 229.472 | 212.64 | 212.208 | 184.91 | 189.354 |

| [a-terp]/ M | I0/I |
| --- | --- |
| 0 | 0.999998 |
| 1e-06 | 1.07175 |
| 2e-06 | 1.1357 |
| 3e-06 | 1.15819 |
| 4e-06 | 1.24387 |
| 5e-06 | 1.32984 |

**Figure S6.** Quenching curves (top) and Stern Volmer plots (bottom) of **1B2C-OH** versus α-terpinene in CHCl_3_ (0, 2, 4, 6, 8, 10 eq.).

Emission intensity/ /counts

| wavelength/ nm | 0 eq. | 2 eq. | 4 eq. | 6 eq. | 8 eq. | 10 eq. |
| --- | --- | --- | --- | --- | --- | --- |
| 450 | 0 | 1.37 | -0.347999 | -1.194 | 0 | -0.333999 |
| 450.5 | 0.046419 | 1.30197 | -0.35229 | -1.05606 | -0.010548 | -0.206548 |
| 451 | 0.112387 | 1.29474 | -0.192387 | -0.963774 | 0.067129 | -0.042645 |
| 451.5 | 0.191741 | 1.25364 | -0.022193 | -0.867677 | 0.088193 | -0.167999 |
| 452 | 0.304193 | 1.29416 | 0.033612 | -0.672838 | 0.151064 | -0.094645 |
| 452.5 | 0.36 | 1.45571 | 0.082935 | -0.360903 | 0.165258 | 0.049806 |
| 453 | 0.419225 | 1.36735 | 0.145354 | -0.230806 | 0.221677 | 0.126387 |
| 453.5 | 0.518483 | 1.30939 | 0.248064 | -0.104354 | 0.29187 | 0.22529 |
| 454 | 0.752741 | 1.24465 | 0.300741 | 0.029516 | 0.502709 | 0.337225 |
| 454.5 | 0.874741 | 1.2711 | 0.336483 | 0.205419 | 0.717838 | 0.513612 |
| 455 | 0.943741 | 1.27761 | 0.522419 | 0.274612 | 0.851741 | 0.602483 |
| 455.5 | 1.08126 | 1.28419 | 0.695612 | 0.40487 | 0.915548 | 0.781064 |
| 456 | 1.13384 | 1.31061 | 0.764935 | 0.508612 | 0.957903 | 0.889967 |
| 456.5 | 1.1959 | 1.28961 | 0.820774 | 0.654354 | 0.972064 | 1.10271 |
| 457 | 1.21561 | 1.42142 | 0.969967 | 0.678354 | 0.983354 | 1.20816 |
| 457.5 | 1.30068 | 1.57055 | 1.22616 | 0.969129 | 1.03245 | 1.33935 |
| 458 | 1.43484 | 1.61058 | 1.2641 | 0.962032 | 1.05329 | 1.54352 |
| 458.5 | 1.48777 | 1.641 | 1.24387 | 0.987322 | 1.26687 | 1.793 |
| 459 | 1.65942 | 1.73245 | 1.29761 | 1.21542 | 1.36477 | 1.85442 |
| 459.5 | 1.76426 | 1.84732 | 1.42884 | 1.23452 | 1.50422 | 1.86071 |
| 460 | 1.98745 | 1.93813 | 1.53968 | 1.34887 | 1.63813 | 1.95352 |
| 460.5 | 2.08632 | 2.01277 | 1.57306 | 1.54648 | 1.74313 | 2.0431 |
| 461 | 2.29026 | 2.1791 | 1.44639 | 1.75926 | 1.84177 | 2.13019 |
| 461.5 | 2.41603 | 2.33494 | 1.48664 | 1.92274 | 1.89481 | 2.08835 |
| 462 | 2.66632 | 2.52797 | 1.55042 | 2.13268 | 1.93739 | 2.2309 |
| 462.5 | 2.92322 | 2.81677 | 1.64435 | 2.32013 | 2.2261 | 2.46355 |
| 463 | 3.09226 | 3.10248 | 1.91006 | 2.42248 | 2.51039 | 2.61052 |
| 463.5 | 3.28968 | 3.36984 | 2.15768 | 2.57745 | 2.66545 | 2.8169 |
| 464 | 3.53858 | 3.60861 | 2.41481 | 2.80545 | 2.79939 | 3.0419 |
| 464.5 | 3.75287 | 3.80558 | 2.71974 | 2.988 | 2.939 | 3.5329 |
| 465 | 4.05319 | 4.06281 | 2.88445 | 3.262 | 3.20381 | 3.58352 |
| 465.5 | 4.37394 | 4.39787 | 3.28213 | 3.36268 | 3.43335 | 3.79532 |
| 466 | 4.50442 | 4.7281 | 3.81861 | 3.34181 | 3.71897 | 4.08103 |
| 466.5 | 4.81184 | 5.12658 | 4.12271 | 3.59261 | 3.95645 | 4.36135 |
| 467 | 5.00339 | 5.62755 | 4.30419 | 3.67626 | 4.23258 | 4.65661 |
| 467.5 | 5.13245 | 6.03206 | 4.49261 | 3.9579 | 4.60448 | 4.89026 |
| 468 | 5.60719 | 6.22677 | 4.79632 | 4.34568 | 5.14381 | 5.10826 |
| 468.5 | 6.11887 | 6.71135 | 5.25358 | 4.85623 | 5.51571 | 5.4299 |
| 469 | 6.5009 | 7.19948 | 5.80148 | 5.08045 | 5.91977 | 5.77058 |
| 469.5 | 6.894 | 7.78539 | 6.25987 | 5.18668 | 6.13742 | 6.0091 |
| 470 | 7.55203 | 8.25471 | 6.56016 | 5.51994 | 6.2859 | 6.50629 |
| 470.5 | 8.43403 | 8.63774 | 7.16058 | 5.95693 | 6.67587 | 6.84342 |
| 471 | 9.13626 | 9.03061 | 7.64261 | 6.36674 | 7.29426 | 7.3531 |
| 471.5 | 9.83735 | 9.60393 | 8.22439 | 6.99535 | 7.70142 | 7.83674 |
| 472 | 10.5929 | 10.3394 | 8.689 | 7.62971 | 8.30968 | 8.38074 |
| 472.5 | 11.5099 | 10.8878 | 9.07261 | 7.99203 | 8.82852 | 8.95068 |
| 473 | 12.3539 | 11.2533 | 9.731 | 8.66564 | 9.39329 | 9.66442 |
| 473.5 | 12.9704 | 11.7634 | 10.3788 | 9.31061 | 9.992 | 10.2982 |
| 474 | 13.7018 | 12.5588 | 11.0755 | 9.79419 | 10.5905 | 10.6246 |
| 474.5 | 14.5285 | 13.2543 | 11.7422 | 10.3115 | 11.1565 | 11.298 |
| 475 | 15.3859 | 14.3087 | 12.2092 | 11.0949 | 11.8704 | 12.146 |
| 475.5 | 15.9507 | 14.9801 | 12.9316 | 11.7023 | 12.5581 | 12.7178 |
| 476 | 16.7735 | 15.7115 | 13.6916 | 12.2783 | 13.2677 | 13.2666 |
| 476.5 | 17.5897 | 16.4081 | 14.581 | 12.9739 | 14.1054 | 13.9531 |
| 477 | 18.2882 | 17.161 | 15.6448 | 13.4849 | 15.0432 | 14.8509 |
| 477.5 | 18.7522 | 17.8785 | 16.6104 | 14.2196 | 15.5711 | 15.4629 |
| 478 | 19.769 | 18.6533 | 17.5884 | 14.8867 | 16.2943 | 16.1706 |
| 478.5 | 21.0552 | 19.7477 | 18.4187 | 15.9365 | 16.9743 | 16.9331 |
| 479 | 22.1327 | 20.4732 | 19.0936 | 17.0153 | 17.981 | 17.5983 |
| 479.5 | 23.646 | 21.3023 | 19.9523 | 18.4034 | 18.9058 | 18.5007 |
| 480 | 24.8306 | 22.4736 | 20.8828 | 19.7499 | 19.7943 | 19.4882 |
| 480.5 | 25.9535 | 23.7049 | 21.7505 | 20.823 | 20.9277 | 20.357 |
| 481 | 27.1882 | 24.8638 | 22.6991 | 21.8525 | 22.2383 | 21.1405 |
| 481.5 | 28.4941 | 25.7783 | 23.7464 | 23.2443 | 23.5815 | 22.2232 |
| 482 | 29.9252 | 26.5539 | 24.7608 | 24.5525 | 24.687 | 23.3189 |
| 482.5 | 31.7266 | 27.4508 | 26.1638 | 25.7808 | 26.0863 | 24.2404 |
| 483 | 33.2943 | 28.7196 | 27.897 | 26.874 | 27.2656 | 25.2564 |
| 483.5 | 34.6015 | 30.002 | 29.3491 | 28.0927 | 28.1882 | 26.7435 |
| 484 | 36.016 | 31.2945 | 30.4935 | 29.319 | 29.2603 | 27.8287 |
| 484.5 | 37.9242 | 32.1961 | 31.7482 | 30.7237 | 30.4827 | 29.1598 |
| 485 | 39.0529 | 33.4585 | 33.1286 | 32.2581 | 31.9063 | 30.7112 |
| 485.5 | 40.8515 | 34.8115 | 34.6698 | 33.6889 | 33.1658 | 31.9789 |
| 486 | 42.4421 | 36.4154 | 36.027 | 35.2545 | 34.4127 | 33.1346 |
| 486.5 | 43.7089 | 38.1705 | 37.4639 | 36.9313 | 35.9712 | 34.3634 |
| 487 | 45.3922 | 40.0959 | 38.7483 | 38.3173 | 37.6258 | 35.6412 |
| 487.5 | 46.7286 | 41.6589 | 40.3989 | 39.5427 | 38.7347 | 36.9495 |
| 488 | 48.025 | 43.0298 | 42.0338 | 41.0598 | 40.2368 | 38.1014 |
| 488.5 | 49.5077 | 44.9187 | 43.7957 | 42.4966 | 42.0269 | 39.2184 |
| 489 | 51.1577 | 47.0225 | 45.2821 | 44.3531 | 43.8672 | 40.6004 |
| 489.5 | 52.9349 | 48.8364 | 46.7913 | 46.0362 | 45.4502 | 42.0013 |
| 490 | 55.3135 | 50.2941 | 48.6374 | 47.8198 | 47.1176 | 43.6802 |
| 490.5 | 57.5113 | 51.4123 | 50.5094 | 49.7627 | 48.8606 | 45.2083 |
| 491 | 59.5979 | 53.3563 | 52.4349 | 51.8029 | 50.4316 | 47.0014 |
| 491.5 | 61.4778 | 55.2748 | 54.3858 | 53.9829 | 52.5953 | 48.8144 |
| 492 | 63.5559 | 57.4527 | 56.1324 | 55.8111 | 54.2352 | 50.2755 |
| 492.5 | 65.84 | 59.3114 | 58.1578 | 57.5868 | 55.9375 | 51.9107 |
| 493 | 68.3881 | 61.7584 | 59.7316 | 59.2705 | 58.0088 | 53.6441 |
| 493.5 | 70.3822 | 63.3814 | 61.7698 | 61.5016 | 59.6873 | 55.4268 |
| 494 | 72.2194 | 65.2412 | 63.7902 | 63.4824 | 61.2462 | 57.1534 |
| 494.5 | 74.3643 | 67.7382 | 65.922 | 65.1038 | 62.9303 | 58.8557 |
| 495 | 76.1086 | 69.782 | 68.0714 | 66.6379 | 64.6274 | 60.5458 |
| 495.5 | 78.417 | 71.7895 | 70.3272 | 68.6906 | 66.3216 | 62.0899 |
| 496 | 81.0008 | 73.8165 | 72.7684 | 70.2837 | 67.4525 | 63.994 |
| 496.5 | 83.3647 | 76.143 | 74.6679 | 72.234 | 69.0325 | 65.7827 |
| 497 | 85.8646 | 78.7382 | 77.188 | 74.1619 | 70.4771 | 67.3706 |
| 497.5 | 87.4855 | 81.4006 | 79.587 | 75.6357 | 71.9279 | 69.5481 |
| 498 | 89.2155 | 84.2942 | 81.8855 | 78.1888 | 73.3207 | 71.6751 |
| 498.5 | 91.6531 | 86.2013 | 83.8402 | 80.4223 | 75.3269 | 73.7443 |
| 499 | 93.7999 | 88.4363 | 86.1776 | 82.3253 | 77.4698 | 74.7618 |
| 499.5 | 95.7861 | 90.73 | 88.9855 | 84.5198 | 79.4432 | 76.3698 |
| 500 | 97.7499 | 93.6122 | 91.0051 | 86.3145 | 81.7029 | 77.7702 |
| 500.5 | 100.348 | 95.9394 | 93.1539 | 88.1565 | 83.9219 | 79.1911 |
| 501 | 102.493 | 98.4939 | 95.6335 | 90.2262 | 86.3845 | 80.7331 |
| 501.5 | 104.747 | 100.292 | 98.3884 | 92.2628 | 88.5813 | 82.244 |
| 502 | 108.214 | 102.696 | 100.66 | 94.2466 | 90.639 | 83.8444 |
| 502.5 | 111.237 | 104.657 | 102.772 | 96.1346 | 92.4211 | 86.266 |
| 503 | 114.13 | 106.834 | 104.905 | 98.8712 | 94.4856 | 87.4962 |
| 503.5 | 117.337 | 109.461 | 107.539 | 101.285 | 96.594 | 89.0972 |
| 504 | 120.256 | 111.688 | 109.495 | 104 | 98.7183 | 91.1124 |
| 504.5 | 123.193 | 114.297 | 112.36 | 106.898 | 100.161 | 92.7808 |
| 505 | 125.936 | 116.299 | 115.048 | 108.862 | 102.213 | 94.7061 |
| 505.5 | 127.845 | 118.456 | 117.05 | 111.321 | 104.231 | 96.1074 |
| 506 | 129.997 | 120.389 | 119.512 | 112.887 | 105.866 | 98.334 |
| 506.5 | 132.854 | 122.483 | 122.293 | 114.653 | 108.018 | 99.8012 |
| 507 | 135.817 | 125.118 | 124.411 | 116.542 | 109.271 | 101.609 |
| 507.5 | 138.616 | 127.389 | 126.805 | 119.207 | 111.061 | 103.145 |
| 508 | 141.455 | 129.84 | 128.75 | 121.72 | 112.839 | 104.596 |
| 508.5 | 143.508 | 131.875 | 131.324 | 124.812 | 114.609 | 106.239 |
| 509 | 146.316 | 134.448 | 132.737 | 126.634 | 117.417 | 108.009 |
| 509.5 | 149.344 | 136.082 | 134.826 | 128.1 | 119.804 | 109.715 |
| 510 | 151.917 | 137.698 | 137.048 | 130.015 | 121.787 | 111.778 |
| 510.5 | 153.846 | 140.517 | 138.946 | 131.706 | 123.982 | 113.388 |
| 511 | 155.925 | 142.537 | 141.013 | 133.019 | 126.286 | 115.07 |
| 511.5 | 157.791 | 144.335 | 142.436 | 135.111 | 129.418 | 117.024 |
| 512 | 159.639 | 146.093 | 144.497 | 137.239 | 130.982 | 118.467 |
| 512.5 | 162.166 | 148.501 | 145.765 | 139.81 | 132.5 | 120.247 |
| 513 | 165.483 | 150.318 | 147.573 | 141.837 | 134.717 | 121.783 |
| 513.5 | 168.091 | 151.799 | 149.503 | 143.349 | 137.143 | 123.428 |
| 514 | 170.103 | 154.218 | 151.485 | 145.664 | 138.958 | 125.147 |
| 514.5 | 172.91 | 156.728 | 153.036 | 147.526 | 140.841 | 127.273 |
| 515 | 174.956 | 158.357 | 154.115 | 149.066 | 143.272 | 129.094 |
| 515.5 | 177.094 | 160.031 | 156.177 | 150.953 | 144.755 | 131.268 |
| 516 | 179.849 | 162.28 | 158.795 | 152.819 | 145.908 | 133.184 |
| 516.5 | 182.04 | 164.011 | 160.534 | 153.967 | 147.669 | 135.179 |
| 517 | 184.497 | 166.123 | 162.679 | 155.783 | 149.422 | 136.799 |
| 517.5 | 185.452 | 167.797 | 164.544 | 157.344 | 150.603 | 138.183 |
| 518 | 187.154 | 169.569 | 165.832 | 159.216 | 152.234 | 138.535 |
| 518.5 | 188.543 | 171.31 | 167.055 | 160.026 | 153.904 | 140.208 |
| 519 | 189.663 | 172.646 | 168.217 | 161.572 | 154.979 | 141.398 |
| 519.5 | 191.083 | 173.912 | 169.545 | 162.866 | 155.85 | 142.23 |
| 520 | 193.08 | 175.122 | 170.992 | 163.384 | 157.99 | 143.407 |
| 520.5 | 194.917 | 177.274 | 172.949 | 164.377 | 159.262 | 144.915 |
| 521 | 196.187 | 179.345 | 174.417 | 165.111 | 161.1 | 146.551 |
| 521.5 | 197.445 | 182.333 | 175.883 | 166.584 | 162.869 | 147.326 |
| 522 | 199.052 | 183.776 | 177.024 | 167.771 | 163.819 | 148.743 |
| 522.5 | 200.432 | 184.647 | 178.193 | 168.688 | 164.719 | 149.899 |
| 523 | 200.992 | 185.902 | 179.582 | 168.863 | 166.234 | 151.74 |
| 523.5 | 202.086 | 187.548 | 180.58 | 169.628 | 167.814 | 153.407 |
| 524 | 204.445 | 188.586 | 181.976 | 169.918 | 168.483 | 154.637 |
| 524.5 | 205.35 | 188.987 | 183.744 | 171.702 | 168.349 | 155.689 |
| 525 | 206.262 | 190.073 | 185.054 | 173.337 | 168.927 | 157.042 |
| 525.5 | 207.533 | 191.346 | 186.446 | 174.505 | 169.64 | 158.001 |
| 526 | 209.561 | 191.835 | 187.896 | 175.517 | 169.84 | 159.036 |
| 526.5 | 211.048 | 192.677 | 188.887 | 177.053 | 170.272 | 160.049 |
| 527 | 212.137 | 194.094 | 190.37 | 178.703 | 170.196 | 160.748 |
| 527.5 | 213.468 | 195.343 | 191.24 | 179.236 | 171.915 | 161.551 |
| 528 | 213.67 | 195.733 | 192.283 | 179.099 | 173.115 | 162.06 |
| 528.5 | 213.929 | 196.75 | 193.265 | 179.849 | 173.499 | 162.738 |
| 529 | 214.584 | 197.339 | 193.575 | 180.609 | 174.135 | 163.522 |
| 529.5 | 215.726 | 197.38 | 193.774 | 180.747 | 174.639 | 163.595 |
| 530 | 215.613 | 197.283 | 193.951 | 181.046 | 175.024 | 163.847 |
| 530.5 | 216.158 | 197.516 | 194.477 | 181.938 | 175.009 | 164.412 |
| 531 | 216.542 | 197.741 | 194.983 | 182.312 | 175.506 | 164.652 |
| 531.5 | 216.08 | 197.814 | 194.596 | 183.051 | 175.919 | 164.648 |
| 532 | 215.414 | 197.662 | 194.509 | 184.038 | 175.715 | 164.837 |
| 532.5 | 214.932 | 197.94 | 193.839 | 184.195 | 175.835 | 165.644 |
| 533 | 214.844 | 198.342 | 193.889 | 184.167 | 175.766 | 166.311 |
| 533.5 | 214.653 | 198.778 | 194.63 | 183.79 | 175.514 | 167.078 |
| 534 | 214.383 | 198.948 | 194.889 | 184.629 | 175.329 | 167.427 |
| 534.5 | 214.755 | 199.41 | 195.004 | 184.94 | 176.144 | 168.047 |
| 535 | 214.667 | 199.576 | 194.921 | 185.299 | 176.756 | 167.909 |
| 535.5 | 213.928 | 199.61 | 194.213 | 185.129 | 176.448 | 167.739 |
| 536 | 213.152 | 199.576 | 193.476 | 185.404 | 176.053 | 166.978 |
| 536.5 | 213.022 | 199.267 | 193.496 | 185.115 | 175.333 | 165.986 |
| 537 | 212.842 | 197.716 | 192.604 | 184.98 | 174.946 | 165.419 |
| 537.5 | 212.153 | 197.513 | 191.762 | 184.379 | 174.666 | 165.011 |
| 538 | 211.351 | 197.124 | 191.282 | 184.497 | 174.597 | 164.343 |
| 538.5 | 211.384 | 195.97 | 190.421 | 184.299 | 174.144 | 163.944 |
| 539 | 210.757 | 194.576 | 189.783 | 184.098 | 173.734 | 163.404 |
| 539.5 | 209.197 | 193.624 | 188.947 | 183.074 | 174.301 | 163.068 |
| 540 | 208.411 | 193.711 | 188.088 | 181.907 | 174.297 | 162.675 |
| 540.5 | 207.567 | 193.425 | 186.969 | 180.906 | 174.179 | 161.642 |
| 541 | 206.261 | 192.151 | 186.162 | 180.066 | 173.611 | 160.731 |
| 541.5 | 204.819 | 191.098 | 185.268 | 179.129 | 173.524 | 160.052 |
| 542 | 204.157 | 189.815 | 184.063 | 178.077 | 173.415 | 158.812 |
| 542.5 | 202.877 | 188.698 | 183.325 | 176.968 | 172.789 | 157.735 |
| 543 | 201.372 | 187.643 | 182.883 | 176.373 | 171.241 | 157.725 |
| 543.5 | 200.615 | 186.458 | 181.862 | 175.608 | 170.565 | 157.557 |
| 544 | 199.538 | 185.327 | 180.362 | 174.962 | 169.296 | 156.08 |
| 544.5 | 198.389 | 184.331 | 179.725 | 173.096 | 167.527 | 154.77 |
| 545 | 196.812 | 183.904 | 179.087 | 171.945 | 166.62 | 154.117 |
| 545.5 | 196.29 | 182.789 | 178.731 | 170.972 | 165.946 | 153.111 |
| 546 | 195.428 | 182.442 | 177.522 | 169.052 | 164.576 | 152.201 |
| 546.5 | 194.245 | 181.838 | 175.627 | 168.233 | 163.068 | 151.393 |
| 547 | 193.694 | 180.573 | 174.568 | 167.132 | 162.145 | 150.338 |
| 547.5 | 192.939 | 179.728 | 173.375 | 166.346 | 161.195 | 149.026 |
| 548 | 191.313 | 178.545 | 172.42 | 165.104 | 160.392 | 148.004 |
| 548.5 | 190.127 | 177.533 | 170.932 | 163.753 | 159.157 | 146.849 |
| 549 | 188.761 | 175.811 | 169.562 | 163.107 | 158.454 | 145.756 |
| 549.5 | 187.972 | 173.721 | 168.544 | 161.235 | 157.776 | 144.299 |

| [a-terp]/ M | I0/I |
| --- | --- |
| 0 | 1 |
| 1e-06 | 1.09508 |
| 2e-06 | 1.11057 |
| 3e-06 | 1.18776 |
| 4e-06 | 1.23382 |
| 5e-06 | 1.31515 |

**Figure S7.** Quenching curves (top) and Stern Volmer plots (bottom) of **1B2C-Pr** versus α-terpinene in CHCl_3_ (0, 2, 4, 6, 8, 10 eq.).

Emission intensity/ /counts

| wavelength/ nm | 0 eq. | 2 eq. | 4 eq. | 6 eq. | 10 eq. |
| --- | --- | --- | --- | --- | --- |
| 450 | -0.779999 | -1.941 | -0.186 | -0.37 | 0 |
| 450.5 | -0.643483 | -1.81203 | -0.051516 | -0.286419 | -0.105354 |
| 451 | -0.334741 | -1.72819 | 0.118419 | -0.266096 | -0.085709 |
| 451.5 | -0.167419 | -1.60306 | 0.277483 | -0.31029 | -0.011709 |
| 452 | -9.6e-05 | -1.38748 | 0.432258 | -0.258064 | 0.085129 |
| 452.5 | 0.133161 | -1.14916 | 0.579483 | -0.187516 | 0.186483 |
| 453 | 0.292483 | -0.978258 | 0.691806 | -0.135161 | 0.149741 |
| 453.5 | 0.387806 | -0.762838 | 0.739612 | -0.046774 | 0.16829 |
| 454 | 0.449354 | -0.558677 | 0.762387 | 0.252516 | 0.279451 |
| 454.5 | 0.733322 | -0.30229 | 0.867225 | 0.412516 | 0.151645 |
| 455 | 0.851612 | 0.078193 | 0.922032 | 0.557451 | 0.25787 |
| 455.5 | 1.00771 | 0.319516 | 0.991032 | 0.760806 | 0.285741 |
| 456 | 1.11771 | 0.568548 | 1.10993 | 0.813387 | 0.442903 |
| 456.5 | 1.29929 | 0.799419 | 1.24022 | 0.930064 | 0.498419 |
| 457 | 1.50806 | 1.1699 | 1.48952 | 1.02881 | 0.562677 |
| 457.5 | 1.70526 | 1.39703 | 1.62071 | 1.27642 | 0.672645 |
| 458 | 1.85432 | 1.55339 | 1.724 | 1.35629 | 0.714258 |
| 458.5 | 2.05265 | 1.61339 | 1.80587 | 1.45584 | 0.889774 |
| 459 | 2.07506 | 1.75255 | 1.82661 | 1.60545 | 0.926516 |
| 459.5 | 2.14287 | 2.08342 | 1.96952 | 1.81213 | 0.917225 |
| 460 | 2.31745 | 2.21942 | 1.96952 | 2.09281 | 0.898677 |
| 460.5 | 2.41581 | 2.57232 | 1.97668 | 2.23142 | 0.981483 |
| 461 | 2.44984 | 2.79268 | 2.02022 | 2.43416 | 1.09081 |
| 461.5 | 2.51784 | 2.87497 | 2.13226 | 2.63232 | 1.39481 |
| 462 | 2.76565 | 3.043 | 2.35171 | 2.67484 | 1.482 |
| 462.5 | 2.96632 | 3.13213 | 2.39465 | 2.696 | 1.72168 |
| 463 | 3.23884 | 3.23742 | 2.58058 | 2.82539 | 1.89216 |
| 463.5 | 3.5551 | 3.42974 | 2.90948 | 3.03784 | 1.99739 |
| 464 | 3.66006 | 3.51903 | 3.15871 | 3.30232 | 2.07103 |
| 464.5 | 3.87084 | 3.71026 | 3.50223 | 3.77677 | 2.1671 |
| 465 | 4.04506 | 3.79245 | 3.63606 | 3.99203 | 2.25813 |
| 465.5 | 4.2781 | 3.96993 | 3.68616 | 4.13503 | 2.30432 |
| 466 | 4.36123 | 4.14619 | 3.85948 | 4.33297 | 2.57587 |
| 466.5 | 4.33587 | 4.51068 | 4.02232 | 4.69903 | 2.9451 |
| 467 | 4.41868 | 4.87664 | 4.19429 | 4.8669 | 3.04616 |
| 467.5 | 4.74665 | 5.1501 | 4.33319 | 5.00945 | 3.20313 |
| 468 | 5.09258 | 5.48306 | 4.53835 | 5.24522 | 3.47068 |
| 468.5 | 5.64648 | 5.83819 | 5.02545 | 5.59158 | 3.82639 |
| 469 | 6.06522 | 6.06439 | 5.40574 | 6.01884 | 3.93664 |
| 469.5 | 6.72739 | 6.37477 | 5.86358 | 6.346 | 4.11555 |
| 470 | 7.18152 | 6.71948 | 6.48945 | 6.63565 | 4.58755 |
| 470.5 | 7.62965 | 7.03174 | 6.9771 | 7.30306 | 4.83281 |
| 471 | 8.07445 | 7.403 | 7.30419 | 7.94274 | 5.13394 |
| 471.5 | 8.60048 | 7.86006 | 7.79426 | 8.44445 | 5.47626 |
| 472 | 9.23016 | 8.40577 | 8.30494 | 9.07242 | 5.99039 |
| 472.5 | 9.745 | 8.88523 | 8.79148 | 9.61374 | 6.4811 |
| 473 | 10.3752 | 9.42297 | 9.31952 | 9.95565 | 6.81352 |
| 473.5 | 10.9781 | 10.0805 | 9.94806 | 10.3081 | 7.39023 |
| 474 | 11.3663 | 10.9395 | 10.4342 | 10.8044 | 7.75723 |
| 474.5 | 11.9019 | 11.5435 | 10.9376 | 11.2971 | 8.3199 |
| 475 | 12.5745 | 11.895 | 11.7183 | 11.6263 | 8.65771 |
| 475.5 | 13.3896 | 12.6604 | 12.4349 | 11.9921 | 9.03113 |
| 476 | 14.265 | 13.3235 | 13.1945 | 12.8663 | 9.42471 |
| 476.5 | 15.1796 | 14.1348 | 13.7096 | 13.6528 | 10.1341 |
| 477 | 15.7991 | 14.9174 | 14.5157 | 14.4517 | 10.69 |
| 477.5 | 16.3999 | 15.7679 | 15.1446 | 15.0449 | 11.4228 |
| 478 | 17.33 | 16.4684 | 15.9382 | 15.8329 | 11.8311 |
| 478.5 | 18.1318 | 17.2841 | 16.6645 | 16.8308 | 12.81 |
| 479 | 19.0497 | 17.9345 | 17.3521 | 17.6045 | 13.6166 |
| 479.5 | 20.2078 | 18.6162 | 18.2064 | 18.2917 | 14.5636 |
| 480 | 21.113 | 19.2998 | 19.2438 | 19.1377 | 15.2872 |
| 480.5 | 21.9718 | 20.1617 | 20.1844 | 20.0628 | 15.8216 |
| 481 | 22.8111 | 21.0642 | 21.2759 | 21.1908 | 16.5254 |
| 481.5 | 24.1553 | 22.1524 | 22.5765 | 22.2912 | 17.134 |
| 482 | 25.3187 | 23.0613 | 23.9877 | 23.4105 | 18.0745 |
| 482.5 | 26.4752 | 24.2688 | 24.9863 | 24.5772 | 19.3755 |
| 483 | 27.6268 | 25.3812 | 25.8728 | 25.5365 | 20.3635 |
| 483.5 | 28.5353 | 26.4426 | 26.801 | 26.7159 | 21.3265 |
| 484 | 29.3933 | 27.7682 | 27.6045 | 27.7677 | 22.3294 |
| 484.5 | 30.5827 | 29.1749 | 28.9137 | 28.6578 | 23.6654 |
| 485 | 31.8255 | 30.5979 | 29.9116 | 29.6113 | 24.7053 |
| 485.5 | 32.9257 | 32.0581 | 30.6206 | 30.6867 | 25.7813 |
| 486 | 34.2069 | 33.8083 | 31.9094 | 31.5317 | 26.964 |
| 486.5 | 35.7606 | 35.5378 | 33.4891 | 32.2762 | 27.9511 |
| 487 | 37.1979 | 36.7231 | 34.8402 | 33.4854 | 29.0905 |
| 487.5 | 38.1203 | 38.284 | 36.1002 | 34.3992 | 29.8417 |
| 488 | 39.7854 | 39.8474 | 37.4193 | 35.988 | 30.706 |
| 488.5 | 41.3588 | 41.7258 | 38.6724 | 37.3335 | 31.7156 |
| 489 | 42.7209 | 43.1435 | 39.7173 | 38.8646 | 32.852 |
| 489.5 | 43.8219 | 44.3339 | 41.7686 | 39.8052 | 33.9116 |
| 490 | 45.4302 | 46.0703 | 43.231 | 40.8991 | 34.9828 |
| 490.5 | 46.7771 | 47.602 | 44.4193 | 42.3701 | 36.6064 |
| 491 | 48.3858 | 49.1367 | 46.1035 | 44.0229 | 38.1222 |
| 491.5 | 50.0188 | 50.3603 | 48.1014 | 45.2599 | 39.6243 |
| 492 | 51.5281 | 51.3545 | 49.833 | 46.4808 | 40.9965 |
| 492.5 | 53.5461 | 53.1546 | 51.345 | 47.6934 | 42.4263 |
| 493 | 55.7176 | 55.183 | 53.6555 | 49.7631 | 43.9324 |
| 493.5 | 57.7552 | 56.9806 | 55.4196 | 51.6025 | 45.4285 |
| 494 | 59.7526 | 58.9007 | 57.1074 | 52.9021 | 46.3692 |
| 494.5 | 61.2943 | 61.2917 | 58.8708 | 54.6051 | 47.4451 |
| 495 | 62.9879 | 63.7328 | 60.683 | 55.9336 | 49.2508 |
| 495.5 | 64.9296 | 66.2283 | 62.0633 | 57.4783 | 50.9319 |
| 496 | 67.3666 | 68.1691 | 64.2412 | 59.022 | 52.4914 |
| 496.5 | 69.7535 | 70.3854 | 66.5453 | 60.4157 | 54.3767 |
| 497 | 71.8496 | 72.457 | 68.0501 | 62.0055 | 55.923 |
| 497.5 | 73.931 | 74.707 | 69.29 | 64.0372 | 57.0627 |
| 498 | 76.5414 | 76.5845 | 71.1849 | 65.9847 | 58.6154 |
| 498.5 | 78.7501 | 78.784 | 73.0763 | 68.0096 | 60.234 |
| 499 | 81.4128 | 80.7326 | 75.1613 | 69.6922 | 61.6264 |
| 499.5 | 83.3404 | 82.697 | 77.7667 | 71.3789 | 63.0745 |
| 500 | 85.6189 | 84.687 | 79.8711 | 73.6565 | 64.0599 |
| 500.5 | 87.8182 | 86.4536 | 82.174 | 75.8534 | 65.9455 |
| 501 | 91.0926 | 88.4343 | 84.9394 | 78.067 | 67.512 |
| 501.5 | 93.4198 | 89.8138 | 87.1419 | 80.1736 | 69.037 |
| 502 | 95.5603 | 91.5272 | 88.8105 | 82.3166 | 70.229 |
| 502.5 | 98.2588 | 94.4159 | 90.7165 | 84.5108 | 72.0074 |
| 503 | 101.869 | 96.4264 | 92.6523 | 86.4641 | 74.8337 |
| 503.5 | 104.077 | 98.8276 | 95.0115 | 88.1002 | 77.7491 |
| 504 | 105.923 | 100.455 | 97.2048 | 89.8848 | 80.3116 |
| 504.5 | 108.15 | 102.284 | 99.5724 | 91.9452 | 82.0178 |
| 505 | 110.94 | 104.179 | 100.969 | 94.4975 | 83.9191 |
| 505.5 | 113.944 | 106.107 | 103.02 | 97.2541 | 85.4968 |
| 506 | 116.227 | 108.794 | 105.084 | 100.025 | 86.6415 |
| 506.5 | 118.406 | 110.983 | 107.307 | 101.933 | 88.1359 |
| 507 | 120.592 | 113.589 | 109.237 | 103.712 | 89.8257 |
| 507.5 | 123.686 | 116.705 | 111.829 | 105.923 | 91.4513 |
| 508 | 126.129 | 118.306 | 114.636 | 108.355 | 91.9784 |
| 508.5 | 128.249 | 120.469 | 116.167 | 110.21 | 93.4818 |
| 509 | 130.343 | 122.469 | 117.754 | 112.06 | 95.6327 |
| 509.5 | 132.489 | 124.684 | 119.581 | 114.469 | 98.0725 |
| 510 | 135.18 | 126.346 | 121.685 | 116.638 | 100.281 |
| 510.5 | 137.902 | 127.823 | 123.666 | 119.169 | 101.595 |
| 511 | 140.605 | 129.169 | 126.592 | 120.814 | 103.341 |
| 511.5 | 143.265 | 131.52 | 128.56 | 122.253 | 105.478 |
| 512 | 145.516 | 133.924 | 130.31 | 124.457 | 107.467 |
| 512.5 | 147.623 | 135.873 | 132.125 | 125.917 | 109.455 |
| 513 | 149.989 | 137.563 | 134.606 | 126.858 | 111.86 |
| 513.5 | 151.604 | 139.843 | 137.084 | 128.083 | 114.057 |
| 514 | 153.933 | 142.53 | 139.438 | 130.339 | 115.804 |
| 514.5 | 155.712 | 145.213 | 142.117 | 132.657 | 117.404 |
| 515 | 157.51 | 147.382 | 144.149 | 134.606 | 119.066 |
| 515.5 | 159.467 | 149.134 | 146.062 | 136.857 | 121.042 |
| 516 | 161.672 | 151.074 | 148.293 | 138.829 | 122.309 |
| 516.5 | 163.017 | 152.87 | 150.282 | 140.266 | 123.583 |
| 517 | 165.004 | 154.695 | 152.322 | 142.197 | 125.355 |
| 517.5 | 166.812 | 156.832 | 154.687 | 145.173 | 127.505 |
| 518 | 168.435 | 158.095 | 156.358 | 147.334 | 128.987 |
| 518.5 | 169.757 | 160.051 | 158.668 | 149.574 | 129.436 |
| 519 | 171.391 | 161.784 | 160.429 | 150.7 | 130.043 |
| 519.5 | 173.816 | 164.098 | 162.057 | 152.84 | 131.183 |
| 520 | 176.302 | 166.932 | 164.07 | 154.77 | 132.757 |
| 520.5 | 178.353 | 169.246 | 165.717 | 156.037 | 134.796 |
| 521 | 180.28 | 171.135 | 167.057 | 157.241 | 136.761 |
| 521.5 | 182.476 | 172.59 | 168.871 | 158.358 | 139.121 |
| 522 | 184.823 | 174.305 | 170.775 | 160.095 | 141.208 |
| 522.5 | 186.613 | 175.795 | 172.136 | 161.516 | 142.38 |
| 523 | 187.333 | 176.803 | 173.623 | 163.042 | 143.211 |
| 523.5 | 188.668 | 178.181 | 174.739 | 163.967 | 145.354 |
| 524 | 190.687 | 178.983 | 176.661 | 165.986 | 147.278 |
| 524.5 | 192.459 | 180.604 | 178.497 | 167.352 | 148.287 |
| 525 | 194.166 | 181.934 | 180.371 | 168.476 | 148.937 |
| 525.5 | 195.843 | 183.385 | 181.811 | 169.252 | 149.474 |
| 526 | 196.656 | 184.679 | 182.388 | 170.476 | 150.521 |
| 526.5 | 197.426 | 186.516 | 182.726 | 172.252 | 151.258 |
| 527 | 197.806 | 188.698 | 183.704 | 173.442 | 151.884 |
| 527.5 | 198.786 | 190.036 | 184.077 | 174.232 | 152.466 |
| 528 | 199.829 | 190.361 | 185.434 | 176.648 | 152.999 |
| 528.5 | 200.322 | 191.361 | 186.194 | 178.152 | 154.394 |
| 529 | 201.383 | 191.751 | 186.694 | 180.029 | 154.391 |
| 529.5 | 202.106 | 191.785 | 187.516 | 180.332 | 154.524 |
| 530 | 202.578 | 192.089 | 188.156 | 180.884 | 155.593 |
| 530.5 | 204.235 | 192.46 | 188.054 | 181.106 | 156.721 |
| 531 | 204.527 | 192.882 | 188.754 | 180.853 | 157.108 |
| 531.5 | 204.98 | 193.53 | 188.716 | 180.987 | 157.766 |
| 532 | 204.862 | 193.958 | 188.698 | 181.91 | 158.148 |
| 532.5 | 204.938 | 194.303 | 188.429 | 181.811 | 158.054 |
| 533 | 204.684 | 194 | 188.378 | 180.618 | 158.412 |
| 533.5 | 204.217 | 193.805 | 188.992 | 180.063 | 158.171 |
| 534 | 203.669 | 193.65 | 188.953 | 180.193 | 158.51 |
| 534.5 | 203.866 | 193.11 | 188.183 | 181.477 | 158.414 |
| 535 | 203.322 | 193.185 | 187.768 | 181.07 | 158.866 |
| 535.5 | 203.916 | 192.461 | 187.551 | 180.42 | 159.017 |
| 536 | 204.332 | 192.133 | 186.983 | 180.185 | 158.461 |
| 536.5 | 203.613 | 191.573 | 186.672 | 180.183 | 157.322 |
| 537 | 203.049 | 191.336 | 186.056 | 179.992 | 156.252 |
| 537.5 | 202.352 | 190.87 | 185.007 | 179.76 | 155.362 |
| 538 | 202.025 | 190.713 | 184.627 | 179.797 | 154.99 |
| 538.5 | 202.14 | 190.177 | 184.035 | 179.26 | 154.485 |
| 539 | 201.653 | 190.516 | 182.88 | 178.655 | 153.783 |
| 539.5 | 201.295 | 190.654 | 181.746 | 177.935 | 152.746 |
| 540 | 200.026 | 190.427 | 181.253 | 177.549 | 151.71 |
| 540.5 | 198.789 | 189.538 | 181.162 | 176.872 | 150.448 |
| 541 | 197.391 | 188.602 | 180.425 | 176.647 | 150.671 |
| 541.5 | 196.399 | 188.159 | 180.856 | 175.959 | 150.098 |
| 542 | 196.034 | 186.966 | 180.657 | 174.591 | 149.08 |
| 542.5 | 194.863 | 184.672 | 179.81 | 173.741 | 147.997 |
| 543 | 194.05 | 182.913 | 179.007 | 173.255 | 146.538 |
| 543.5 | 193.253 | 182.364 | 178.45 | 171.146 | 146.008 |
| 544 | 192.075 | 181.436 | 177.056 | 170.327 | 144.482 |
| 544.5 | 190.925 | 180.12 | 175.822 | 169.393 | 143.586 |
| 545 | 190.227 | 178.66 | 174.557 | 168.506 | 143.243 |
| 545.5 | 189.423 | 177.599 | 172.982 | 167.359 | 141.949 |
| 546 | 188.269 | 176.339 | 171.752 | 166.602 | 140.834 |
| 546.5 | 187.313 | 175.573 | 170.602 | 165.679 | 140.578 |
| 547 | 185.927 | 174.221 | 170.366 | 164.908 | 139.418 |
| 547.5 | 185.087 | 173.355 | 169.368 | 163.299 | 139.161 |
| 548 | 184.082 | 173.233 | 168.18 | 162.133 | 138.204 |
| 548.5 | 183.783 | 173.275 | 166.904 | 161.166 | 137.087 |
| 549 | 183.101 | 172.603 | 165.829 | 159.992 | 136.47 |
| 549.5 | 182.249 | 170.818 | 164.598 | 158.762 | 135.276 |

| [a-terp]/ M | I0/I |
| --- | --- |
| 0 | 0.999988 |
| 1e-06 | 1.06348 |
| 2e-06 | 1.09277 |
| 3e-06 | 1.134 |
| 4e-06 | 1.18898 |
| 5e-06 | 1.28947 |

**Figure S8.** Quenching curves (top) and Stern Volmer plots (bottom) of **2B1C** versus α-terpinene in CHCl_3_ (0, 2, 4, 6, 8, 10 eq.).

Emission intensity/ A.U.

| wavelenth/ nm | 0 eq. | 2 eq. | 4 eq. | 6 eq. | 8 eq. | 10 eq. |
| --- | --- | --- | --- | --- | --- | --- |
| 440 | 3.738 | 6.791 | -1.336 | -1.56 | -1.397 | 1.023 |
| 440.5 | 3.52968 | 6.50877 | -1.02022 | -1.45935 | -1.17129 | 0.95987 |
| 441 | 3.29484 | 6.08981 | -0.856064 | -1.372 | -1.01645 | 0.948838 |
| 441.5 | 3.01113 | 5.77419 | -0.68587 | -1.2411 | -0.868935 | 0.870064 |
| 442 | 2.87632 | 5.40732 | -0.573741 | -1.14713 | -0.680387 | 0.888709 |
| 442.5 | 2.68806 | 5.03393 | -0.354645 | -1.04316 | -0.549419 | 0.926677 |
| 443 | 2.45023 | 4.69616 | -0.103999 | -0.862225 | -0.448935 | 0.958096 |
| 443.5 | 2.24548 | 4.53955 | 0.102064 | -0.748161 | -0.318032 | 1.04668 |
| 444 | 2.00765 | 4.17261 | 0.249741 | -0.594064 | -0.20087 | 1.00016 |
| 444.5 | 1.79952 | 3.91493 | 0.352419 | -0.44329 | -0.110741 | 1.18781 |
| 445 | 1.60155 | 3.80513 | 0.62458 | -0.242322 | -0.013838 | 1.24503 |
| 445.5 | 1.44577 | 3.75929 | 0.74958 | -0.091387 | 0.120612 | 1.24055 |
| 446 | 1.2971 | 3.749 | 0.864806 | 0.12358 | 0.416258 | 1.35877 |
| 446.5 | 1.08903 | 3.73587 | 1.0611 | 0.291419 | 0.62529 | 1.25716 |
| 447 | 0.874419 | 3.77713 | 1.27994 | 0.445516 | 0.783387 | 1.33713 |
| 447.5 | 0.716258 | 3.54268 | 1.41739 | 0.667 | 0.907548 | 1.35874 |
| 448 | 0.622096 | 3.40794 | 1.51829 | 0.704161 | 0.959387 | 1.37716 |
| 448.5 | 0.592419 | 3.36687 | 1.44739 | 0.640193 | 0.97958 | 1.48616 |
| 449 | 0.576258 | 2.99068 | 1.45071 | 0.653677 | 0.938903 | 1.41487 |
| 449.5 | 0.536516 | 2.69474 | 1.36648 | 0.663645 | 0.877806 | 1.43768 |
| 450 | 0.417709 | 2.28971 | 1.41494 | 0.717129 | 0.871129 | 1.43522 |
| 450.5 | 0.361935 | 2.02748 | 1.50855 | 0.774354 | 0.959935 | 1.47445 |
| 451 | 0.342032 | 1.8381 | 1.49881 | 0.801032 | 1.00429 | 1.44184 |
| 451.5 | 0.342322 | 1.76455 | 1.42119 | 0.848129 | 1.086 | 1.331 |
| 452 | 0.442225 | 1.78739 | 1.45403 | 0.821516 | 1.15432 | 1.40903 |
| 452.5 | 0.429193 | 1.62223 | 1.36987 | 0.814806 | 1.19871 | 1.42526 |
| 453 | 0.482516 | 1.63494 | 1.33774 | 0.78458 | 1.15771 | 1.50626 |
| 453.5 | 0.483 | 1.62165 | 1.32155 | 0.767741 | 1.16103 | 1.55752 |
| 454 | 0.516129 | 1.57645 | 1.34764 | 0.747806 | 1.17455 | 1.51829 |
| 454.5 | 0.658483 | 1.60974 | 1.28964 | 0.74429 | 1.17103 | 1.58932 |
| 455 | 0.698419 | 1.62277 | 1.2899 | 0.837612 | 1.14058 | 1.69658 |
| 455.5 | 0.695419 | 1.5709 | 1.35535 | 0.851032 | 1.06287 | 1.80052 |
| 456 | 0.688774 | 1.58323 | 1.20348 | 0.90458 | 1.02194 | 1.73919 |
| 456.5 | 0.748354 | 1.64142 | 1.22955 | 0.88787 | 1.02519 | 1.71968 |
| 457 | 0.883806 | 1.68681 | 1.28764 | 0.84458 | 1.04313 | 1.8201 |
| 457.5 | 0.907 | 1.79642 | 1.30706 | 0.938774 | 1.06448 | 1.91461 |
| 458 | 0.95987 | 1.97123 | 1.30016 | 0.938806 | 1.08216 | 1.94697 |
| 458.5 | 1.026 | 2.13545 | 1.394 | 0.942387 | 1.06168 | 1.99239 |
| 459 | 1.07906 | 2.09684 | 1.45945 | 0.95587 | 1.13748 | 1.87671 |
| 459.5 | 1.09881 | 2.20364 | 1.48213 | 0.962645 | 1.124 | 1.92184 |
| 460 | 1.1711 | 2.21652 | 1.53032 | 0.972903 | 1.18839 | 1.91532 |
| 460.5 | 1.13452 | 2.19342 | 1.38335 | 0.922677 | 1.23642 | 2.00703 |
| 461 | 1.10813 | 2.13497 | 1.48674 | 0.97229 | 1.2709 | 2.0911 |
| 461.5 | 1.18397 | 2.27748 | 1.54542 | 0.891516 | 1.26135 | 2.2569 |
| 462 | 1.29629 | 2.35855 | 1.57781 | 0.844419 | 1.14894 | 2.40339 |
| 462.5 | 1.38145 | 2.49771 | 1.66584 | 0.874451 | 1.31868 | 2.429 |
| 463 | 1.40474 | 2.60768 | 1.91952 | 0.901193 | 1.43232 | 2.49361 |
| 463.5 | 1.41142 | 2.6559 | 1.93929 | 0.987903 | 1.57877 | 2.50052 |
| 464 | 1.501 | 2.53197 | 1.93277 | 1.11452 | 1.69413 | 2.30419 |
| 464.5 | 1.56352 | 2.50964 | 2.06603 | 1.09768 | 1.8131 | 2.24268 |
| 465 | 1.68613 | 2.52348 | 2.30952 | 1.1611 | 1.99087 | 2.24887 |
| 465.5 | 1.80952 | 2.53322 | 2.40368 | 1.24748 | 2.12084 | 2.23181 |
| 466 | 1.84952 | 2.556 | 2.42529 | 1.22335 | 2.20339 | 2.27732 |
| 466.5 | 1.963 | 2.48822 | 2.53574 | 1.26661 | 2.19997 | 2.29977 |
| 467 | 2.00248 | 2.47816 | 2.82977 | 1.42035 | 2.22384 | 2.28965 |
| 467.5 | 2.09103 | 2.54248 | 2.90735 | 1.58755 | 2.398 | 2.32423 |
| 468 | 2.25352 | 2.75693 | 3.11081 | 1.74923 | 2.53787 | 2.441 |
| 468.5 | 2.3589 | 2.78529 | 3.36952 | 1.91939 | 2.71916 | 2.52777 |
| 469 | 2.5301 | 2.8371 | 3.69974 | 2.22987 | 2.73587 | 2.49232 |
| 469.5 | 2.62345 | 2.99842 | 3.99506 | 2.54068 | 2.79365 | 2.61123 |
| 470 | 2.79374 | 3.05984 | 4.09535 | 2.90477 | 2.99842 | 2.67606 |
| 470.5 | 2.893 | 3.25184 | 4.36022 | 3.11845 | 3.13926 | 2.87142 |
| 471 | 3.14168 | 3.50061 | 4.46171 | 3.52126 | 3.32129 | 2.894 |
| 471.5 | 3.42342 | 3.72535 | 4.59239 | 3.70326 | 3.56668 | 3.24084 |
| 472 | 3.53587 | 3.79323 | 4.77229 | 4.05519 | 3.62761 | 3.48174 |
| 472.5 | 3.59294 | 3.80658 | 4.91068 | 4.27177 | 3.68845 | 3.71513 |
| 473 | 3.756 | 3.84939 | 5.07571 | 4.27481 | 3.88742 | 3.93555 |
| 473.5 | 3.97455 | 4.0701 | 5.14361 | 4.51103 | 4.22913 | 4.02065 |
| 474 | 4.16974 | 4.32 | 5.06048 | 4.81861 | 4.55858 | 4.22197 |
| 474.5 | 4.23516 | 4.52155 | 5.09603 | 5.10074 | 4.57084 | 4.56697 |
| 475 | 4.357 | 4.70358 | 5.21277 | 5.40555 | 4.8461 | 4.86348 |
| 475.5 | 4.47606 | 4.93545 | 5.61232 | 5.63964 | 5.16152 | 5.0769 |
| 476 | 4.73413 | 5.09916 | 5.98271 | 6.05781 | 5.52313 | 5.13935 |
| 476.5 | 4.9991 | 5.38374 | 6.11187 | 6.42094 | 5.66465 | 5.30716 |
| 477 | 5.38168 | 5.47403 | 6.35868 | 6.79845 | 5.78132 | 5.40642 |
| 477.5 | 5.72494 | 5.89771 | 6.57445 | 7.2889 | 6.17742 | 5.66022 |
| 478 | 6.18055 | 6.16452 | 6.86384 | 7.77758 | 6.37613 | 5.94723 |
| 478.5 | 6.60406 | 6.27865 | 7.14297 | 8.15668 | 6.7339 | 6.24955 |
| 479 | 6.9629 | 6.55571 | 7.57923 | 8.37729 | 6.92187 | 6.66671 |
| 479.5 | 7.18522 | 6.9521 | 7.72232 | 8.67323 | 7.02877 | 7.17203 |
| 480 | 7.53435 | 7.30016 | 7.84552 | 9.18661 | 7.31965 | 7.83265 |
| 480.5 | 7.97113 | 7.62719 | 8.15926 | 9.54948 | 7.49965 | 8.46648 |
| 481 | 8.30142 | 8.18474 | 8.35403 | 9.88019 | 7.75471 | 8.83235 |
| 481.5 | 8.87706 | 8.5701 | 8.69422 | 10.246 | 8.15287 | 9.37026 |
| 482 | 9.40939 | 9.16403 | 8.81426 | 10.7574 | 8.63016 | 9.90035 |
| 482.5 | 10.0347 | 9.80358 | 8.93752 | 11.1374 | 9.37058 | 10.3655 |
| 483 | 10.3311 | 10.1886 | 9.28084 | 11.6136 | 10.006 | 10.8732 |
| 483.5 | 10.5717 | 10.5224 | 9.77881 | 11.959 | 10.4648 | 11.446 |
| 484 | 11.0657 | 11.0032 | 10.0539 | 12.2233 | 11.0981 | 12.1699 |
| 484.5 | 11.3943 | 11.594 | 10.2487 | 12.6031 | 11.8915 | 12.6656 |
| 485 | 11.8772 | 12.0165 | 10.2932 | 13.1463 | 12.4436 | 13.2028 |
| 485.5 | 12.4809 | 12.4988 | 10.7202 | 13.4408 | 12.9145 | 13.735 |
| 486 | 13.0626 | 13.0409 | 10.9639 | 13.8366 | 13.299 | 13.9882 |
| 486.5 | 13.4453 | 13.7183 | 11.4154 | 13.9605 | 13.9428 | 14.584 |
| 487 | 14.0838 | 14.1032 | 12.0436 | 14.3042 | 14.5855 | 14.8636 |
| 487.5 | 14.5675 | 14.5677 | 12.4182 | 14.8357 | 15.2011 | 15.1827 |
| 488 | 15.0403 | 15.1463 | 13.0248 | 15.4037 | 15.8183 | 15.5439 |
| 488.5 | 15.7395 | 15.5799 | 13.5417 | 16.1207 | 16.1835 | 15.8832 |
| 489 | 16.2505 | 16.2187 | 14.1645 | 16.8601 | 16.544 | 16.3019 |
| 489.5 | 17.1579 | 16.7341 | 14.9488 | 17.2377 | 17.1733 | 16.9162 |
| 490 | 17.995 | 17.3623 | 15.6845 | 17.773 | 18.0807 | 17.7837 |
| 490.5 | 18.7865 | 17.9535 | 16.2415 | 18.3036 | 18.7542 | 18.2524 |
| 491 | 19.2942 | 18.6562 | 16.5527 | 19.0756 | 19.1824 | 18.8678 |
| 491.5 | 19.8692 | 19.3516 | 17.0913 | 19.5414 | 19.6544 | 19.7198 |
| 492 | 20.5225 | 19.9678 | 17.8939 | 20.1069 | 20.4044 | 20.1325 |
| 492.5 | 21.3277 | 20.7937 | 18.6509 | 20.8428 | 21.0341 | 20.7468 |
| 493 | 21.9909 | 21.2372 | 19.3087 | 21.2442 | 21.8027 | 21.5503 |
| 493.5 | 22.4663 | 21.8087 | 19.8128 | 21.6305 | 22.59 | 22.2682 |
| 494 | 23.3263 | 22.541 | 20.9686 | 22.2774 | 23.2765 | 22.7017 |
| 494.5 | 24.0698 | 23.2915 | 21.7269 | 23.0142 | 24.2501 | 23.0679 |
| 495 | 24.8706 | 24.0462 | 22.7651 | 23.5172 | 25.0545 | 23.7364 |
| 495.5 | 25.3782 | 24.6498 | 23.5128 | 23.9133 | 25.6369 | 24.3249 |
| 496 | 25.8364 | 25.3765 | 24.134 | 24.5822 | 26.3503 | 24.8545 |
| 496.5 | 26.9023 | 26.1374 | 24.9387 | 25.375 | 27.0512 | 25.7927 |
| 497 | 27.6064 | 26.6382 | 25.412 | 26.1077 | 27.8375 | 26.6056 |
| 497.5 | 28.4131 | 27.1744 | 26.1534 | 26.8194 | 28.8724 | 27.1023 |
| 498 | 29.0664 | 27.8204 | 27.0094 | 27.6101 | 29.3829 | 27.652 |
| 498.5 | 29.9279 | 28.5665 | 27.8417 | 28.5386 | 29.7427 | 28.2389 |
| 499 | 31.1112 | 29.2018 | 28.6006 | 29.4035 | 30.2544 | 28.9937 |
| 499.5 | 31.8235 | 29.9159 | 29.2316 | 30.2894 | 30.7143 | 29.5999 |
| 500 | 32.8546 | 30.3637 | 29.8818 | 30.8603 | 31.3639 | 30.5965 |
| 500.5 | 33.8456 | 30.9773 | 30.7038 | 31.6633 | 32.1348 | 31.4652 |
| 501 | 34.6034 | 31.7967 | 31.5805 | 32.7881 | 33.2918 | 32.3232 |
| 501.5 | 35.5207 | 32.5526 | 32.4352 | 33.5954 | 34.3395 | 33.1633 |
| 502 | 36.5819 | 33.0722 | 33.2467 | 34.774 | 35.0042 | 33.4846 |
| 502.5 | 37.357 | 33.7885 | 34.0553 | 35.9285 | 35.75 | 34.2433 |
| 503 | 38.4043 | 34.6714 | 35.2376 | 36.4333 | 36.3563 | 35.247 |
| 503.5 | 39.2921 | 35.7764 | 35.9068 | 37.1738 | 37.1958 | 36.0212 |
| 504 | 40.3281 | 37.0262 | 36.7587 | 37.9845 | 38.2264 | 36.8503 |
| 504.5 | 41.0951 | 37.8101 | 37.6889 | 38.6373 | 39.2403 | 37.8236 |
| 505 | 41.7822 | 38.6631 | 38.3493 | 39.7835 | 40.0445 | 38.6566 |
| 505.5 | 42.4768 | 39.5485 | 38.8621 | 40.7403 | 40.4814 | 39.1062 |
| 506 | 43.6728 | 40.4401 | 39.6289 | 41.6628 | 41.2239 | 40.0212 |
| 506.5 | 44.6437 | 41.4844 | 40.3675 | 42.3081 | 42.1488 | 40.7096 |
| 507 | 45.8005 | 42.3821 | 41.0249 | 42.9113 | 43.0623 | 41.2607 |
| 507.5 | 46.9982 | 42.8992 | 41.7768 | 43.6689 | 43.7119 | 42.3509 |
| 508 | 47.5104 | 43.5091 | 42.549 | 44.3142 | 44.5596 | 43.191 |
| 508.5 | 48.1744 | 44.3952 | 43.362 | 45.1863 | 45.3786 | 43.9409 |
| 509 | 49.216 | 45.0477 | 44.4293 | 45.7075 | 46.0256 | 45.2252 |
| 509.5 | 49.9832 | 45.7566 | 44.9858 | 46.2911 | 47.402 | 46.4014 |
| 510 | 50.7695 | 46.385 | 45.7191 | 47.0305 | 48.0995 | 47.3887 |
| 510.5 | 51.4827 | 47.1473 | 46.4978 | 47.9575 | 48.8071 | 48.5497 |
| 511 | 52.5325 | 47.7074 | 47.4079 | 49.0004 | 49.9239 | 49.5057 |
| 511.5 | 53.7989 | 48.8595 | 48.1786 | 50.1952 | 50.8402 | 50.4191 |
| 512 | 54.3137 | 49.6353 | 49.3327 | 50.8045 | 52.191 | 51.0964 |
| 512.5 | 55.0096 | 50.5745 | 50.4812 | 51.3166 | 52.6747 | 51.7023 |
| 513 | 55.9596 | 51.5745 | 51.3667 | 51.8012 | 52.8669 | 52.7168 |
| 513.5 | 56.6961 | 52.3176 | 52.3014 | 52.6131 | 53.5515 | 53.7311 |
| 514 | 57.4787 | 53.3127 | 53.2487 | 52.9891 | 54.5319 | 54.5913 |
| 514.5 | 57.9573 | 54.1011 | 54.1375 | 53.8916 | 55.6452 | 55.1177 |
| 515 | 58.6186 | 54.51 | 55.5349 | 54.9121 | 56.4704 | 55.7889 |
| 515.5 | 59.1233 | 55.4684 | 56.7871 | 55.9907 | 57.0999 | 56.6452 |
| 516 | 59.708 | 56.3405 | 58.0456 | 56.8562 | 58.3185 | 57.1942 |
| 516.5 | 60.2503 | 56.9184 | 59.2523 | 57.1131 | 58.9719 | 58.2244 |
| 517 | 61.3559 | 57.7199 | 60.2359 | 58.0274 | 59.8584 | 58.9734 |
| 517.5 | 61.8433 | 58.3599 | 60.8474 | 58.9032 | 61.0933 | 60.3502 |
| 518 | 62.5734 | 59.0198 | 61.4108 | 59.3849 | 61.5584 | 61.6449 |
| 518.5 | 62.8762 | 59.8373 | 61.7196 | 60.1411 | 62.5763 | 62.5865 |
| 519 | 63.4373 | 60.3781 | 62.3725 | 60.6414 | 63.1505 | 63.4162 |
| 519.5 | 63.7165 | 60.8819 | 62.8543 | 60.828 | 63.9171 | 64.1561 |
| 520 | 64.2646 | 61.4031 | 63.4823 | 61.2474 | 65.0152 | 64.6545 |
| 520.5 | 64.6112 | 62.2249 | 65.0589 | 61.9492 | 65.5107 | 65.4642 |
| 521 | 65.3345 | 62.9179 | 66.1841 | 62.3493 | 66.7041 | 66.3465 |
| 521.5 | 65.6645 | 63.7999 | 67.2716 | 62.7707 | 67.4379 | 67.2083 |
| 522 | 66.5337 | 64.8191 | 68.3029 | 63.6325 | 68.1131 | 67.9984 |
| 522.5 | 67.1851 | 65.8132 | 69.2295 | 64.6139 | 68.5024 | 68.8899 |
| 523 | 67.8239 | 67.2499 | 69.9058 | 65.2691 | 69.2004 | 69.3775 |
| 523.5 | 68.4742 | 67.7884 | 70.556 | 66.3679 | 69.9299 | 70.1062 |
| 524 | 69.4128 | 68.0499 | 71.2963 | 66.9966 | 70.5981 | 70.3366 |
| 524.5 | 69.5754 | 69.174 | 71.5568 | 68.0702 | 71.2395 | 70.0183 |
| 525 | 69.8264 | 69.9317 | 71.9567 | 68.8404 | 71.118 | 70.4023 |
| 525.5 | 70.7948 | 70.3367 | 72.3577 | 69.5118 | 71.6902 | 70.6513 |
| 526 | 72.1232 | 70.8485 | 72.8541 | 70.2176 | 72.2679 | 70.7211 |
| 526.5 | 72.9617 | 71.4659 | 73.0124 | 70.6757 | 72.8012 | 71.1428 |
| 527 | 73.5716 | 71.7542 | 73.7634 | 70.9775 | 74.0415 | 71.747 |
| 527.5 | 74.561 | 72.722 | 74.0909 | 71.6833 | 74.1915 | 72.3982 |
| 528 | 75.0898 | 73.5287 | 74.7162 | 72.5358 | 74.9028 | 73.0686 |
| 528.5 | 75.4041 | 74.3052 | 75.5567 | 73.4299 | 75.5218 | 73.5374 |
| 529 | 76.139 | 74.9933 | 75.9743 | 74.1504 | 75.9223 | 74.1184 |
| 529.5 | 76.2213 | 75.3782 | 75.8997 | 74.9465 | 76.039 | 74.7886 |
| 530 | 76.6288 | 75.8288 | 76.3712 | 75.2878 | 76.0363 | 75.339 |
| 530.5 | 77.4819 | 76.7165 | 76.35 | 75.4466 | 76.5356 | 75.5618 |
| 531 | 77.6889 | 77.0023 | 76.3988 | 75.7038 | 77.0253 | 75.8352 |
| 531.5 | 78.3385 | 77.4144 | 76.3454 | 75.9789 | 76.4997 | 76.5051 |
| 532 | 78.7285 | 77.4025 | 76.1747 | 76.5262 | 76.6685 | 76.0764 |
| 532.5 | 78.5285 | 77.8166 | 76.0817 | 76.9067 | 76.6051 | 76.2242 |
| 533 | 79.5142 | 78.4321 | 76.7263 | 76.6807 | 76.1594 | 75.999 |
| 533.5 | 79.4709 | 79.0527 | 76.9627 | 76.8708 | 76.4075 | 75.7545 |
| 534 | 80.1101 | 79.3355 | 77.4156 | 77.3551 | 76.4245 | 75.629 |
| 534.5 | 80.9125 | 79.1386 | 77.5227 | 77.9853 | 76.759 | 75.5935 |
| 535 | 81.7183 | 79.1184 | 78.2718 | 78.3041 | 76.6591 | 75.6096 |
| 535.5 | 81.9459 | 79.0638 | 78.5648 | 78.3345 | 76.3403 | 75.9848 |
| 536 | 81.7814 | 78.7341 | 77.7222 | 78.2188 | 76.3142 | 75.9838 |
| 536.5 | 81.7518 | 78.7318 | 77.739 | 78.5189 | 76.3251 | 76.0974 |
| 537 | 81.7067 | 78.9455 | 77.3428 | 78.898 | 76.4725 | 75.8991 |
| 537.5 | 81.7582 | 78.3823 | 77.1157 | 79.0822 | 76.1824 | 76.0147 |
| 538 | 81.4468 | 78.0485 | 77.0927 | 78.7854 | 76.6978 | 75.9519 |
| 538.5 | 81.1441 | 77.9253 | 77.2042 | 78.9092 | 76.9113 | 75.9814 |
| 539 | 81.2478 | 78.2159 | 77.0705 | 78.6348 | 76.4647 | 75.751 |
| 539.5 | 80.7726 | 78.6058 | 77.0373 | 78.8785 | 76.6342 | 75.7849 |

| [a-terp]/ M | I0/I |
| --- | --- |
| 0 | 1 |
| 1e-06 | 1.02242 |
| 2e-06 | 1.04373 |
| 3e-06 | 1.03754 |
| 4e-06 | 1.05411 |
| 5e-06 | 1.07036 |

**Figure S9.** Quenching curves (top) and Stern Volmer plots (bottom) of **1PB2C** versus α-terpinene in CHCl_3_ (0, 2, 4, 6, 8, 10 eq.).

Emission intensity/ /counts

| wavelength/ nm | 0 eq. | 2 eq. | 4 eq. | 6 eq. | 8 eq. | 10 eq. |
| --- | --- | --- | --- | --- | --- | --- |
| 450 | 45.066 | -2.633 | -1.685 | -0.969999 | 0 | 28.242 |
| 450.5 | 43.62 | -1.88694 | -1.18726 | -0.522741 | 0.217838 | 27.3301 |
| 451 | 40.683 | -1.11519 | -0.739064 | -0.007322 | 0.453516 | 25.9822 |
| 451.5 | 38.527 | -0.51058 | 0.022 | 0.350741 | 0.745548 | 24.7827 |
| 452 | 36.1728 | 0.02558 | 0.69429 | 0.814387 | 1.15223 | 23.436 |
| 452.5 | 34.1548 | 0.460225 | 1.71987 | 1.436 | 1.36071 | 22.2788 |
| 453 | 32.0555 | 1.08355 | 2.43158 | 2.27019 | 1.96281 | 21.1956 |
| 453.5 | 29.3206 | 1.6981 | 3.24135 | 2.76561 | 2.4651 | 20.0194 |
| 454 | 27.0484 | 2.28493 | 3.90977 | 3.6649 | 2.72206 | 18.5525 |
| 454.5 | 24.5955 | 3.2071 | 4.40471 | 4.34984 | 3.38429 | 17.4376 |
| 455 | 22.9624 | 3.79448 | 5.1429 | 4.9111 | 4.09916 | 16.4725 |
| 455.5 | 21.7134 | 4.56068 | 5.74129 | 5.73929 | 4.60684 | 15.4899 |
| 456 | 19.7361 | 5.40203 | 6.37077 | 6.35077 | 5.34981 | 14.5281 |
| 456.5 | 18.2873 | 6.08552 | 7.01026 | 6.95948 | 5.95658 | 13.317 |
| 457 | 16.5778 | 7.02487 | 7.94923 | 7.54113 | 6.759 | 12.2916 |
| 457.5 | 13.605 | 8.0199 | 9.00677 | 7.99923 | 7.60406 | 11.1146 |
| 458 | 13.0605 | 8.80681 | 9.77274 | 8.74377 | 8.05674 | 10.8289 |
| 458.5 | 13.1735 | 9.32135 | 10.4462 | 9.4089 | 8.9699 | 11.3562 |
| 459 | 12.952 | 9.93181 | 11.006 | 10.2463 | 9.65671 | 11.836 |
| 459.5 | 12.9805 | 10.5207 | 11.7507 | 11.0141 | 10.2948 | 12.1097 |
| 460 | 15.7326 | 11.4745 | 12.4417 | 11.7517 | 10.5471 | 12.5331 |
| 460.5 | 16.3992 | 12.2226 | 13.3234 | 12.3 | 10.9439 | 12.856 |
| 461 | 16.1199 | 13.2656 | 14.2703 | 13.215 | 11.5903 | 13.2856 |
| 461.5 | 17.9467 | 14.4713 | 15.055 | 14.0664 | 12.2006 | 13.787 |
| 462 | 20.0842 | 15.2426 | 15.5528 | 14.4135 | 13.0673 | 14.6599 |
| 462.5 | 21.8118 | 16.0583 | 16.0978 | 15.1575 | 13.7514 | 15.4598 |
| 463 | 21.0332 | 17.1781 | 17.0869 | 15.8437 | 14.5844 | 16.1273 |
| 463.5 | 20.8317 | 18.2633 | 18.1781 | 16.1874 | 15.4179 | 16.6588 |
| 464 | 21.4389 | 19.428 | 19.3683 | 16.8781 | 16.3187 | 17.5168 |
| 464.5 | 23.3514 | 20.4408 | 20.2184 | 17.7812 | 17.2245 | 18.1385 |
| 465 | 24.5792 | 21.4594 | 21.4404 | 18.6298 | 18.2621 | 18.9579 |
| 465.5 | 25.4558 | 22.243 | 22.7584 | 19.7333 | 19.3834 | 19.6931 |
| 466 | 25.8506 | 23.2102 | 23.9638 | 20.8293 | 20.4676 | 20.2983 |
| 466.5 | 27.2521 | 24.0951 | 24.8278 | 21.9105 | 21.4463 | 21.4516 |
| 467 | 28.8794 | 25.5805 | 25.6577 | 22.8201 | 22.583 | 22.4473 |
| 467.5 | 29.7941 | 26.914 | 26.5198 | 24.1287 | 23.8741 | 23.3549 |
| 468 | 30.9704 | 28.625 | 27.64 | 25.1368 | 25.2047 | 24.391 |
| 468.5 | 33.771 | 30.2175 | 28.6015 | 26.3497 | 26.2569 | 25.2366 |
| 469 | 35.4381 | 31.8577 | 29.6635 | 27.6508 | 27.1729 | 25.9761 |
| 469.5 | 35.7646 | 33.6935 | 31.26 | 29.0146 | 28.5812 | 27.288 |
| 470 | 38.003 | 34.8619 | 32.8652 | 30.374 | 29.4754 | 28.5343 |
| 470.5 | 40.0834 | 36.1907 | 34.025 | 31.9043 | 30.8848 | 29.8793 |
| 471 | 42.7318 | 37.6338 | 35.5364 | 33.6608 | 32.0581 | 31.2761 |
| 471.5 | 42.867 | 39.2824 | 37.6265 | 34.7703 | 33.4586 | 32.3243 |
| 472 | 42.2198 | 41.1522 | 39.6047 | 35.969 | 34.8892 | 33.4066 |
| 472.5 | 42.9084 | 43.2211 | 41.2245 | 37.2118 | 36.2989 | 34.5464 |
| 473 | 44.7897 | 45.4058 | 42.3084 | 38.8959 | 37.7843 | 36.237 |
| 473.5 | 46.7673 | 47.6083 | 43.9121 | 40.3809 | 38.9692 | 37.7621 |
| 474 | 50.4127 | 49.5676 | 45.9598 | 42.0166 | 40.2418 | 39.0126 |
| 474.5 | 54.3761 | 51.6181 | 47.7959 | 43.4123 | 42.0055 | 40.7463 |
| 475 | 58.0841 | 54.1056 | 49.8452 | 44.7764 | 43.9728 | 42.1985 |
| 475.5 | 56.7908 | 55.3314 | 52.3962 | 46.5359 | 46.2185 | 43.7239 |
| 476 | 60.2817 | 57.7195 | 54.3146 | 48.1992 | 48.4878 | 44.9704 |
| 476.5 | 64.3417 | 59.9785 | 56.1204 | 49.8373 | 50.607 | 46.8559 |
| 477 | 68.1745 | 62.3615 | 58.1888 | 51.203 | 52.8005 | 48.9196 |
| 477.5 | 70.0861 | 65.0069 | 60.6985 | 53.2798 | 54.8701 | 50.4821 |
| 478 | 72.7965 | 67.1972 | 63.7667 | 55.4372 | 56.8185 | 51.9475 |
| 478.5 | 76.6634 | 69.9583 | 66.1065 | 57.7579 | 59.1313 | 54.1432 |
| 479 | 77.5926 | 72.9547 | 68.5321 | 60.6597 | 61.6801 | 55.6859 |
| 479.5 | 78.2437 | 75.7142 | 70.9878 | 62.965 | 64.2662 | 57.5055 |
| 480 | 81.435 | 78.2187 | 73.5887 | 65.3853 | 66.1699 | 59.6233 |
| 480.5 | 87.1579 | 80.6219 | 76.4877 | 67.8392 | 68.6539 | 62.0242 |
| 481 | 89.248 | 84.0651 | 79.589 | 69.7104 | 71.1933 | 64.2033 |
| 481.5 | 92.3894 | 87.31 | 82.1355 | 71.8229 | 73.3801 | 66.3093 |
| 482 | 97.1915 | 90.4361 | 84.9613 | 73.8555 | 76.2366 | 68.7019 |
| 482.5 | 99.4708 | 93.1862 | 88.0068 | 76.1461 | 78.613 | 70.7721 |
| 483 | 102.344 | 95.6 | 90.5689 | 78.648 | 81.1739 | 73.5415 |
| 483.5 | 108.736 | 98.1586 | 93.0709 | 81.6548 | 83.4665 | 76.0678 |
| 484 | 110.168 | 100.996 | 96.5529 | 84.7335 | 85.7518 | 77.9889 |
| 484.5 | 116.141 | 104.408 | 99.9907 | 88.3368 | 88.5922 | 80.3597 |
| 485 | 120.098 | 107.251 | 103.005 | 91.6455 | 91.5923 | 82.4529 |
| 485.5 | 125.073 | 110.959 | 105.763 | 94.3716 | 94.5544 | 85.0157 |
| 486 | 127.721 | 115.229 | 109.926 | 96.7432 | 97.0786 | 87.1521 |
| 486.5 | 128.236 | 119.11 | 113.431 | 99.5162 | 100.113 | 89.7792 |
| 487 | 134.738 | 123.46 | 116.034 | 103.073 | 102.756 | 92.5777 |
| 487.5 | 139.763 | 126.609 | 118.846 | 106.54 | 105.656 | 95.5576 |
| 488 | 144.3 | 130.184 | 121.369 | 110.254 | 108.941 | 98.0399 |
| 488.5 | 152.469 | 133.346 | 125.129 | 114.127 | 112.522 | 100.373 |
| 489 | 159.586 | 136.519 | 128.871 | 117.54 | 116.169 | 103.764 |
| 489.5 | 162.988 | 140.105 | 131.96 | 121.293 | 118.851 | 107.665 |
| 490 | 165.257 | 144.613 | 135.2 | 124.343 | 121.715 | 110.587 |
| 490.5 | 169.295 | 148.615 | 138.011 | 127.694 | 124.11 | 113.235 |
| 491 | 175.255 | 154.226 | 141.301 | 130.737 | 126.668 | 116.277 |
| 491.5 | 178.764 | 157.813 | 145.569 | 133.683 | 129.792 | 120.137 |
| 492 | 187.486 | 161.151 | 149.016 | 136.776 | 132.531 | 122.851 |
| 492.5 | 187.721 | 164.783 | 151.841 | 140.546 | 135.313 | 124.857 |
| 493 | 190.038 | 167.642 | 153.629 | 144.055 | 138.183 | 127.648 |
| 493.5 | 194.335 | 171.82 | 156.234 | 147.724 | 141.04 | 131.034 |
| 494 | 198.66 | 175.731 | 159.834 | 151.022 | 143.602 | 133.212 |
| 494.5 | 205.045 | 179.642 | 162.429 | 155.151 | 145.882 | 136.195 |
| 495 | 211.265 | 183.54 | 166.003 | 158.838 | 148.433 | 138.573 |
| 495.5 | 212.726 | 187.542 | 169.283 | 161.104 | 151.672 | 140.922 |
| 496 | 218.73 | 191.78 | 171.624 | 164.301 | 154.393 | 144.182 |
| 496.5 | 222.899 | 195.799 | 174.207 | 168.145 | 157.292 | 148.035 |
| 497 | 234.299 | 200.2 | 177.616 | 171.114 | 160.271 | 151.087 |
| 497.5 | 237.231 | 203.973 | 181.234 | 174.058 | 163.269 | 153.444 |
| 498 | 241.598 | 208.486 | 184.553 | 177.442 | 166.371 | 156.136 |
| 498.5 | 246.486 | 212.782 | 188.426 | 180.887 | 169.063 | 158.454 |
| 499 | 248.204 | 217.227 | 192.228 | 183.939 | 171.972 | 161.294 |
| 499.5 | 247.368 | 221.685 | 195.845 | 185.622 | 175.75 | 165.198 |
| 500 | 246.389 | 225.997 | 198.887 | 188.293 | 178.841 | 168.392 |
| 500.5 | 248.841 | 229.984 | 202.162 | 190.851 | 181.347 | 171.302 |
| 501 | 250.411 | 233.303 | 206.152 | 193.828 | 184.636 | 173.543 |
| 501.5 | 252.144 | 236.472 | 208.033 | 196.984 | 187.389 | 176.874 |
| 502 | 257.158 | 239.384 | 210.623 | 199.089 | 190.74 | 179.616 |
| 502.5 | 257.314 | 242.245 | 213.659 | 201.576 | 194.08 | 182.429 |
| 503 | 262.182 | 246.004 | 216.804 | 203.773 | 196.648 | 184.846 |
| 503.5 | 264.901 | 249.772 | 220.503 | 205.707 | 198.686 | 187.24 |
| 504 | 265.736 | 253.233 | 223.69 | 207.558 | 200.486 | 190.14 |
| 504.5 | 264.999 | 257.14 | 226.525 | 209.175 | 204.056 | 191.641 |
| 505 | 264.855 | 259.839 | 229.611 | 210.458 | 207.838 | 192.274 |
| 505.5 | 265.546 | 261.793 | 232.508 | 213.267 | 210.781 | 194.171 |
| 506 | 270.838 | 264.184 | 235.363 | 215.804 | 213.139 | 197.169 |
| 506.5 | 273.127 | 264.836 | 237.043 | 218.1 | 215.69 | 199.495 |
| 507 | 273.031 | 267.37 | 238.921 | 220.183 | 217.077 | 201.298 |
| 507.5 | 270.056 | 270.965 | 241.443 | 222.474 | 218.544 | 203.048 |
| 508 | 275.256 | 272.969 | 244.75 | 224.916 | 220.691 | 205.814 |
| 508.5 | 276.426 | 276.326 | 248.858 | 226.264 | 222.529 | 208.141 |
| 509 | 275.587 | 279.394 | 250.672 | 226.951 | 224.117 | 210.61 |
| 509.5 | 276.184 | 282.178 | 252.47 | 228.187 | 225.978 | 212.303 |
| 510 | 279.805 | 283.284 | 255.454 | 228.185 | 228.216 | 214.719 |
| 510.5 | 282.166 | 285.163 | 256.544 | 229.505 | 230.063 | 217.629 |
| 511 | 284.82 | 287.97 | 259.122 | 231.736 | 232.171 | 219.625 |
| 511.5 | 280.504 | 290.599 | 262.267 | 233.583 | 234.024 | 220.371 |
| 512 | 284.277 | 292.508 | 264.468 | 234.905 | 235.702 | 220.261 |
| 512.5 | 279.852 | 293.474 | 265.862 | 237.342 | 237.783 | 221.264 |
| 513 | 282.355 | 295.663 | 267.378 | 239.733 | 239.378 | 222.91 |
| 513.5 | 285.386 | 297.158 | 268.942 | 242.604 | 241.099 | 224.397 |
| 514 | 287.701 | 299.208 | 270.465 | 244.026 | 243.224 | 225.385 |
| 514.5 | 289.005 | 299.737 | 271.643 | 245.225 | 244.916 | 226.881 |
| 515 | 293.967 | 300.415 | 272.99 | 247.616 | 245.821 | 227.26 |
| 515.5 | 300.072 | 299.632 | 274.983 | 248.759 | 246.434 | 228.073 |
| 516 | 303.332 | 300.559 | 275.383 | 249.597 | 247.975 | 229.129 |
| 516.5 | 307.755 | 301.396 | 276 | 250.174 | 248.71 | 230.538 |
| 517 | 307.764 | 302.045 | 277.513 | 251.226 | 249.333 | 230.711 |
| 517.5 | 310.038 | 304.303 | 279.456 | 252.856 | 250.058 | 230.909 |
| 518 | 313.889 | 304.747 | 280.848 | 253.938 | 250.747 | 230.856 |
| 518.5 | 312.115 | 306.299 | 281.936 | 255.494 | 251.423 | 231.824 |
| 519 | 313.557 | 306.706 | 283.35 | 256.208 | 252.667 | 232.932 |
| 519.5 | 312.83 | 308.852 | 283.261 | 256.742 | 252.958 | 233.72 |
| 520 | 313.498 | 310.441 | 283.445 | 258.136 | 252.566 | 234.774 |
| 520.5 | 314.392 | 310.627 | 284.291 | 259.597 | 251.644 | 236.029 |
| 521 | 317.232 | 310.755 | 285.213 | 259.823 | 251.747 | 237.37 |
| 521.5 | 316.534 | 311.221 | 286.159 | 259.998 | 252.752 | 237.456 |
| 522 | 316.282 | 312.405 | 287.476 | 259.623 | 253.433 | 236.855 |
| 522.5 | 317.599 | 313.073 | 288.445 | 260.346 | 253.914 | 237.024 |
| 523 | 316.343 | 312.945 | 289.189 | 260.267 | 254.761 | 238.042 |
| 523.5 | 316.353 | 313.342 | 290.081 | 259.922 | 254.753 | 238.487 |
| 524 | 320.85 | 312.82 | 291.664 | 261.217 | 254.78 | 238.897 |
| 524.5 | 322.396 | 312.214 | 292.522 | 262.291 | 256.044 | 238.138 |
| 525 | 327.014 | 311.444 | 292.886 | 262.718 | 256.386 | 238.88 |
| 525.5 | 325.226 | 311.947 | 292.47 | 262.888 | 256.101 | 239.318 |
| 526 | 326.018 | 311.695 | 293.204 | 262.816 | 255.905 | 238.818 |
| 526.5 | 330.721 | 311.012 | 292.497 | 262.076 | 255.364 | 238.322 |
| 527 | 331.949 | 309.889 | 291.952 | 261.055 | 254.697 | 238.581 |
| 527.5 | 330.986 | 308.304 | 291.221 | 261.655 | 254.332 | 239.168 |
| 528 | 329.571 | 307.97 | 290.719 | 260.987 | 253.68 | 239.128 |
| 528.5 | 328.928 | 307.212 | 290.273 | 260.39 | 253.394 | 239.166 |
| 529 | 326.534 | 306.819 | 289.896 | 259.284 | 253.3 | 239.166 |
| 529.5 | 322.206 | 306.353 | 289.575 | 258.117 | 252.365 | 238.778 |
| 530 | 321.293 | 306.893 | 289.181 | 257.278 | 251.722 | 237.302 |
| 530.5 | 323.089 | 306.371 | 287.505 | 255.523 | 251.247 | 237.191 |
| 531 | 317.976 | 307.388 | 286.545 | 254.478 | 251.832 | 236.834 |
| 531.5 | 315.103 | 306.52 | 286.355 | 254.318 | 249.976 | 236.175 |
| 532 | 309.21 | 305.19 | 284.711 | 254.601 | 249.117 | 234.818 |
| 532.5 | 311.694 | 304.008 | 283.769 | 253.775 | 248.905 | 234.445 |
| 533 | 309.612 | 301.653 | 282.064 | 253.169 | 247.571 | 233.713 |
| 533.5 | 310.253 | 300.212 | 280.853 | 251.982 | 245.044 | 233.303 |
| 534 | 309.12 | 297.317 | 279.649 | 249.712 | 243.293 | 231.888 |
| 534.5 | 307.788 | 294.907 | 278.062 | 248.978 | 240.935 | 230.241 |
| 535 | 306.464 | 291.098 | 277.568 | 247.218 | 239.577 | 228.344 |
| 535.5 | 305.235 | 286.889 | 276.049 | 244.895 | 238.065 | 225.876 |
| 536 | 306.856 | 285.656 | 273.914 | 242.837 | 237.554 | 223.979 |
| 536.5 | 305.749 | 284.48 | 271.943 | 241.615 | 235.273 | 222.085 |
| 537 | 303.396 | 282.937 | 269.943 | 240.908 | 233.754 | 220.944 |
| 537.5 | 308.397 | 281.443 | 268.527 | 240.327 | 232.31 | 220.27 |
| 538 | 306.963 | 279.994 | 265.683 | 239.016 | 231.173 | 218.952 |
| 538.5 | 307.523 | 278.161 | 263.625 | 238.433 | 228.543 | 216.959 |
| 539 | 302.159 | 276.334 | 260.531 | 236.829 | 227.088 | 214.806 |
| 539.5 | 297.377 | 274.51 | 257.22 | 234.353 | 225.682 | 213.049 |
| 540 | 295.923 | 272.133 | 255.85 | 232.436 | 222.909 | 212.112 |
| 540.5 | 294.591 | 269.573 | 253.359 | 230.011 | 220.353 | 210.206 |
| 541 | 297.463 | 266.861 | 250.928 | 229.039 | 218.837 | 207.767 |
| 541.5 | 292.255 | 264.382 | 248.952 | 226.98 | 217.275 | 205.284 |
| 542 | 284.039 | 262.619 | 246.833 | 225.858 | 214.419 | 203.545 |
| 542.5 | 283.655 | 261.45 | 243.998 | 224.368 | 211.912 | 201.371 |
| 543 | 279.77 | 260.494 | 241.463 | 221.161 | 209.358 | 198.666 |
| 543.5 | 277.313 | 258.577 | 239.43 | 218.712 | 206.673 | 196.708 |
| 544 | 273.868 | 255.985 | 237.206 | 216.374 | 203.807 | 194.061 |
| 544.5 | 268.798 | 252.843 | 234.316 | 213.78 | 200.81 | 191.946 |
| 545 | 268.423 | 249.689 | 231.515 | 211.427 | 198.369 | 190.432 |
| 545.5 | 264.495 | 246.264 | 228.885 | 209.638 | 196.255 | 189.034 |
| 546 | 260.059 | 243.297 | 226.932 | 208.089 | 193.692 | 187.19 |
| 546.5 | 258.804 | 240.672 | 223.297 | 205.541 | 190.917 | 185.27 |
| 547 | 259.501 | 238.381 | 220.753 | 202.654 | 189.608 | 183.388 |
| 547.5 | 260.932 | 235.957 | 218.539 | 199.911 | 187.902 | 182.002 |
| 548 | 256.701 | 233.581 | 215.867 | 197.682 | 185.587 | 179.473 |
| 548.5 | 253.133 | 230.998 | 213.098 | 195.062 | 183.462 | 177.659 |
| 549 | 251.101 | 228.834 | 210.304 | 193.027 | 182.627 | 175.754 |
| 549.5 | 246.512 | 226.236 | 207.556 | 191.454 | 181.017 | 174.331 |

| [a-terp]/ M | I0/I |
| --- | --- |
| 0 | 1 |
| 4e-05 | 1.07119 |
| 8e-05 | 1.137 |
| 0.00012 | 1.27157 |
| 0.00016 | 1.30331 |
| 0.0002 | 1.39135 |

**Figure S10.** Quenching curves (top) and Stern Volmer plots (bottom) of **OH-BTZ** versus α-terpinene in CHCl_3_ (0, 2, 4, 6, 8, 10 eq.).

Emission intensity/ /counts

| wavelength/ nm | 0 eq. | 2 eq. | 4 eq. | 6 eq. | 8 eq. | 10 eq. |
| --- | --- | --- | --- | --- | --- | --- |
| 450 | 21.463 | -1.905 | 1.188 | 19.569 | 1.523 | 11.787 |
| 450.5 | 23.5551 | 0.850322 | 3.92932 | 20.8785 | 3.85048 | 13.132 |
| 451 | 25.5767 | 3.86068 | 6.61952 | 22.0785 | 5.90326 | 15.0087 |
| 451.5 | 27.2401 | 7.34955 | 9.63006 | 23.1194 | 8.29471 | 16.6628 |
| 452 | 28.785 | 10.3267 | 12.469 | 24.5344 | 10.4839 | 19.0029 |
| 452.5 | 30.0933 | 13.345 | 15.4986 | 25.9927 | 12.8816 | 20.9456 |
| 453 | 31.8138 | 15.754 | 18.3504 | 27.4633 | 15.3075 | 22.4357 |
| 453.5 | 33.4603 | 19.163 | 21.895 | 28.5714 | 17.6733 | 23.9084 |
| 454 | 35.0314 | 22.1111 | 24.8507 | 29.617 | 20.0008 | 25.4839 |
| 454.5 | 36.4969 | 25.16 | 27.6633 | 31.4633 | 22.2805 | 27.1743 |
| 455 | 38.6652 | 28.4449 | 30.4859 | 32.864 | 24.6883 | 28.8104 |
| 455.5 | 40.7945 | 31.8414 | 33.5239 | 34.5207 | 27.2891 | 30.7458 |
| 456 | 43.0615 | 34.9389 | 36.4688 | 36.3291 | 29.8621 | 32.9997 |
| 456.5 | 45.388 | 38.0785 | 39.6631 | 37.8116 | 32.5803 | 34.9964 |
| 457 | 47.7567 | 41.0581 | 42.4365 | 39.4887 | 35.0341 | 37.0375 |
| 457.5 | 50.1401 | 44.6146 | 45.6952 | 41.418 | 37.6705 | 39.4103 |
| 458 | 52.7325 | 47.6344 | 48.5293 | 43.5745 | 40.0748 | 41.3993 |
| 458.5 | 55.2415 | 49.2399 | 50.4709 | 45.7254 | 42.1477 | 43.7955 |
| 459 | 58.1133 | 51.3971 | 52.5814 | 48.4385 | 44.466 | 46.043 |
| 459.5 | 60.9401 | 54.2844 | 55.3365 | 50.1962 | 46.7255 | 48.6559 |
| 460 | 63.352 | 57.0909 | 57.8845 | 52.1243 | 48.971 | 50.951 |
| 460.5 | 66.1615 | 59.8483 | 60.7665 | 54.5882 | 51.3784 | 53.2189 |
| 461 | 68.88 | 62.7981 | 63.285 | 57.3663 | 53.6475 | 55.543 |
| 461.5 | 72.7115 | 65.8866 | 66.0021 | 59.5194 | 56.1984 | 57.5571 |
| 462 | 76.0784 | 69.3749 | 68.9452 | 62.7892 | 58.8018 | 60.496 |
| 462.5 | 79.2632 | 72.786 | 72.2963 | 65.8866 | 61.2565 | 63.2287 |
| 463 | 82.9086 | 76.3005 | 75.9034 | 69.6615 | 63.9152 | 65.9024 |
| 463.5 | 86.3202 | 79.3496 | 79.0873 | 72.5426 | 66.0592 | 68.4115 |
| 464 | 90.0878 | 82.85 | 82.4513 | 75.6924 | 68.6627 | 70.7764 |
| 464.5 | 93.3557 | 86.8874 | 85.285 | 79.3041 | 71.6685 | 73.8503 |
| 465 | 97.1415 | 90.5821 | 88.323 | 82.872 | 75.0175 | 76.2714 |
| 465.5 | 101.145 | 93.9002 | 91.8198 | 86.1606 | 78.538 | 79.2324 |
| 466 | 104.962 | 98.2571 | 94.9236 | 89.6796 | 81.4085 | 81.9601 |
| 466.5 | 108.902 | 102.126 | 97.6327 | 93.2971 | 84.1641 | 84.396 |
| 467 | 113.316 | 106.505 | 101.127 | 96.8095 | 87.036 | 87.4157 |
| 467.5 | 117.792 | 110.637 | 104.573 | 99.9183 | 90.2106 | 90.4324 |
| 468 | 122.689 | 114.604 | 107.548 | 103.926 | 92.7415 | 93.5264 |
| 468.5 | 127.077 | 119.238 | 111.026 | 107.393 | 95.4087 | 96.9972 |
| 469 | 131.251 | 123.026 | 114.422 | 111.455 | 98.784 | 100.491 |
| 469.5 | 136.247 | 127.71 | 118.711 | 115.337 | 102.189 | 103.675 |
| 470 | 141.533 | 132.263 | 123.432 | 119.508 | 105.138 | 107.432 |
| 470.5 | 146.544 | 136.457 | 128.03 | 123.129 | 108.804 | 110.428 |
| 471 | 151.412 | 139.94 | 132.808 | 127.096 | 113.296 | 114.472 |
| 471.5 | 156.251 | 144.628 | 137.279 | 130.826 | 116.793 | 117.493 |
| 472 | 161.307 | 150.012 | 141.841 | 135.559 | 120.716 | 121.282 |
| 472.5 | 166.002 | 155.63 | 145.483 | 139.898 | 124.873 | 125.135 |
| 473 | 171.464 | 161.255 | 149.048 | 144.277 | 128.648 | 128.811 |
| 473.5 | 176.28 | 165.966 | 152.387 | 148.478 | 132.658 | 132.448 |
| 474 | 181.726 | 171.946 | 157.084 | 152.529 | 136.56 | 135.889 |
| 474.5 | 186.891 | 177.286 | 161.877 | 157.044 | 140.401 | 139.662 |
| 475 | 192.294 | 182.004 | 166.494 | 163.12 | 144.351 | 144.058 |
| 475.5 | 198.125 | 187.068 | 171.784 | 168.402 | 148.898 | 148.735 |
| 476 | 203.56 | 192.738 | 176.767 | 173.174 | 152.871 | 153.182 |
| 476.5 | 209.269 | 198.138 | 182.353 | 177.727 | 157.404 | 157.817 |
| 477 | 214.708 | 203.281 | 188.788 | 183.221 | 161.675 | 162.685 |
| 477.5 | 220.87 | 208.608 | 193.981 | 187.398 | 166.413 | 167.237 |
| 478 | 227.737 | 213.98 | 197.528 | 191.254 | 170.839 | 171.308 |
| 478.5 | 233.313 | 218.711 | 201.769 | 194.621 | 175.173 | 175.583 |
| 479 | 238.475 | 224.583 | 207.004 | 199.248 | 180.064 | 180.572 |
| 479.5 | 244.529 | 229.994 | 212.175 | 203.706 | 186.135 | 185.962 |
| 480 | 250.473 | 235.202 | 217.592 | 207.266 | 190.797 | 190.298 |
| 480.5 | 256.848 | 240.559 | 223.367 | 211.786 | 195.431 | 194.044 |
| 481 | 262.396 | 245.768 | 229.587 | 216.388 | 200.007 | 198.294 |
| 481.5 | 267.055 | 250.471 | 234.983 | 221.224 | 204.128 | 203.258 |
| 482 | 272.597 | 255.074 | 241.519 | 226.477 | 208.105 | 208.58 |
| 482.5 | 277.902 | 259.973 | 246.789 | 231.75 | 212.673 | 214.016 |
| 483 | 283.342 | 265.588 | 252.819 | 237.484 | 216.693 | 218.838 |
| 483.5 | 289.01 | 271.245 | 258.329 | 242.833 | 221.824 | 223.917 |
| 484 | 295.087 | 277.295 | 263.121 | 248.146 | 227.488 | 228.691 |
| 484.5 | 300.873 | 282.265 | 268.638 | 253.591 | 232.262 | 233.377 |
| 485 | 306.018 | 286.594 | 273.215 | 258.889 | 236.646 | 238.031 |
| 485.5 | 310.865 | 291.746 | 277.934 | 264.199 | 242.149 | 242.52 |
| 486 | 316.588 | 298.284 | 283.482 | 268.95 | 246.434 | 247.948 |
| 486.5 | 323.145 | 305.087 | 288.529 | 273.867 | 250.484 | 251.636 |
| 487 | 328.698 | 311.614 | 293.828 | 278.869 | 254.281 | 255.813 |
| 487.5 | 333.366 | 317.537 | 297.927 | 282.645 | 257.886 | 260.563 |
| 488 | 339.02 | 323.377 | 303.786 | 287.347 | 261.883 | 265.182 |
| 488.5 | 345.168 | 328.815 | 309.678 | 292.975 | 266.258 | 269.482 |
| 489 | 352.22 | 334.264 | 315.826 | 298.342 | 270.988 | 273.146 |
| 489.5 | 358.633 | 339.356 | 320.758 | 302.825 | 274.807 | 276.695 |
| 490 | 364.073 | 344.623 | 325.311 | 306.41 | 278.267 | 280.288 |
| 490.5 | 369.701 | 349.347 | 329.514 | 309.362 | 281.572 | 283.425 |
| 491 | 374.641 | 354.206 | 333.567 | 313.518 | 285.14 | 286.418 |
| 491.5 | 379.769 | 359.504 | 337.921 | 317.986 | 288.328 | 290.578 |
| 492 | 384.295 | 364.351 | 340.84 | 322.203 | 293.345 | 294.709 |
| 492.5 | 388.338 | 369.836 | 343.536 | 325.715 | 297.961 | 297.518 |
| 493 | 392.262 | 374.082 | 347.125 | 330.106 | 301.42 | 300.332 |
| 493.5 | 396 | 378.896 | 352.086 | 333.925 | 304.871 | 305.04 |
| 494 | 399.167 | 384.074 | 355.426 | 337.835 | 307.772 | 309.414 |
| 494.5 | 405.604 | 387.027 | 360.708 | 341.63 | 310.545 | 312.44 |
| 495 | 410.401 | 392.066 | 365.531 | 345.875 | 312.781 | 314.46 |
| 495.5 | 414.65 | 396.667 | 369.501 | 350.943 | 315.98 | 317.338 |
| 496 | 418.811 | 401.229 | 372.272 | 354.216 | 318.836 | 320.67 |
| 496.5 | 423.825 | 406.762 | 374.924 | 358.029 | 321.653 | 323.74 |
| 497 | 429.989 | 411.338 | 378.907 | 360.672 | 324.793 | 327.303 |
| 497.5 | 434.147 | 415.176 | 381.967 | 362.917 | 328.557 | 329.334 |
| 498 | 438.439 | 418.154 | 385.515 | 365.747 | 331.561 | 331.018 |
| 498.5 | 442.909 | 421.257 | 388.054 | 368.058 | 334.873 | 333.333 |
| 499 | 446.329 | 424.274 | 391.091 | 370.179 | 336.997 | 335.355 |
| 499.5 | 449.405 | 425.922 | 394.182 | 372.971 | 338.764 | 338.688 |
| 500 | 452.208 | 429.257 | 396.928 | 375.393 | 342.745 | 341.502 |
| 500.5 | 455.679 | 432.402 | 400.215 | 377.96 | 346.901 | 343.386 |
| 501 | 458.927 | 436.235 | 402.774 | 379.223 | 350.115 | 345.228 |
| 501.5 | 461.904 | 437.16 | 405.049 | 381.557 | 352.976 | 347.065 |
| 502 | 463.279 | 438.438 | 407.064 | 384.141 | 355.341 | 349.077 |
| 502.5 | 465.338 | 439.096 | 409.543 | 386.95 | 358.117 | 351.718 |
| 503 | 467.92 | 440.357 | 413.752 | 389.46 | 360.935 | 352.732 |
| 503.5 | 469.721 | 442.18 | 417.119 | 391.858 | 362.365 | 354.261 |
| 504 | 471.353 | 444.001 | 416.972 | 392.016 | 364.89 | 355.924 |
| 504.5 | 472.63 | 445.587 | 416.933 | 391.981 | 365.887 | 358.176 |
| 505 | 473.052 | 446.69 | 418.301 | 394.479 | 367.212 | 360.188 |
| 505.5 | 476.5 | 448.12 | 419.761 | 397.103 | 369.272 | 361.516 |
| 506 | 479.241 | 449.279 | 421.629 | 399.409 | 370.505 | 363.217 |
| 506.5 | 480.811 | 450.831 | 422.699 | 399.678 | 372.173 | 364.525 |
| 507 | 481.486 | 451.109 | 423.302 | 401.931 | 375.034 | 364.833 |
| 507.5 | 483.535 | 452.079 | 425.36 | 404.092 | 374.839 | 364.925 |
| 508 | 485.59 | 452.897 | 427.574 | 404.849 | 374.472 | 366.483 |
| 508.5 | 487.868 | 453.028 | 429.349 | 405.878 | 374.973 | 366.459 |
| 509 | 489.637 | 452.035 | 430.61 | 406.814 | 374.609 | 366.358 |
| 509.5 | 491.128 | 452.236 | 432.311 | 407.412 | 375.026 | 366.661 |
| 510 | 489.847 | 454.228 | 431.031 | 408.136 | 375.314 | 365.312 |
| 510.5 | 491.721 | 453.446 | 431.066 | 408.612 | 375.985 | 366.744 |
| 511 | 492.192 | 452.988 | 431.964 | 409.343 | 375.88 | 367.798 |
| 511.5 | 491.606 | 453.469 | 433.865 | 409.496 | 375.885 | 368.146 |
| 512 | 490.675 | 453.386 | 434.329 | 409.372 | 375.261 | 367.985 |
| 512.5 | 489.65 | 454.403 | 434.02 | 409.78 | 374.539 | 366.422 |
| 513 | 490.091 | 455.29 | 433.761 | 410.674 | 374.477 | 366.739 |
| 513.5 | 490.061 | 455.833 | 433.079 | 409.883 | 375.126 | 366.76 |
| 514 | 489.462 | 455.229 | 433.342 | 409.131 | 374.904 | 366.467 |
| 514.5 | 489.622 | 455.598 | 432.108 | 408.982 | 375.09 | 367.01 |
| 515 | 489.926 | 456.751 | 431.612 | 408.34 | 375.343 | 365.482 |
| 515.5 | 490.319 | 457.752 | 431.731 | 408.864 | 373.495 | 364.174 |
| 516 | 489.687 | 458.691 | 431.36 | 408.767 | 372.339 | 363.946 |
| 516.5 | 489.508 | 457.979 | 430.783 | 408.611 | 371.363 | 364.579 |
| 517 | 489.957 | 458.321 | 430.702 | 407.53 | 369.605 | 363.826 |
| 517.5 | 490.984 | 457.628 | 431.026 | 406.397 | 368.298 | 362.664 |
| 518 | 491.87 | 458.391 | 429.474 | 405.579 | 368.073 | 361.33 |
| 518.5 | 492.415 | 458.45 | 426.595 | 404.689 | 368.353 | 361.146 |
| 519 | 492.502 | 456.888 | 424.207 | 402.821 | 369.509 | 360.022 |
| 519.5 | 492.304 | 455.579 | 425.452 | 402.551 | 368.265 | 359.234 |
| 520 | 489.668 | 455.158 | 425.619 | 403.027 | 366.882 | 358.808 |
| 520.5 | 488.201 | 454.199 | 425.473 | 400.769 | 366.756 | 358.21 |
| 521 | 485.811 | 453.652 | 424.495 | 399.058 | 366.075 | 357.733 |
| 521.5 | 483.653 | 453.475 | 422.291 | 398.178 | 366.045 | 356.322 |
| 522 | 482.491 | 452.534 | 421.627 | 398.279 | 364.531 | 355.74 |
| 522.5 | 482.083 | 451.858 | 421.342 | 395.328 | 362.957 | 355.392 |
| 523 | 480.705 | 450.444 | 420.526 | 392.399 | 361.827 | 354.892 |
| 523.5 | 478.974 | 448.366 | 418.174 | 391.084 | 361.469 | 353.822 |
| 524 | 476.194 | 447.972 | 416.565 | 388.888 | 360.952 | 353.958 |
| 524.5 | 475.08 | 446.945 | 414.145 | 387.461 | 360.755 | 352.627 |
| 525 | 472.719 | 444.601 | 411.757 | 385.353 | 360.012 | 350.798 |
| 525.5 | 470.761 | 441.584 | 410.995 | 383.245 | 359.891 | 351.094 |
| 526 | 467.147 | 439.82 | 408.658 | 382.437 | 357.592 | 348.475 |
| 526.5 | 466.056 | 437.962 | 406.212 | 380.828 | 356.067 | 346.72 |
| 527 | 463.309 | 433.805 | 404.103 | 380.348 | 355.226 | 345.402 |
| 527.5 | 460.78 | 431.751 | 401.608 | 379.662 | 354.232 | 344.966 |
| 528 | 458.598 | 428.915 | 400.835 | 378.556 | 353.145 | 343.793 |
| 528.5 | 456.161 | 425.579 | 400.056 | 376.784 | 351.39 | 342.228 |
| 529 | 453.935 | 422.251 | 398.598 | 375.315 | 348.694 | 341.214 |
| 529.5 | 451.957 | 420.591 | 395.925 | 374.951 | 347.037 | 339.39 |
| 530 | 449.151 | 416.829 | 394.944 | 372.977 | 345.457 | 335.957 |
| 530.5 | 446.201 | 413.285 | 392.716 | 370.383 | 343.017 | 333.971 |
| 531 | 444.218 | 409.795 | 388.881 | 367.305 | 340.137 | 332.281 |
| 531.5 | 442.623 | 405.581 | 386.063 | 364.606 | 336.917 | 329.456 |
| 532 | 439.93 | 402.515 | 382.643 | 361.806 | 333.552 | 325.751 |
| 532.5 | 434.546 | 399.228 | 378.802 | 360.807 | 332.299 | 323.651 |
| 533 | 429.615 | 398.412 | 375.202 | 357.821 | 329.186 | 322.436 |
| 533.5 | 425.605 | 395.833 | 372.679 | 354.737 | 325.784 | 320.21 |
| 534 | 421.746 | 392.347 | 370.962 | 352.676 | 322.196 | 316.216 |
| 534.5 | 418.608 | 389.666 | 367.835 | 350.274 | 317.806 | 312.91 |
| 535 | 414.261 | 386.981 | 364.957 | 347.429 | 314.77 | 309.096 |
| 535.5 | 411.699 | 383.423 | 362.808 | 344.371 | 311.885 | 305.748 |
| 536 | 409.494 | 378.825 | 359.042 | 341.455 | 308.821 | 302.523 |
| 536.5 | 405.684 | 373.67 | 356.053 | 338.36 | 305.706 | 299.492 |
| 537 | 401.441 | 368.869 | 353.556 | 334.098 | 302.14 | 296.31 |
| 537.5 | 398.016 | 365.445 | 349.71 | 329.213 | 300.029 | 291.793 |
| 538 | 394.474 | 361.19 | 344.791 | 326.137 | 297.065 | 287.658 |
| 538.5 | 390.734 | 358.163 | 340.683 | 323.043 | 294.157 | 284.307 |
| 539 | 386.657 | 355.4 | 337.456 | 319.155 | 290.257 | 281.194 |
| 539.5 | 382.211 | 350.839 | 333.321 | 315.487 | 285.972 | 276.86 |
| 540 | 376.51 | 347.613 | 330.278 | 312.2 | 283.051 | 273.725 |
| 540.5 | 372.815 | 344.327 | 327.669 | 309.091 | 280.668 | 269.914 |
| 541 | 369.808 | 341.587 | 324.782 | 306.376 | 277.343 | 266.031 |
| 541.5 | 365.719 | 337.953 | 320.716 | 301.857 | 275.05 | 262.673 |
| 542 | 361.305 | 333.459 | 316.959 | 297.34 | 272.151 | 258.503 |
| 542.5 | 358.555 | 330.594 | 312.05 | 293.672 | 268.703 | 254.815 |
| 543 | 355.322 | 325.969 | 308.21 | 289.358 | 265.467 | 250.152 |
| 543.5 | 350.928 | 322.068 | 302.677 | 285.224 | 261.546 | 246.972 |
| 544 | 345.908 | 318.958 | 297.561 | 280.447 | 258.229 | 244.018 |
| 544.5 | 341.439 | 315.714 | 292.701 | 276.529 | 254.973 | 239.936 |
| 545 | 336.445 | 311.146 | 288.994 | 271.226 | 251.209 | 236.253 |
| 545.5 | 332.139 | 307.956 | 285.392 | 267.191 | 247.621 | 233.464 |
| 546 | 327.403 | 304.201 | 282.555 | 263.485 | 243.718 | 230.276 |
| 546.5 | 322.206 | 300.396 | 280.225 | 259.368 | 240.808 | 226.629 |
| 547 | 316.552 | 296.977 | 276.152 | 255.519 | 238.365 | 224.512 |
| 547.5 | 312.146 | 293.211 | 272.866 | 252.494 | 235.577 | 222.53 |
| 548 | 308.747 | 289.697 | 269.287 | 248.261 | 232.456 | 218.766 |
| 548.5 | 305.039 | 284.032 | 265.191 | 245.299 | 229.113 | 214.936 |
| 549 | 300.723 | 278.908 | 261.264 | 241.637 | 226.089 | 211.724 |
| 549.5 | 296.384 | 275.018 | 256.688 | 238.23 | 222.382 | 209.135 |

| [a-terp]/ M | I0/I |
| --- | --- |
| 0 | 1 |
| 4e-05 | 1.08654 |
| 8e-05 | 1.13943 |
| 0.00012 | 1.2024 |
| 0.00016 | 1.30944 |
| 0.0002 | 1.33821 |

**Figure S11.** Quenching curves (top) and Stern Volmer plots (bottom) of **mOH-BTZ** versus α-terpinene in CHCl_3_ (0, 2, 4, 6, 8, 10 eq.).

Emission intensity/ /counts

| wavelength/ nm | 0 eq. | 2 eq. | 4 eq. | 6 eq. | 8 eq. | 10 eq. |
| --- | --- | --- | --- | --- | --- | --- |
| 450 | -3.06 | -1.618 | -1.962 | 12.126 | -0.844999 | 1.662 |
| 450.5 | -2.84268 | -1.4831 | -1.91768 | 11.8872 | -0.606645 | 1.63561 |
| 451 | -2.57613 | -1.32197 | -1.84374 | 11.6896 | -0.437999 | 1.50597 |
| 451.5 | -2.32265 | -1.18261 | -1.77687 | 11.4878 | -0.249064 | 1.38258 |
| 452 | -2.08887 | -0.990967 | -1.69897 | 11.0849 | -0.145612 | 1.23648 |
| 452.5 | -1.9181 | -0.843032 | -1.52742 | 10.9939 | -0.058516 | 1.14545 |
| 453 | -1.61623 | -0.503161 | -1.38965 | 10.7282 | -0.020451 | 1.0739 |
| 453.5 | -1.24113 | -0.350516 | -1.22158 | 10.5269 | 0.21258 | 0.983032 |
| 454 | -0.96458 | -0.298451 | -0.985032 | 10.305 | 0.324322 | 0.872548 |
| 454.5 | -0.655161 | -0.18529 | -0.813419 | 10.1982 | 0.415709 | 0.755806 |
| 455 | -0.348838 | -0.098483 | -0.641967 | 10.3268 | 0.502516 | 0.622806 |
| 455.5 | -0.141387 | -0.016064 | -0.440354 | 10.0217 | 0.53658 | 0.560387 |
| 456 | 0.115193 | -0.055645 | -0.294935 | 9.6899 | 0.644225 | 0.482161 |
| 456.5 | 0.488387 | -0.034645 | -0.108064 | 9.61177 | 0.707032 | 0.468483 |
| 457 | 0.859193 | 0.042806 | 0.027129 | 9.307 | 0.842967 | 0.373967 |
| 457.5 | 1.09952 | 0.203129 | 0.150193 | 9.26535 | 0.840387 | 0.29229 |
| 458 | 1.35968 | 0.355999 | 0.209774 | 9.41361 | 0.706354 | 0.27729 |
| 458.5 | 1.65226 | 0.623225 | 0.303483 | 9.01258 | 0.641548 | 0.390354 |
| 459 | 1.81913 | 0.864612 | 0.396419 | 8.7749 | 0.66987 | 0.570774 |
| 459.5 | 1.85174 | 0.965354 | 0.520064 | 8.55277 | 0.808419 | 0.745903 |
| 460 | 2.05761 | 1.18377 | 0.767935 | 8.4701 | 0.922387 | 0.823387 |
| 460.5 | 2.19545 | 1.33732 | 0.985193 | 8.1439 | 1.00803 | 0.836709 |
| 461 | 2.43926 | 1.53968 | 1.05616 | 7.81261 | 1.11371 | 0.955903 |
| 461.5 | 2.73674 | 1.61906 | 1.26174 | 7.52006 | 1.25639 | 1.29081 |
| 462 | 3.01045 | 1.85697 | 1.47358 | 7.48487 | 1.52106 | 1.61026 |
| 462.5 | 3.19116 | 2.15106 | 1.68435 | 7.61326 | 1.95997 | 1.93784 |
| 463 | 3.42468 | 2.36784 | 1.89139 | 7.60461 | 2.35287 | 2.27577 |
| 463.5 | 3.85161 | 2.49871 | 2.26868 | 7.29152 | 2.4059 | 2.39455 |
| 464 | 4.21239 | 2.7309 | 2.44126 | 7.35 | 2.63332 | 2.66358 |
| 464.5 | 4.58561 | 3.026 | 2.70761 | 7.36026 | 2.75087 | 2.93487 |
| 465 | 4.98316 | 3.40368 | 3.02213 | 7.47868 | 3.01952 | 3.30332 |
| 465.5 | 5.31555 | 3.94852 | 3.43519 | 7.76058 | 3.26126 | 3.55232 |
| 466 | 5.78513 | 4.36316 | 3.72071 | 7.74442 | 3.37439 | 3.65929 |
| 466.5 | 6.03145 | 4.6581 | 4.11097 | 7.772 | 3.71181 | 3.89535 |
| 467 | 6.50293 | 5.10868 | 4.59261 | 7.69484 | 4.0921 | 4.22506 |
| 467.5 | 6.89571 | 5.38613 | 5.17877 | 8.00326 | 4.437 | 4.538 |
| 468 | 7.50765 | 5.8299 | 5.64897 | 8.28752 | 4.8439 | 4.9341 |
| 468.5 | 7.94745 | 6.24655 | 6.13797 | 8.59023 | 5.40281 | 5.26206 |
| 469 | 8.4359 | 6.51703 | 6.64293 | 9.20813 | 5.63793 | 5.47129 |
| 469.5 | 8.93942 | 7.03306 | 7.16648 | 9.58864 | 6.07126 | 5.84432 |
| 470 | 9.33439 | 7.69152 | 7.74632 | 9.93635 | 6.5171 | 6.16713 |
| 470.5 | 10.076 | 8.39445 | 8.2151 | 10.2662 | 7.01668 | 6.74019 |
| 471 | 10.6947 | 9.20481 | 8.58955 | 10.635 | 7.66894 | 7.256 |
| 471.5 | 11.5943 | 9.99626 | 9.17652 | 11.3705 | 8.17929 | 7.70345 |
| 472 | 12.357 | 10.6499 | 9.77377 | 12.0449 | 8.76623 | 8.12068 |
| 472.5 | 12.9549 | 11.2919 | 10.351 | 12.8401 | 9.39232 | 8.46952 |
| 473 | 13.9545 | 11.9365 | 11.179 | 13.679 | 10.2815 | 8.89342 |
| 473.5 | 14.8551 | 12.8196 | 11.8335 | 13.8523 | 11.1977 | 9.49387 |
| 474 | 15.5622 | 13.5713 | 12.4061 | 14.4183 | 11.7805 | 10.1486 |
| 474.5 | 16.4505 | 14.542 | 13.279 | 15.2706 | 12.6012 | 10.5264 |
| 475 | 17.4638 | 15.7589 | 14.286 | 16.0092 | 13.3057 | 11.2276 |
| 475.5 | 18.3677 | 16.9035 | 15.028 | 16.5068 | 14.179 | 12.0163 |
| 476 | 19.1896 | 18.1808 | 15.8039 | 17.6176 | 15.1831 | 12.5969 |
| 476.5 | 20.1408 | 19.1583 | 16.8115 | 18.5379 | 16.2288 | 13.4135 |
| 477 | 21.4115 | 20.2972 | 17.6541 | 19.5118 | 17.3735 | 14.0988 |
| 477.5 | 22.8479 | 21.2308 | 18.226 | 20.5552 | 18.1478 | 14.6541 |
| 478 | 24.0744 | 22.3334 | 19.1266 | 21.651 | 19.0116 | 15.199 |
| 478.5 | 25.1508 | 23.5621 | 20.4032 | 22.5351 | 20.014 | 15.7106 |
| 479 | 26.4151 | 24.7339 | 21.208 | 23.5669 | 21.6108 | 16.5773 |
| 479.5 | 27.7651 | 25.7702 | 22.6248 | 24.7533 | 22.6589 | 17.6277 |
| 480 | 29.1715 | 26.9187 | 24.1348 | 26.0095 | 23.6826 | 18.6583 |
| 480.5 | 30.2115 | 28.2148 | 25.6333 | 27.2348 | 24.4694 | 19.7853 |
| 481 | 31.7537 | 29.1873 | 26.8133 | 28.3739 | 25.3252 | 21.042 |
| 481.5 | 33.1346 | 30.5382 | 28.7355 | 29.9277 | 26.5451 | 22.1808 |
| 482 | 34.8585 | 32.2117 | 30.3562 | 31.6841 | 27.5384 | 23.3923 |
| 482.5 | 36.2539 | 33.843 | 31.6561 | 33.091 | 28.5602 | 24.7191 |
| 483 | 38.2053 | 35.2506 | 32.9275 | 34.0502 | 30.045 | 26.3688 |
| 483.5 | 39.8454 | 37.0799 | 34.4021 | 34.9352 | 31.327 | 27.6892 |
| 484 | 41.6972 | 38.5281 | 36.0035 | 36.0668 | 32.3946 | 28.9604 |
| 484.5 | 43.2502 | 40.245 | 37.7465 | 37.1629 | 33.6535 | 30.7879 |
| 485 | 44.9517 | 41.9848 | 39.3794 | 38.434 | 34.8444 | 32.1428 |
| 485.5 | 47.026 | 43.578 | 41.2744 | 39.9132 | 36.2049 | 33.6846 |
| 486 | 48.3747 | 45.4059 | 42.9477 | 41.7335 | 37.2529 | 35.0702 |
| 486.5 | 50.3955 | 47.2724 | 44.7165 | 43.5752 | 39.0771 | 36.6249 |
| 487 | 52.7244 | 49.1021 | 46.4811 | 45.1865 | 41.2248 | 38.1639 |
| 487.5 | 54.9049 | 50.8911 | 48.5013 | 46.7875 | 43.1021 | 39.6144 |
| 488 | 57.2277 | 53.0946 | 50.7806 | 48.3668 | 44.574 | 41.4165 |
| 488.5 | 59.1635 | 55.131 | 52.9445 | 49.8522 | 45.9428 | 43.6664 |
| 489 | 61.6457 | 57.0828 | 54.881 | 51.4988 | 47.6191 | 45.5532 |
| 489.5 | 64.3476 | 59.7458 | 57.1496 | 53.2991 | 49.5801 | 47.3425 |
| 490 | 67.1956 | 61.638 | 59.2287 | 55.273 | 51.2741 | 49.4548 |
| 490.5 | 69.5447 | 63.6083 | 61.0397 | 57.1723 | 52.9973 | 51.2267 |
| 491 | 72.1283 | 65.7264 | 63.3584 | 59.2927 | 54.9641 | 53.0156 |
| 491.5 | 75.3489 | 67.5988 | 65.419 | 61.3288 | 56.4882 | 54.9928 |
| 492 | 78.1016 | 69.8979 | 67.6289 | 63.4858 | 58.2327 | 56.6658 |
| 492.5 | 80.9364 | 72.2731 | 70.1634 | 65.6417 | 60.1617 | 58.3785 |
| 493 | 83.5085 | 74.7503 | 73.0736 | 67.6221 | 62.1209 | 60.7019 |
| 493.5 | 86.2379 | 77.0258 | 75.8628 | 68.9602 | 63.9665 | 62.6534 |
| 494 | 88.5615 | 79.2827 | 77.79 | 71.3543 | 65.5364 | 64.7336 |
| 494.5 | 90.9897 | 82.3099 | 80.7646 | 74.1307 | 67.3111 | 66.7724 |
| 495 | 93.0486 | 85.254 | 83.3977 | 76.2664 | 69.2468 | 68.882 |
| 495.5 | 95.9692 | 88.8155 | 85.8603 | 79.0132 | 71.6395 | 70.9756 |
| 496 | 98.9362 | 91.5788 | 87.7913 | 81.3495 | 74.2882 | 72.7741 |
| 496.5 | 101.49 | 94.1426 | 90.7918 | 83.5938 | 77.1003 | 74.1764 |
| 497 | 104.562 | 97.0438 | 92.7765 | 86.0716 | 79.5229 | 76.0214 |
| 497.5 | 107.203 | 100.031 | 95.3141 | 88.6663 | 81.6874 | 78.2905 |
| 498 | 109.93 | 102.187 | 97.6874 | 91.0005 | 84.2477 | 80.9941 |
| 498.5 | 112.359 | 105.004 | 100.635 | 93.499 | 85.8228 | 82.8631 |
| 499 | 115.035 | 107.245 | 103.461 | 96.6112 | 88.075 | 85.6118 |
| 499.5 | 117.459 | 110.509 | 106.114 | 99.0374 | 91.0002 | 88.2928 |
| 500 | 120.817 | 113.313 | 108.915 | 101.751 | 93.8757 | 90.2481 |
| 500.5 | 123.874 | 116.024 | 110.862 | 104.628 | 95.9701 | 92.3813 |
| 501 | 127.037 | 118.723 | 112.901 | 107.148 | 98.2319 | 94.1071 |
| 501.5 | 130.302 | 121.043 | 115.584 | 109.082 | 101.219 | 96.1706 |
| 502 | 133.627 | 123.737 | 118.199 | 111.481 | 102.948 | 98.2709 |
| 502.5 | 136.774 | 126.696 | 120.665 | 114.558 | 105.145 | 100.261 |
| 503 | 139.442 | 130.342 | 123.316 | 117.024 | 107.747 | 102.593 |
| 503.5 | 142.197 | 132.89 | 125.627 | 119.601 | 110.782 | 105.049 |
| 504 | 144.789 | 135.467 | 127.737 | 122.633 | 113.826 | 107.112 |
| 504.5 | 147.103 | 138.001 | 130.349 | 125.672 | 116.688 | 109.226 |
| 505 | 149.799 | 140.462 | 132.637 | 128.25 | 118.774 | 111.379 |
| 505.5 | 152.587 | 143.321 | 135.393 | 130.536 | 120.955 | 113.72 |
| 506 | 156.016 | 146.462 | 138.011 | 133.117 | 123.775 | 115.64 |
| 506.5 | 159.347 | 149.299 | 140.132 | 135.622 | 125.973 | 117.918 |
| 507 | 162.045 | 152.334 | 142.222 | 137.75 | 129.096 | 120.285 |
| 507.5 | 164.681 | 155.543 | 144.986 | 140.085 | 132.028 | 122.531 |
| 508 | 166.645 | 158.601 | 147.736 | 142.505 | 134.177 | 124.784 |
| 508.5 | 169.028 | 161.804 | 149.671 | 144.278 | 136.243 | 126.537 |
| 509 | 171.583 | 164.547 | 151.998 | 147 | 138.778 | 128.936 |
| 509.5 | 174.995 | 166.839 | 154.834 | 148.483 | 141.918 | 131.193 |
| 510 | 178.427 | 168.446 | 156.666 | 150.67 | 144.546 | 133.362 |
| 510.5 | 182.848 | 171.208 | 159.218 | 153.159 | 146.932 | 135.132 |
| 511 | 185.272 | 173.309 | 161.278 | 155.161 | 149.144 | 136.756 |
| 511.5 | 187.752 | 176.177 | 164.484 | 156.883 | 150.97 | 138.6 |
| 512 | 190.554 | 179.25 | 166.442 | 159.333 | 152.835 | 141.493 |
| 512.5 | 193.406 | 181.424 | 168.918 | 160.729 | 154.801 | 143.675 |
| 513 | 195.885 | 182.991 | 170.346 | 162.892 | 158.035 | 145.147 |
| 513.5 | 198.55 | 185.814 | 172.642 | 164.822 | 160.083 | 146.643 |
| 514 | 200.596 | 188.529 | 174.93 | 166.455 | 162.889 | 148.656 |
| 514.5 | 202.994 | 191.617 | 177.434 | 168.68 | 164.825 | 150.096 |
| 515 | 205.603 | 194.069 | 179.516 | 171.081 | 166.4 | 151.205 |
| 515.5 | 208.204 | 196.881 | 181.358 | 172.393 | 167.939 | 152.166 |
| 516 | 210.99 | 199.756 | 184.456 | 173.539 | 169.987 | 153.586 |
| 516.5 | 213.244 | 201.813 | 186.998 | 175.715 | 172.864 | 155.317 |
| 517 | 215.511 | 203.393 | 189.02 | 177.793 | 175.243 | 157.255 |
| 517.5 | 217.087 | 205.83 | 191.502 | 179.253 | 177.55 | 159.652 |
| 518 | 219.144 | 208.176 | 194.259 | 180.536 | 178.736 | 161.408 |
| 518.5 | 222.064 | 210.038 | 196.342 | 182.209 | 179.675 | 162.943 |
| 519 | 224.75 | 213.035 | 197.742 | 183.576 | 180.751 | 164.438 |
| 519.5 | 227.912 | 216.157 | 199.697 | 184.778 | 182.137 | 166.202 |
| 520 | 230.461 | 219.335 | 201.899 | 186.108 | 183.585 | 167.345 |
| 520.5 | 232.943 | 221.414 | 203.712 | 187.722 | 185.684 | 168.759 |
| 521 | 234.552 | 223.406 | 205.295 | 189.303 | 188.108 | 169.977 |
| 521.5 | 236.02 | 224.704 | 207.433 | 190.309 | 189.128 | 171.449 |
| 522 | 236.915 | 225.824 | 209.557 | 192.616 | 190.361 | 173.027 |
| 522.5 | 238.388 | 226.95 | 209.806 | 194.667 | 191.545 | 174.035 |
| 523 | 240.441 | 228.078 | 209.502 | 196.371 | 192.131 | 175.143 |
| 523.5 | 242.662 | 229.347 | 210.201 | 197.214 | 193.445 | 176.784 |
| 524 | 244.577 | 230.497 | 211.303 | 198.642 | 194.709 | 178.221 |
| 524.5 | 246.231 | 232.118 | 211.952 | 199.818 | 195.131 | 179.397 |
| 525 | 247.884 | 233.877 | 212.652 | 201.536 | 195.817 | 180.879 |
| 525.5 | 249.528 | 235.516 | 214.524 | 202.265 | 196.882 | 181.832 |
| 526 | 250.059 | 235.726 | 214.863 | 203.059 | 197.838 | 182.852 |
| 526.5 | 250.681 | 236.858 | 215.212 | 204.299 | 198.701 | 183.83 |
| 527 | 250.767 | 236.934 | 215.689 | 205.301 | 199.903 | 184.794 |
| 527.5 | 251.862 | 237.668 | 216.739 | 205.52 | 200.384 | 184.864 |
| 528 | 253.139 | 238.839 | 217.511 | 206.324 | 200.942 | 185.969 |
| 528.5 | 254.295 | 239.267 | 218.33 | 206.979 | 200.212 | 187.585 |
| 529 | 255.432 | 240.225 | 219.121 | 207.895 | 199.996 | 188.232 |
| 529.5 | 257.414 | 240.7 | 219.286 | 209.353 | 199.967 | 188.936 |
| 530 | 258.366 | 240.388 | 219.773 | 209.108 | 200.522 | 189.246 |
| 530.5 | 259.471 | 239.822 | 220.405 | 209.328 | 200.651 | 190.472 |
| 531 | 259.559 | 239.452 | 221.245 | 210.177 | 200.451 | 191.736 |
| 531.5 | 259.941 | 239.329 | 220.903 | 211.128 | 200.937 | 192.922 |
| 532 | 260.32 | 240.043 | 220.921 | 210.753 | 200.309 | 193.366 |
| 532.5 | 260.679 | 241.222 | 220.748 | 210.683 | 199.349 | 192.95 |
| 533 | 260.876 | 241.047 | 220.353 | 211.099 | 199.295 | 192.499 |
| 533.5 | 259.945 | 241.027 | 220.004 | 210.539 | 199.298 | 192.989 |
| 534 | 259.087 | 242.028 | 220.327 | 209.845 | 199.081 | 193.021 |
| 534.5 | 258.718 | 241.057 | 220.986 | 209.735 | 199.015 | 193.135 |
| 535 | 258.271 | 239.823 | 221.227 | 209.525 | 198.543 | 192.681 |
| 535.5 | 257.71 | 238.742 | 220.765 | 208.893 | 197.867 | 192.152 |
| 536 | 256.448 | 237.842 | 221.449 | 208.365 | 197.367 | 191.703 |
| 536.5 | 255.808 | 236.752 | 220.894 | 208.097 | 196.012 | 190.994 |
| 537 | 254.782 | 236.643 | 220.425 | 207.146 | 195.872 | 190.18 |
| 537.5 | 253.738 | 237.51 | 219.828 | 205.96 | 195.855 | 188.916 |
| 538 | 253.422 | 237.347 | 221.112 | 205.957 | 194.337 | 188.274 |
| 538.5 | 252.795 | 236.22 | 222.171 | 205.021 | 194.03 | 187.868 |
| 539 | 250.924 | 234.906 | 221.328 | 205.087 | 193.429 | 186.462 |
| 539.5 | 249.936 | 234.254 | 221.306 | 204.566 | 192.961 | 185.751 |
| 540 | 248.904 | 233.522 | 221.329 | 204.091 | 192.492 | 185.264 |
| 540.5 | 247.399 | 233.009 | 221.582 | 204.107 | 191.657 | 184.008 |
| 541 | 245.743 | 232.408 | 220.345 | 203.269 | 190.452 | 183.227 |
| 541.5 | 244.764 | 231.65 | 219.296 | 202.102 | 189.976 | 182.514 |
| 542 | 244.303 | 230.432 | 218.832 | 200.508 | 188.501 | 181.686 |
| 542.5 | 244.285 | 230.075 | 217.674 | 199.137 | 186.879 | 180.929 |
| 543 | 243.081 | 228.63 | 216.169 | 198.234 | 186.206 | 179.79 |
| 543.5 | 241.312 | 227.091 | 214.941 | 197.166 | 185.441 | 178.195 |
| 544 | 239.673 | 226.905 | 214.338 | 194.802 | 184.756 | 176.491 |
| 544.5 | 238.216 | 225.086 | 213.148 | 193.807 | 184.408 | 175.281 |
| 545 | 236.191 | 223.656 | 212.126 | 192.002 | 183.377 | 173.525 |
| 545.5 | 234.606 | 222.915 | 210.338 | 190.931 | 181.845 | 172.064 |
| 546 | 232.784 | 222.234 | 208.034 | 189.817 | 180.627 | 170.288 |
| 546.5 | 231.466 | 221.284 | 205.687 | 188.499 | 179.79 | 168.969 |
| 547 | 230.274 | 220.005 | 204.106 | 186.875 | 178.267 | 167.396 |
| 547.5 | 228.019 | 218.81 | 202.309 | 186.028 | 176.787 | 166.118 |
| 548 | 226.318 | 217.481 | 200.913 | 184.694 | 174.918 | 165.127 |
| 548.5 | 224.661 | 215.747 | 199.645 | 183.365 | 173.18 | 163.772 |
| 549 | 223.299 | 213.879 | 197.682 | 182.287 | 171.918 | 161.866 |
| 549.5 | 221.454 | 210.738 | 195.011 | 181.402 | 171.361 | 160.921 |

| [a-terp]/ M | I0/I |
| --- | --- |
| 0 | 1 |
| 4e-05 | 1.08226 |
| 8e-05 | 1.1839 |
| 0.00012 | 1.2358 |
| 0.00016 | 1.30899 |
| 0.0002 | 1.3552 |

**Figure S12.** Quenching curves (top) and Stern Volmer plots (bottom) of **OPr-BTZ** versus α-terpinene in CHCl_3_ (0, 2, 4, 6, 8, 10 eq.).

Emission intensity/ A.U.

| wavelenth/ nm | 0 eq. | 2 eq. | 4 eq. | 6 eq. | 8 eq. | 10 eq. |
| --- | --- | --- | --- | --- | --- | --- |
| 440 | 0 | 3.338 | 6.78 | 15.757 | -0.476 | 1.193 |
| 440.5 | 0.047745 | 3.27192 | 6.6662 | 15.2066 | -0.21096 | 1.22761 |
| 441 | 0.146058 | 3.14388 | 6.54953 | 14.6563 | -0.12796 | 1.23957 |
| 441.5 | 0.148901 | 3.10827 | 6.51033 | 14.1174 | -0.050666 | 1.15359 |
| 442 | 0.213588 | 3.2517 | 6.476 | 13.635 | 0.085686 | 1.14053 |
| 442.5 | 0.213647 | 3.17435 | 6.49843 | 13.082 | 0.230529 | 1.15561 |
| 443 | 0.261803 | 3.06873 | 6.23251 | 12.4979 | 0.223529 | 1.08643 |
| 443.5 | 0.362607 | 2.98276 | 6.06224 | 11.9444 | 0.144156 | 1.07043 |
| 444 | 0.424176 | 2.9248 | 6.15574 | 11.3321 | 0.232666 | 1.09631 |
| 444.5 | 0.587176 | 2.78867 | 6.17445 | 10.8328 | 0.307294 | 1.19202 |
| 445 | 0.626666 | 2.74237 | 6.13616 | 10.3982 | 0.418372 | 1.20959 |
| 445.5 | 0.742098 | 2.66237 | 6.06167 | 9.80561 | 0.532058 | 1.24959 |
| 446 | 0.820764 | 2.51173 | 5.8968 | 9.21867 | 0.796607 | 1.19727 |
| 446.5 | 0.90498 | 2.48696 | 5.7602 | 8.67412 | 0.913725 | 1.15608 |
| 447 | 0.952549 | 2.39527 | 5.64069 | 8.12412 | 0.982705 | 1.16804 |
| 447.5 | 0.958137 | 2.30939 | 5.57188 | 7.56547 | 1.04306 | 1.23051 |
| 448 | 0.980686 | 2.22355 | 5.43582 | 7.03182 | 1.15694 | 1.24243 |
| 448.5 | 1.05108 | 2.13761 | 5.35814 | 6.56031 | 1.29567 | 1.29918 |
| 449 | 1.02298 | 2.1498 | 5.14271 | 5.99294 | 1.48741 | 1.31974 |
| 449.5 | 0.961294 | 2.12539 | 4.97821 | 5.40323 | 1.59839 | 1.3372 |
| 450 | 0.944313 | 2.02267 | 4.83357 | 4.80802 | 1.73469 | 1.30184 |
| 450.5 | 0.972313 | 2.02392 | 4.60998 | 4.21274 | 1.88235 | 1.32243 |
| 451 | 0.994764 | 1.97449 | 4.56347 | 3.60614 | 2.0181 | 1.34563 |
| 451.5 | 1.08708 | 2.00051 | 4.56159 | 3.05312 | 2.1459 | 1.27076 |
| 452 | 1.23029 | 1.9621 | 4.64869 | 2.57633 | 2.29372 | 1.33047 |
| 452.5 | 1.26686 | 1.99386 | 4.61888 | 2.0011 | 2.39641 | 1.34545 |
| 453 | 1.27808 | 1.97896 | 4.60449 | 1.73724 | 2.50667 | 1.34161 |
| 453.5 | 1.26684 | 1.9789 | 4.694 | 1.79323 | 2.64694 | 1.39504 |
| 454 | 1.23045 | 1.9901 | 4.81708 | 1.75073 | 2.75276 | 1.46524 |
| 454.5 | 1.26729 | 2.01802 | 4.96127 | 1.77588 | 2.7388 | 1.62561 |
| 455 | 1.29514 | 1.95335 | 5.02055 | 1.82357 | 2.7808 | 1.69312 |
| 455.5 | 1.36843 | 1.98435 | 5.19465 | 1.78967 | 2.81421 | 1.7268 |
| 456 | 1.42186 | 2.06016 | 5.48667 | 1.77845 | 2.82527 | 1.70704 |
| 456.5 | 1.54239 | 2.03767 | 5.492 | 1.75059 | 2.86702 | 1.84684 |
| 457 | 1.55376 | 2.00672 | 5.53139 | 1.83245 | 2.87782 | 1.99778 |
| 457.5 | 1.6641 | 2.07365 | 5.59059 | 1.88594 | 2.9447 | 2.00018 |
| 458 | 1.75445 | 2.13265 | 5.55102 | 1.87463 | 2.90857 | 2.09543 |
| 458.5 | 1.80498 | 2.19733 | 5.5478 | 1.87761 | 3.15218 | 2.05365 |
| 459 | 1.8388 | 2.25894 | 5.66575 | 1.89467 | 3.27784 | 2.00367 |
| 459.5 | 1.87814 | 2.37941 | 5.86408 | 1.92302 | 3.31092 | 2.15751 |
| 460 | 1.87271 | 2.45212 | 6.03649 | 1.96835 | 3.40308 | 2.30233 |
| 460.5 | 1.97114 | 2.61363 | 6.12943 | 2.06704 | 3.39745 | 2.22684 |
| 461 | 2.11131 | 2.63875 | 6.31359 | 2.10133 | 3.36384 | 2.28812 |
| 461.5 | 2.20447 | 2.6612 | 6.44231 | 2.12386 | 3.52569 | 2.34127 |
| 462 | 2.31447 | 2.71757 | 6.41425 | 2.25059 | 3.67098 | 2.47533 |
| 462.5 | 2.33139 | 2.78502 | 6.59853 | 2.41953 | 3.83849 | 2.53953 |
| 463 | 2.26643 | 2.89951 | 6.7097 | 2.50939 | 3.96651 | 2.64835 |
| 463.5 | 2.29441 | 3.02845 | 6.6958 | 2.51506 | 3.95218 | 2.79106 |
| 464 | 2.37296 | 3.15769 | 6.6609 | 2.58575 | 4.0167 | 2.89706 |
| 464.5 | 2.4208 | 3.23324 | 6.78082 | 2.79173 | 4.15682 | 3.03153 |
| 465 | 2.43765 | 3.2782 | 6.90018 | 2.85324 | 4.20463 | 3.13814 |
| 465.5 | 2.47424 | 3.36796 | 6.92182 | 2.93786 | 4.1342 | 3.2619 |
| 466 | 2.55041 | 3.56725 | 7.14345 | 3.19225 | 4.18975 | 3.52359 |
| 466.5 | 2.66839 | 3.69055 | 7.34647 | 3.22369 | 4.32137 | 3.71065 |
| 467 | 2.86684 | 3.83586 | 7.51702 | 3.26604 | 4.49463 | 3.88892 |
| 467.5 | 3.04892 | 3.72233 | 7.66514 | 3.3341 | 4.51978 | 3.93314 |
| 468 | 3.1499 | 3.81743 | 7.74708 | 3.49933 | 4.58161 | 3.97994 |
| 468.5 | 3.34855 | 3.92445 | 8.08206 | 3.64349 | 4.8459 | 4.206 |
| 469 | 3.47098 | 4.09337 | 8.25159 | 3.78653 | 5.084 | 4.40337 |
| 469.5 | 3.64463 | 4.19676 | 8.27898 | 3.87147 | 5.29139 | 4.61006 |
| 470 | 3.68392 | 4.44531 | 8.41647 | 3.93345 | 5.30827 | 4.62684 |
| 470.5 | 3.75427 | 4.52123 | 8.55696 | 3.96373 | 5.29731 | 4.81696 |
| 471 | 3.78212 | 4.66188 | 8.58735 | 4.2139 | 5.31098 | 4.99627 |
| 471.5 | 3.85523 | 4.92039 | 8.86076 | 4.38625 | 5.24376 | 5.23421 |
| 472 | 3.87261 | 4.99127 | 9.13351 | 4.5278 | 5.50394 | 5.46325 |
| 472.5 | 3.8759 | 5.13474 | 9.32675 | 4.72586 | 5.67398 | 5.49086 |
| 473 | 4.04855 | 5.35857 | 9.57702 | 4.90008 | 5.77176 | 5.74157 |
| 473.5 | 4.37435 | 5.64569 | 9.84461 | 4.96816 | 5.77714 | 5.96349 |
| 474 | 4.60498 | 5.9209 | 10.0227 | 5.07641 | 5.84239 | 6.0782 |
| 474.5 | 4.8618 | 6.06394 | 10.289 | 5.33939 | 5.85961 | 6.25678 |
| 475 | 5.14208 | 6.21741 | 10.5243 | 5.43592 | 6.03604 | 6.42614 |
| 475.5 | 5.45369 | 6.3801 | 10.819 | 5.66537 | 6.346 | 6.63253 |
| 476 | 5.66647 | 6.59147 | 11.0138 | 6.00365 | 6.53276 | 6.9232 |
| 476.5 | 5.87402 | 6.80031 | 11.2024 | 6.27851 | 6.50253 | 7.18129 |
| 477 | 6.00429 | 6.8169 | 11.2134 | 6.48861 | 6.7939 | 7.52745 |
| 477.5 | 6.14239 | 6.98539 | 11.3663 | 6.59837 | 6.86902 | 7.69965 |
| 478 | 6.46076 | 7.08857 | 11.7027 | 6.83176 | 7.03322 | 7.87782 |
| 478.5 | 6.67941 | 7.36882 | 12.0634 | 7.15239 | 7.22002 | 8.0089 |
| 479 | 6.95755 | 7.59094 | 12.1765 | 7.45835 | 7.34788 | 8.18669 |
| 479.5 | 7.12631 | 7.83127 | 12.4198 | 7.74145 | 7.59986 | 8.26718 |
| 480 | 7.385 | 8.17863 | 12.5343 | 7.94786 | 7.84455 | 8.44767 |
| 480.5 | 7.69925 | 8.44474 | 12.7463 | 8.078 | 8.03941 | 8.62547 |
| 481 | 7.74735 | 8.67449 | 12.8288 | 8.28027 | 8.19563 | 8.83561 |
| 481.5 | 8.01665 | 8.99449 | 12.9019 | 8.70635 | 8.47055 | 9.17525 |
| 482 | 8.16482 | 9.37827 | 13.2189 | 9.05016 | 8.72124 | 9.34572 |
| 482.5 | 8.28255 | 9.80414 | 13.6135 | 9.37572 | 8.95525 | 9.48282 |
| 483 | 8.4529 | 10.0909 | 14.02 | 9.55847 | 9.23412 | 9.67518 |
| 483.5 | 8.71647 | 10.391 | 14.456 | 9.85255 | 9.66492 | 9.92386 |
| 484 | 8.85761 | 10.6526 | 14.786 | 10.1221 | 9.79059 | 10.2466 |
| 484.5 | 9.0277 | 10.9245 | 14.9384 | 10.3255 | 9.87776 | 10.5934 |
| 485 | 9.33276 | 11.1315 | 14.9517 | 10.5016 | 10.0957 | 10.61 |
| 485.5 | 9.71576 | 11.4756 | 15.001 | 10.6964 | 10.35 | 10.7532 |
| 486 | 10.0035 | 11.7319 | 15.3263 | 10.9001 | 10.6442 | 11.0842 |
| 486.5 | 10.1173 | 11.9825 | 15.4711 | 11.172 | 10.9861 | 11.3164 |
| 487 | 10.3577 | 12.3627 | 15.5933 | 11.4297 | 11.1409 | 11.8263 |
| 487.5 | 10.6939 | 12.5992 | 15.9791 | 11.7605 | 11.5128 | 12.0169 |
| 488 | 11.1332 | 12.8816 | 16.1805 | 11.9193 | 11.659 | 12.4451 |
| 488.5 | 11.5723 | 13.1498 | 16.4298 | 12.2361 | 11.8503 | 12.9258 |
| 489 | 11.9687 | 13.3291 | 16.7357 | 12.6232 | 12.1051 | 13.0426 |
| 489.5 | 12.3859 | 13.5535 | 16.9124 | 12.7399 | 12.2458 | 13.2364 |
| 490 | 12.7885 | 13.8282 | 17.0598 | 12.8594 | 12.3828 | 13.397 |
| 490.5 | 13.0523 | 14.2827 | 17.2292 | 13.1962 | 12.7425 | 13.4629 |
| 491 | 13.5064 | 14.6451 | 17.5455 | 13.5745 | 13.2817 | 13.5522 |
| 491.5 | 13.8509 | 14.9433 | 17.7069 | 13.7459 | 13.5502 | 13.816 |
| 492 | 14.1126 | 15.2148 | 17.8783 | 14.0514 | 13.9215 | 13.9972 |
| 492.5 | 14.3877 | 15.3526 | 18.1825 | 14.3927 | 14.2122 | 14.1509 |
| 493 | 14.6267 | 15.602 | 18.3019 | 14.7068 | 14.4948 | 14.4648 |
| 493.5 | 15.0131 | 15.9296 | 18.4644 | 14.8887 | 14.7703 | 14.7249 |
| 494 | 15.2856 | 16.3632 | 18.6777 | 15.119 | 14.9968 | 14.9374 |
| 494.5 | 15.429 | 16.6648 | 18.9852 | 15.3417 | 15.3058 | 15.2288 |
| 495 | 15.6796 | 16.9086 | 19.126 | 15.6802 | 15.6557 | 15.4051 |
| 495.5 | 16.045 | 17.2534 | 19.2005 | 16.0149 | 15.9263 | 15.8232 |
| 496 | 16.2616 | 17.6698 | 19.5604 | 16.2964 | 16.1494 | 16.1748 |
| 496.5 | 16.5443 | 17.9769 | 19.6676 | 16.431 | 16.5301 | 16.5287 |
| 497 | 16.8198 | 18.1325 | 19.8056 | 16.6327 | 16.8114 | 16.8736 |
| 497.5 | 17.2675 | 18.4084 | 19.963 | 16.8189 | 16.9024 | 17.1207 |
| 498 | 17.6116 | 18.6909 | 20.1927 | 17.0486 | 17.1436 | 17.4072 |
| 498.5 | 17.9681 | 18.8601 | 20.2371 | 17.198 | 17.3967 | 17.5497 |
| 499 | 18.0902 | 18.9703 | 20.3637 | 17.6344 | 17.6444 | 17.8699 |
| 499.5 | 18.3691 | 19.0804 | 20.5741 | 17.9301 | 17.8777 | 18.0048 |
| 500 | 18.8332 | 19.2083 | 20.8006 | 18.1688 | 18.3121 | 18.1145 |
| 500.5 | 19.155 | 19.476 | 20.8774 | 18.405 | 18.6219 | 18.2716 |
| 501 | 19.495 | 19.946 | 21.0699 | 18.5997 | 18.6452 | 18.6029 |
| 501.5 | 19.543 | 20.1345 | 21.3264 | 18.7069 | 18.8463 | 18.6817 |
| 502 | 19.7381 | 20.4392 | 21.4469 | 18.9022 | 19.1004 | 18.7478 |
| 502.5 | 19.9955 | 20.8809 | 21.6477 | 19.1249 | 19.1005 | 18.8548 |
| 503 | 20.2299 | 21.4794 | 21.7802 | 19.4742 | 19.3693 | 18.9544 |
| 503.5 | 20.3918 | 21.8625 | 21.6264 | 19.6601 | 19.5599 | 19.1689 |
| 504 | 20.9291 | 21.9505 | 21.7179 | 19.7109 | 19.6565 | 19.5383 |
| 504.5 | 21.3156 | 22.1899 | 21.9972 | 19.8622 | 19.8589 | 19.7794 |
| 505 | 21.6763 | 22.4807 | 22.0129 | 20.1048 | 19.9139 | 20.0513 |
| 505.5 | 21.8614 | 22.5534 | 22.0519 | 20.5382 | 20.2322 | 20.3558 |
| 506 | 22.1427 | 22.7831 | 22.1658 | 20.883 | 20.5344 | 20.5066 |
| 506.5 | 22.4991 | 23.1583 | 22.3957 | 21.0715 | 20.784 | 20.7915 |
| 507 | 22.6208 | 23.4211 | 22.5546 | 21.0673 | 21.0374 | 20.9311 |
| 507.5 | 22.8963 | 23.6942 | 22.5659 | 21.329 | 21.1494 | 20.9588 |
| 508 | 23.3279 | 23.8655 | 22.5383 | 21.4312 | 21.3663 | 21.3213 |
| 508.5 | 23.4993 | 24.2112 | 22.5899 | 21.542 | 21.5645 | 21.4389 |
| 509 | 23.7179 | 24.535 | 22.6493 | 21.8644 | 21.7315 | 21.5044 |
| 509.5 | 24.2618 | 24.8421 | 22.6695 | 22.1591 | 21.8085 | 21.7339 |
| 510 | 24.4531 | 25.0401 | 23.073 | 22.3632 | 22.0409 | 22.0194 |
| 510.5 | 24.6696 | 25.3165 | 23.3219 | 22.5879 | 22.2676 | 22.3043 |
| 511 | 24.9736 | 25.556 | 23.6299 | 22.778 | 22.3646 | 22.2763 |
| 511.5 | 25.1312 | 25.6165 | 23.7879 | 22.9947 | 22.5688 | 22.2874 |
| 512 | 25.5371 | 25.9017 | 23.8099 | 23.0174 | 22.811 | 22.3652 |
| 512.5 | 25.7091 | 26.1134 | 23.8972 | 23.2316 | 23.0631 | 22.1653 |
| 513 | 25.7836 | 26.2171 | 23.9201 | 23.4219 | 23.2714 | 22.2903 |
| 513.5 | 25.7269 | 26.3093 | 24.1149 | 23.5841 | 23.4698 | 22.3452 |
| 514 | 25.9336 | 26.3648 | 24.2692 | 23.5347 | 23.5407 | 22.2405 |
| 514.5 | 26.0436 | 26.5676 | 24.5656 | 23.6422 | 23.742 | 22.603 |
| 515 | 26.1882 | 26.573 | 25.058 | 23.7808 | 24.0131 | 22.8575 |
| 515.5 | 26.3119 | 26.8028 | 25.1889 | 23.915 | 24.3796 | 23.0964 |
| 516 | 26.5478 | 26.9324 | 25.2257 | 24.0773 | 24.3966 | 23.2831 |
| 516.5 | 26.7595 | 26.9939 | 25.2831 | 24.2078 | 24.3013 | 23.5917 |
| 517 | 26.9078 | 27.0368 | 25.3882 | 24.3675 | 24.3373 | 23.6042 |
| 517.5 | 27.093 | 27.1126 | 25.599 | 24.5675 | 24.3874 | 23.5695 |
| 518 | 27.1941 | 27.1222 | 25.6376 | 24.6692 | 24.3987 | 23.7237 |
| 518.5 | 27.1201 | 27.3713 | 25.6763 | 24.777 | 24.6145 | 23.8104 |
| 519 | 27.0652 | 27.4918 | 25.7543 | 25.0394 | 24.7695 | 23.8858 |
| 519.5 | 27.1892 | 27.4312 | 25.8619 | 25.0686 | 24.845 | 23.9 |
| 520 | 27.3448 | 27.5097 | 25.9105 | 25.0358 | 24.9367 | 23.907 |
| 520.5 | 27.4353 | 27.7175 | 25.9913 | 25.1487 | 24.6866 | 24.0989 |
| 521 | 27.4399 | 27.6424 | 26.0289 | 25.2531 | 24.8336 | 24.0531 |
| 521.5 | 27.5519 | 27.5946 | 26.0059 | 25.33 | 25.1372 | 24.077 |
| 522 | 27.7607 | 27.5868 | 26.0717 | 25.431 | 25.1166 | 24.1805 |
| 522.5 | 27.9177 | 27.7265 | 26.1856 | 25.5484 | 25.2028 | 24.0044 |
| 523 | 28.0202 | 27.8783 | 26.2778 | 25.6445 | 25.3978 | 23.9522 |
| 523.5 | 28.0895 | 27.8741 | 26.2697 | 25.6413 | 25.5327 | 23.9259 |
| 524 | 28.1363 | 27.9228 | 26.3575 | 25.7551 | 25.6947 | 24.0452 |
| 524.5 | 28.1071 | 27.8237 | 26.3798 | 25.7328 | 25.8083 | 23.9023 |
| 525 | 28.0989 | 27.9046 | 26.5319 | 25.7386 | 25.82 | 23.9654 |
| 525.5 | 27.9891 | 28.0547 | 26.4864 | 25.5666 | 25.6242 | 24.0512 |
| 526 | 27.8206 | 28.1069 | 26.5821 | 25.6983 | 25.3933 | 24.2655 |
| 526.5 | 27.5436 | 28.0794 | 26.4474 | 25.756 | 25.4652 | 24.1167 |
| 527 | 27.8686 | 27.9883 | 26.5718 | 25.9 | 25.3627 | 24.2387 |
| 527.5 | 27.8604 | 27.8451 | 26.5852 | 26.1376 | 25.4302 | 24.4459 |
| 528 | 27.8307 | 27.6413 | 26.632 | 26.3414 | 25.5959 | 24.5459 |
| 528.5 | 27.8885 | 27.3827 | 26.5747 | 26.3907 | 25.6916 | 24.5003 |
| 529 | 27.8543 | 27.1121 | 26.631 | 26.3324 | 25.6344 | 24.5142 |
| 529.5 | 27.7175 | 27.3233 | 26.3965 | 26.4433 | 25.7262 | 24.4825 |
| 530 | 27.5203 | 27.4234 | 26.164 | 26.5454 | 25.4434 | 24.6825 |
| 530.5 | 27.5108 | 27.3305 | 26.1156 | 26.4429 | 25.4872 | 24.7478 |
| 531 | 27.477 | 27.4669 | 26.0972 | 26.2145 | 25.2989 | 24.5489 |
| 531.5 | 27.2778 | 27.4889 | 26.0681 | 26.0683 | 25.2559 | 24.6517 |
| 532 | 27.3831 | 27.3528 | 26.0982 | 25.928 | 25.1965 | 24.47 |
| 532.5 | 27.4053 | 27.3082 | 26.1528 | 25.8606 | 24.8919 | 24.4657 |
| 533 | 27.1934 | 27.1445 | 26.2535 | 25.7904 | 24.7906 | 24.5969 |
| 533.5 | 27.0268 | 26.9323 | 26.3175 | 25.6725 | 24.7182 | 24.3264 |
| 534 | 26.9118 | 26.5629 | 26.1661 | 25.7708 | 24.5717 | 24.3618 |
| 534.5 | 26.8145 | 26.5263 | 25.9293 | 25.7363 | 24.4955 | 24.3266 |
| 535 | 26.4301 | 26.3591 | 25.7452 | 25.4719 | 24.5624 | 24.1574 |
| 535.5 | 26.3698 | 26.2308 | 25.3575 | 25.5422 | 24.5439 | 24.0397 |
| 536 | 26.2951 | 25.8101 | 25.3153 | 25.6213 | 24.4723 | 24.0266 |
| 536.5 | 26.0812 | 25.6658 | 25.1714 | 25.6975 | 24.4493 | 24.2614 |
| 537 | 26.0273 | 25.6926 | 24.9268 | 25.507 | 24.3941 | 24.3668 |
| 537.5 | 25.8614 | 25.3075 | 24.8435 | 25.5099 | 24.1734 | 24.5092 |
| 538 | 25.711 | 24.9537 | 24.8399 | 25.3124 | 24.0754 | 24.6106 |
| 538.5 | 25.6141 | 24.833 | 24.8467 | 25.093 | 23.8345 | 24.5152 |
| 539 | 25.5905 | 24.7753 | 24.6212 | 24.8974 | 23.8937 | 24.2962 |
| 539.5 | 25.3545 | 24.591 | 24.1961 | 24.8926 | 23.8408 | 24.1888 |

| [a-terp]/ M | I0/I |
| --- | --- |
| 0 | 1 |
| 1e-05 | 1.00765 |
| 2e-05 | 1.06749 |
| 3e-05 | 1.09245 |
| 4e-05 | 1.09502 |
| 5e-05 | 1.17014 |

**Figure S13.** Quenching curves (top) and Stern Volmer plots (bottom) of **OHP-BTZ** versus α-terpinene in CHCl_3_ (0, 2, 4, 6, 8, 10 eq.).

5. [Gel](#_Toc205299237) Permeation Chromatography (GPC) data

Intensity/ counts

| Retention Time /min | Intensity |
| --- | --- |
| 0 | 912 |
| 0.00833 | 912 |
| 0.01667 | 364 |
| 0.025 | 0 |
| 0.03333 | 2 |
| 0.04167 | 5 |
| 0.05 | 8 |
| 0.05833 | 10 |
| 0.06667 | 9 |
| 0.075 | 7 |
| 0.08333 | 5 |
| 0.09167 | 4 |
| 0.1 | 3 |
| 0.10833 | 5 |
| 0.11667 | 7 |
| 0.125 | 11 |
| 0.13333 | 13 |
| 0.14167 | 13 |
| 0.15 | 11 |
| 0.15833 | 6 |
| 0.16667 | 2 |
| 0.175 | -1 |
| 0.18333 | -3 |
| 0.19167 | -3 |
| 0.2 | -3 |
| 0.20833 | -4 |
| 0.21667 | -5 |
| 0.225 | -4 |
| 0.23333 | -4 |
| 0.24167 | -3 |
| 0.25 | -3 |
| 0.25833 | -2 |
| 0.26667 | -1 |
| 0.275 | 0 |
| 0.28333 | 0 |
| 0.29167 | -1 |
| 0.3 | -4 |
| 0.30833 | -6 |
| 0.31667 | -7 |
| 0.325 | -6 |
| 0.33333 | -3 |
| 0.34167 | -1 |
| 0.35 | 2 |
| 0.35833 | 3 |
| 0.36667 | 3 |
| 0.375 | 3 |
| 0.38333 | 2 |
| 0.39167 | 1 |
| 0.4 | 0 |
| 0.40833 | -2 |
| 0.41667 | -4 |
| 0.425 | -6 |
| 0.43333 | -7 |
| 0.44167 | -8 |
| 0.45 | -8 |
| 0.45833 | -7 |
| 0.46667 | -6 |
| 0.475 | -5 |
| 0.48333 | -3 |
| 0.49167 | -1 |
| 0.5 | 0 |
| 0.50833 | 0 |
| 0.51667 | 1 |
| 0.525 | 3 |
| 0.53333 | 6 |
| 0.54167 | 8 |
| 0.55 | 8 |
| 0.55833 | 7 |
| 0.56667 | 3 |
| 0.575 | -1 |
| 0.58333 | -4 |
| 0.59167 | -4 |
| 0.6 | -2 |
| 0.60833 | 0 |
| 0.61667 | 1 |
| 0.625 | 1 |
| 0.63333 | 0 |
| 0.64167 | -1 |
| 0.65 | -2 |
| 0.65833 | -3 |
| 0.66667 | -4 |
| 0.675 | -6 |
| 0.68333 | -7 |
| 0.69167 | -8 |
| 0.7 | -8 |
| 0.70833 | -6 |
| 0.71667 | -3 |
| 0.725 | -1 |
| 0.73333 | 0 |
| 0.74167 | -1 |
| 0.75 | -3 |
| 0.75833 | -5 |
| 0.76667 | -7 |
| 0.775 | -7 |
| 0.78333 | -8 |
| 0.79167 | -9 |
| 0.8 | -9 |
| 0.80833 | -9 |
| 0.81667 | -7 |
| 0.825 | -4 |
| 0.83333 | -1 |
| 0.84167 | 1 |
| 0.85 | 3 |
| 0.85833 | 4 |
| 0.86667 | 4 |
| 0.875 | 4 |
| 0.88333 | 4 |
| 0.89167 | 4 |
| 0.9 | 4 |
| 0.90833 | 3 |
| 0.91667 | 3 |
| 0.925 | 2 |
| 0.93333 | 1 |
| 0.94167 | 1 |
| 0.95 | 1 |
| 0.95833 | 2 |
| 0.96667 | 1 |
| 0.975 | 0 |
| 0.98333 | 1 |
| 0.99167 | 4 |
| 1 | 7 |
| 1.00833 | 9 |
| 1.01667 | 9 |
| 1.025 | 7 |
| 1.03333 | 4 |
| 1.04167 | 3 |
| 1.05 | 4 |
| 1.05833 | 7 |
| 1.06667 | 10 |
| 1.075 | 14 |
| 1.08333 | 15 |
| 1.09167 | 15 |
| 1.1 | 14 |
| 1.10833 | 15 |
| 1.11667 | 15 |
| 1.125 | 17 |
| 1.13333 | 18 |
| 1.14167 | 19 |
| 1.15 | 18 |
| 1.15833 | 17 |
| 1.16667 | 15 |
| 1.175 | 12 |
| 1.18333 | 11 |
| 1.19167 | 11 |
| 1.2 | 13 |
| 1.20833 | 17 |
| 1.21667 | 19 |
| 1.225 | 19 |
| 1.23333 | 16 |
| 1.24167 | 14 |
| 1.25 | 13 |
| 1.25833 | 14 |
| 1.26667 | 17 |
| 1.275 | 20 |
| 1.28333 | 21 |
| 1.29167 | 21 |
| 1.3 | 20 |
| 1.30833 | 18 |
| 1.31667 | 16 |
| 1.325 | 15 |
| 1.33333 | 15 |
| 1.34167 | 16 |
| 1.35 | 17 |
| 1.35833 | 19 |
| 1.36667 | 21 |
| 1.375 | 22 |
| 1.38333 | 23 |
| 1.39167 | 24 |
| 1.4 | 25 |
| 1.40833 | 25 |
| 1.41667 | 25 |
| 1.425 | 24 |
| 1.43333 | 22 |
| 1.44167 | 21 |
| 1.45 | 22 |
| 1.45833 | 25 |
| 1.46667 | 29 |
| 1.475 | 34 |
| 1.48333 | 37 |
| 1.49167 | 39 |
| 1.5 | 40 |
| 1.50833 | 38 |
| 1.51667 | 36 |
| 1.525 | 33 |
| 1.53333 | 32 |
| 1.54167 | 33 |
| 1.55 | 37 |
| 1.55833 | 42 |
| 1.56667 | 47 |
| 1.575 | 52 |
| 1.58333 | 55 |
| 1.59167 | 58 |
| 1.6 | 58 |
| 1.60833 | 58 |
| 1.61667 | 55 |
| 1.625 | 52 |
| 1.63333 | 49 |
| 1.64167 | 47 |
| 1.65 | 47 |
| 1.65833 | 48 |

**Figure S14.** GPC chromatogram of **2B2C** oligomer.

**Table S1.** Summarised results from GPC chromatogram of **2B2C** oligomer.

| Peak# | Mn | Mw | Mz | Mw/Mn | Start Time/ min | Top Time/ min | End Time/ min |
| --- | --- | --- | --- | --- | --- | --- | --- |
| 1 | 2130 | 3282 | 4647 | 1.54125 | 14.19 | 16.48 | 19.29 |

6. Single Crystal X-ray Structures and Information

**General crystallographic information:** Data for **2B1C**, **1B2C** were collected at 100K on a Bruker D8 Venture diffractometer operating with Mo- and Cu-Ka radiation, respectively. Data for **1B2C-Pr** were collected at 100K on a Rigaky Synergy diffractometer operating with Cu-Ka radiation generated using a rotating anode source. Specific details relating to each structure are listed below under each figure, including CCDC reference numbers.

**Figure S15.** Single crystal X-ray structures of **2B1C** in ball and stick representation. Colour code: C – grey, N – blue, O – red, S – yellow. Impertinent H atoms except those involved in stabilising H-bonding interactions (shown as dashed red lines) at the lower-rim are omitted for clarity.

**Crystal Data for 2B1C (CCDC 2555361):** C_58_H_48_N_4_O_4_S_2_, *M* =929.12 g/mol, monoclinic, space group *P*2_1_/*n* (no. 14), *a* = 12.5222(4) Å, *b* = 25.9753(7) Å, *c* = 14.0145(4) Å, *β* = 93.0361(11)°, *V* = 4552.1(2) Å^3^, *Z* = 4, *T* = 100(2) K, μ(MoKα) = 0.173 mm^-1^, *Dcalc* = 1.356 g/cm^3^, 144939 reflections measured (5.284° ≤ 2Θ ≤ 60.088°), 13331 unique (*R*_int_ = 0.0799, R_sigma_ = 0.0427) which were used in all calculations. The final *R*_1_ was 0.0560 (I > 2σ(I)) and *wR*_2_ was 0.1523 (all data).

**Figure S16.** Single crystal X-ray structure of **1B2C** in ball and stick representation. Colour code: C – grey, N – blue, O – red, S – yellow. H atoms except those involved in stabilising H-bonding interactions (shown as dashed red lines) at the lower-rim are omitted for clarity.

**Crystal Data for 1B2C (CCDC 2555362):** C_75.5_H_71.5_Cl_4.5_N_2_O_8_S, *M* =1326.43 g/mol, triclinic, space group *P*-1 (no. 2), *a* = 10.2303(2) Å, *b* = 11.5271(2) Å, *c* = 16.5492(3) Å, *α* = 93.7210(10)°, *β* = 107.2520(10)°, *γ* = 114.6070(10)°, *V* = 1654.15(5) Å^3^, *Z* = 1, *T* = 100(2) K, μ(CuKα) = 2.579 mm^-1^, *Dcalc* = 1.332 g/cm^3^, 56000 reflections measured (5.728° ≤ 2Θ ≤ 159.416°), 7112 unique (*R*_int_ = 0.0516, R_sigma_ = 0.0328) which were used in all calculations. The final *R*_1_ was 0.0549 (I > 2σ(I)) and *wR*_2_ was 0.1681 (all data).

**Figure S17.** Singel crystal X-ray structure of **1B2C-Pr** in ball and stick representation showing the BTZ PS linker tethered to aryl rings that are in the pinched positions within their respective C[4] pinched cone conformers. Colour code: C – grey, N – blue, O – red, S – yellow. H atoms omitted for clarity

**Crystal Data for 1B2C-Pr (CCDC 2555363):** C_87_H_97.501_N_2.5_O_8_S, *M* =1338.23 g/mol, monoclinic, space group *P*2/*c* (no. 13), *a* = 18.0612(3) Å, *b* = 9.3019(2) Å, *c* = 44.1180(9) Å, *β* = 99.962(2)°, *V* = 7300.2(3) Å^3^, *Z* = 4, *T* = 100.15 K, μ(Cu Kα) = 0.861 mm^-1^, *Dcalc* = 1.218 g/cm^3^, 51772 reflections measured (4.968° ≤ 2Θ ≤ 143.464°), 20454 unique (*R*_int_ = 0.1369, R_sigma_ = 0.0744) which were used in all calculations. The final *R*_1_ was 0.0833 (I > 2σ(I)) and *wR*_2_ was 0.2173 (all data).

**Figure S18.** Partial single crystal X-ray structure of **1B2C-Pr** in ball and stick representation showing the BTZ PS linker tethered to aryl rings that are in the pinched positions within their respective C[4] pinched cone conformers. Colour code: C – grey, N – blue, O – red, S – yellow. Note the extreme disorder associated with the entire molecule, with C[4]s being over two positions and the BTZ linker being over 4 positions. It was not possible to arrive at a sensible model; hence we have only reported the unit cell parameters for this structure: Monoclinic, space group *P*2_1_/*c*, *a* = 9.3532(6), *b* = 16.4246(7), *c* = 23.4067(8) Å, *β* = 97.575(5) °.

7. [Photosensitisation tests in batch](#_Toc205299241)

General photosensitisation test procedure:

A 25 mL capped glass vial was charged with a magnetic stirring bar and BTZ photosensitiser (0.005 mmol). To this, *α*-terpinene (27.2 mg, 0.2 mmol) and solvent (3 mL) were added and the vial closed. The solution was placed on a magnetic stirring plate (360 rpm) at 7 cm distance from a 12 W 410–420 nm LED block. The reaction was monitored at the time intervals specified in **Table S2** by collecting an aliquot of 0.2 mL of reaction solution into an NMR tube. These samples were examined by ^1^H NMR using a Bruker 300 MHz spectrometer, by integrating typical peaks for *α*-terpinene (m, 5.6 ppm) and ascaridole (dd, 6.5 ppm).^3^

**Figure S19.** Stacked ^1^H NMR spectra reflecting the reaction progression of the photo-oxidation of *α*-terpinene at time 0, 20, 40, and 60 minutes, in the range of 6.6-5.4 ppm. In yellow: typical peaks for *α*-terpinene (m, 5.6 ppm); in purple: typical ascaridole peak (dd, 6.5 ppm).^3^

[Table S2](#_Toc205299242). Photosensitisation tests for the conversion of α-terpinene to ascaridole in batch. (a) test performed under nitrogen atmosphere. (b) photosensitiser not fully dissolved in solvent tested. (c) loading halved to 1.25 mol% due to presence of two catalytic active sites per photosensitiser unit. (d) additive DABCO singlet oxygen scavenger 2 eq. (e) no LED lamp used. (f) Oxygen gas flown through solution for 3 minutes prior to addition of *α*-terpinene and irradiation. (g) catalyst recovery test, using 1B2C recycled from a previous run.

| ENTRY | PC | SOLVENT | TIME/ hours | LOADING/ mol% | CONVERSION/ % |
| --- | --- | --- | --- | --- | --- |
| Controls: |  |  |  |  |  |
| 1 | OH-BTZ | CDCl_3_ | 1 | 2.5 | **12** |
|  | OH-BTZ | CDCl_3_ | 2 | 2.5 | **23** |
| 2 | mOH-BTZ | CDCl_3_ | 1 | 2.5 | **16** |
| 3 | OHP-BTZ | CDCl_3_ | 1 | 2.5 | **15** |
| 4 | Pr-BTZ | CDCl_3_ | 1 | 2.5 | **24** |
| 5 | no | CDCl_3_ | 1 | - | **0** |
|  | no | CDCl_3_ | 2 | - | **0** |
| 6^a^ | 1B2C | CDCl_3_ | 1 | 2.5 | **trace** |
| 7^e^ | 1B2C | CDCl_3_ | 1 | 2.5 | **trace** |
| 8^b^ | 1B2C | acetone | 1 | 2.5 | **83** |
| 9^b^ | 1B2C | acetonitrile | 1 | 2.5 | **88** |
| 10 | 1B2C | CHCl_3_ | 1 | 2.5 | **94** |
| 11 | 1B2C | THF | 1 | 2.5 | **87** |
| Reproducibility tests |  |  |  |  |  |
| 12 | 1B2C | CDCl_3_ | 1 | 2.5 | **99** |
|  | 1B2C | CDCl_3_ | 2 | 2.5 | **99** |
| 13 | 1B2C | CDCl_3_ | 1 | 2.5 | **98** |
|  | 1B2C | CDCl_3_ | 2 | 2.5 | **99** |
| 14 | 1B2C | CDCl_3_ | 1 | 2.5 | **93** |
|  | 1B2C | CDCl_3_ | 2 | 2.5 | **99** |
| PC Library tests |  |  |  |  |  |
| 15^c^ | 2B2C | CDCl_3_ | 1 | 1.25^c^ | **90** |
| 16 | 1B2C-Pr | CDCl_3_ | 1 | 2.5 | **44** |
| 17 | 1PB2C | CDCl_3_ | 1 | 2.5 | **17** |
| 18^c^ | 2B1C | CDCl_3_ | 1 | 1.25^c^ | **33** |
| 19 | 1B2C-Me | CDCl_3_ | 1 | 2.5 | **94** |
| 20 | 1B2C-OH | CDCl_3_ | 1 | 2.5 | **92** |
| 21^d^ | 1B2C | CDCl_3_ | 1 | 2.5 | **trace** |
| 22^f^ | 1B2C | CDCl_3_ | 0.5 | 5 | **98** |
| 23^g^ | 1B2C | CDCl_3_ | 1 | 2.5 | **88** |

**Michaelis-Menten Analysis**

V0 (µM/min)

| [α-terpinene] (mM) | v0 uM/min |
| --- | --- |
| 0.00125 | 75 |
| 0.0025 | 87.5 |
| 0.005 | 82 |
| 0.0125 | 105 |
| 0.025 | 125 |

**Figure S20**. Michaelis-Menten plot, using varying concentrations of [α-terpinene] to **1B2C** (0.5, 1, 2, 5, 10 molar equivalents). V_0_ for each point was obtained by measuring conversion of [ascaridole] (µM, *via* ^1^H NMR integration) over the first 5 minutes of reaction.^16–18^

1/V0 (min µM-1)

| 1/[α-terpinene] (mM-1) | 1/v0 |
| --- | --- |
| 800 | 0.00266667 |
| 400 | 0.00228571 |
| 200 | 0.00243902 |
| 80 | 0.00190476 |
| 40 | 0.0016 |

**Figure S21**. Lineweaver-Burk plot, using varying concentrations of [α-terpinene] to **1B2C** (0.5, 1, 2, 5, 10 molar eq.) obtained from data reported in figure S20.^16–18^

Resulting values of K_m_ and V_max_ according to literature protocol:^16–18^

K_m_: 6.21 x10^-4^ mM

V_max_: 545.5 µM min^-1^

8. [References](#_Toc205299249)

1	C. M. S. Jones, A. Gakamsky and J. Marques-Hueso, The upconversion quantum yield (UCQY): a review to standardize the measurement methodology, improve comparability, and define efficiency standards, *Sci. Technol. Adv. Mater.*, 2021, **22**, 810–848.

2	C. D. Gutsche and P. F. Pagoria, Calixarenes. 16. Functionalized calixarenes: the direct substitution route, *J. Org. Chem.*, 1985, **50**, 5795–5802.

3	M. U. Main, D. Taylor, L. Amicosante, J. M. Tobin, C. M. S. Jones, J. Marques-Hueso, L. J. McCormick McPherson, S. J. Teat, D. Ellis, M. J. Paterson, F. Vilela and S. J. Dalgarno, Host Cavities Enhance the Photocatalytic Conversion of α -Terpinene to Ascaridole Under Visible-Light Irradiation, *Chem. - A Eur. J.*, 2025, **31**, e02113.

4	M. A. Tairov, M. O. Vysotsky, O. I. Kalchenko, V. V Pirozhenko and V. I. Kalchenko, Symmetrical and inherently chiral water-soluble calix [ 4 ] arenes bearing dihydroxyphosphoryl groups, 2002, **6**, 1405–1411.

5	A. Arduini, E. Brindani, G. Giorgi, A. Pochini and A. Secchi, Recognition of guests bearing donor and acceptor hydrogen bonding groups by heteroditopic calix [ 4 ] arene receptors, *Tetrahedron*, 2003, **59**, 7587–7594.

6	C. D. Gutsche and L.-G. Lin, Calixarenes 12, *Tetrahedron*, 1986, **42**, 1633–1640.

7	M. Lee, K. Yang, C. Tsoo, C. Shu and L. Lin, Calix [ 4 ] quinone . Part 1 : Synthesis of 5-hydroxycalix [ 4 ] arene by calix [ 4 ] quinone monoketal route, *Tetrahedron*, 2001, **57**, 8095–8099.

8	M. Ve, J. Gagnon, K. Villeneuve, M. Drouin and P. D. Harvey, ( η 5 -Pentamethylcyclopentadienyl ) rhodium Complexes of Upper-Rim Monophosphinated Calix [ 4 ] arene, *Organometallics*, 2001, **20**, 273–281.

9	G. Hennrich, T. Murillo, P. Prados, H. Al-saraierh, A. Teshome, I. Asselberghs and K. Clays, Alkynyl Expanded Donor – Acceptor Calixarenes : Geometry and Second- Order Nonlinear Optical Properties, *Chem. - A Eur. J.*, 2007, **13**, 7753–7761.

10	F. De Vega and Y. Sasson, Selective para-Bromination of Toluene catalysed by Na-Y Zeolite in the Presence of an Epoxide, *J. Chem. Soc. Chem. Commun.*, 1989, 653–654.

11	E. H. G. da Cruz, P. H. P. R. Carvalho, J. R. Corrêa, D. A. C. Silva, E. B. T. Diogo, J. D. de Souza Filho, B. C. Cavalcanti, C. Pessoa, H. C. B. de Oliveira, B. C. Guido, D. A. da Silva Filho, B. A. D. Neto and E. N. da Silva Júnior, Design, synthesis and application of fluorescent 2,1,3-benzothiadiazole-triazole-linked biologically active lapachone derivatives, *New J. Chem.*, 2014, **38**, 2569.

12	J. P. Heiskanen, P. Vivo, N. M. Saari, T. I. Hukka, T. Kastinen, K. Kaunisto, H. J. Lemmetyinen and O. E. O. Hormi, Synthesis of Benzothiadiazole Derivatives by Applying C-C Cross-Couplings, *J. Org. Chem.*, 2016, **81**, 1535–1546.

13	D. Taylor, T. Malcomson, A. Zhakeyev, S.-X. Cheng, G. M. Rosair, J. Marques-Hueso, Z. Xu, M. J. Paterson, S. J. Dalgarno and F. Vilela, 4,7-Diarylbenzo[ c ][1,2,5]thiadiazoles as fluorophores and visible light organophotocatalysts, *Org. Chem. Front.*, 2022, **9**, 5473–5484.

14	X. Zhang, H. Gorohmaru, M. Kadowaki and T. Kobayashi, Benzo-2,1,3-thiadiazole-based, highly dichroic fluorescent dyes for fluorescent host–guest liquid crystal displays, *J. Mater. Chem.*, 2004, 1901–1904.

15	P. H. Jun Zhang, Audun Konsmo, Alexander Sandberg, Xiongyu Wu, Sofie Nyström, Ulrike Obermüller, Bettina M. Wegenast-Braun, Peter Konradsson, Mikael Lindgren, Phenolic Bis-styrylbenzo[c]-1,2,5-thiadiazoles as Probes for Fluorescence Microscopy Mapping of Aβ Plaque Heterogeneity, *J. Med. Chem.*, 2019, **62**, 2038–2048.

16	P. K. Robinson, Enzymes: principles and biotechnological applications., *Essays Biochem.*, 2015, **59**, 1–41.

17	A. Cornish-Bowden, *Fundamentals of Enzyme Kinetics*, Wiley, 2012.

18	Symbolism and Terminology in Enzyme Kinetics Recommendations 1981, *Eur. J. Biochem.*, 1982, **128**, 281–291.
